# Fluoride-containing podophyllum derivatives exhibit antitumor activities through enhancing mitochondrial apoptosis pathway by increasing the expression of caspase-9 in HeLa cells

**DOI:** 10.1038/srep17175

**Published:** 2015-11-26

**Authors:** Wei Zhao, Yong Yang, Ya-Xuan Zhang, Chen Zhou, Hong-Mei Li, Ya-Ling Tang, Xin-Hua Liang, Tao Chen, Ya-Jie Tang

**Affiliations:** 1 Key Laboratory of Fermentation Engineering (Ministry of Education), Hubei Provincial Key Laboratory of Industrial Microbiology, Hubei Provincial Cooperative Innovation Center of Industrial Fermentation, Hubei University of Technology, Wuhan 430068 China; 2 State Key Laboratory of Oral Diseases West China Hospital of Stomatology (Sichuan University), Chengdu Sichuan 610041 China; 3 Key Laboratory of Systems Bioengineering (Ministry of Education), School of Chemical Engineering and Technology, Tianjin University, Tianjin 300072 China

## Abstract

This work aims to provide sampling of halogen-containing aniline podophyllum derivatives and their mode of action with an in-depth comparison among fluorine, chloride and bromide for clarifying the important role and impact of fluorine substitution on enhancing antitumor activity, with an emphasis on the development of drug rational design for antitumor drug. The tumor cytotoxicity of fluoride-containing aniline podophyllum derivatives were in general improved by 10–100 times than those of the chloride and bromide-containing aniline podophyllum derivatives since fluoride could not only strongly solvated in protic solvents but also forms tight ion pairs in most aprotic solvents. When compared with chloride and bromide, the higher electronegativity fluoride substituted derivatives significantly enhanced mitochondrial apoptosis pathway by remarkably increasing the expression of caspase-9 in HeLa cells. The current findings would stimulate an enormous amount of research directed toward exploiting novel leading compounds based on podophyllum derivatives, especially for the fluoride-substituted structures with promising antitumor activity.

Podophyllotoxin (PTOX, **1**) is a naturally occurring aryltetralin lignan first found in the roots of the *Podophyllum Peltatum Linnaeus*, which containing various other podophyllotoxin analogues have been used as folk remedies in traditional medicinal of many diverse cultures. Especially, extract of plants with high podophyllotoxin related contents was widely used in the Chinese, Japanese and the Eastern world folk medicine (even today in China, as Bajiaolian) as remedies for gout, tuberculosis, gonorrhoea, syphilis, menstrual disorders, dropsy, cough, psoriasis, venereal warts and certain tumours. Podophyllotoxin was first found to have the curative effect of inhibition of tumor growth in 1942 and is still accepted today as an effective treatment for chemotherapy of caners, especially cauliflower excrescence, melanoma, and oophoroma. As the representative of the bioactive natural leading compound, podophyllotoxin and its derivative 4′-demethylepipodophyllotoxin (DMEP, **1′**) are lignans of aryltetralin family found in the plants of podophyllum genus, exhibiting anti-tumor activity^1^. As a strong microtubule destabilizing agent, PTOX can bind to the colchicine site of tubulin to induce the cancer cell apoptosis^2^. Unlike that of PTOX, DMEP derivatives bind to the DNA topoisomerase II (Topo II)^3^, and the resulting DNA-strand breaks initiate multiple recombination/repair pathways and trigger cell death pathways^4^. The initial attempts with respect to the possible clinical utility of podophyllotoxin derivatives as antitumor agents largely have failed because of their side effects (e.g., liver injury, twitch, local inflammation, and arrest of bone marrow). Therefore, the remarkable biological activities and the very extensive usages (e.g., external application, oral) in traditional medicines make podophyllotoxin derivatives an important family of leading compound for the development of novel therapeutic agents based on structural modifications. Extensive structural modifications of podophyllotoxin have been undertaken, which culminated in the clinical introduction of semisynthetic glucoconjugate (e.g., GL331) analogues of etoposide^5^. Although the clinically important podophyllum anticancer drug etoposide is active in the treatment of many cancers (e.g., small cell and non-small cell lung cancer, testicular cancer, acute lymphoblastic leukemia) and is widely used in the chemotherapy, it presents several limitations, such as moderate potency, development of drug resistance, and toxic effects (e.g., anemia, myelosuppression, collapse, even death)^1^. Because of this, podophyllum compounds are still the hot research focus. In order to overcome the limitations described above, numerous studies on *Podophyllum* lignans currently focus on its structure modification of the cycloparaffin (C-ring) in the tetranap skeleton to generate derivatives with superior pharmacological profiles^1^.

Halogen-substituents, especially the fluorine and chlorine, have become a widespread and important drug component in the drug design^6^. Halogen for hydrogen substitution on aromatic rings of drugs affords compounds the carbon–halogen bonds which are catabolically more stable than the corresponding C–H bonds. Usually, halogen atoms in drugs or drug-like molecules are considered to be involved in non-directional hydrophobic interactions with target protein or just inserted into relatively empty protein spaces or cavities without major stabilizing contacts. However, since potential electron-rich sites such as oxygen, nitrogen, and sulfur atoms as well as aromatic p-electron systems are abundant in proteins, halogen atoms can also form, when structurally possible, stabilizing interactions through such as halogen bonds with the surrounding amino acids. Thus, halogen atoms can modulate the physicochemical properties to modify drug’s pharmacokinetics, such as improving the bioavailability, alter the conformation of a molecule to enhance the selectivity and binding affinity to the target proteins, and block metabolically labile sites to increase the metabolic stability of drugs^7^. The advantages have stimulated an enormous amount of research directed toward exploiting these properties, and the large inventory of synthetic fluorinated analogues continues to grow^8^. As electronegativities and hydrophobic moieties, fluorinated analogues often modified the compound in order to fill into empty hydrophobic cavities of the target protein, to prolong the lifetime of drugs and enhance membrane permeability^9^. Recently, the halogen bond, directional noncovalent interactions, began to attract great interest. It is a short-range R-X···Y-R′ interaction, driven by the σ-hole^10^. X is a halogen atom, acting as a Lewis acid, while Y is acts as a Lewis base, such as oxygen, nitrogen, or sulfur atoms^11^. Lee *et al.*^12^ reported design, synthesis, and biological testing of 2-Fluoropodophyllotoxin (11) and several 4beta-anilino-2-fluoro-4′-demethylpodophyllotoxin analogues. These compounds were moderately active against some cancer cell lines, but they were less active than the corresponding nonfluorinated analogues. Halogen bonds have been found to occur in a number of biological systems. Halogen bonds allow halogenated ligands tightly bound to the protein binding sites which formed in the binding pocket. These properties impart special advantages to the usage of halogen substitution in drug design.

Halogens are widely used substituents in medicinal chemistry based on hydrophobic moieties and Lewis bases in accordance with their electronegativities. However, the significance of different electronegativity of halogen atoms on the antitumor activity of the natural leading compounds still remains to be determined. For systematically comparing the effect of halogen (i.e., F, Cl, Br) on the antitumor activity of podophyllum derivatives and determining their precise apoptosis mechanism, a series of halogen-containing aniline podophyllum derivatives based on the structure of the target protein tubulin and topoisomerase II (Topo II) were designed. The process of drug molecular design was performed in three steps including link-bond design, substituents design, and virtual screening. According to bioisosterism^13^, the electron cloud of imino bond (-*NH*-) (the Pauling electronegativity scale was more than 4.00) with a higher electronegativity were denser than that of the oxygen atom (the Pauling electronegativity scale was about 3.44). Thus, the -*NH*- bond preferentially integrates with the large biological protein molecule with the hydrogen bond in the tumor cell. Aniline-derivatives such as 4β-(4-nitroanilino)-4′-demethylepipodophyllotoxin (GL331)^14^ and 4β-(4-fluoranilino)-4′-demethylepipodophyllotoxin (NPF)^15^ as belong to this category. It was found that the electron cloud of nitrogen atom with a higher electronegativity was denser than that of the oxygen atom. The C-NH bonds linkage between the substituent group and the C-4 position of PTOX and DMEP made a significant contribution for the antitumor proliferation activity of podophyllum derivatives. Aniline can form π-π packing with biomacromolecules, especially, halogen-substituted on aniline could change electrostatic potential mapped onto the electron density surface for highlighting the anisotropy of the electron density^6^. To halogen bonding acceptors, halogen atoms can provide a class of highly directional stabilizing contacts that can be systematically introduced at various positions of the skeleton to explore specific interactions of the halogen with active-site amino acid residues of the enzyme.

In the present work, by taking podophyllum compounds (**1** and **1′**) as model structures, the research is aiming to systematically investigate the effect of halogen atoms (i.e., F, Cl, Br) on the antitumor activity of podophyllum derivatives and identify their preliminary mechanism of apoptosis induction. These results would provide the determinants of tubulin and Topo II binding affinity for this important class of anti-tumor agents and pave the way for further rational structural modification.

## Results

### Halogen atom subsitituted podophyllum derivatives

**Figure 1:**
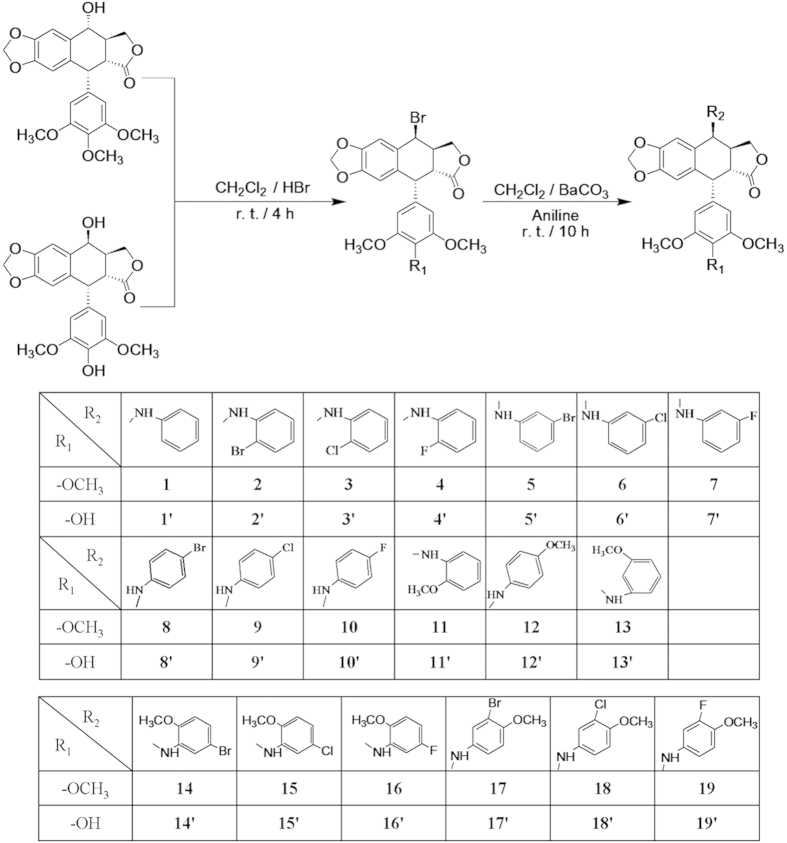
Synthesis of 4β-aniline derivatives of podophyllum derivatives from podophyllotoxin and 4′-demethylepipodophyllotoxin.

Compared with the ortho- (i.e., compound **1**–**3** and **1′**–**3′**) and para-substituted halogen (i.e., compound **8**–**10** and **8′-10′**), the cancer cell cytotoxicity of the meta-substituted halogen-containing aniline podophyllum derivatives (i.e., compound **5**–**7** and **5′**–**7′**) were in general improved by 4–7 times than those of the ortho- and para-substituted halogen aniline podophyllum derivatives. The meta-substituted halogen exhibited higher anti-tumor activity and was determined as a valid way of modification (Table 1). While, compared with the meta-substituted methoxy-containing aniline podophyllum derivatives (i.e., compound **13** and **13′**), the ortho- (i.e., compound **11** and **11′**) and para-substituted (i.e., compound **12** and **12′**) methoxy-containing aniline podophyllum derivatives exhibited higher anti-tumor activity (Table 1). So the ortho- and para-substituted methoxy was determined as another effective way of modification. Therefore, the advantages of substituted-positions of the halogen and methoxy were combined for the next chemical modification. In these efforts, twelve rationally designed 4*β*-*NH*-(halogen-methoxyaniline)-podophyllum derivatives were synthesized as target compounds (i.e., compound **14**–**19** and **14′**–**19′**) with the yields of 47%–81% (Fig. 1). The structures of all compounds were characterized by the combination evaluation of ^1^H NMR, ^13^C NMR, 2D NMR correlations (^1^H-^1^H COSY, HMQC and HMBC) and MS (see supporting information).

### Cytotoxicity assay

Compared with the clinically used podophyllum-derived anticancer drug etoposide, the antitumor activities of 4β-*NH*-(halogen-methoxyaniline)-podophyllum derivatives were in general significantly improved by the halogen bond introduction. Compared with 4β-*NH*-(3-bromide-4-methoxyaniline)-4-deoxy-podophyllotoxin (**17**, IC_50_ value of 1.52 ± 0.21 μM), 4β-*NH*-(3-chloride-4-methoxyaniline)-4-deoxy-podophyllotoxin (**18**, IC_50_ value of 5.92 ± 0.39 μM), and the clinically important podophyllum anticancer drug etoposide (IC_50_ value of 59.35 ± 2.45 μM), the IC_50_ value of 4β-*NH*-(3-fluoro-4-methoxyaniline)-4-deoxy-podophyllotoxin (**19,** IC_50_ value of 0.72 ± 0.08 μM) with the higher electronegativity fluoride against the tumor HeLa cell line significantly improved. (Table 2). Moreover, the cytotoxicity of synthesized compounds on human normal hepatic immortal cell line (HL-7702) decreased indicating the selectivity for cancer cells. Notably, some potential SARs could be deduced from these results. Firstly, halogen substituent was found to be beneficial for improving the anticancer activity, moreover, F-substituted derivative showed greater cytotoxicity than its corresponding analogues, and the Cl-substituted compounds showed greater cytotoxicity than that of Br-substituted compounds. This fact, for example, could be observed from the comparison of cytotoxicity effects among the series of **17**, **18**, **19**, 4β-*NH*-(3-bromide-4-methoxyaniline)-4-deoxy-4′-demethylepipodophyllotoxin **(17′)**, 4β-*NH*-(3-chloride-4-methoxyaniline)-4-deoxy-4′-demethylepipodophyllotoxin **(18′)**, and 4β-*NH*-(3-fluoro-4-methoxyaniline)-4-deoxy-4′-demethylepipodophyllotoxin **(19′)**. For aniline-substituted podophyllum derivatives, as the higher electronegativity of fluoride was adding, the anti-tumor activity was more potential. The above results demonstrated that most of the halogen modification was beneficial to enhance the antitumor activity and reduce the cytotoxicity to normal cells. In addition, fluoride modified aniline-substituted podophyllum derivatives was better than that of chloride and bromide in the inhibition of proliferation against cancer cells.

### Cell Cycle Arrest

**Figure 2:**
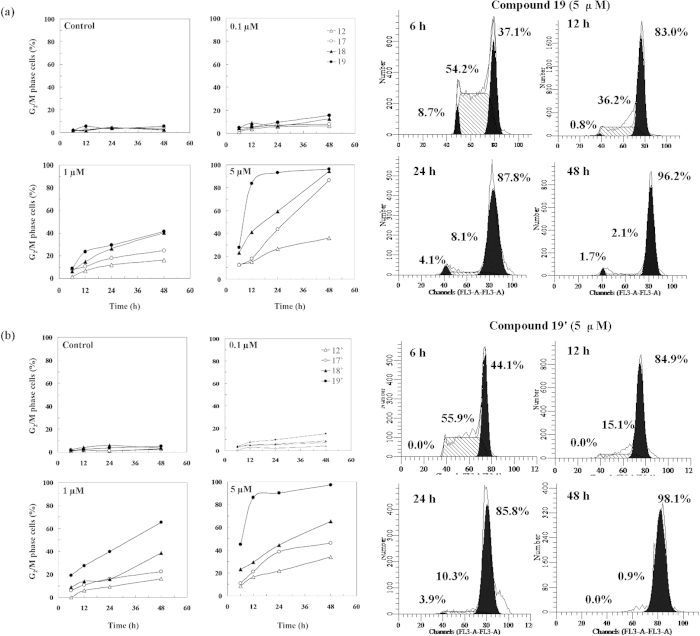
Effects of podophyllum derivatives on the HeLa cell cycle arrest. (**a**) 4β-*NH*-(2-anisidine)-4-deoxy-podophyllotoxin (**12**), 4β-*NH*-(3-bromide-4-methoxyaniline)-4-deoxy-podophyllotoxin **(17)**, 4β-*NH*-(3-chloride-4-methoxyaniline)-4-deoxy-podophyllotoxin **(18)**, and 4β-*NH*-(3-fluoro-4-methoxyaniline)-4-deoxy-podophyllotoxin **(19)** arrested cell cycle in HeLa cells in a dose- and time-dependent manner. (**b**) 4β-*NH*-(2-anisidine)-4-deoxy-4′-demethylepipodophyllotoxin (**12**′), 4β-*NH*-(3-bromide-4-methoxyaniline)-4-deoxy-4′-demethylepipodophyllotoxin **(17**′), 4β-*NH*-(3-chloride-4-methoxyaniline)-4-deoxy-4′-demethylepipodophyllotoxin **(18**′), and 4β-*NH*-(3-fluoro-4-methoxyaniline)-4-deoxy-4′-demethylepipodophyllotoxin **(19**′) arrested cell cycle in HeLa cells in a dose- and time-dependent manner.

The ratio of HeLa cells in each phase of cell cycle was determined after the incubation of 6, 12, 24, and 48 h at a concentration of 0.1, 1.0, 5.0 μM podophyllotoxin derivatives 4β-*NH*-(3-anisidine)-4-deoxy-podophyllotoxin (**12**), **17**, **18**, **19**, and 4′-demethylepipodophyllotoxin derivatives 4*β*-*NH*-(3-anisidine)-4-deoxy-4′-demethylepipodophyllotoxin (**12′**)**, 17′, 18′, 19′** (Fig. 2). Cell cycle analysis illustrated that cells were arrested at the G_2_/M phase following the treatment with compounds **12, 17**, **18**, **19, 12′, 17′, 18′,** and **19′**. All designed compounds had dose- and time-dependent activity on G_2_/M phase of cell cycle arrest and induced cell apoptosis at a high concentration of 1.0 μM after 12 h (Fig. 2). When Hela cells were treated by podophyllotoxin derivatives **12**, **17**, **18**, and **19** or 4′-demethyepipodophyllotoxin derivatives **12**′**, 17**′, **18**′, and **19**′ at a low concentration of 0.1 μM for 6, 12, 24, and 48 h, the ratio of cell cycle arrest was less than 10% of the total cells indicating there was no significantly cell cycle arrest. While, when drug concentration increased from 0.1 to 5.0 μM, the percentage of cell cycle arrest for compound **19** (Fig. 2a) and **19′** (Fig. 2b) were 83.0% and 84.9% at 12 h, respectively, and then maintained a stable from 24 h to 48 h. The total cell cycle arrest percentage of **12, 17**, **18**, and **19** was 35.0%, 86.5%, 94.4% and 96.2% under the treatment of 5.0 μM of podophyllotoxin derivatives for 48 hours, respectively. These results indicated that the effects of the higher electronegativity of haloid Compound **19** (Fig. 2a) and **19**′ (Fig. 2b) were more significant than that of compound **17** and **18** on the HeLa cells G_2_/M phase arrest at a moderate concentration of 1.0 μM after the treatment of 12 to 48 h.

### Cell Apoptosis

**Figure 3:**
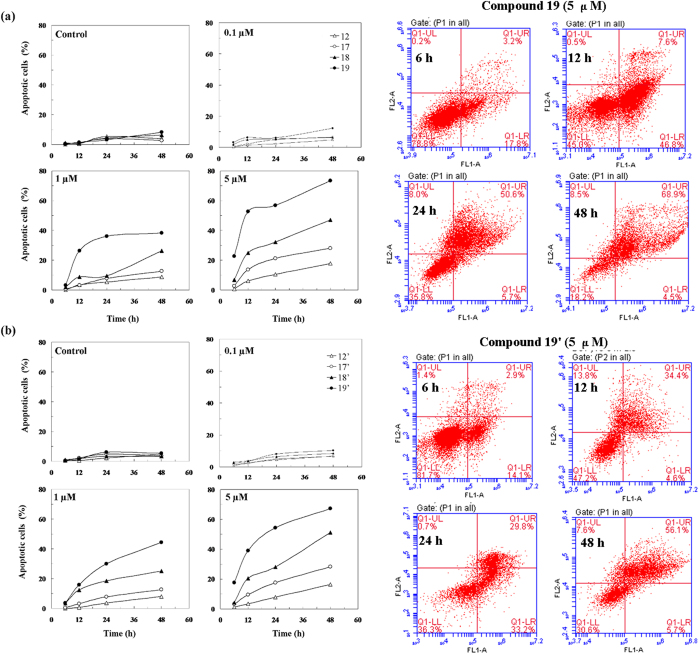
Effects of podophyllum derivatives dose and time on the HeLa cell apoptosis. (**a**) 4β-*NH*-(2-anisidine)-4-deoxy-podophyllotoxin (**12**), 4β-*NH*-(3-bromide-4-methoxyaniline)-4-deoxy-podophyllotoxin **(17)**, 4β-*NH*-(3-chloride-4-methoxyaniline)-4-deoxy-podophyllotoxin **(18)**, and 4β-*NH*-(3-fluoro-4-methoxyaniline)-4-deoxy-podophyllotoxin **(19)** reduced apoptosis in HeLa cells in a dose- and time-dependent manner. (**b**) 4β-*NH*-(2-anisidine)-4-deoxy-4′-demethylepipodophyllotoxin (**12**′), 4β-*NH*-(3-bromide-4-methoxyaniline)-4-deoxy-4′-demethylepipodophyllotoxin **(17**′), 4β-*NH*-(3-chloride-4-methoxyaniline)-4-deoxy-4′-demethylepipodophyllotoxin **(18**′), and 4β-*NH*-(3-fluoro-4-methoxyaniline)-4-deoxy-4′-demethylepipodophyllotoxin **(19**′) reduced apoptosis in HeLa cells in a dose- and time-dependent manner.

**Figure 4:**
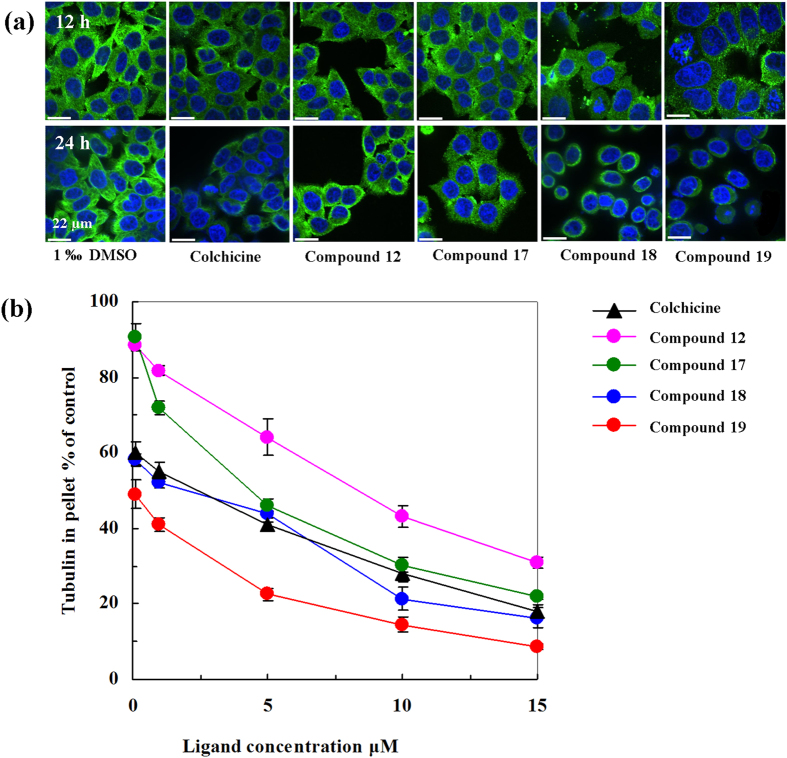
(**a**) Effects of colchicine, 4β-*NH*-(2-anisidine)-4-deoxy-podophyllotoxin (**12**), 4β-*NH*-(3-bromide-4-methoxyaniline)-4-deoxy-podophyllotoxin **(17)**, 4β-*NH*-(3-chloride-4-methoxyaniline)-4-deoxy-podophyllotoxin **(18)**, and 4β-*NH*-(3-fluoro-4-methoxyaniline)-4-deoxy-podophyllotoxin **(19)** on the tubulin polymerization in HeLa cells. Microtubules (green) were stained with α-tubulin antibodies, and DNA (blue) was stained with DAPI for 12 and 24 h. Insets were mitotic spindles from the same preparation. The scale bar represents 22 μm. (**b**) Inhibition of tubulin assembly *in vitro* by colchicine, compounds 12, 17–19.

To check whether synthesized compounds exhibiting antiproliferative activity induce the apoptosis of HeLa cells, an annexin V-FITC/propidium iodide (PI) binding assay was performed (see Supporting information). Induction of apoptosis was measured by Annexin-V/PI double-staining assay after treatment with compounds **12, 17**, **18**, **19, 12′, 17′, 18′,** and **19′** at the concentration of 0.1, 1.0, 5.0 μM for 6, 12, 24, 48 h. Results showed that the ratio of apoptotic Hela cells was less than 15% of the total cells and there was no significant cell apoptosis by using a low concentration of 0.1 μM podophyllotoxin derivatives **12, 17**, **18**, and **19** (Fig. 3a) and 4′-demethylepipodophyllotoxin derivatives **12′, 17′, 18′,** and **19′** (Fig. 3b) for 6, 12, 24, and 48 h . At the highest concentration of 5.0 μM, time-dependent relation was more significant. With the higher electronegativity fluoride modification, **19** fairly induced apoptosis more effective than **17** and **18** at 48 h.

### Microtubule Assembly Inhibition

Tubulin-targeting natural products and their synthetic derivatives have been widely used in cancer chemotherapy. Kita *et al.* found that human anti-phospholipid antibody (ApA) synergistically bound to tubulin in association with actin, inhibited tubulin polymerization, and prevented spindle formation and mitosis in tumor cells by using fluorescence microscopy observations and photoaffinity-tag approaches^16^. As a strong microtubule destabilizing agent, podophyllotoxin can bind to the colchicine site of tubulin^2^. Therefore, the effect of podophyllotoxin derivatives on microtubule stability and distribution in cultured HeLa cells was evaluated as well. Colchicine, as a well-known tubulin destabilizer, was used as positive control at the same concentration and 0.1% DMSO as negative control. When compared with the negative control, all studied compounds (**12**, **17**, **18** and **19**) were able to cause cellular depolymerization of microtubules, but with a fair difference in potency (Fig. 4a). Compared with the 12 h treatment, all studied compounds (**12**, **17**, **18** and **19**) with 24 hours of treatment were stronger cause cellular depolymerization of microtubules. Microtubules were greatly disrupted and disappeared by the treatment of compound **18** and **19**. Furthermore, the degree of tubulin polymerization was evaluated through pellet mass formation in centrifugation assays in the presence of stoichiometric and semi-stoichiometric concentrations of each lignan. Inhibition curves were used to determine GI_50_, which is the concentration that causes 50% growth inhibition. Inhibition of cellular microtubule polymerization shown in Fig. 4b, Compound 18 and 19 displayed effects higher than colchicine, used as positive control, especially, compound 19 exhibited strongest microtubule depolymerization (GI_50_ <1 μM). The results indicated that most of designed compound had stronger ability to promote microtubule depolymerization than colchicines and compound **19** with the higher electronegativity fluoride modification was observed to fairly inhibit microtubule formation more effective than **17** and **18**.

### Inhibition of Topoisomerase II

**Figure 5:**
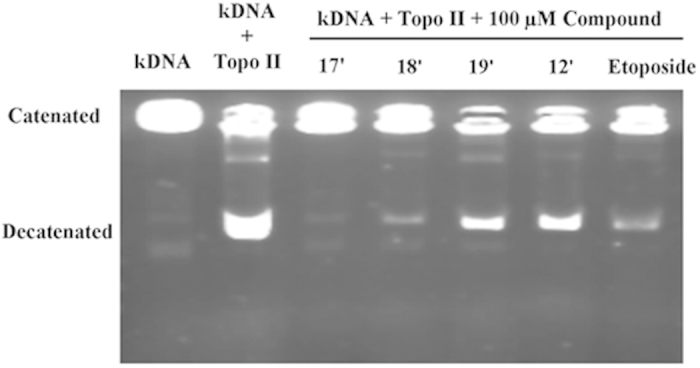
Inhibition of topoisomerase II catalytic activity by 4β-*NH*-(2-anisidine)-4-deoxy-4′-demethy-lepipodophyllotoxin (12′), 4β-*NH*-(3-bromide-4-methoxyaniline)-4-deoxy-4′-demethylepipodo-phyllotoxin (17′), 4β-*NH*-(3-chloride-4-methoxyaniline)-4-deoxy-4′-demethylepipodophyllotoxin (18′), and 4β-*NH*-(3-fluoro-4-methoxyaniline)-4-deoxy-4′-demethylepipodophyllotoxin (19′). lane 1, kDNA without adding topoisomerase II (Topo II); lane 2, kDNA plus 5 units of topoisomerase II; lanes 3–7, DNA plus 5 units of topoisomerase II in the presence of 100 μM Compounds **12′, 17′~19′**, and etoposide. All reaction samples were electrophoresed in 1% agarose gels, stained with ethidium bromide, and photographed under UV light as described in the Topo II mediated DNA relaxation assay.

Unlike the inducing cell apoptosis mechanism of podophyllotoxin, 4′-demethylepipodophyllotoxin could bind to the DNA topoisomerase II (Topo II) and the resulting DNA strand breaks would accordingly initiate multiple recombination/repair pathways and trigger cell death pathways^17^. Thus, DNA fragmentations, induced by 4′-demethylepipodophyllotoxin derivatives VP-16 due to the topoisomerase II inhibition, could be used a typical biochemical hallmark^3^. To evaluate the effect of synthesized compounds on Topo II decatenation activity, the kDNA decatenation assay has been utilized for the representative compounds **12′, 17′, 18′,** and **19′** (Fig. 5). Etoposide was employed as a positive control. Compounds **17′** completely inhibited the catalytic activity of Topo II at the concentration of 100 μM, which was much more effective than that of etoposide at the same concentration. The results show that the Topo II inhibition activity of compounds **17′, 18′,** and **19′** has regularity: as in the case of **17′**, such higher electronegativity of haloid could further enhance Topo II inhibition activities of aniline-substituted 4′-demethylepipodophyllotoxin derivatives. However, the molecular basis of the haloid enhancement of Topo II inhibition of these dual-acting conjugates is not entirely clear. The result indicated that most of designed compound had stronger ability to inhibit the activity of Topo II than etoposide. The higher electronegativity halogen modification **17′** was fairly inhibited Topo II more effective than **18′, 19′** and **12′**.

### Apoptosis Pathway Detection

**Figure 6:**
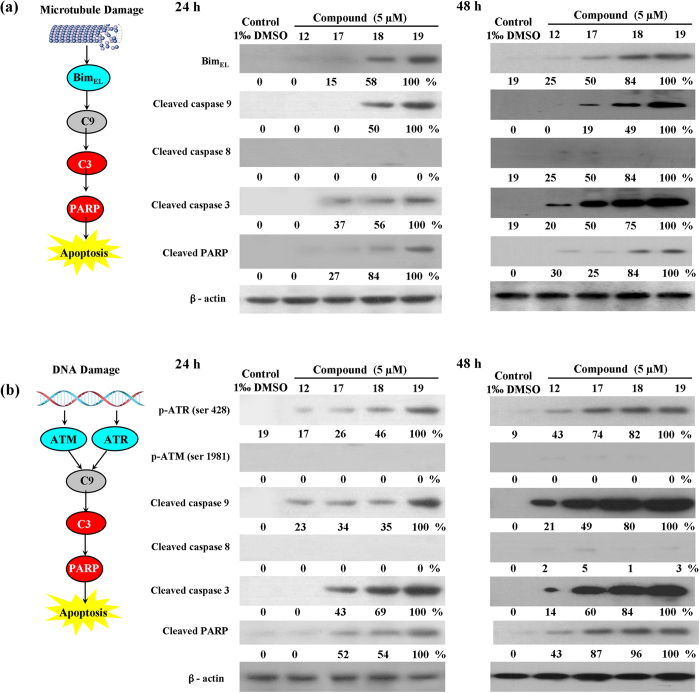
Detection of apoptosis protein in HeLa cells by using Western blot analysis. (**a**) Effects of 4β-*NH*-(3-bromide-4-methoxyaniline)-4-deoxy-podophyllotoxin **(17)**, 4β-*NH*-(3-chloride-4-methoxyaniline)-4-deoxy-podophyllotoxin **(18)**, and 4β-*NH*-(3-fluoro-4-methoxyaniline)-4-deoxy-podophyllotoxin **(19)** with an adding concentration of 5 μM on the levels of Bim, Caspase-9, Caspase-8, Caspase-3, PARP for 48 h. (**b**) Effects of 4β-*NH*-(3-bromide-4-methoxyaniline)-4-deoxy-4′-demethylepipodophyllotoxin **(17**′), 4β-*NH*-(3-chloride-4-methoxyaniline)-4-deoxy-4′-demethylepipodophyllotoxin **(18**′), and 4β-*NH*-(3-fluoro-4-methoxyaniline)-4-deoxy-4′-demethylepipodophyllotoxin **(19**′) with an adding concentration of 5 μM on the levels of ATR, ATM, Caspase-9, Caspase-8, Caspase-3, PARP for 24 and 48 h.

**Figure 7:**
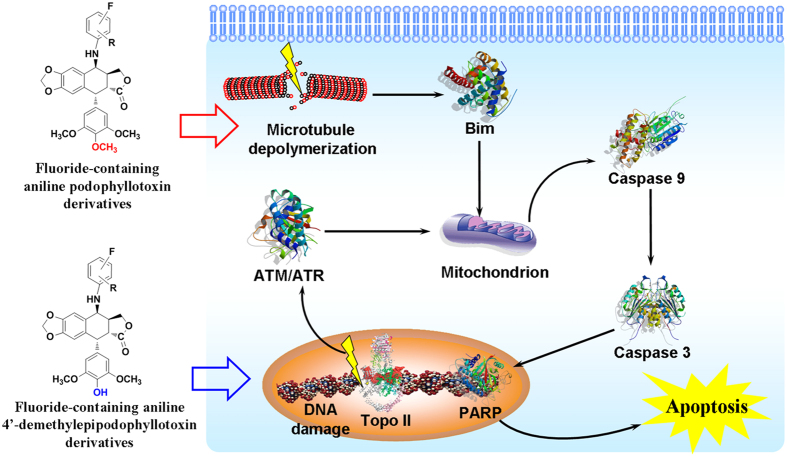
The integrated apoptotic pathways a schematic diagram showing some of the known components of the intrinsic and the death receptor apoptotic programs and the mitochondrial apoptotic pathways. Antitumor mechanistic studies performed with carbon-sulfur and carbon-amine bond modification podophyllum compounds showed that the mitochondrial apoptotic pathway was activated by the halogen-containing aniline podophyllum derivatives.

Caspase plays a key role in the apoptotic response. Its activation by specific signals triggers proteolysis of cellular substrates thereby executes apoptotic events^18^. There are two main apoptotic pathways, namely the extrinsic pathway that involves membrane-bound death receptors leading to the activation of caspase-8 and the mitochondria-related caspase-9-dependent intrinsic pathway. Both pathways converge onto the effectors caspase-3 activated^19^. However, whether the difference between two pathways is cell-specific or compound dependent remains unclear. In the present study, the details of apoptosis induction by compounds **12, 17**~**19, 12′, 17′~19′** were studied in HeLa cells. PTOX could bind to the colchicine site of tubulin, and, Bim_EL_, a pro-apoptotic protein significantly increased after treatment with compounds 18 and 19 after 24 h treatment. And the extents of compound 17-induced Bim_EL_, caspase 3, caspase 9 and PARP were 15%, 37%, 0% and 27%, respectively, after 24 h treatment. The extents of compound 18-induced Bim_EL_, caspase 3, caspase 9 and PARP were 58%, 56%, 50% and 84%, respectively, after 24 h treatment. 4β-*NH*-(fluoride-methoxyaniline)-podophyllum derivatives 19 have higher Bim_EL_, caspase 3, caspase 9 and PARP phosphorylation ability remarkably than chlorine and bromine substituted compound 17 and 18. The expression of total caspase 8 remains substantially unchanged, after 24 and 48 h treatments of compounds 12, 17~19. Compared with the data of 24 h treatment with compounds, compounds 12, 17~19 exhibited continuously phosphorylated Bim_EL_, caspase 3, caspase 9 and PARP protein. Compound 17 may induce MMP decreased by enhancing combinations of free tubulin and VDAC phosphorylation (Fig. 6a). Homoplastically, 4′-demethylepipodophyllotoxin could bind to the DNA topoisomerase II (Topo II) and the resulting DNA strand breaks initiate multiple recombination/repair pathways and can trigger cell death pathways. Therefore, ATR (Ser 15), a serine/threonine-specific protein kinase that is involved in sensing DNA damage and activating the DNA damage checkpoint were significantly increased after treatment with compounds **12′, 17′~19′**. And the extents of compound **17′**-induced ATR, caspase 3, caspase 9 and PARP were 26%, 43%, 34% and 52%, respectively, after 24 h treatment. The extents of compound **18′**-induced ATR, caspase 3, caspase 9 and PARP were 46%, 69%, 35% and 54%, respectively, after 24 h treatment. 4β-*NH*-(fluoride-methoxyaniline)-podophyllum derivatives **19′** have higher ATR, caspase 3, caspase 9 and PARP phosphorylation ability remarkably than chlorine and bromine substituted compound **17′** and **18′**. Compared with the data of 24 h treatment with compounds, compounds **12′, 17′~19′** exhibited continuously phosphorylated ATR, caspase 3, caspase 9 and PARP protein. The expression of total ATR and caspase 8 remains substantially unchanged, after 24 and 48 h treatments of compounds **12′, 17′~19′** (Fig. 6b). Furthermore, all compound treatment generated an active caspase-3 by the cleavage of procaspase-3, which was further confirmed by the observation of the PARP cleavage, a downstream target of caspase-3 (Fig. 7). These results suggested that podophyllotoxin derivatives **17**~**19** and 4′-demethylepipodophyllotoxin derivatives **17′~19′** could induce HeLa cells apoptosis by activating the caspase-9-dependent intrinsic pathway. Notably, no matter compounds **17**~**19** or **17′~19′**, as the higher electronegativity of haloid was introduced, the higher levels of active forms of caspase-9 and caspase-3 formation were.

## Discussion

Compared with the clinically important podophyllum anticancer drug etoposide, the antitumor activity of the novel podophyllum compounds exhibited promising *in vitro* antitumor activity, especially halogen-containing aniline podophyllum derivatives modified by fluoride were significantly improved. The correctness of drug design and structure-function relationship was strictly demonstrated by the biological activity tests. In the drug design process, halogen substituents are often introduced to lead compounds in order to provide van der Waals contacts with hydrophobic residues or fill empty hydrophobic cavities in the target protein, as well as to improve drug lifetime and membrane permeability. As a result, a large proportion of drugs or drug candidates are halogenated. Being a halogen atom with electron withdrawing property in nature, fluoride is commonly regarded as a negative site around which the partial negative charge is uniformly distributed. However, theoretical and experimental data have shown that, in halocarbons, the actual charge distribution on the halogen atom does in fact correspond to the formation of a small, positively charged area at the tip of the C–X bond extension (the so-called “σ-hole”) and the consequent lateral accumulation of the partial negative charge to form an equatorial belt on the halogen atom, coaxial with the C–X bond. Therefore, halogen substituents can potentially form roughly linear, stabilizing halogen bonds with electron-donors, the strength of the interaction generally increasing with the size and polarizability of the halogen atom itself. Substitution of H by F can profoundly change the conformational preferences of a small molecule because of size and stereoelectronic effects. A comparison between methoxyphenyl and benzene halide groups illustrates the influence of F on conformation. In order to gain insight into the stability and dynamics properties of the complexes, studies were performed on the basis of a model of interaction complex between tubulin and podophyllotoxin derivatives or Topo II and 4′-demethylepipodophyllotoxin derivatives reported by **17**~**19** or **17′~19′**. Docking of 4′-demethylepipodophyllotoxin derivatives led to energetically and geometrically adequate results for all those analyzed compounds was shown in Fig. 1S and Table 3. In order to facilitate a more complete comparison, ability to dock, hydrogen bonds,π-π bonds and cellular cytotoxicity for representative compounds were summarized in the current study. Comparable experimental and calculated data for most compounds seem to be in global agreement, with some exceptions that could be interpreted taking into consideration the expected influences of the different electronegative halogen-substituents and the degree of site occupation on the respective docking energy. Higher electronegative halogen-substituents can form more hydrogen bond as well as π-π bond with tubulin or Topo II. Moreover, methoxy groups lie in the plane of the phenyl ring because the p orbital of the sp^2^-hybridized O is in p conjugation with the aromatic p system. Orienting F bonds antiperiplanar to the lone pairs of the now sp^3^-hybridized O results in an anomeric n_O_–σ*_F_ conjugation with concomitant lengthening of the F bonds^20^. This effect enhance the conjugation between F and the aromatic p system and eliminates the energetic preference of a planar, in-plane conformation. Fluorine introduction also strongly reduces amine basicity, impacting membrane permeability^21^ and the potential liability for phospholipidosis^22^. In relation to the antineoplastic cytotoxicity of aniline-substituted podophyllum derivatives (Table 2), it had already been recognized that the anti-tumor activity could be improved with the addition of halogen. Significantly, as the higher electronegativity of haloid is bearing with, the anti-tumor activity is more potential than their corresponding analogues. This fact would indicate that the electronegativity of haloid of aniline-substituted podophyllum derivatives at C-4 should have more importance for the activity than the nature or type of function located at that position. This statement would be reinforced by the higher cytotoxicity of Hela cells showed by compound **19**, with an aromatic group at C-4. However, taking into account advantageous effects on physicochemical properties, an overall benefit may well result from the decoration of ligands with fluorobenzene residues to occupy apolar aromatic pockets. The assays and studies that focused on the mechanism of action of these series of compounds have demonstrated a global parallelism between cytotoxicity, cell cycle arrest apoptosis, tubulin polymerization inhibition from the colchicine binding site, (Figs 2, 3, 4) and Topo II inhibition from the etoposide binding site (Fig. 5) by these tested compounds. Additionally the compounds assayed behaved similarly to podophyllotoxin in arresting the cellular cycle of HeLa cells at the G_2_/M phase, with differences in potency (Fig. 4). Chemists have known about the inductive effects of fluoride for decades from small molecule studies, such as Hammett linear free-energy relationships. Moreover, the capacity of fluoride to enhance metabolic stability has become increasingly clear recently^22^. In contrast, the understanding of how fluoride affects binding affinity and selectivity at the molecular level is lacking. It is becoming clear that F can enhance binding efficacy and selectivity in pharmaceuticals. As small atoms of high electronegativity, F substituents on ligands prefer to orient toward electropositive regions of receptor sites. Thus, halogen-containing aniline-substituted podophyllum derivatives obtained from podophyllotoxin structure retain or even enhance the global antimitotic or antitubulin properties of podophyllotoxin and that the other main series, whose compounds belong to the epipodophyllotoxin series, were evaluated to be fairly more potent.

This work expanded the role of fluorine in drug development and design for enhancing antitumor activity by systematically comparing the antitumor activity and mechanism of fluoride, chloride, and bromide modified podophyllum derivatives, which was a representative of the bioactive natural leading compound. Fluorine can modulate the physicochemical and pharmacokinetic properties to improve bioavailability, alter the conformation of a molecule to enhance the selectivities and binding affinity to target proteins, and block metabolically labile sites to increase the metabolic stability of drugs. The active pockets of Topo II and tubulin suggested that the docking of podophyllotoxin and 4′-demethylepipodophyllotoxin derivatives led to interaction the Asn residue at β-tubulin interface and the WHD active region in Topo II. Most of the fluoride-containing aniline podophyllum derivatives exhibited much higher cytotoxic potency than that of lower electronegativity halogen (i.e., Cl, Br) against cancer cell lines. Compound **19**, higher electronegativity haloid pendants enhanced caspase-dependent apoptosis mediated by the intrinsic caspase-9-dependent apoptotic pathway, enhanced the apoptosis of HeLa cells due to super inhibition activities of tubulin. The study provided a useful strategy to introduce fluorine and fluorinated substitutes in the small molecule for structure-based medicinal chemistry and provided a powerful direction in the discovery of potential the fluoride-containing aniline podophyllum tubulin and Topo II inhibitors with superior anti-tumor activity.

## Methods

### Chemistry

All reagents and solvents were purchased from commercial sources and were used as received unless otherwise specified. ^1^H, ^13^C NMR, COSY, HMQC, and HMBC were recorded on Bruker AC 300 (300 MHz) and Bruker DRX 400(400 MHz) instruments with TMS as the internal standard. Silica gel 60 (Hai Yang, 230–400 mesh) was used for flash chromatography. Precoated silica gel plates (Merck, Kieselgel 60 F254, 0.25 mm) were used for TLC analysis. For EIMS analysis, an Agilent-TS250 mass spectrometer (70 eV) was used. Before biological testing, compound purity was evaluated by reversed-phase HPLC. HPLC analysis was performed using an Dionex 3000 series equipped with a Synergy Max-RP C18, 250 mm × 4.6 mm column, with gradient H_2_O + 0.1% TFA/CH_3_CN + 0.1% TFA from 45% to 85% organic in 45 min and from 85% to 100% organic in 5 min, a flow rate of 0.8 mL/min, and UV detection at 210 nm. From HPLC data, the percentage purity is given for each compound. All biologically evaluated compounds are >95% chemically pure as measured by HPLC.

### General procedure for the synthesis of 4-bromo-4-desoxypodophyllum derivatives

A solution of podophyllotoxin (800 mg, 2 mmol) or 4′-demethylepipodophyllotoxin (828 g, 2 mmol) in 25 mL of dry dichloromethane was refluxed for 1 h, and dry hydrogen bromide gas was bubbled through the solution, kept at 0 °C and evaporated to dryness. In most cases, the resulting products (3) were obtained with >88% purity as judged by HPLC analysis.

### General procedure for the synthesis of products (4)

A solution containing 4-bromo-podophy-llotoxin (477mg, 1 mmol) or 4-bromo-4′-demethylepipodophyllotoxin (465 mg, 1 mmol), anhydrous barium carbonate (196 mg, 1 mmol), and the appropriate arylamine (1 mmol) in 25 mL of dry dichloromethane was stirred overnight at room temperature. The reaction mixture was filtered, dried over anhydrous magnesium sulfate, and purified via column chromatography and pre-HPLC. The ^1^H and ^13^C NMR and ESI-MS data of Compound 1–19 and 1′–19′ structure were all shown in supplementary information.

### Cytotoxicity Assays

HeLa, BGC823, A-549, MCF-7 and HT-7702 cell lines were obtained from the ATCC. Cell lines were maintained in DMEM medium supplemented with 12% fetal calf serum (FCS), 2 mM/L -glutamine, 100 mg/L penicillin G and 100 mg/L streptomycin at 37 °C and 5% CO_2_ in a humidified atmosphere. Tumor cells (3500–13,000) were seeded into 96-well microtest plates in 100 μL of culture medium per well, incubated for 48 h in the presence or absence of test compounds (8 different concentrations ranging from 100 to 0.01 μg/mL). For quantitative estimation of cytotoxicity, the (MTT) method was used, essentially performed as described previously. Briefly, cells were washed twice with PBS, fixed for 15 min in 1% glutaraldehyde solution, rinsed twice in PBS, and stained in 0.5% MTT solution for 4 h at 37 °C. Drug stock solutions were prepared in DMSO, and the final solvent concentration was ≤2% DMSO (v/v), a concentration that does not affect cell replication. The initial seeding densities varied among the cell lines to ensure a final absorbance reading in control (untreated) cultures within the range of 0.6–0.8 *A*_492_ units. Drug exposure was carried out for 2 days, and the IC_50_ value, i.e., the drug concentration that decreased the absorbance by 50%, was extrapolated from dose-response data. Each test was performed in triplicate, and the absorbance readings between the triplicates varied by no more than 10%.

### Cell Cycle Analysis

The HeLa cell line was used for cell cycle analysis. Progression through the cell cycle was assessed by flow cytometry DNA determination with Propidium Iodide (PI). Cells (200000 per mL) were incubated with several concentrations of the compounds or drugs for 6–48 h, and incubated in DMEM medium supplemented with 12% fetal calf serum (FCS), 2 mM/L -glutamine, and 100 mg/L penicillin G and 100 mg/L streptomycin at 37 °C and 5% CO_2_. The cells were washed with PBS twice, centrifuged at 206 g for 5 min, and 5–10^5^ cells were collected and fixed with 70% ethanol for 4 h, treated with RNase, and stained with PI. Flow cytometric analysis was performed using a BD accur C6 flow cytometer. Analysis was with Mod FIT software.

### Cell apoptosis analysis

The HeLa cell line was used for cell apoptosis. Cells (20000 per mL) were incubated with several concentrations of the compounds or drugs for 6–48 h, and incubated in DMEM medium supplemented with 12% fetal calf serum (FCS), 2 mM L -glutamine, and 100 mg/L penicillin G and 100 mg/L streptomycin at 37 °C and 5% CO_2_. The cells were washed with PBS twice, centrifuged at 206 g for 5 min, and 5–10^5^ cells were collected. Binding buffer suspension (500 μL) was added to the cells, and then 5 μL of the FITC-Annexin V mix was added. Next, 10 μL of the PI mix was added, and the suspension was mixed and kept at room temperature for 30 min in the dark. Analysis was with a BD accur C6 flow cytometer.

### Inmunofluorescence

HeLa cells were continuously maintained in DMEM medium supplemented with 12% fetal calf serum (FCS), 2 mM/L -glutamine, and 100 mg/L penicillin G and 100 mg/L streptomycin at 37 °C and 5% CO_2_. Cells (20000 per mL) were plated a onto 6-well tissue culture plates containing 12 mm round coverslips, cultured overnight, and then treated with different drugs at 5 mM concentrations or drug vehicle (0.1% DMSO) for 12 h and 24 h. Attached cells were permeabilized. Cytoskeletons were incubated with α-tubulin, washed twice, and incubated with FITC goat anti-mouse immunoglobulins. The coverslips were washed, and 1 μg/mL DAPI to stain chromatin was added. The mixture was incubated for 30 min. After the samples were washed, they were examined and photographed using an Olympus epifluorescence microscope. The images were recorded with a Perkin Elmer camera.

### Tubulin Assembly

Ligands were dissolved in DMSO at 20 mM and kept at −80 °C. Work solutions were done in DMSO and kept at −20 °C. The 50% inhibitory ligand concentration of tubulin assembly was determined with a centrifugation assay. Tubulin was equilibrated prior to use in 3.4 M glycerol, 1 mM EGTA, 0.1 mM GTP, pH 7.0, buffer through a 25 cm × 0.9 cm Sephadex G-25 column. Aggregates were removed by a centrifugation at 90000 g × 10 min in a TLA 120 rotor at 4 °C in an Optima TLX centrifuge. Tubulin concentration was determined as described by Andreu^42^. Tubulin was kept at 4 °C, and 0.9 mM GTP and 6 mM MgCl_2_ were added to the sample. The solution was distributed in 200 μL polycarbonate tubes for the TL100 rotor. Growing concentrations of the ligands ranging from 0 to 25 μM were added to the samples (DMSO content of the samples, 2.5%), which were incubated for 30 min at 37 °C. Microtubules were separated from unassembled tubulin by a centrifugation at 90000 g × 10 min in a TLA100 rotor at 37 °C in an Optima TLX centrifuge. The supernatant containing unassembled tubulin was carefully collected and the microtubule pellet resuspended in 10 mM sodium phosphate buffer, pH 7.0, containing 1% SDS. Both supernatants and pellets were diluted 1:5 in the same buffer, and tubulin concentrations were measured fluorometrically (λexc = 280; λems = 323) using tubulin standards calibrated spectrophotometrically. The 50% inhibitory ligand concentration of tubulin assembly was determined with a centrifugation assay that measured the decrease in the concentrations of microtubules assembled in the presence of different concentrations of the compound.

### kDNA Decatenation Assay

The decatenation of kDNA was assayed according to TopoGen protocol in order to determine topoisomerase II activity. The substrate kDNA (200 ng) and 100 μM drugs were combined in assay buffer and incubated for 10 min on ice. Next, 1 U of topoisomerase II was added and the reaction was allowed to proceed for 15 min at 37 °C. The reaction was quenched via the addition of loading buffer (1% sarkosyl, 0.025% bromophenol blue, and 5% glycerol) and was then analyzed by electrophoresis on a 1% agarose gel in TBE buffer for 30 min at 130 V. The gel was stained with SYBR Green I (Molecular Probes) for 30 min and was visualized under UV illumination and photographed on a Tanon Imager.

### Western blot analysis

For electrophoresis, the proteins were separated by sodium dodecyl sulfate–polyacrylamide gel electro-phoresis (SDS–PAGE). The proteins were then transferred to a nitrocellulose membrane, which was blocked with 5% skimmed milk in phosphate buffered saline Tween-20 (PBST). A specific primary antibody was added to bind the target proteins for either 1 h at room temperature or overnight at 4 °C. A horseradish peroxidase (HRP) conjugated secondary antibody was added to the membrane after the primary antibody was washed off. All signals were detected after the HRP was activated by enhanced chemiluminescence. The band intensities of Western results were quantitated with the Image Quant program (Molecular Dynamics Inc.), analyzed and graphed. The untreated controls and the etoposideinduced activation of signaling molecules were taken as 0% and 100%, respectively. Statistic analysis was performed with the uni-polar, paired Student t-test. Data were considered significant when P value was less than 0.05.

### Molecular docking

The docking mode was based on the tubulin-colchicine complex structure (PDB code: 1SA1) and topoisomerase II-DNA-VP-16 complex structure (PDB code: 3QX3) and Discovery Studio 3.0 was used for molecular docking. Water molecules, co-crystallized ligand and ions were removed, all hydrogen atoms were added, the Gasteiger charges were calculated, and nonpolar hydrogen atoms were merged with the carbon atoms. Then, based on the Discovery Studio 3.0, a docking procedure was applied to position the conformation of these compounds correctly with regard to their active sites. The others docking parameters were set as default. During docking, a maximum number of 100 conformers were considered.

## Additional Information

**How to cite this article**: Zhao, W. *et al.* Fluoride-containing podophyllum derivatives exhibit antitumor activities through enhancing mitochondrial apoptosis pathway by increasing the expression of caspase-9 in HeLa cells. *Sci. Rep.* **5**, 17175; doi: 10.1038/srep17175 (2015).

## Supplementary Material

Supplementary Information

## Figures and Tables

**Table 1:** The IC_50_ values of 4β*-NH*-(aniline)-podophyllum derivatives 1–13 and 1′-13′.

Compounds	Cytotoxic activity (IC_50_, μM)^a^				
	HeLa^b^	BGC-823^b^	A549^b^	MCF-7^b^	HL-7702^b^
**1**	35.13 ± 1.03	86.46 ± 4.21	43.24 ± 3.32	37. 57 ± 1.35	41.79 ± 2.47
**2**	26.64 ± 2.03	17.86 ± 0.98	35.13 ± 2.37	29.14 ± 1.78	65.87 ± 2.37
**3**	26.71 ± 1.32	18.06 ± 1.03	27.21 ± 1.95	21.07 ± 0.76	13.18 ± 1.35
**4**	22.07 ± 0.81	22.72 ± 0.97	16.80 ± 1.22	16.14 ± 0.17	15.95 ± 0.66
**5**	9.01 ± 0.11	11.17 ± 3.61	13.4 ± 2.54	8.35 ± 0.42	13.58 ± 0.11
**6**	5.43 ± 0.27	9.32 ± 1.79	7.23 ± 0.95	6.57 ± 0.15	35.27 ± 0.21
**7**	3.12 ± 0.18	5.21 ± 1.32	6.80 ± 0.31	4.05 ± 0.26	29.86 ± 0.74
**8**	42.14 ± 0.91	46.24 ± 2.97	52.9 ± 4.36	19.74 ± 0.62	27.14 ± 2.31
**9**	21.32 ± 0.11	23.08 ± 1.74	22.76 ± 1.08	19.32 ±1.43	41.57 ± 3.11
**10**	17.51 ± 0.04	16.98 ± 0.18	12.08 ± 0.63	7.06 ± 0.37	27.41 ± 1.98
**11**	13.37 ± 1.31	14.18 ± 0.98	14.05 ± 0.37	10.24 ± 0.61	37.49 ± 1.75
**12**	51.32 ± 2.13	46.84 ± 0.87	31.32 ± 2.73	97.38 ± 1.37	25.32 ± 2.67
**13**	11.64 ± 0.83	12.67 ± 1.06	12.41 ± 0.38	13.84 ± 3.98	38.78 ± 2.37
**1′**	44.34 ± 0.34	68.15 ± 4.13	42.47 ± 5.11	26.75 ± 0.67	53.41 ± 4.34
**2′**	31.31 ± 1.43	28.71 ± 1.04	44.89 ± 3.12	27.64 ± 0.91	47.42 ±3.14
**3′**	23.42 ±1.40	23.07 ± 1.87	20.46 ± 0.42	37.53 ± 1.03	2.42 ± 0.71
**4′**	20.73 ± 0.75	17.81 ± 0.34	15.58 ± 1.75	15.32 ± 0.42	31.47 ± 2.81
**5′**	8.83 ± 0.12	13.58 ± 0.16	11.85 ± 0.74	10.74 ± 1.48	44.32 ± 1.78
**6′**	5.13 ± 0.05	6.59 ± 0.21	5.03 ± 1.07	4.75 ± 0.37	32.45 ± 0.86
**7′**	5.07 ± 0.31	2.24 ± 0.06	3.88 ± 0.33	3.14 ± 0.15	21.45 ± 1.57
**8′**	21.07 ± 0.17	62.14 ± 4.39	62.37 ± 3.66	28.89 ± 1.06	32.13 ± 0.96
**9′**	16.74 ± 1.31	14.27 ± 1.14	29.81 ± 0.75	37.68 ± 0.16	15.83 ± 1.17
**10′**	16.32 ± 0.09	28.61 ± 0.06	17.73 ± 0.47	9.84 ± 0.73	29.58 ± 2.33
**11′**	13.46 ± 1.38	9.68 ± 0.17	8.68± 0.29	9.42 ± 0.24	63.57 ± 0.19
**12′**	24.46 ± 0.16	36.01 ± 3.21	22.27 ± 1.21	18.75 ± 0.24	28.64 ± 2.35
**13′**	12.31 ± 0.74	13.82 ± 1.42	7.27 ± 2.38	11.04 ± 1.26	39.77 ± 2.73
Etoposide	59.38 ± 2.45	25.46 ±1.42	20.12 ± 1.95	25.29 ± 1.93	24.61 ± 3.82

^a^MTT methods, drugs exposure was for 48 h.

^b^Standard deviation (SD) of triplicate samples was calculated from three independent samples, mean ± SD (n = 3).

**Table 2:** The IC_50_ values of 4β-*NH*-(halogen-methoxyaniline)-podophyllum derivatives 14–19 and 14′-19′.

Compounds	Cytotoxic activity (IC_50_, μM)^a^				
	HeLa^b^	BGC-823^b^	A549^b^	MCF-7^b^	HL-7702^b^
14	7.07 ± 0.63	6.26 ± 0.36	6.85 ± 0.75	4.97 ± 0.03	87.62 ± 4.13
**15**	2.64 ± 0.04	3.86 ± 0.29	1.71 ± 0.26	2.24 ± 0.14	19.34 ± 1.34
**16**	1.46 ± 0.19	1.37 ± 0.17	1.43 ± 0.09	1.92 ± 0.23	27.68 ± 0.77
**17**	5.92 ± 0.39	7.91 ± 0.59	9.08 ± 0.26	10.14 ± 0.18	62.44 ± 1.96
**18**	1.52 ±0.21	3.52 ± 0.23	3.72 ± 0.29	3.54 ± 0.14	78.97 ± 2.33
**19**	0.72 ± 0.08	1.61 ± 0.11	1.76 ± 0.07	2.01 ± 0.21	55.41 ± 0.64
**14′**	6.47 ± 0.17	22.17 ± 2.02	54.28 ± 4.49	4.21 ± 0.25	87.62 ± 5.04
**15′**	5.89 ± 0.48	27.12 ± 1.17	29.72 ± 1.67	2.16 ± 0.37	67.58 ± 4.32
**16′**	1.72 ± 0.30	1.32 ± 0.16	3.17 ± 0.49	1.53 ± 0.07	24.34 ± 0.69
**17′**	7.89 ± 0.31	5.63 ± 0.12	5.21 ± 0.41	6.51 ± 0.37	91.82 ± 1.57
**18′**	1.97 ± 0.16	1.77 ± 0.23	3.04 ± 0.23	3.18 ± 0.51	127.39 ± 0.46
**19′**	0.56 ± 0.12	0.40 ± 0.07	1.20 ± 0.25	1.32 ± 0.24	187.64 ± 7.33
Etoposide	59.38 ± 2.45	25.46 ± 1.42	20.12 ± 1.95	25.29 ± 1.93	24.61 ± 3.82

^a^MTT methods, drugs exposure was for 48 h.

^b^Standard deviation (SD) of triplicate samples was calculated from three independent samples, mean ± SD (n = 3).

**Table 3:** Calculated docking of the complex of podophyllum derivatives with tubulin/Topo II.

Compounds	CDOCKER Energy (kcal/mol)	H-bonds	π-π Packing	Compounds	CDOCKER Energy	H-bonds	π-π Packing
14	−8.80	Leu 225	–	14′	−3.77	DG 7	DT 9
15	−0.26	Thr 179	Thr 179, Leu 248	15′	−3.84	–	2Arg 503
16	−2.97	–	Lys 352	16′	−2.38	–	DC 8, Arg 503
17	6.48	Thr 179	Lys 352	17′	7.94	2Asp 479, DG 13	Arg 503, DA 12, DG 13
18	7.13	2Thr 353	Leu 248	18′	9.63	2Asp 479, DG 13	Arg 503, DA12, DG 13
19	9.78	2Gln 11, Thr 179	Thr 179, Lys 352	19′	10.05	Asp 479, DG 13	Arg 503, DT 9, DA12, DG 13
PTOX	4.11	Leu 248	Ala 316	VP-16	6.38	Asp 479	Arg 503, DC 8, DG 13
