# Supplementary material for: Fluoride-containing podophyllum derivatives exhibit antitumor activities through enhancing mitochondrial apoptosis pathway by increasing the expression of caspase-9 in HeLa cells
Source: Sci Rep. 2015 Nov 26;5:17175. doi: 10.1038/srep17175 (PMC4660440; doi:10.1038/srep17175)
Supplement: Supplementary Information [file srep17175-s1.pdf]

Fluoride-containing podophyllum derivatives exhibit antitumor activities through enhancing mitochondrial apoptosis pathway by increasing the expression of caspase-9 in HeLa cells

Wei Zhao [1, a], Yong Yang [1, a], Ya-Xuan Zhang [1, a], Chen Zhou [a], Hong-Mei Li [a], Ya-Ling Tang [b], Xin-Hua Liang [b], Tao Chen [c], and Ya-Jie Tang [a] *

[a] *Key Laboratory of Fermentation Engineering (Ministry of Education), Hubei Provincial Cooperative Innovation Center of Industrial Fermentation, Hubei University of Technology, Wuhan 430068 China*

[b] *State Key Laboratory of Oral Diseases West China Hospital of Stomatology (Sichuan University), Chengdu Sichuan 610041 China*

[c] *Key Laboratory of Systems Bioengineering (Ministry of Education), School of Chemical Engineering and Technology, Tianjin University, Tianjin 300072 China*

*Corresponding author. Tel. & Fax: +86-27-5975.0491 Email: yajietang@hotmail.com

[1] Equally contributed to this work

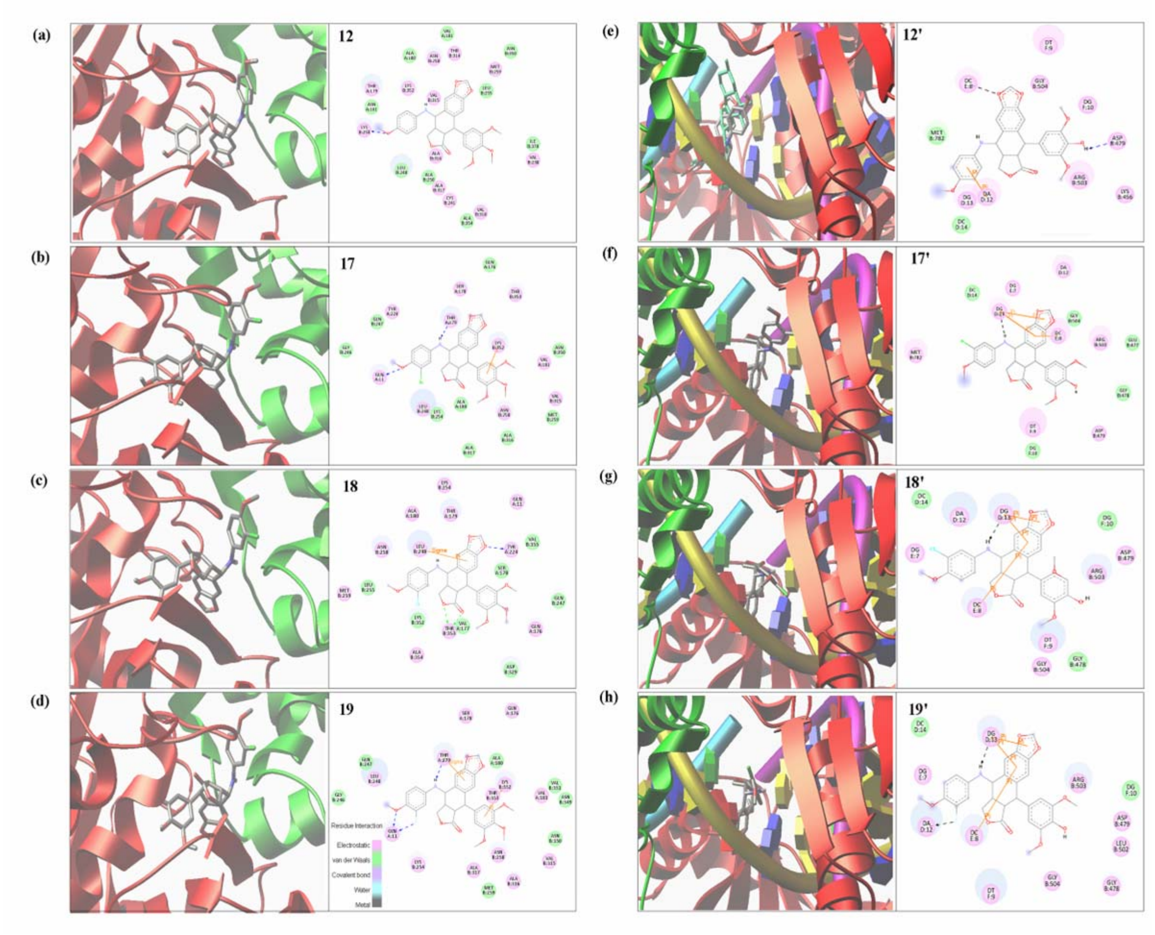

Figure [1S] Structure of the tubulin and topoisomerase II cleavage complex stabilized by substituted podophyllum derivatives. Schematic representation of the interactions between protein and compounds interactions between ligand atoms and protein residues are marked with lines: the black dotted lines were H-bonds to the protein; green dotted lines were Van der Waals' force to the protein; the orange yellow solid lines were π-π stacking interactions to the protein.

NMR and MS spectrum of compounds.

^{13}C NMR spectrum of 4*β*-*NH*-(aniline)-4-deoxy-podophyllotoxin **(1)**.

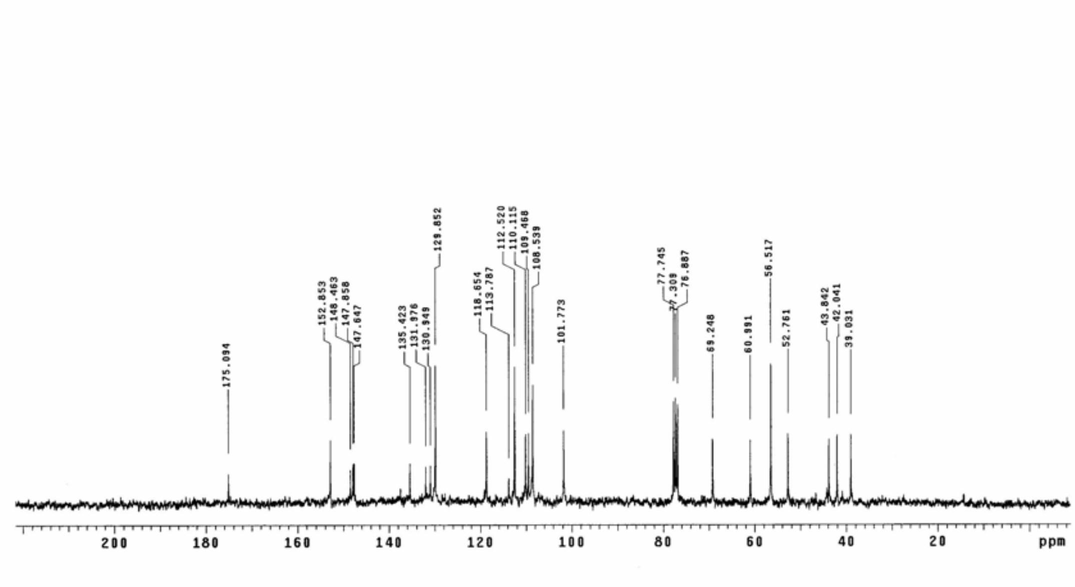

^{1}H NMR spectrum of Compound **1**.

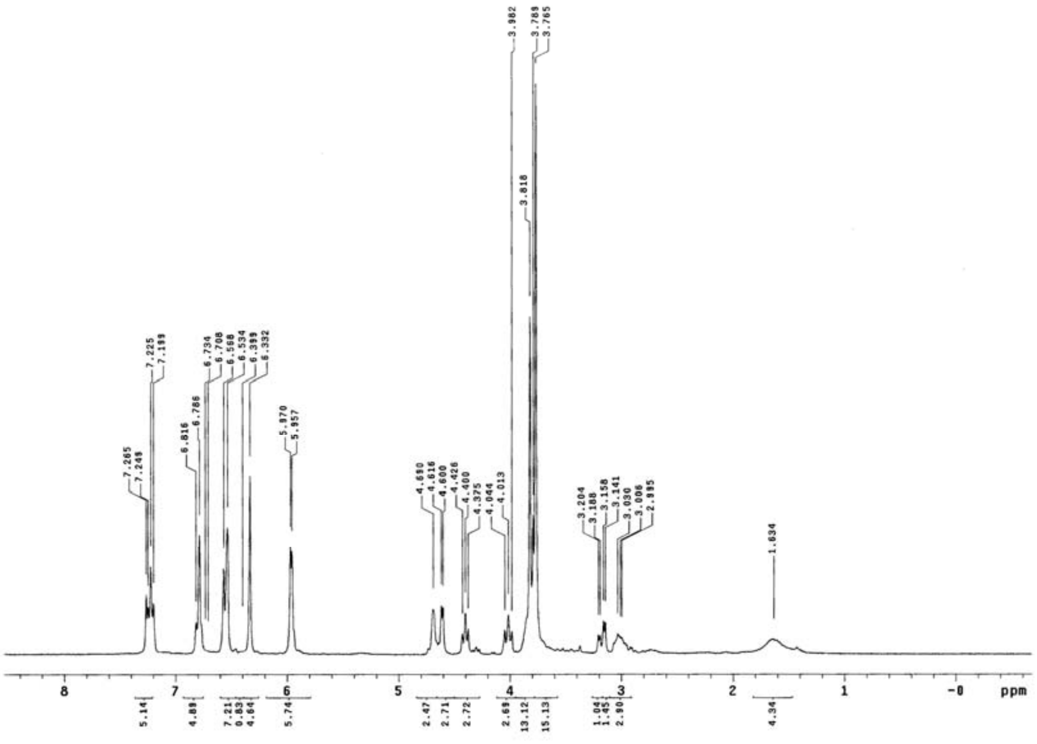

HMBC spectrums for Compound **1**.

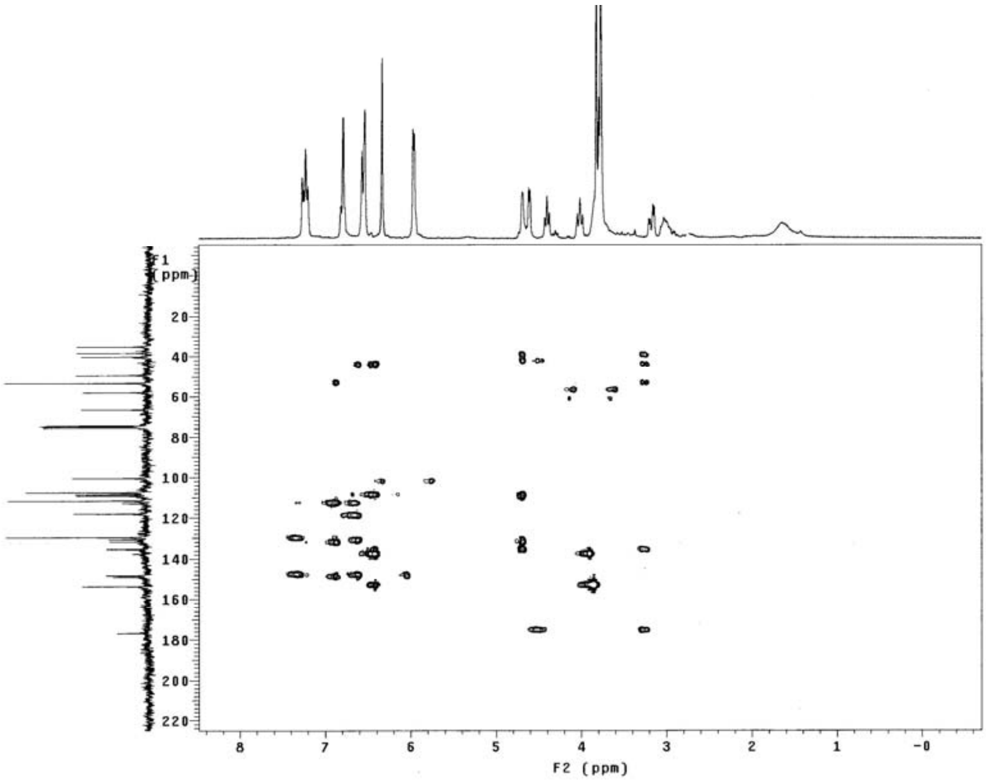

HSQC spectrums for Compound **1**.

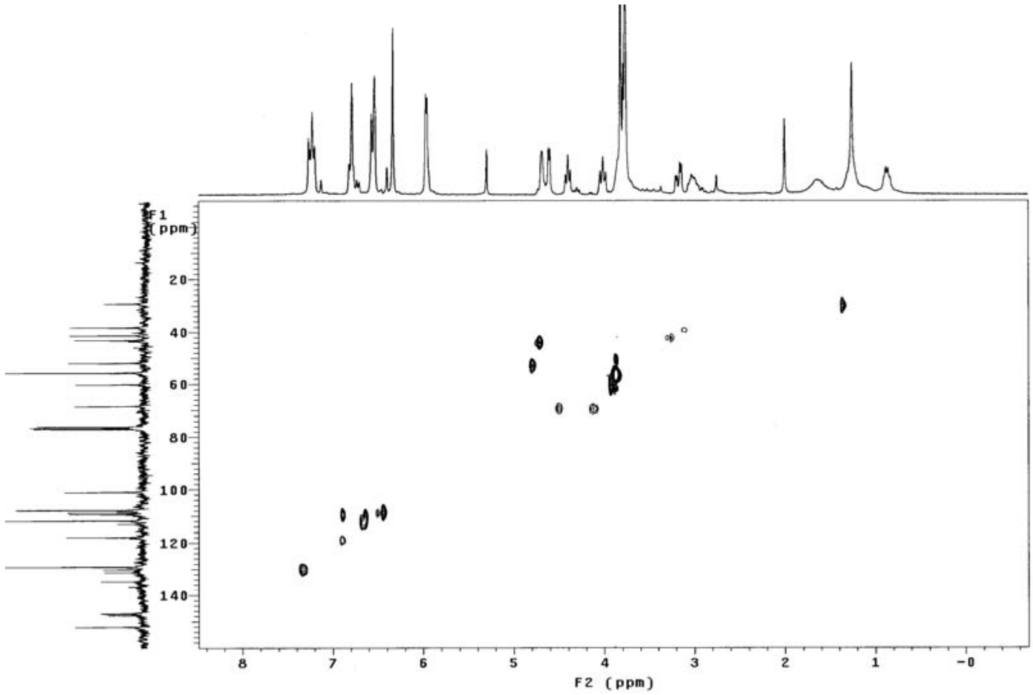

^{1}H-^{1}H COSY spectrums for Compound **1**.

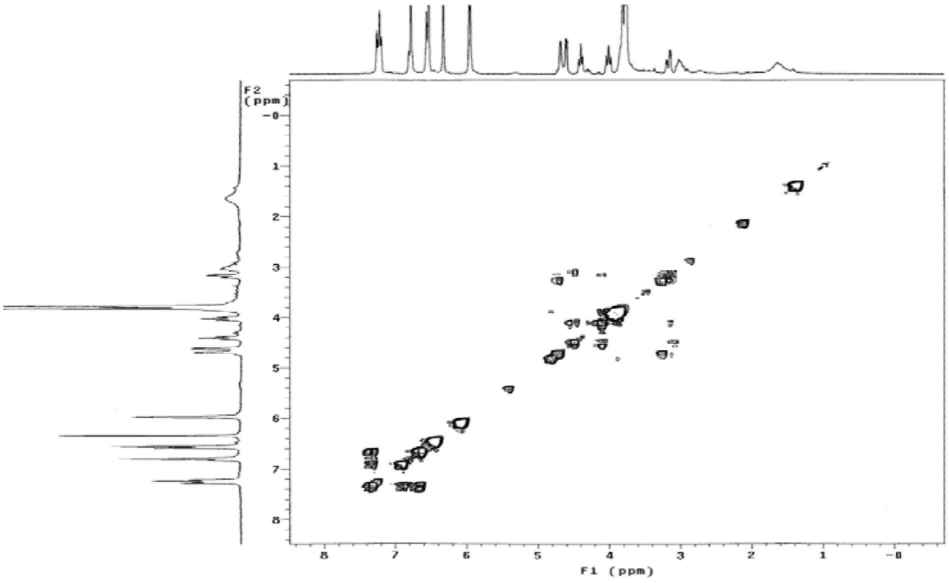

MS spectrums for Compound **1**

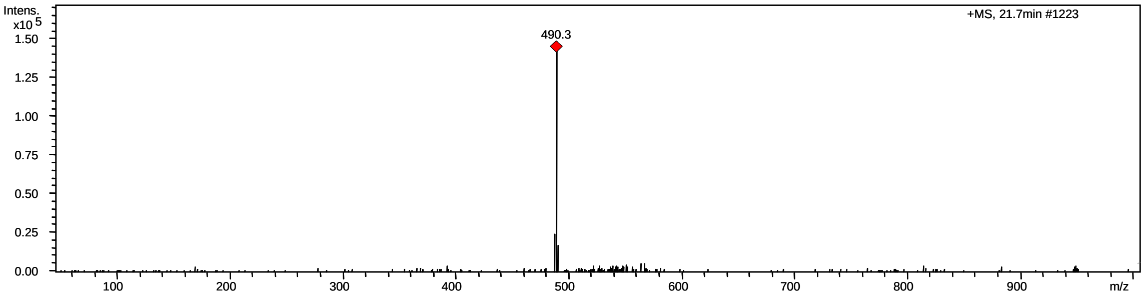

Compound 1 (4β-*NH*-(aniline)-4-deoxy-podophyllotoxin): 75% yield as white solid, ^{1}H NMR (300 MHz, $CDCl_3$): δ 2.995 (m, 1H, 2-H), 3.141 (dd, *J*=4.8 Hz, 1H, 3-H), 3.765 (s, 6H, 3′, 5′-OCH_3), 3.818 (s, 3H, 4′-OCH_3), 4.013 (t, *J*=9.3 Hz, 1H, 11-H), 4.400(t, *J*=7.8 Hz, 1H, 11-H), 4.600 (d, *J*=4.8 Hz, 1H, 1-H), 4.690(s, 1H, 4-H,) 5.957 (d, *J*=3.9 Hz 2H, OCH_2O), 6.332 (s, 2H, ArH) , 6.534 (s, 3H, ArH), 6.786 (s, 2H, ArH), 7.225 (m, 2H, ArH) ^{13}C NMR (100 MHz, $CDCl_3$): δ 39.031 , 42.014, 43.842, 52.761, 56.517, 60.991, 69.248, 101.773, 108.539, 109.468, 110.115, 112.520, 113.787, 118.654, 129.852, 130.949, 131.976, 135.423, 147.647, 147.858, 148.463, 152.853 175.904; MS (ESI): m/z: 490 $[M+H]^+$

^{13}C NMR spectrum of Compound 4*β*-*NH*-(2-chloroaniline)-4-deoxy-podophyllotoxin **(3**)

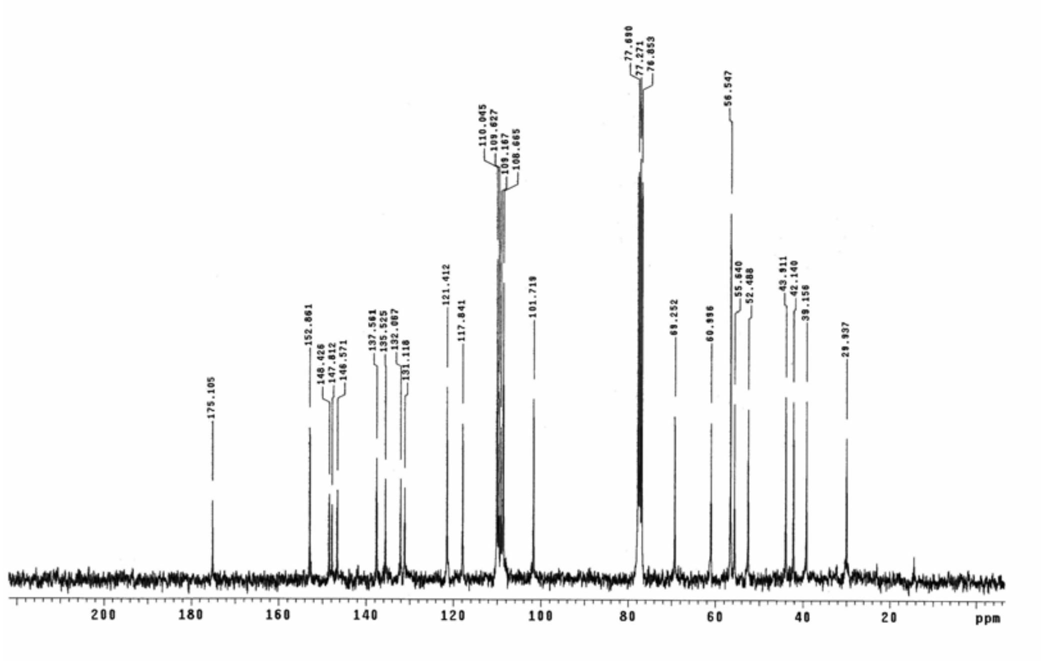

^{1}H NMR spectrum of Compound **3**.

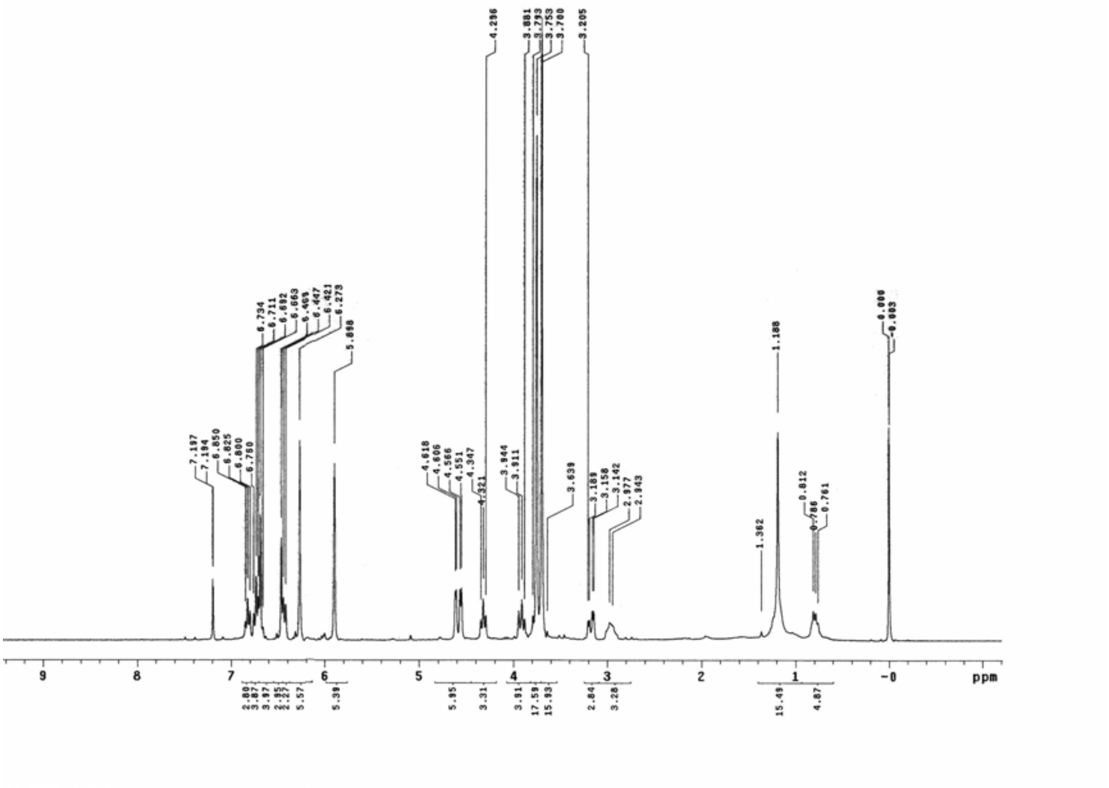

HMBC spectrums for Compound **3**.

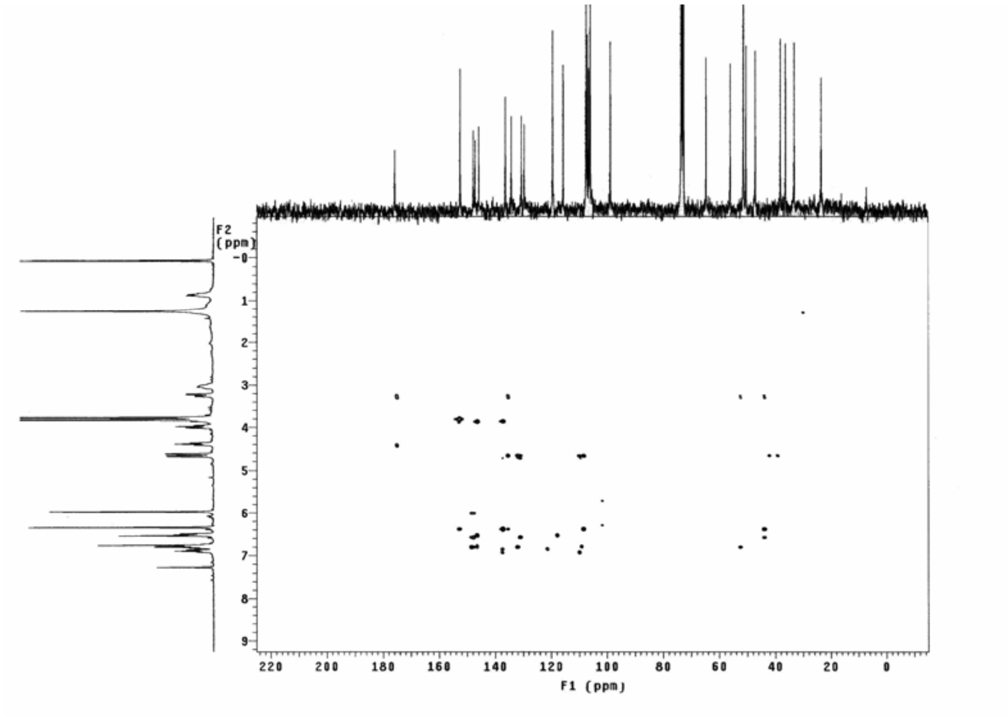

HSQC spectrums for Compound **3**.

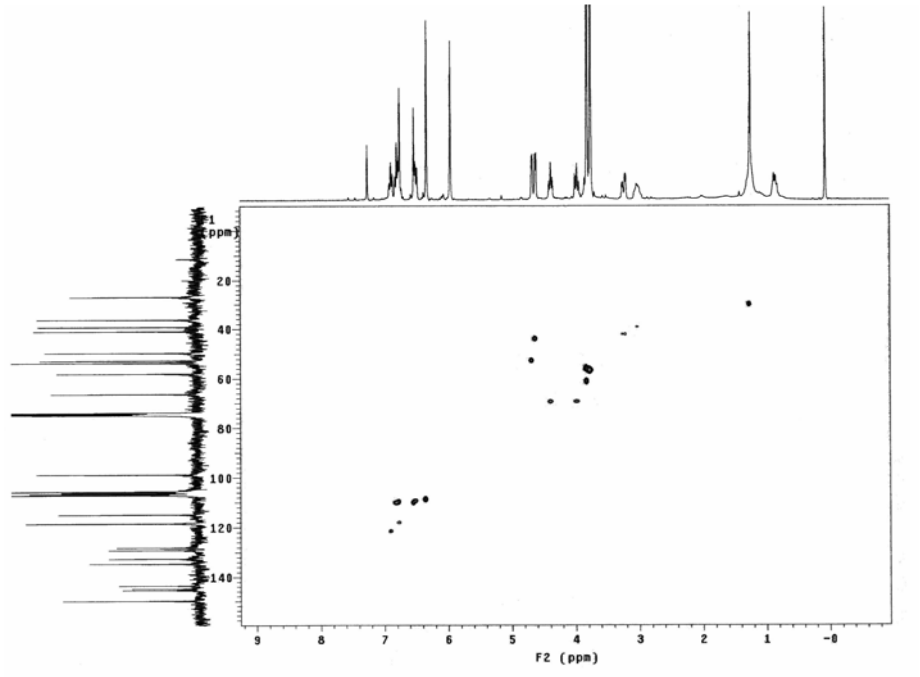

^{1}H-^{1}H COSY spectrums for Compound **3**.

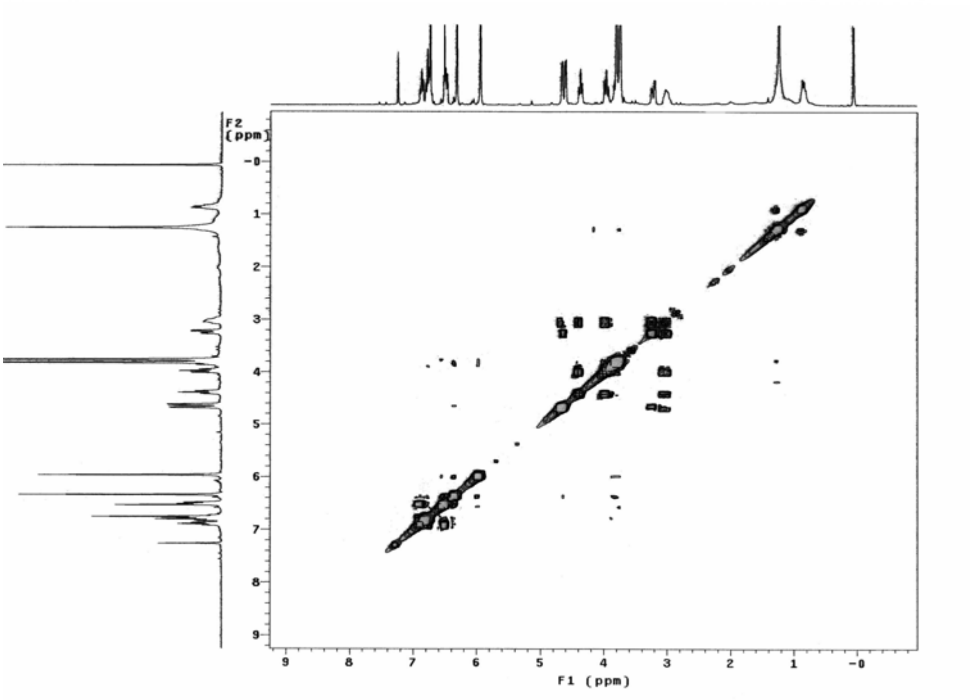

1.3.6. MS spectrums for Compound **3**.

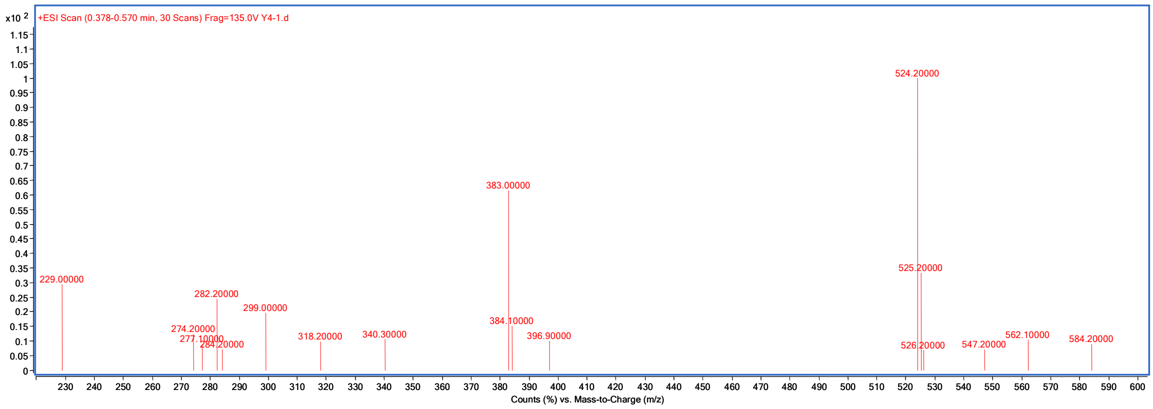

Compound 3 (4β-*NH*-(2-chloroaniline)-4-deoxy-podophyllotoxin): 82% yield as white solid, ^{1}H NMR (400 MHz, CDCl3): 2.992 (m, 1H, 2-H), 3.082 (dd, *J*=4.8 Hz, 1H, 3-H), 3.753 (s, 6H, 3′, 5′-OCH3), 3.794(s, 3H, 4′-OCH3), 3.949 (t, *J*=8.8 Hz, 1H, 11-H), 4.390 (t, *J*=7.6 Hz, 1H, 11-H), 4.571 (t, *J*=4.8 Hz, 1H, 4-H), 4.666 (s,1H, 1-H), 5.929 (d, *J*=9.2 Hz 2H, OCH2O), 6.313(s, 2H, ArH), 6.420 (d, *J*=1.6 Hz, 1H, ArH) , 6.513 (s, 1H, ArH), 6.540 (s, 1H, ArH), 6.723 (m, 2H, ArH), 7.092(m, 1H, ArH) ^{13}C NMR (75 MHz, CDCl3): δ 38.576 , 41.749, 43.535, 52.343, 56.218, 60.737, 68.775, 101.592,108.148, 109.185, 109.897, 110.599, 111.973, 118.255, 130.109, 130.629,

131.785, 135.049, 135.331, 137.629, 147.629, 148.290,148.473, 152.579, 174.701; MS (ESI): m/z: 524 $[M+H]^+$

^{13}C NMR spectrum of 4*β*-*NH*-(2-fluoroaniline)-4-deoxy-podophyllotoxin **(4)**

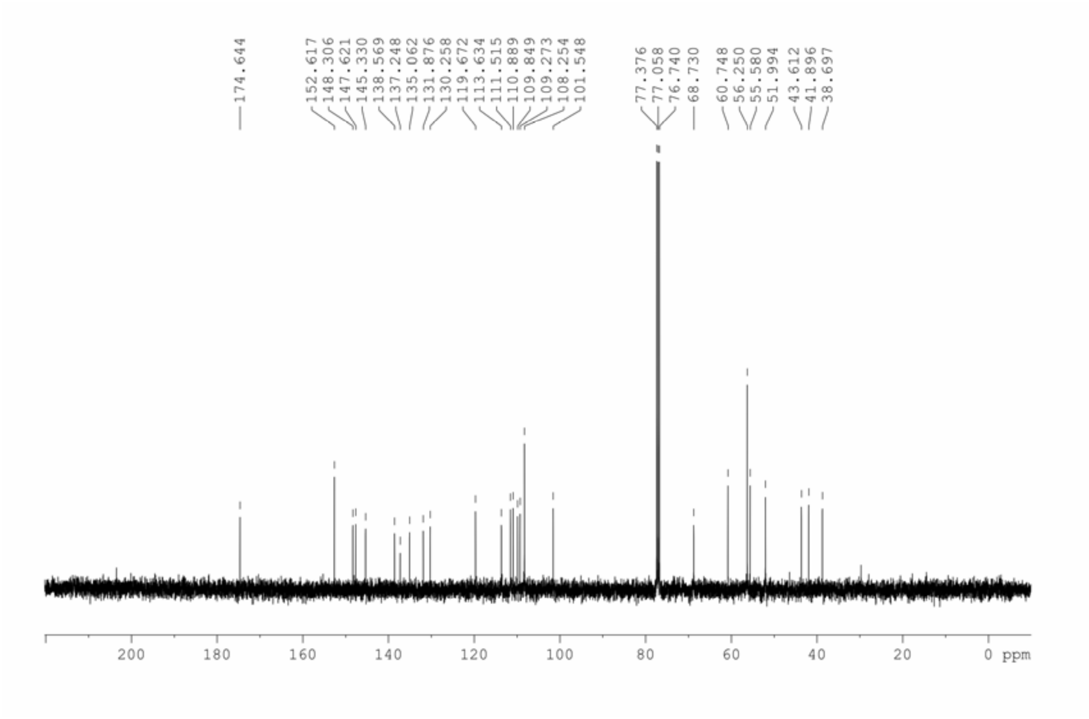

^{1}H NMR spectrum of Compound **4**.

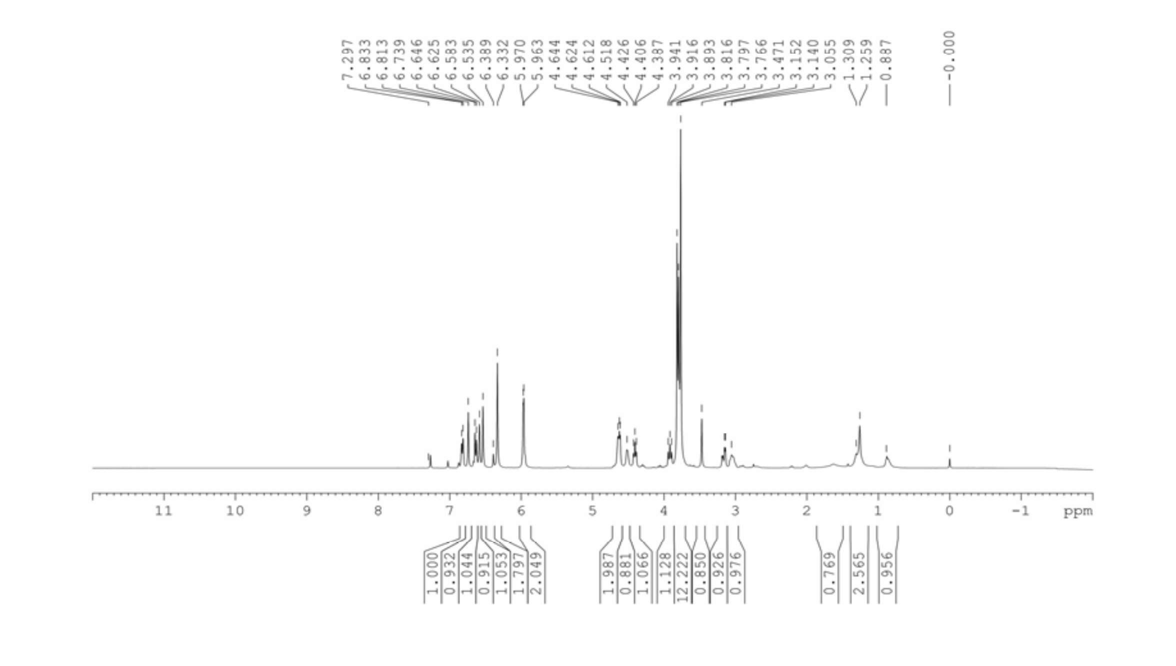

HMBC spectrums for Compound **4**.

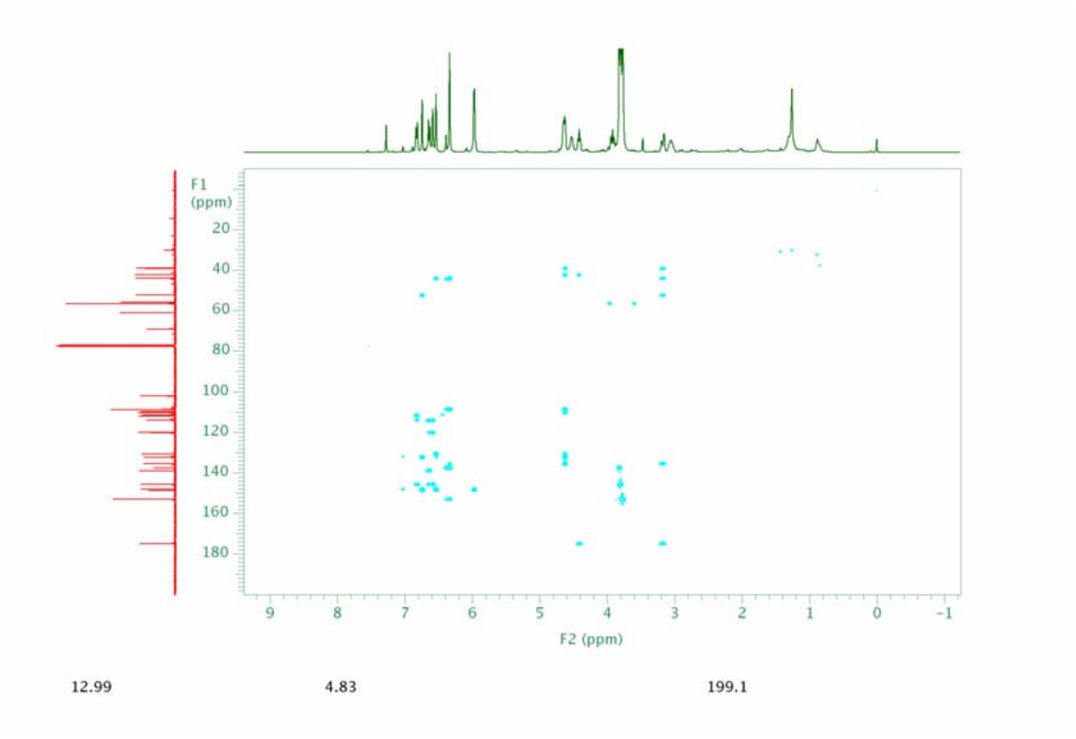

HSQC spectrums for Compound 4.

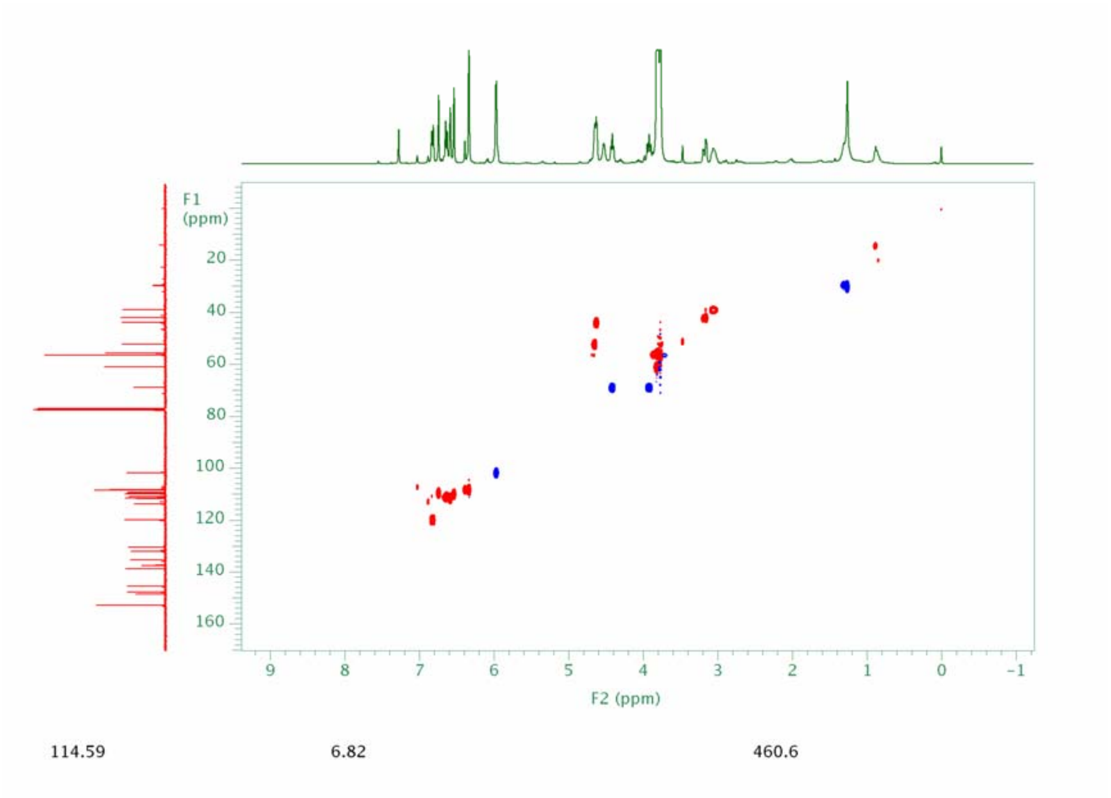

^{1}H-^{1}H COSY spectrums for Compound **4**.

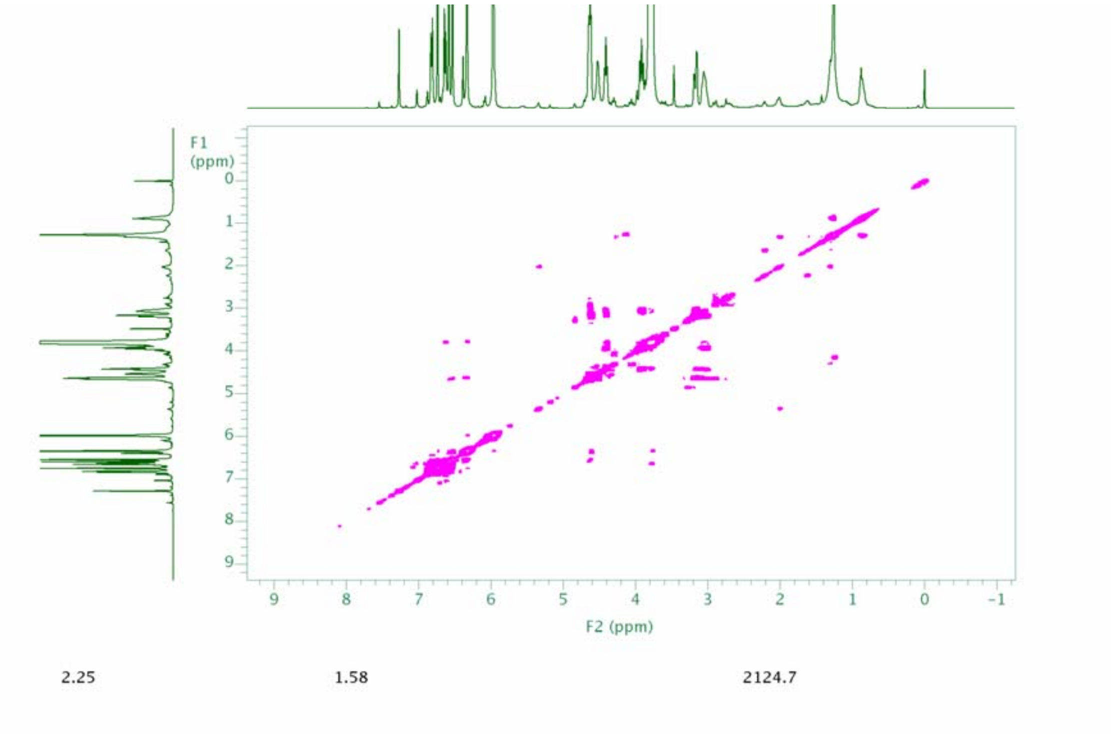

MS spectrums for Compound **4**.

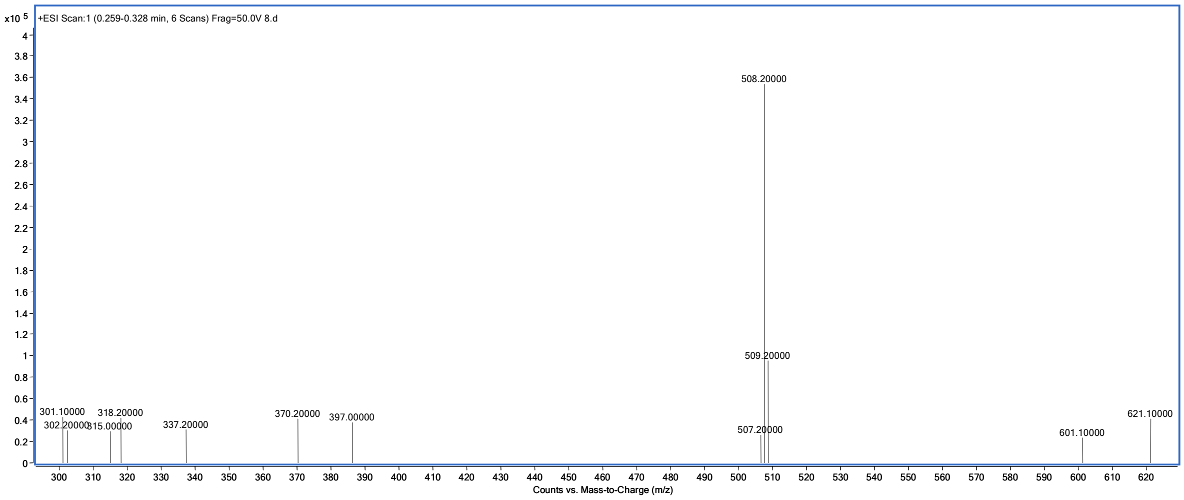

Compound 4 (4β-*NH*-(2-fluoroaniline)-4-deoxy-podophyllotoxin): 69% yield as white solid, ^{1}H NMR (400 MHz, $CDCl_3$): δ 7. 02 (m, 2H, ArH), 6.76 (s, 1H, ArH), 6.71 (t, *J*=8.0 Hz, ArH), 6.58 (t, *J*=8.0 Hz, 2H, ArH), 6.53 (s, 1H, ArH), 6.32 (s, 2H, ArH), 5.95 (s, 2H, OCH_2O), 4.67 (d, *J*=4.0 Hz, 1H, 1-H), 4.61 (d, *J*=4.0 Hz, 1H, 1-H), 4.39 (t, *J*=8.0Hz, 1H, 11-H), 3.98 (t, *J*=8.0 Hz, 1H, 11-H), 3.81(s, 3H, 4'-OCH_3), 3.75(s, 6H, 3', 5'-OCH_3), 3.17 (dd, *J*=8.0 Hz, 1H, 3-H), 3.02(m, 1H, 2-H);

^{13}C NMR (100 MHz, $CDCl_3$): δ174.61, 152.59, 148.37, 147.68, 137.21, 135.94, 135.83, 135.06, 131.82, 130.18, 124.77, 117.84, 114.94, 111.12, 109.93, 109.12, 108.26, 101.58, 68.73, 60.76, 56.27, 52.34, 43.57, 41.73, 38.71; MS (ESI): m/z: 508 $[M+H]^+$

^{13}C NMR spectrum of 4*β*-*NH*-(3-bromoaniline)-4-deoxy-podophyllotoxin **(5)**

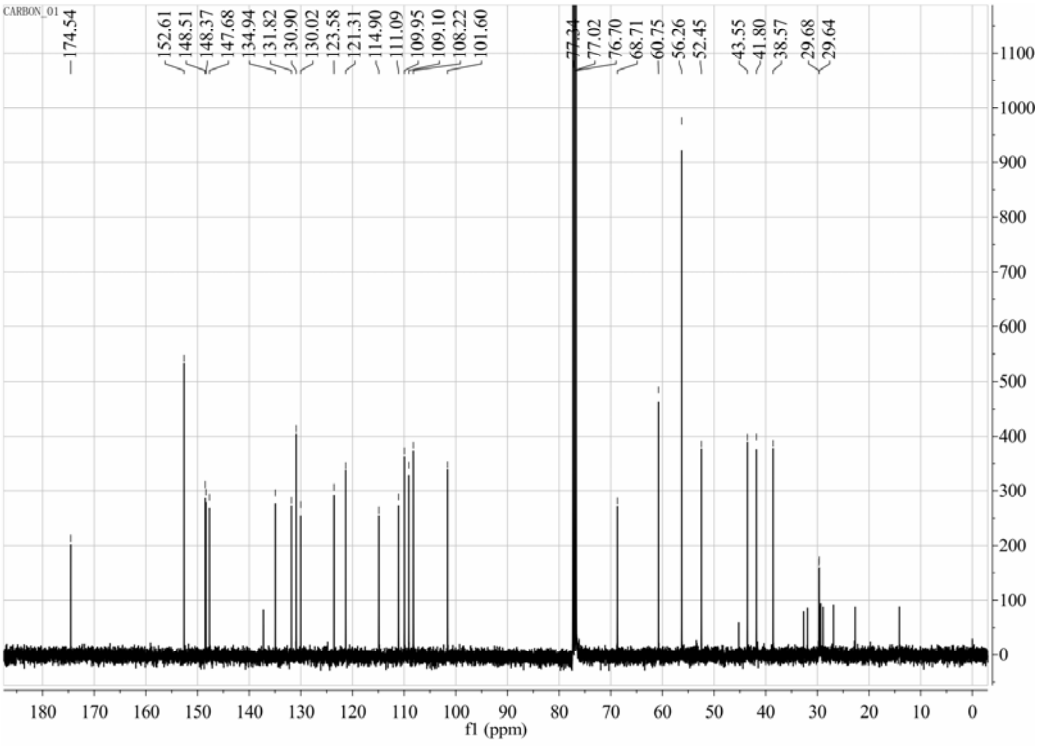

^1H NMR spectrum of Compound **5**.

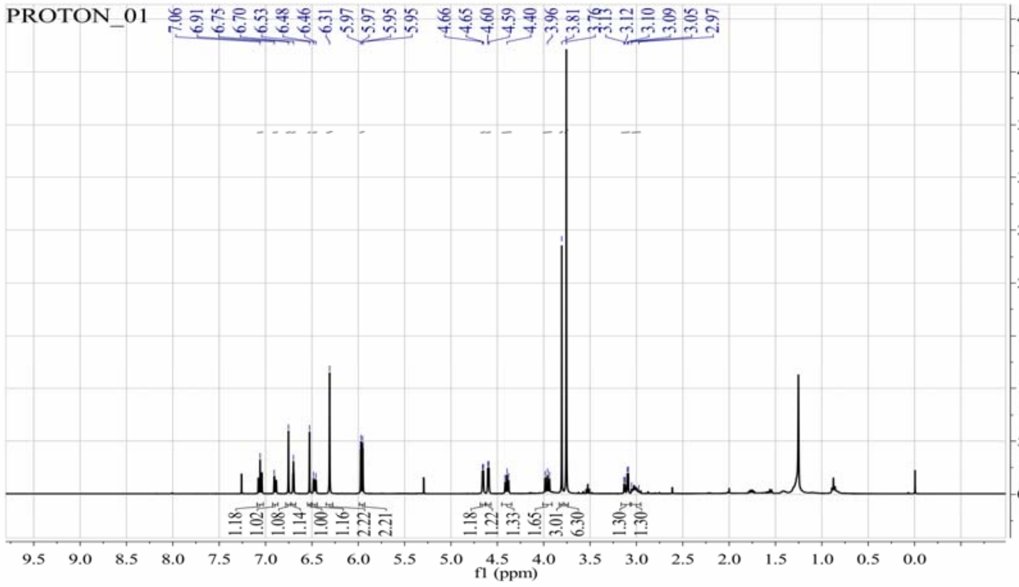

MS spectrums for Compound **5**.

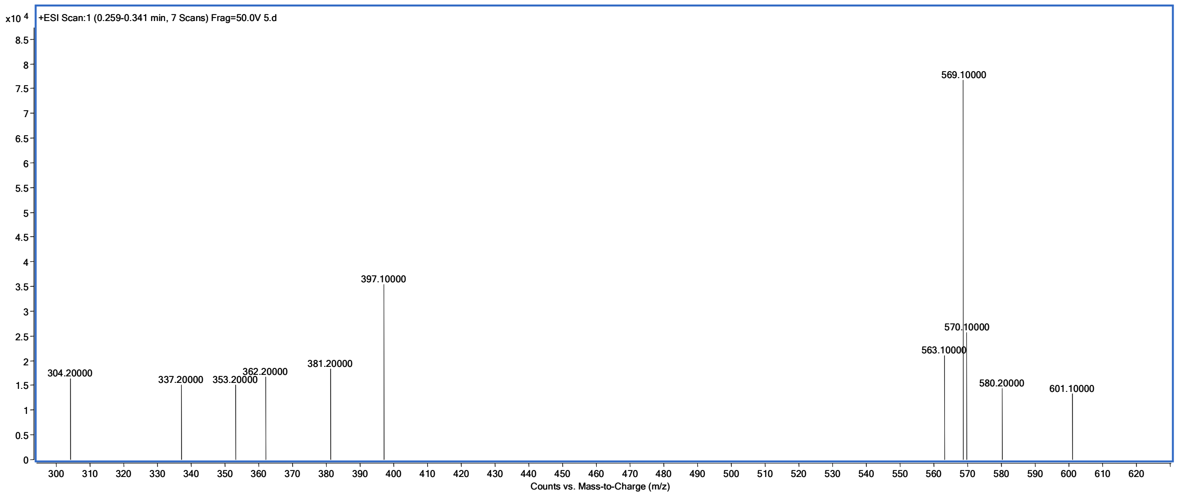

Compound 5 (4β-*NH*-(3-bromoaniline)-4-deoxy-podophyllotoxin): 73% yield as white solid, ^{1}H NMR (400 MHz, $CDCl_3$): δ7.06 (t, *J*=1.6 Hz, 1H, ArH), 6.75 (d, *J*=1.2 Hz, 1H, ArH), 6.70 (s, 1H, ArH), 6.62 (s, 1H, ArH), 6.53 (s, 1H, ArH), 6.46 (d, *J*=8.0 Hz, 1H, ArH), 6.31 (s, 2H, ArH), 5.95 (d, *J*=4.0 Hz, 2H, OCH_2O), 4.65 (d, *J*=4.0 Hz, 1H, 4H), 4.59 (d, *J*=4.0 Hz,1H, 1H), 4.40 (t, *J*=6.3Hz, 1H, 11-H), 3.96 (t, *J*=8.0 Hz, 1H, 11-H), 3.81 (s, 3H, 4′-OCH_3), 3.76 (s, 6H, 3’, 5’-OCH_3), 3.09 (dd, J=4.8 Hz, 1H, 3-H), 2.97 (m, 1H, 2-H); ^{13}C NMR (75 MHz, $CDCl_3$): δ 174.54, 152.61, 148.51, 148.37, 147.68, 134.94, 131.82, 130.90, 130.02, 123.58, 121.31, 114.90, 111.09, 109.95, 109.10, 108.22, 101.60, 68.71, 60.75, 56.26, 52.45, 43.55, 41.80, 38.57; MS (ESI): m/z: 569 $[M+H]^+$

^{13}C NMR spectrum of 4*β*-*NH*-(3-chloroaniline)-4-deoxy-podophyllotoxin **(6)**

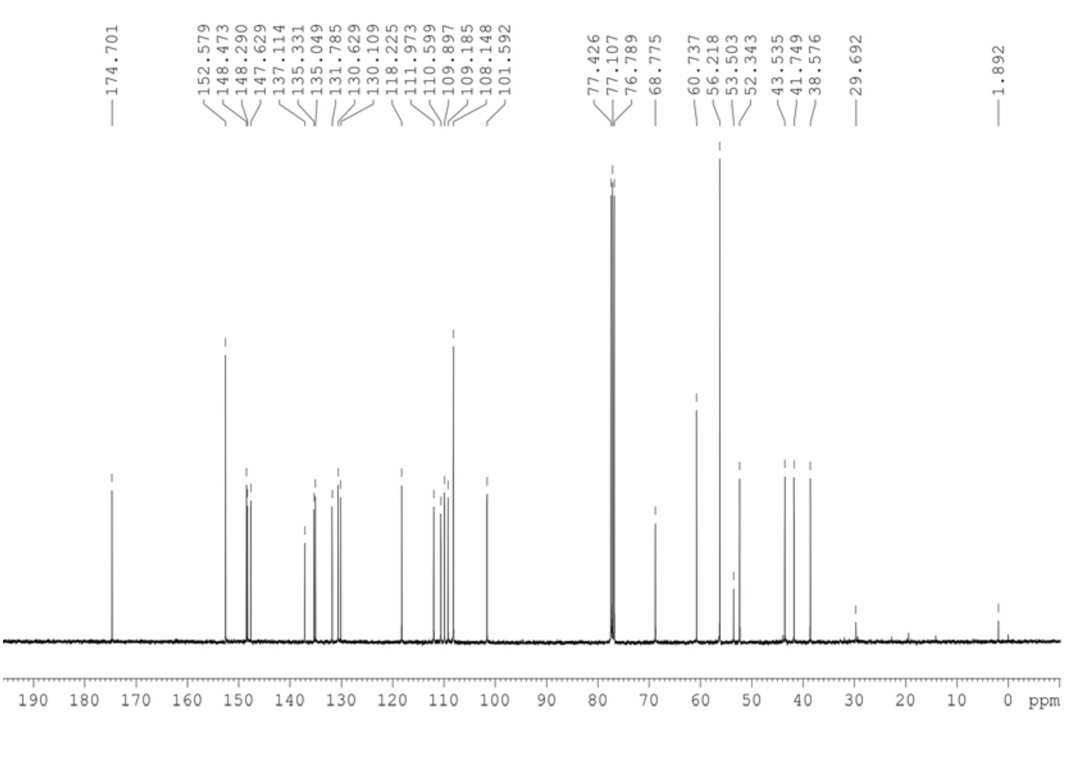

^{1}H NMR spectrum of Compound **6**.

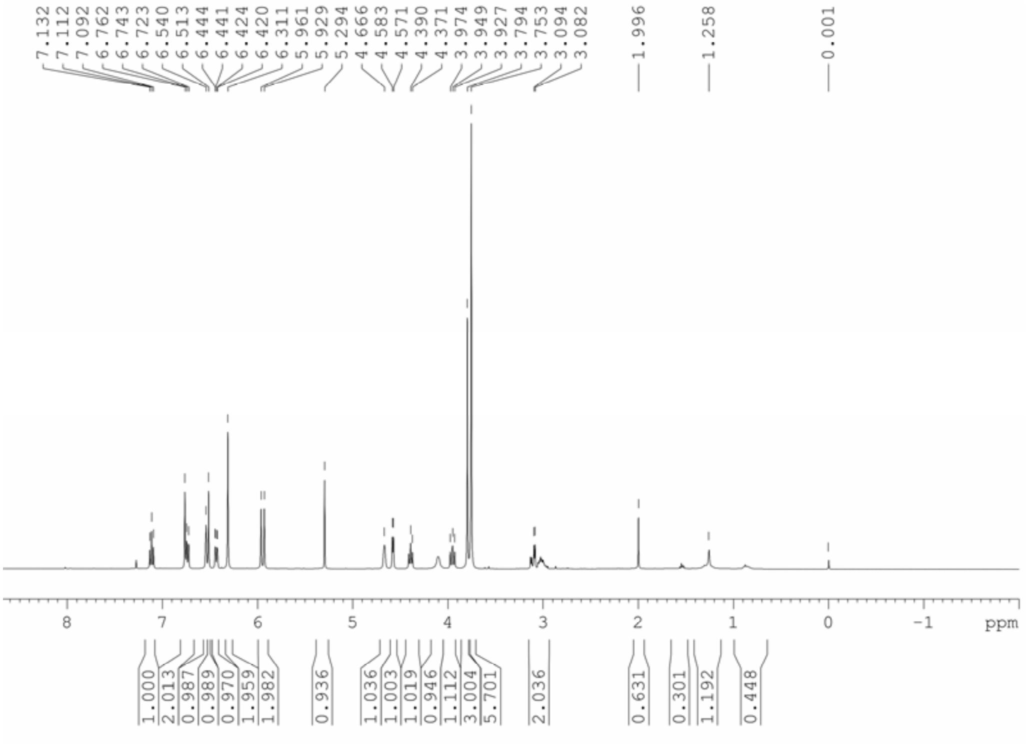

MS spectrums for Compound **6**.

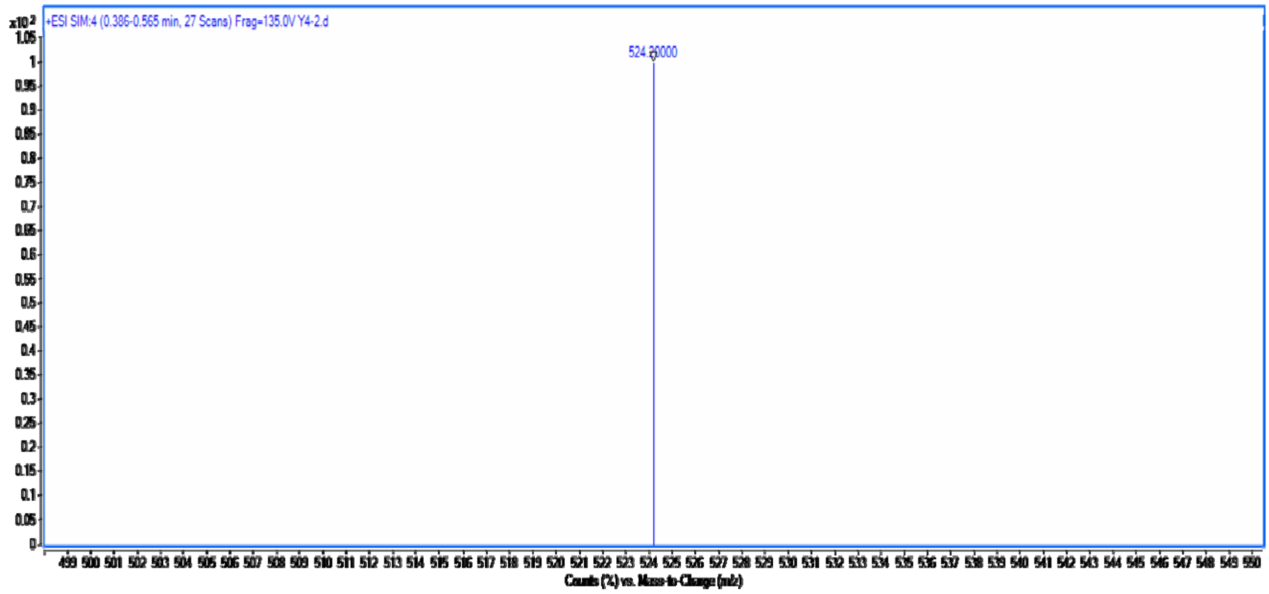

Compound 6 (4β-*NH*-(3-chloroaniline)-4-deoxy-podophyllotoxin): 77% yield as white solid, ^{1}H NMR (400 MHz, $CDCl_3$): δ 7.09 (m, 1H, ArH), 6.72 (m, 2H, ArH), 6.54 (s, 1H, ArH), 6.51 (s, 1H, ArH), 6.42 (d, *J*=1.6 Hz, 1H, ArH), 6.31 (s, 2H, ArH), 5.93 (d, *J*=9.2 Hz, 2H, OCH_2O), 4.67 (s, 1H, 1-H), 4.57 (t, *J*=4.8 Hz, 1H, 4-H), 4.39 (t, *J*=7.6 Hz, 1H, 11-H), 3.95 (t, *J*=8.8 Hz, 1H, 11-H), 3.79 (s, 3H, 4'-OCH_3), 3.75 (s, 6H, 3', 5'-OCH_3), 3.08 (dd, *J*=4.8 Hz, 1H, 3-H), 2.99 (m, 1H, 2-H); ^{13}C NMR (75 MHz, $CDCl_3$): δ 174.70, 152.58, 148.47, 148.29, 147.63, 137.63, 135.33, 135.05, 131.79, 130.63, 130.11, 118.26, 111.97, 110.60, 109.90, 109.19, 108.15, 101.59, 68.78, 60.74, 56.22, 52.34, 43.54, 41.75, 38.58; MS (ESI): m/z: 524 $[M+H]^+$

^{13}C NMR spectrum of 4*β*-*NH*-(3-fluoroaniline)-4-deoxy-podophyllotoxin **(7)**

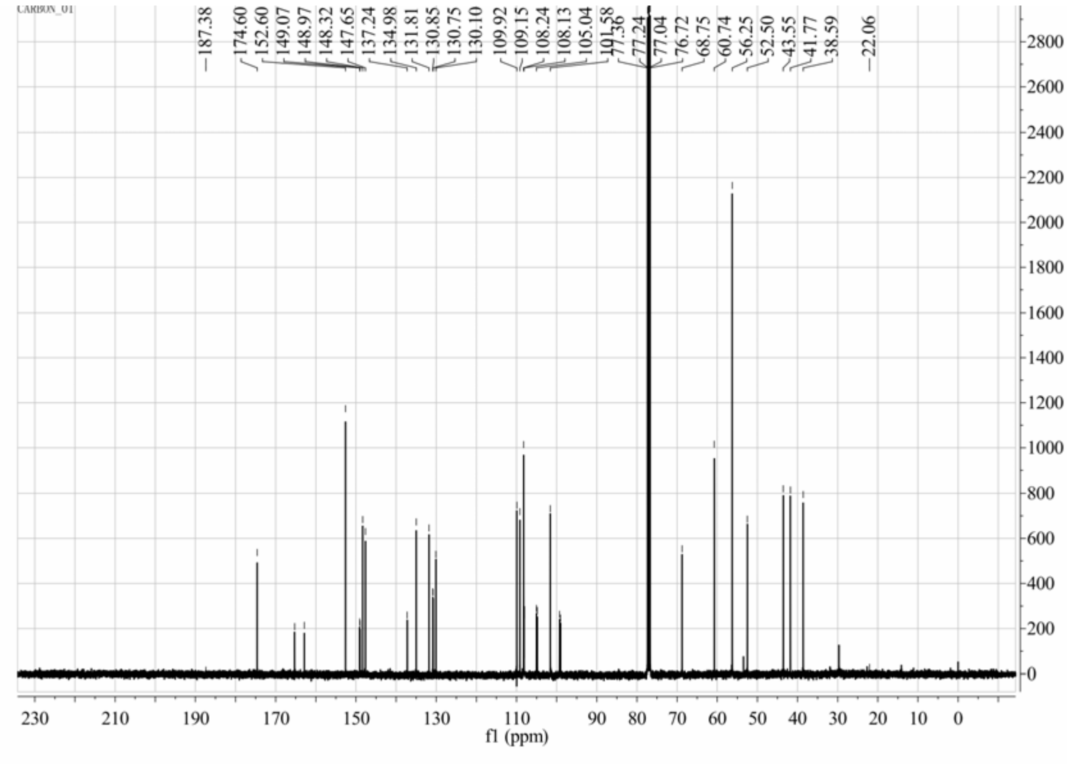

^{1}H NMR spectrum of Compound **7**.

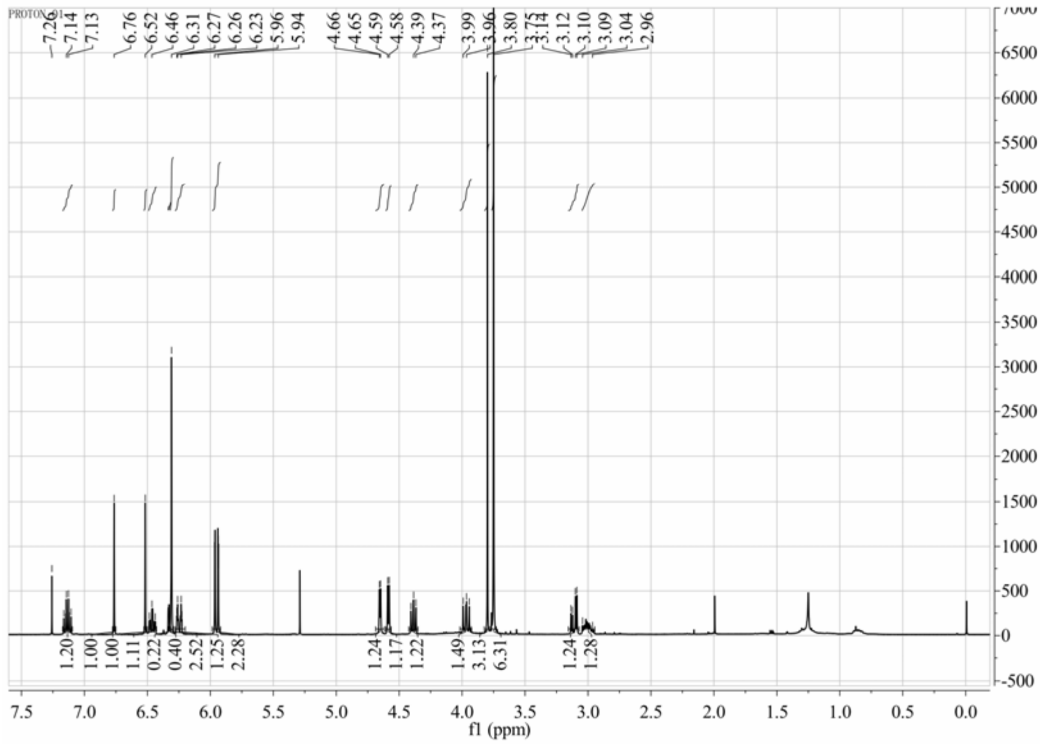

MS spectrums for Compound **7**.

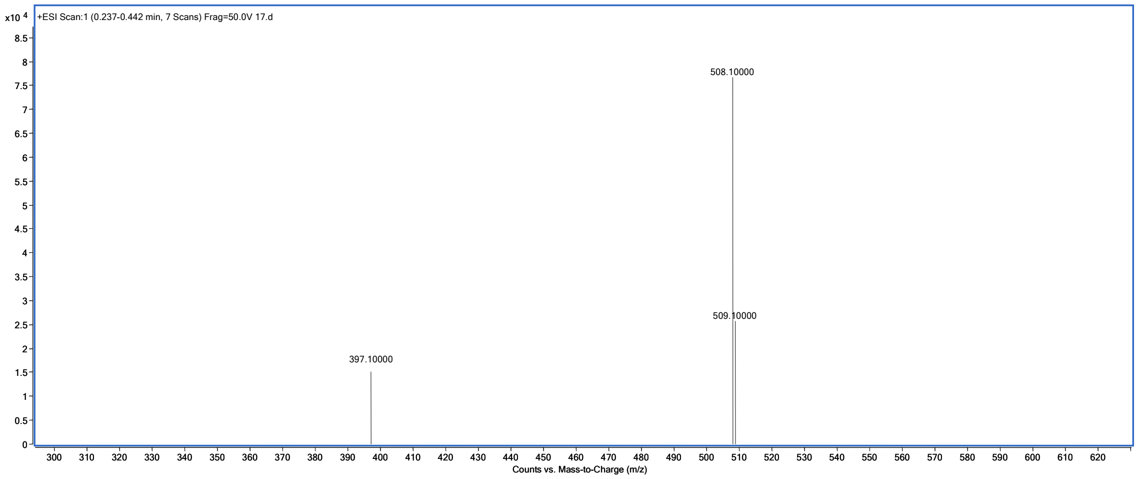

Compound 7 (4β-*NH*-(3-fluoroaniline)-4-deoxy-podophyllotoxin): 88% yield as white solid, ^{1}H NMR (400 MHz, $CDCl_3$): 7.13 (m, 1H, ArH), 6.76 (s, 1H, ArH), 6.52 (s, 1H, ArH), 6.46 (t, *J*=4.0 Hz, 1H, ArH), 6.31 (s, 2H, ArH), 6.26(d, *J*=4.0 Hz, 1H, ArH), 5.94 (d, *J*=8.0 Hz 2H, OCH_2O), 4.65 (d, *J*=4.0 Hz, 1H, 1-H), 4.58 (d, *J*=4.0 Hz, 1H, 4-H), 4.37 (t, *J*=8.0 Hz, 1H, 11-H), 3.96 (t, *J*=12.0 Hz, 1H, 11-H), 3.80(s, 3H, 4'-OCH_3), 3.75 (s, 6H, 3', 5'-OCH_3), 3.09 (dd, *J*=4.0 Hz, 1H, 3-H), 2.96 (m, 1H, 2-H); ^{13}C NMR (100 MHz, $CDCl_3$): δ 174.60, 152.60, 149.07, 148.97, 148.32, 147.65, 137.24, 134.98, 131.81, 130.85, 130.75, 130.10, 109.92, 109.15, 108.24, 108.13, 105.04, 101.58, 68.75, 60.74, 56.25, 52.50, 43.55, 41.77, 38.59; MS (ESI): m/z: 508 $[M+H]^+$

^{13}C NMR spectrum of 4*β*-*NH*-(4-bromoaniline)-4-deoxy-podophyllotoxin **(8)**

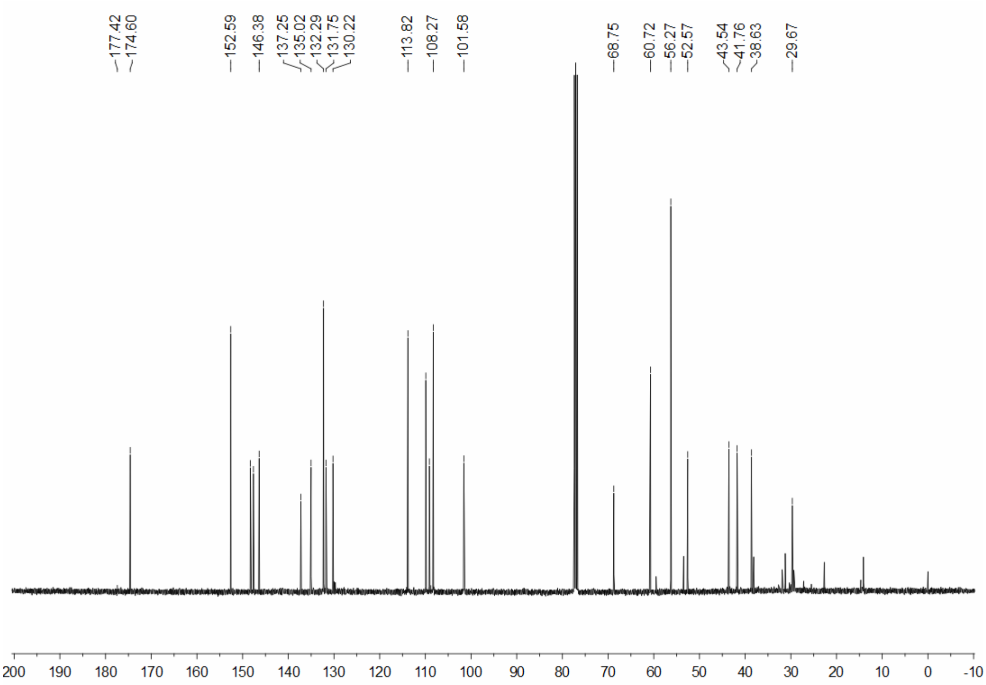

^{1}H NMR spectrum of Compound **8**.

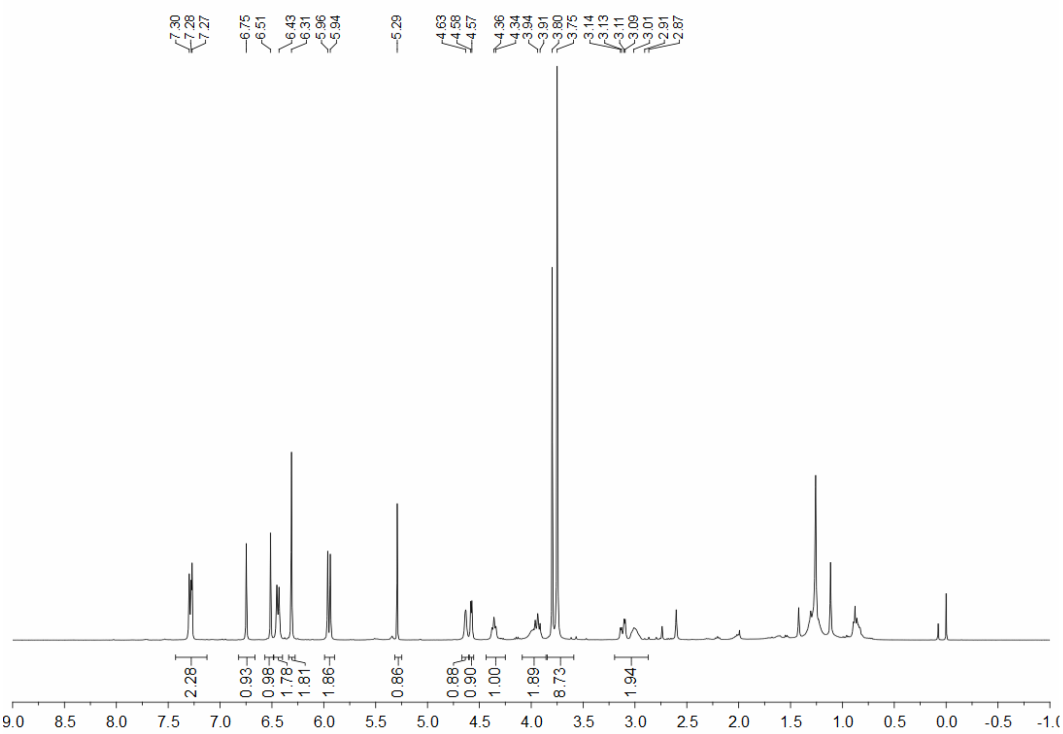

HMBC spectrums for Compound **8**.

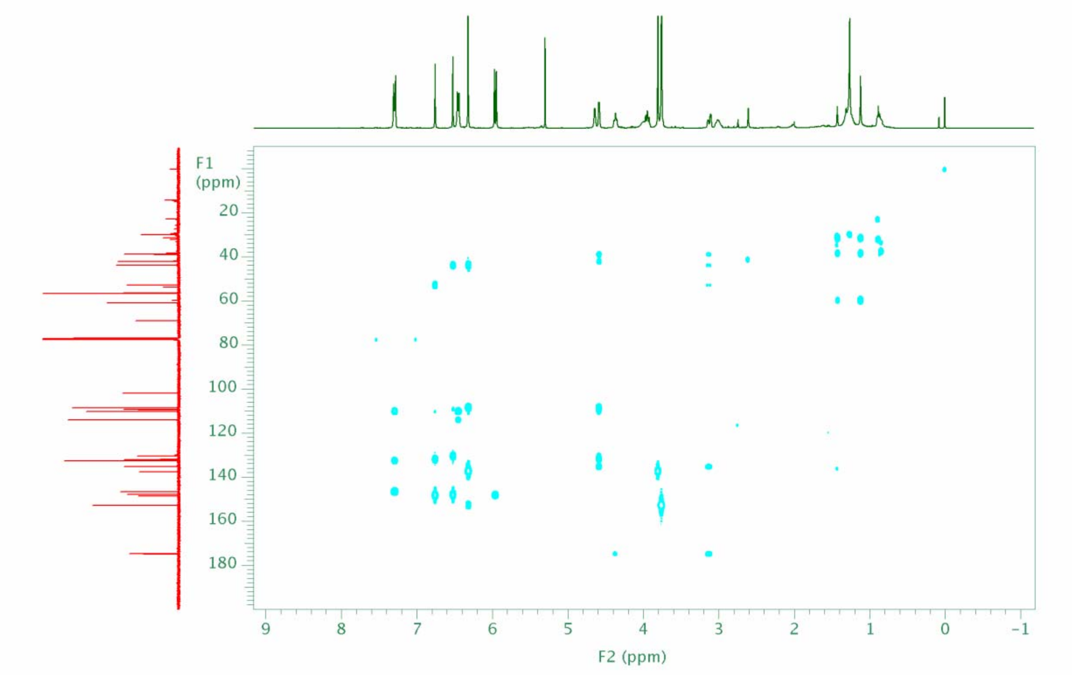

HSQC spectrums for Compound **8**.

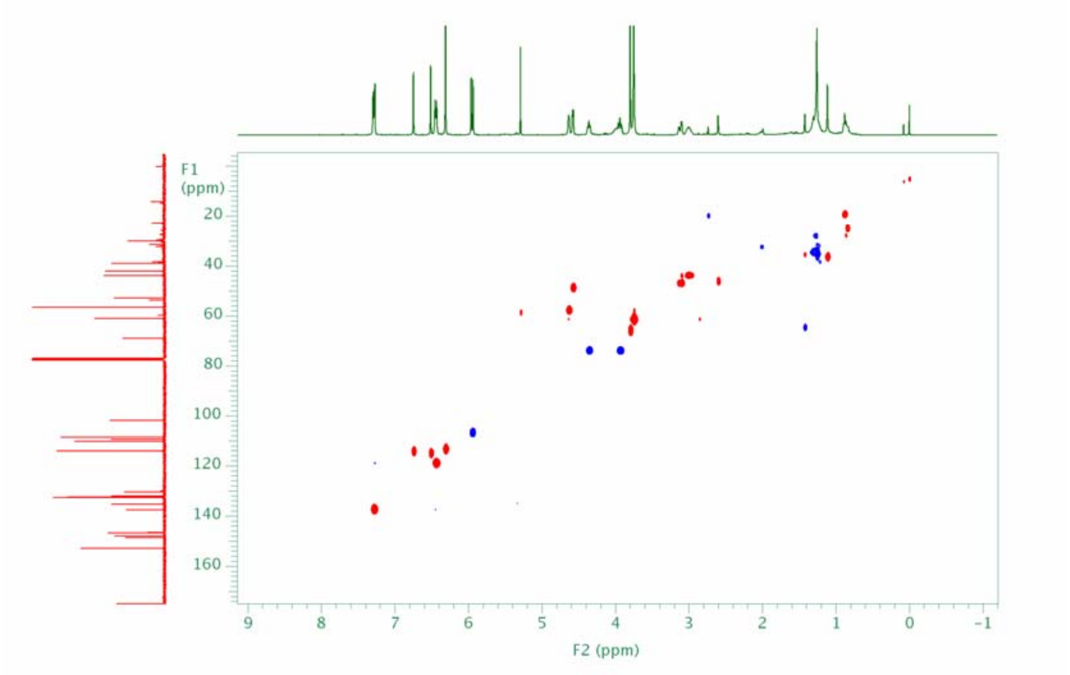

^{1}H-^{1}H COSY spectrums for Compound **8**.

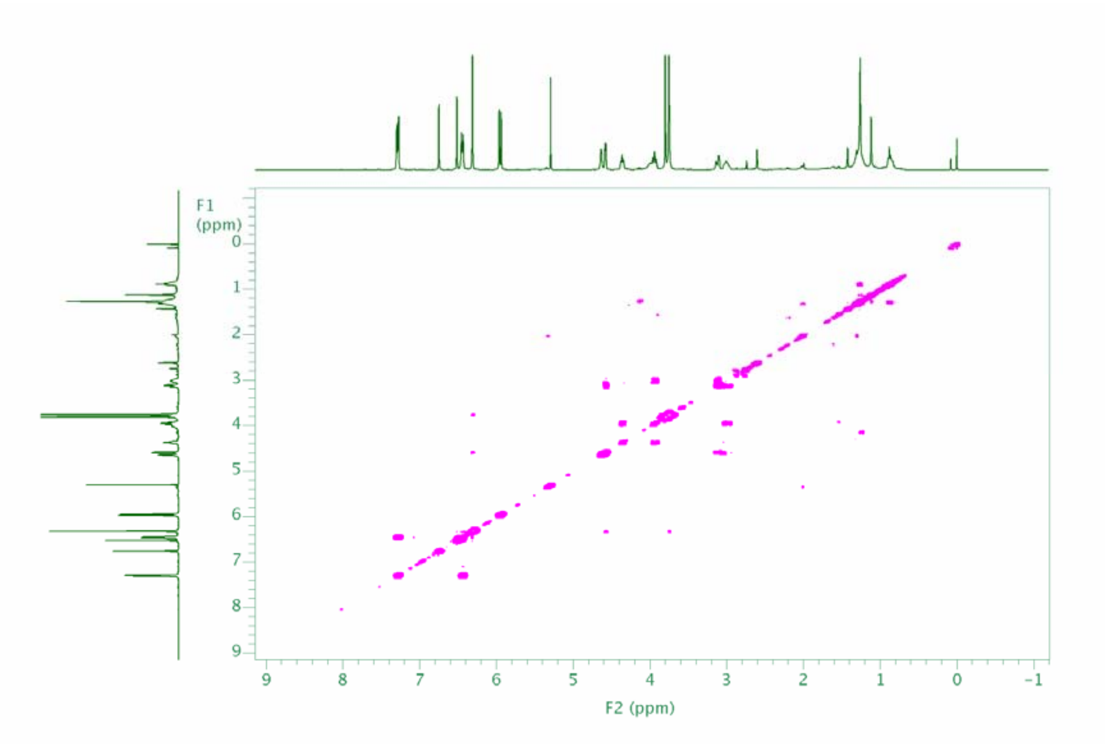

MS spectrums for Compound **8**.

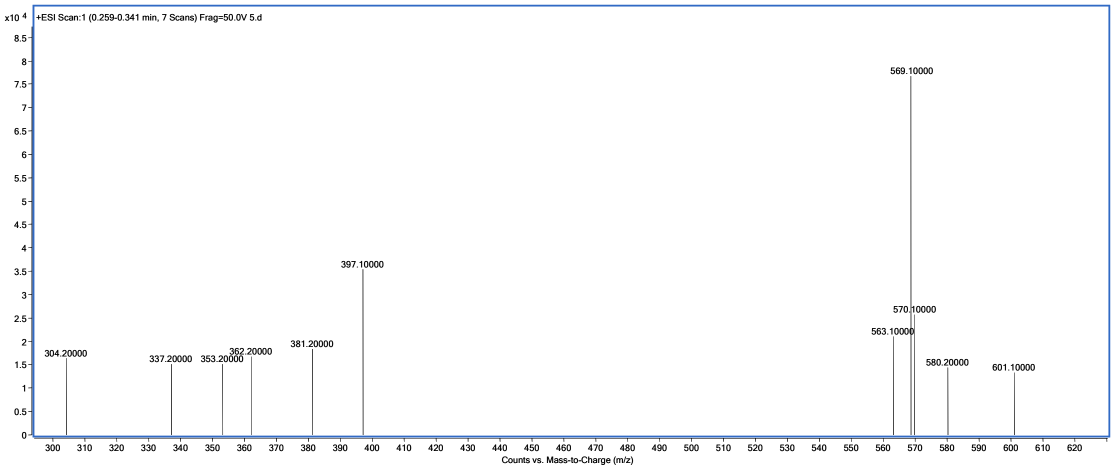

Compound 8 (4β-*NH*-(4-bromoaniline)-4-deoxy-podophyllotoxin): 71% yield as white solid, ^{1}H NMR (400 MHz, $CDCl_3$): δ 7.28 (d, *J*=8.0 Hz, 2H, ArH), 6.75 (s, 1H, ArH), 6.51 (s, 1H, ArH), 6.43 (d, *J*=8.0 Hz, 2H, ArH), 6.31 (s, 2H, ArH), 5.94 (d, *J*=8.0 Hz, 2H, OCH_2O), 4.63 (s, 1H, 4-H), 4.57 (d, *J*=4.0 Hz, 1H, 1-H), 4.36 (t, *J*=8.0Hz, 1H, 11-H), 3.94 (t, *J*=8.0 Hz, 1H, 11-H), 3.80(s, 3H, 4'-OCH_3), 3.75 (s, 6H, 3', 5'-OCH_3), 3.09 (dd, *J*=8.0 Hz, 1H, 3-H), 3.01(m, 1H, 2-H); ^{13}C NMR (100 MHz, $CDCl_3$): δ 174.60, 152.59, 148.29, 147.63, 146.38, 137.25, 135.05, 132.29, 131.75,

130.22, 113.82, 109.90, 109.12, 108.28, 101.58, 68.75, 60.72, 56.27, 52.58, 43.55, 41.76, 38.63;

MS (ESI): m/z: 569 $[M+H]^+$

^{13}C NMR spectrum of 4*β*-*NH*-(4-chloroaniline)-4-deoxy-podophyllotoxin **(9)**.

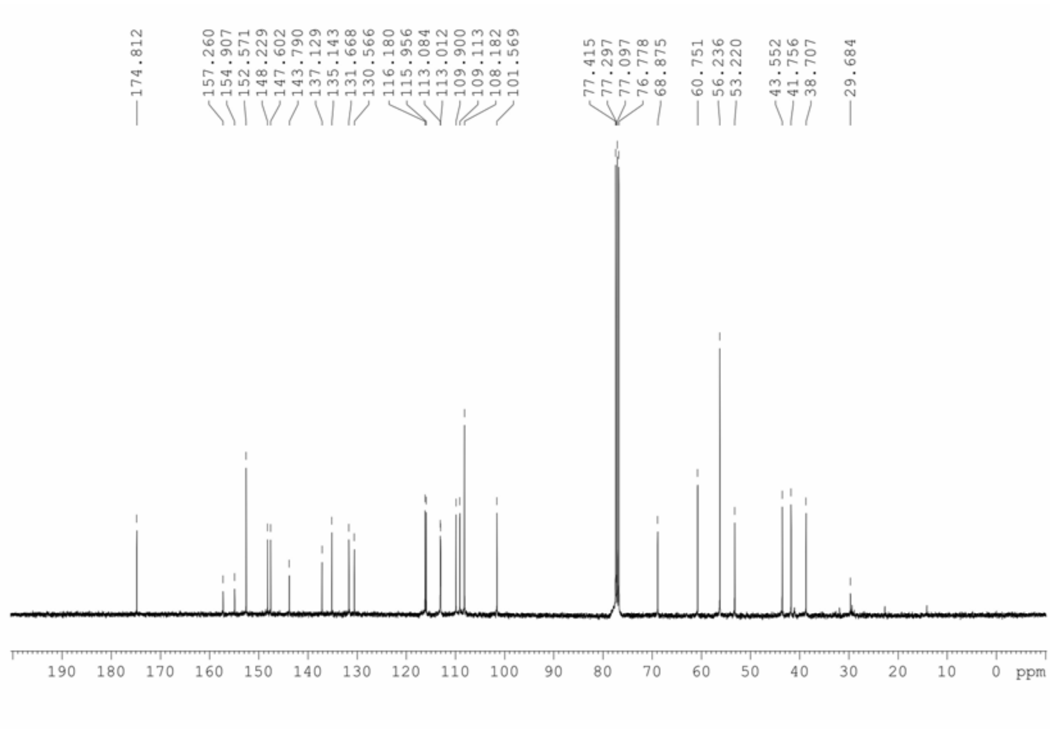

^{1}H NMR spectrum of compound **9**.

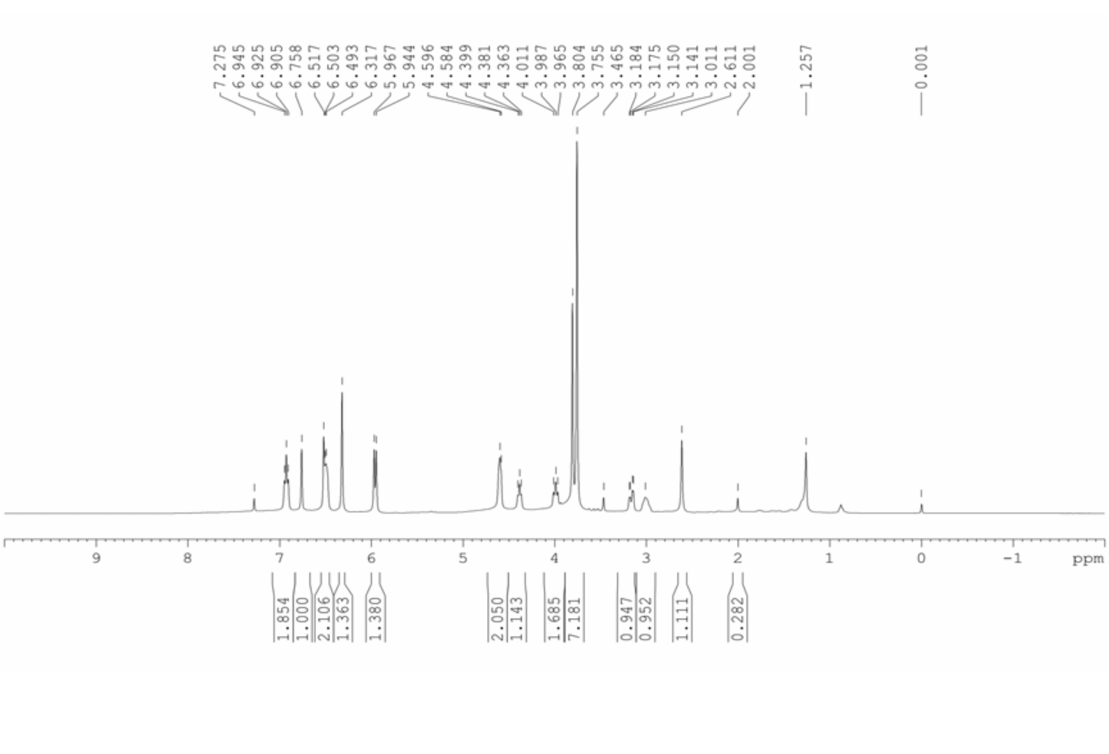

MS spectrums for Compound **9**.

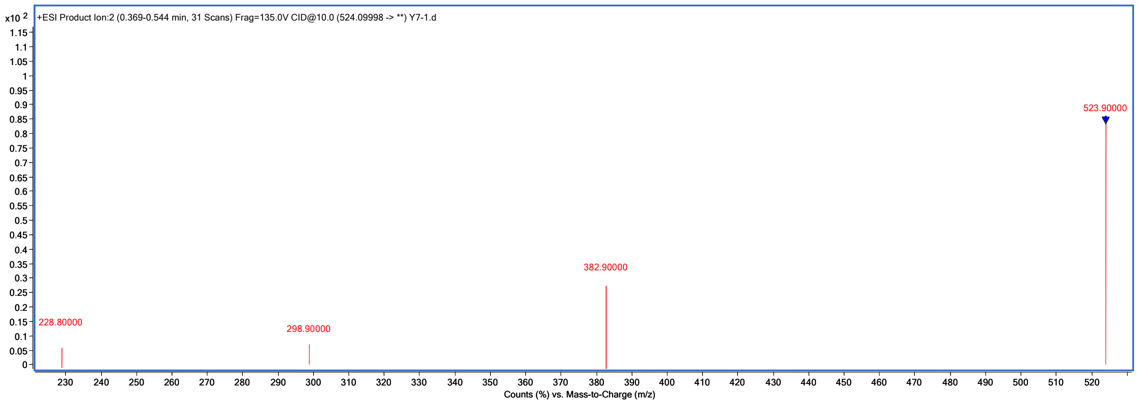

Compound 9 (4β-*NH*-(4-chloroaniline)-4-deoxy-podophyllotoxin): 77% yield as white solid, ^{1}H NMR (400 MHz, $CDCl_3$): δ 7.15 (d, *J*=8.0 Hz, 2H, ArH), 6.75 (s, 1H, ArH), 6.51 (s, 1H, ArH), 6.49 (d, *J*=8.0 Hz, 2H, ArH), 6.31 (s, 2H, ArH), 5.94 (d, *J*=8.0 Hz, 2H, OCH_2O), 4.58 (d, *J*=4.0 Hz, 2H, 1-H, 4-H), 4.38 (t, *J*=8.0Hz, 1H, 11-H), 3.98 (t, *J*=8.0 Hz, 1H, 11-H), 3.80 (s, 3H, 4'-OCH_3), 3.75 (s, 6H, 3', 5'-OCH_3), 3.14 (dd, *J*=4.0 Hz, 1H, 3-H), 3.01(m, 1H, 2-H); ^{13}C NMR (100 MHz, $CDCl_3$): δ 174.81, 152.57, 148.23, 147.60, 146.38, 137.13, 135.14, 132.33, 131.68, 130.56, 113.82, 109.90, 109.12, 108.28, 101.58, 68.75, 60.72, 56.23, 53.22, 43.55, 41.76, 38.71; MS (ESI): m/z: 524 $[M+H]^+$

^{13}C NMR spectrum of 4*β*-*NH*-(3-fluoroaniline)-4-deoxy-podophyllotoxin **(10)**.

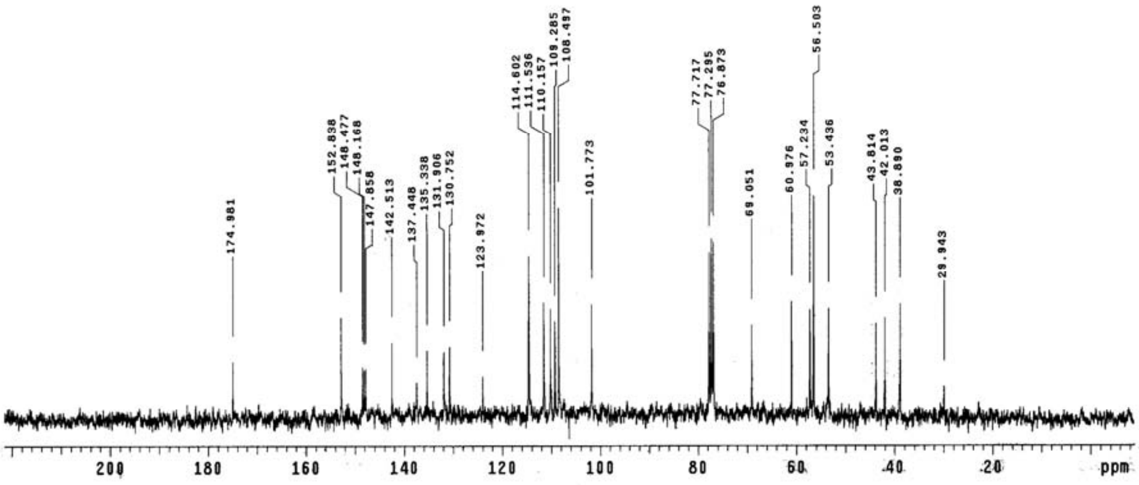

^{1}H NMR spectrum of Compound **10**.

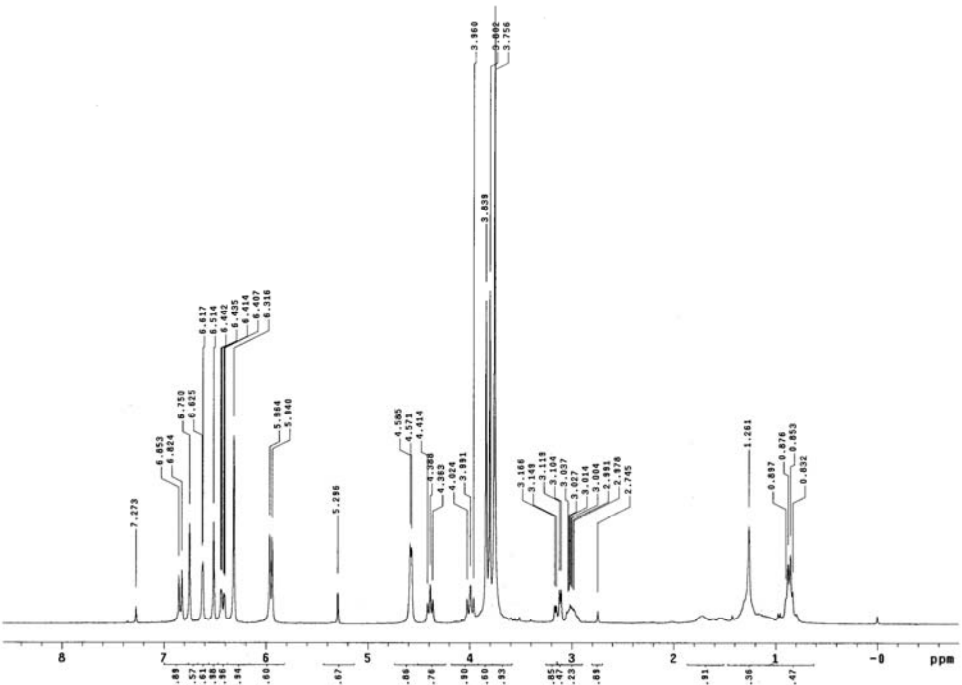

HMBC spectrums for Compound **10**.

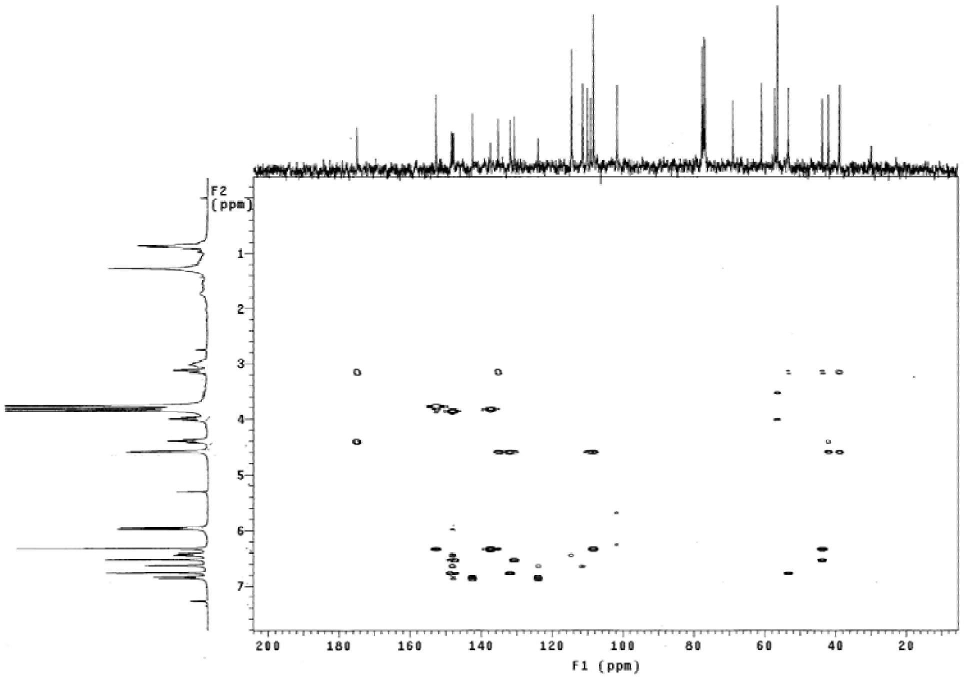

HSQC spectrums for Compound **10**.

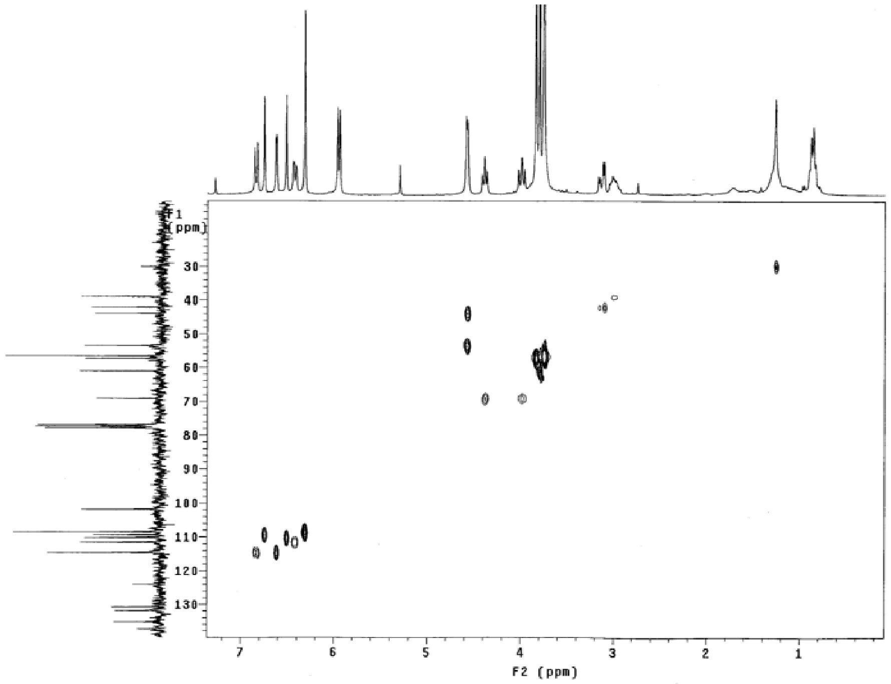

^{1}H-^{1}H COSY spectrums for Compound **10**.

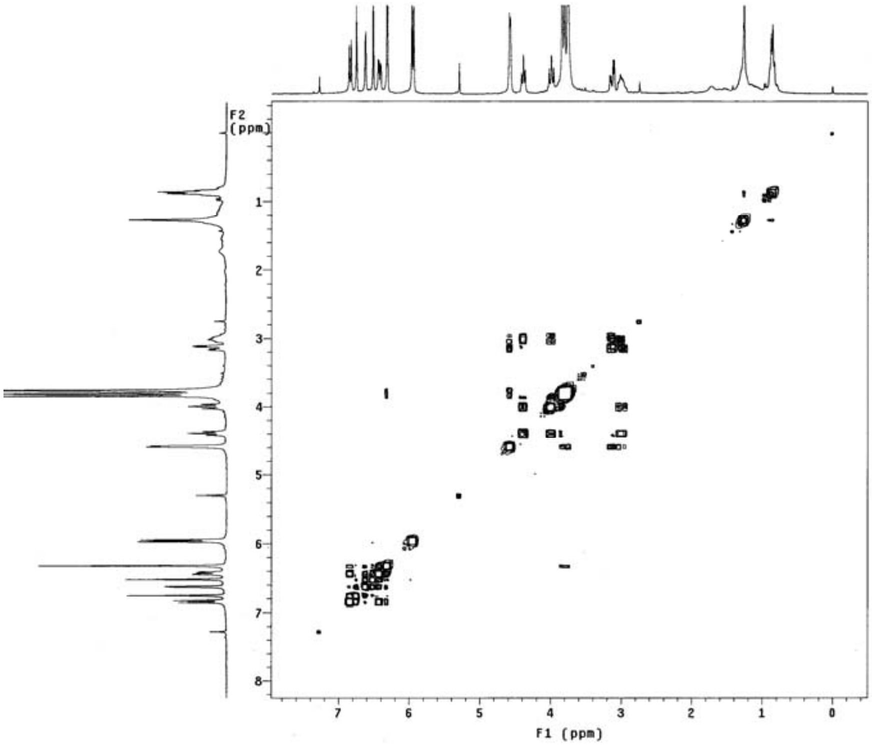

MS spectrums for Compound **10**.

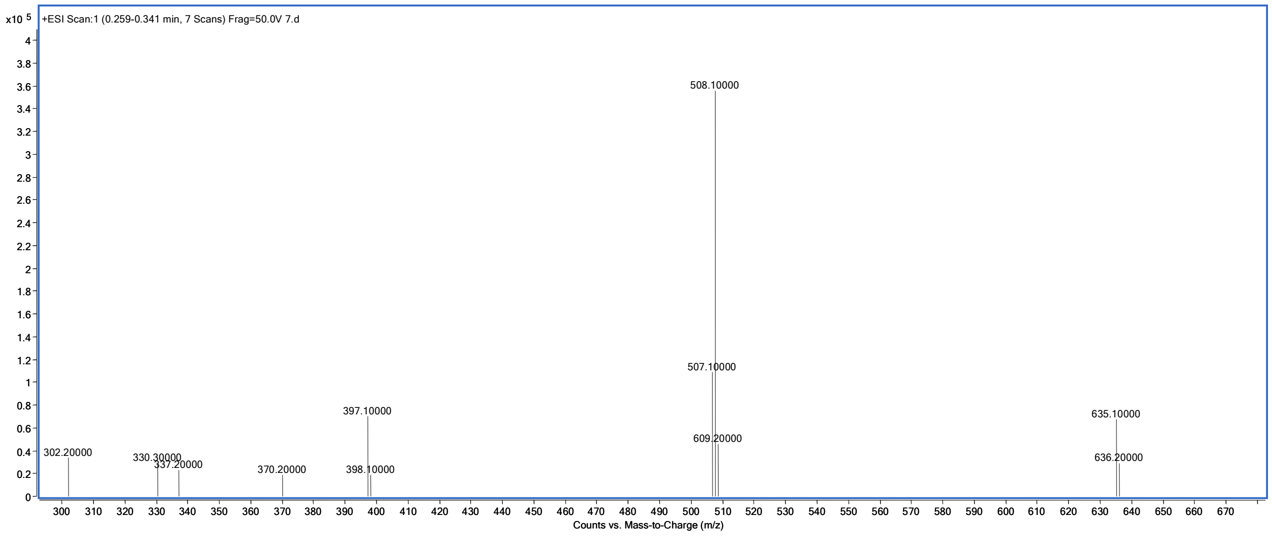

Compound 10 (4β-*NH*-(4-fluoroaniline)-4-deoxy-podophyllotoxin): 81% yield as white solid, ^{1}H NMR (400 MHz, $CDCl_3$): δ 7.15(d, *J*=8.0 Hz, 2H, ArH), 6.74 (s, 1H, ArH), 6.52 (s, 1H, ArH), 6.46 (d, *J*=8.0 Hz, 2H, ArH), 6.30 (s, 2H, ArH), 5.95 (d, *J*=8.0 Hz, 2H, OCH_2O), 4.59 (m, 2H, 1-H, 4-H), 4.38 (t, *J*=8.0Hz, 1H, 11-H), 3.95 (t, *J*=8.0 Hz, 1H, 11-H), 3.80 (s, 3H, 4'-OCH_3), 3.75 (s, 6H, 3',

5’-OCH3), 3.11 (dd, J=4.0 Hz, 1H, 3-H), 3.01 (m, 1H, 2-H); ^{13}C NMR (100 MHz, CDCl3): δ 174.60, 152.60, 148.34, 147.66, 145.97, 137.21, 134.97, 131.76, 130.20, 129.45, 123.08, 113.31, 109.95, 109.06, 108.20, 101.59, 68.74, 60.77, 56.26, 52.76, 43.54, 41.79, 38.63; MS (ESI): m/z: 508 [M+H]$^+$

^{13}C NMR spectrum of 4β-*NH*-(1-anisidine)-4-deoxy-podophyllotoxin **(11)**

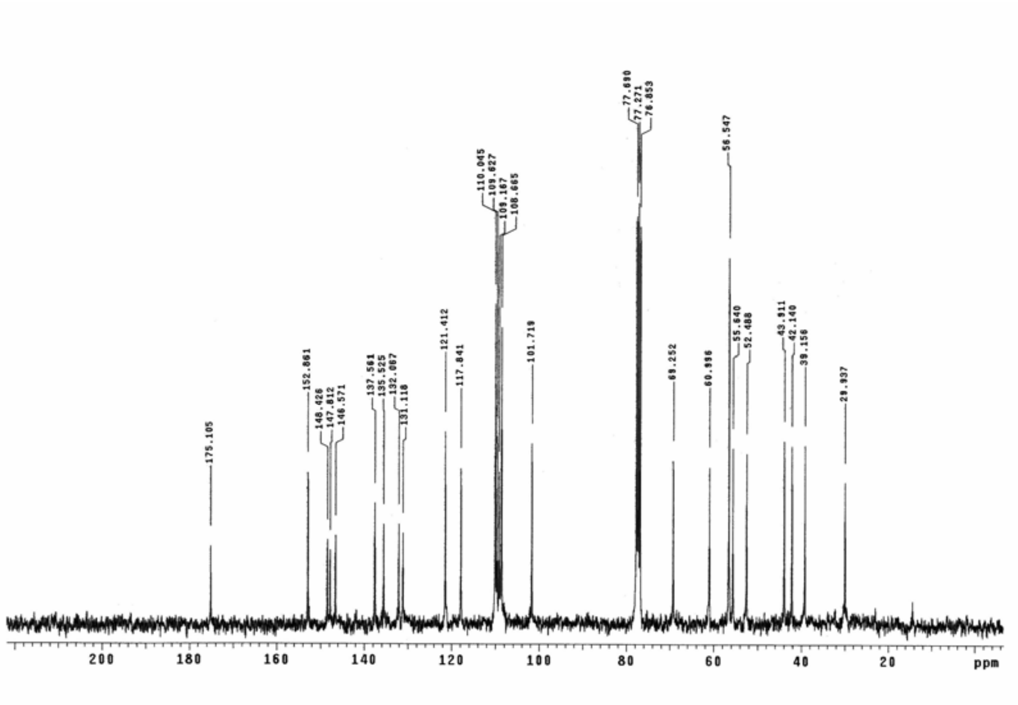

^{1}H NMR spectrum of Compound 11

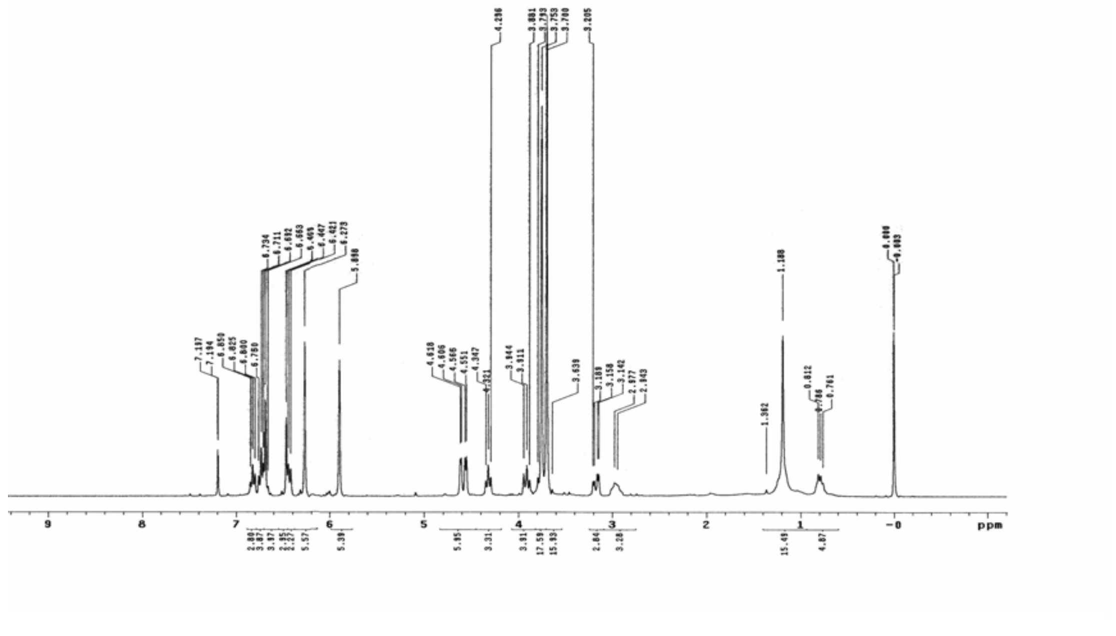

HMBC spectrums for Compound **11**.

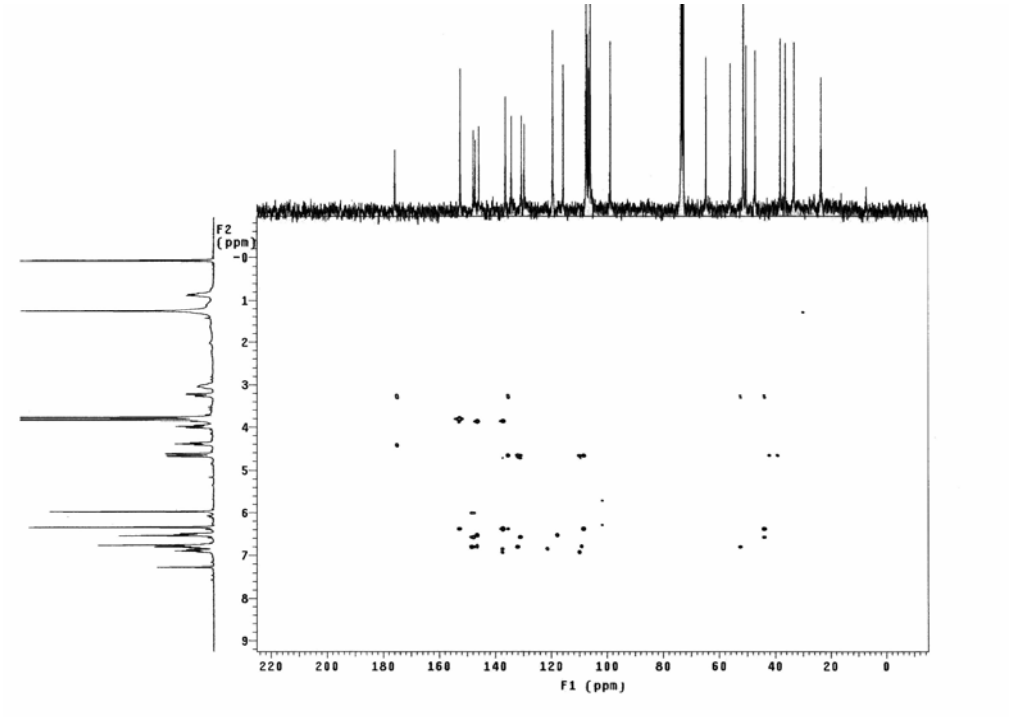

HSQC spectrums for Compound **11**.

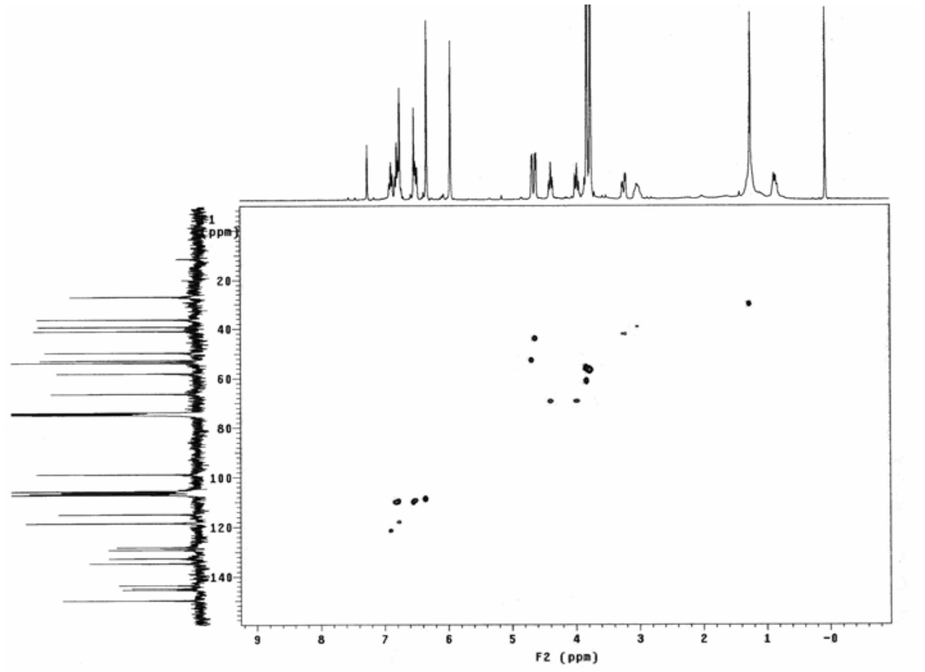

^{1}H-^{1}H COSY spectrums for Compound **11**.

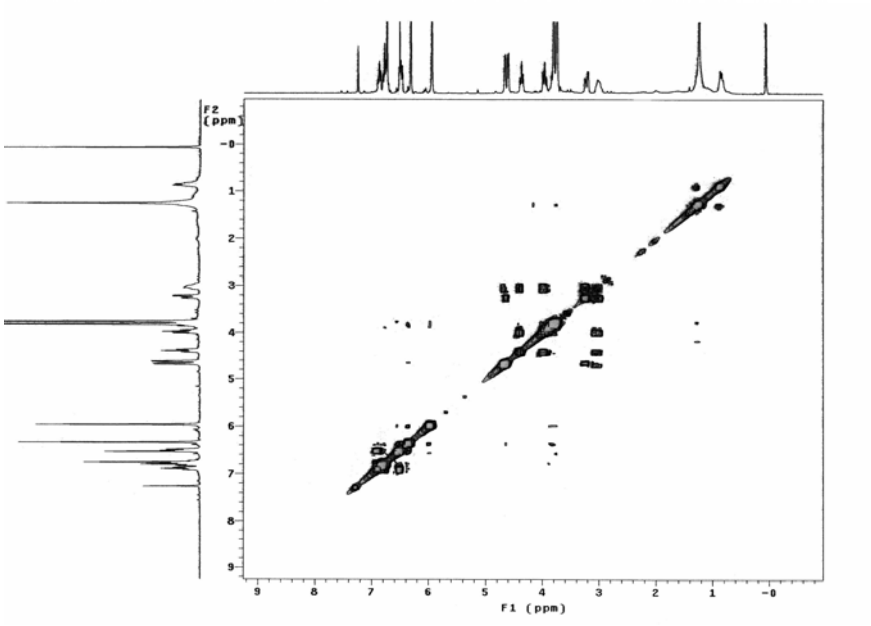

MS spectrums for Compound **11**.

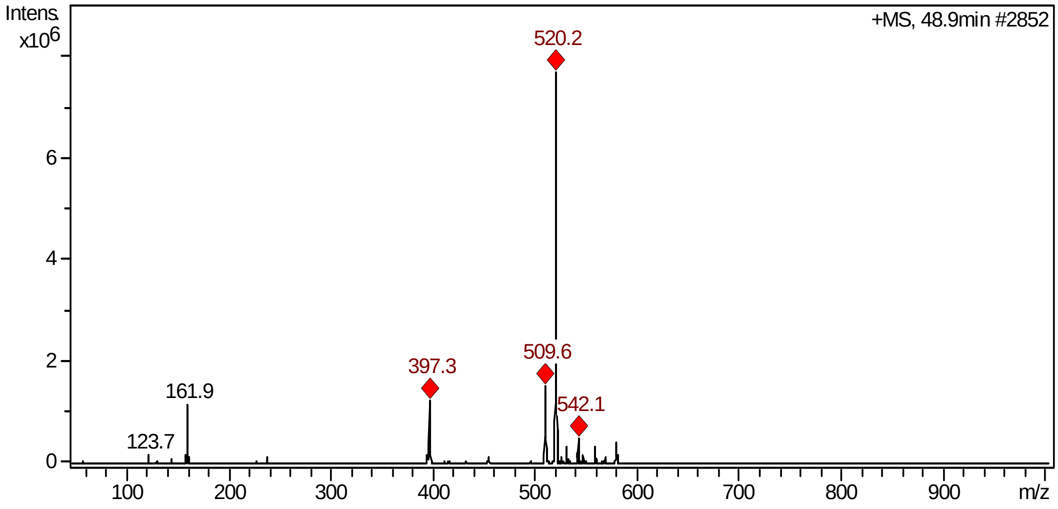

Compound 11 (4β-*NH*-(1-anisidine)-4-deoxy-podophyllotoxin): 57% yield as white solid, ^{1}H NMR (300 MHz, CDCl3): δ 2.943-2.977 (m, 1H, 2-H), 3.142 (dd, *J*=4.8 Hz, 1H, 3-H), 3.700 (s, 6H, 3′, 5′-OCH3), 3.700 (s, 6H, Ar-OCH3, 4′-OCH3), 3.911 (t, J=9.9 Hz, 1H, 11-H), 4.321 (t, *J*=7.8 Hz, 1H, 11-H), 4.551 (d, *J*=4.5 Hz, 1H,4-H), 4.606 (d, *J*=3.6 Hz, 1H, 1-H), 5.898 (s, 2H, OCH2O), 6.273 (s, 2H, ArH) , 6.421-6.469 (m, 2H, ArH), 6.663-6.760 (m, 3H, ArH), 6.825 (t, *J*=7.5 Hz, 1H, ArH) ^{13}C NMR (75 MHz, CDCl3): δ 39.156 , 42.140, 43.911, 52.488, 55.640, 56.547, 60.996,

69.252, 101.719, 108.665, 109.167, 109.627, 110.045, 117.841, 121.412, 131.118, 132.067, 135.525, 137.561, 146.571, 147.812, 148.426, 152.861, 175.105; MS (ESI): m/z: 520 [M+H]$^+$

^{13}C NMR spectrum of 4*β*-*NH*-(2-anisidine)-4-deoxy-podophyllotoxin **(12)**.

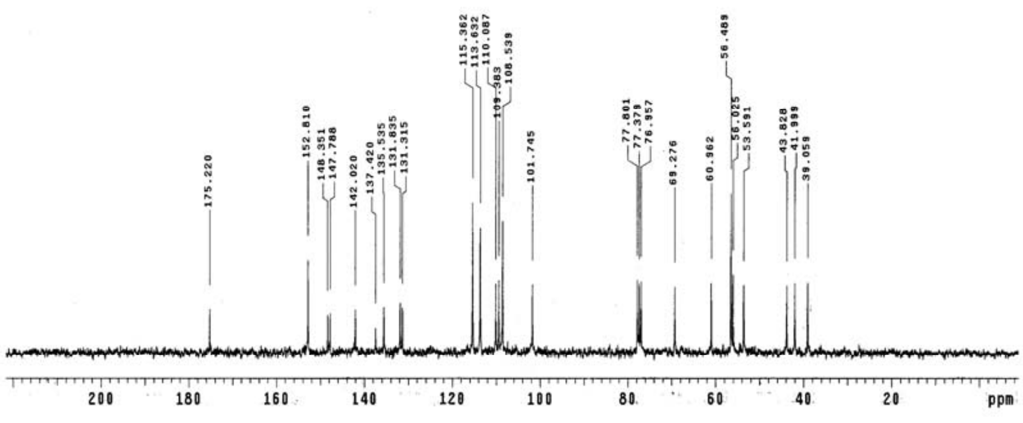

^{1}H NMR spectrum of Compound **12**.

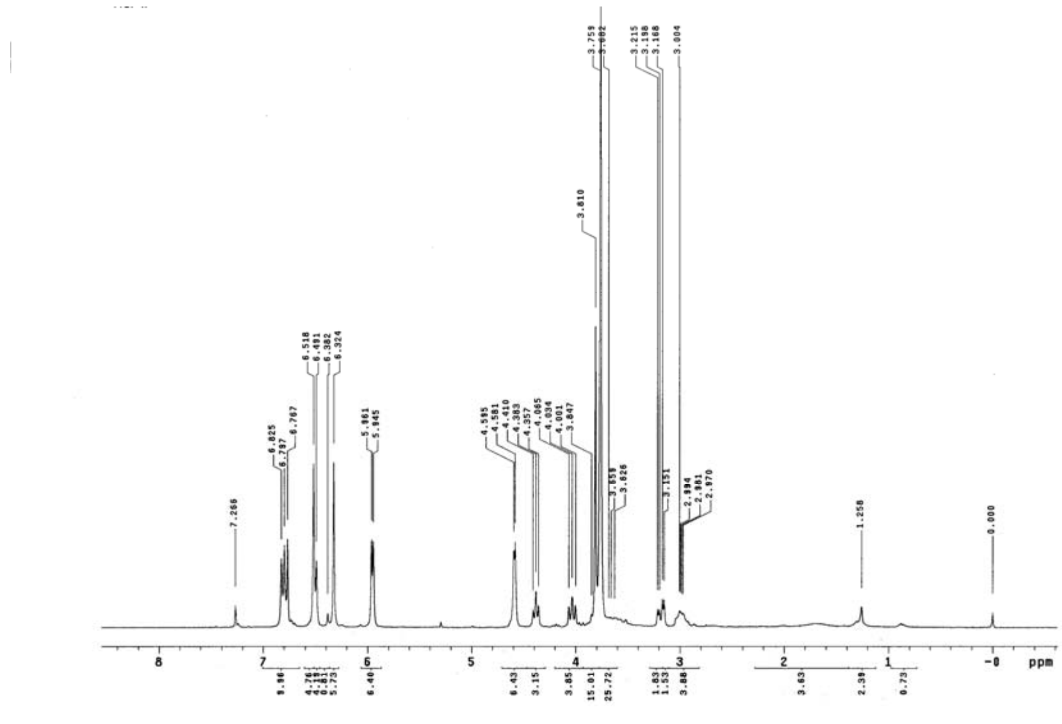

HMBC spectrums for Compound **12**.

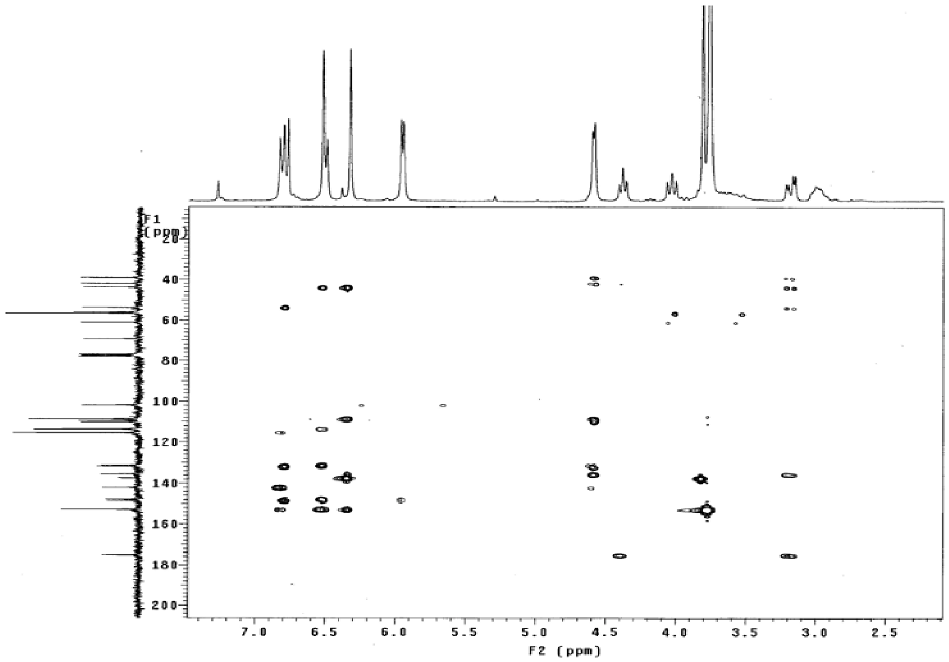

HSQC spectrums for Compound **12**.

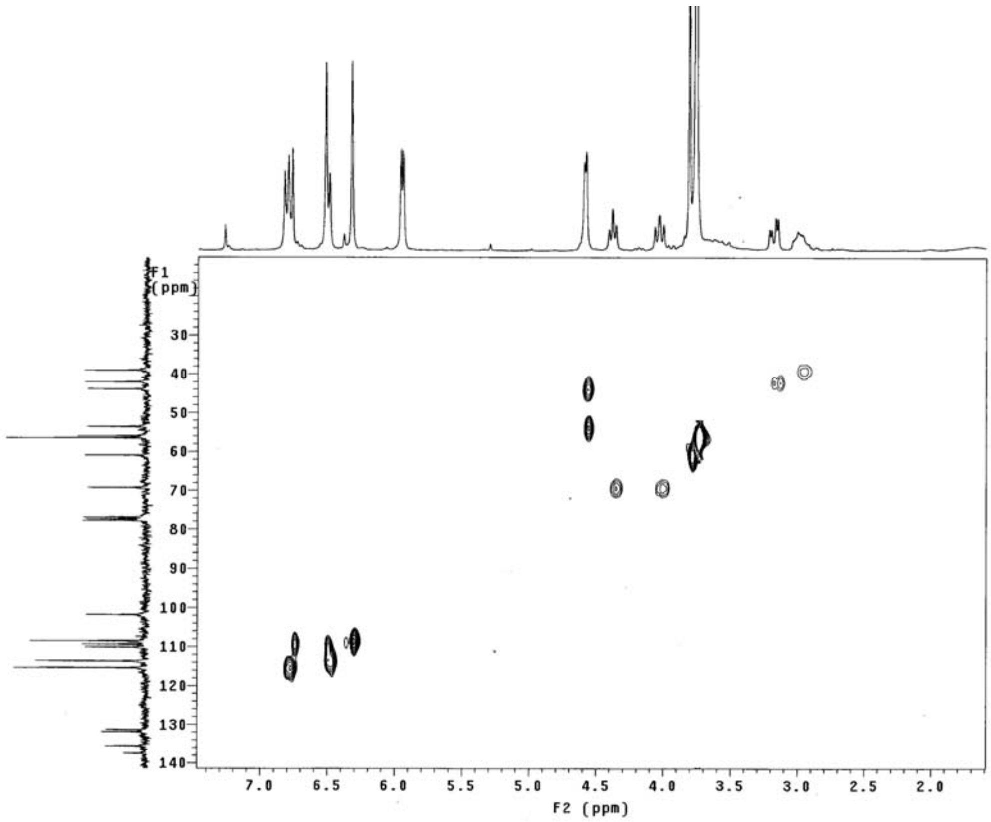

^{1}H-^{1}H COSY spectrums for Compound **12**.

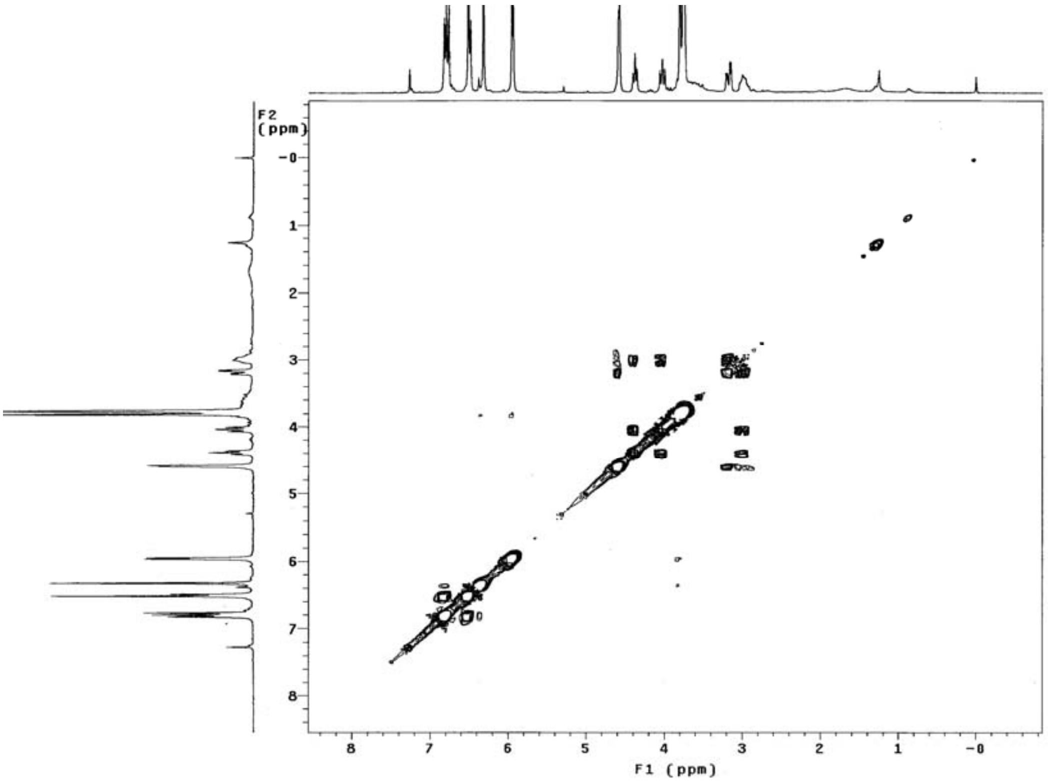

MS spectrums for compound **12**.

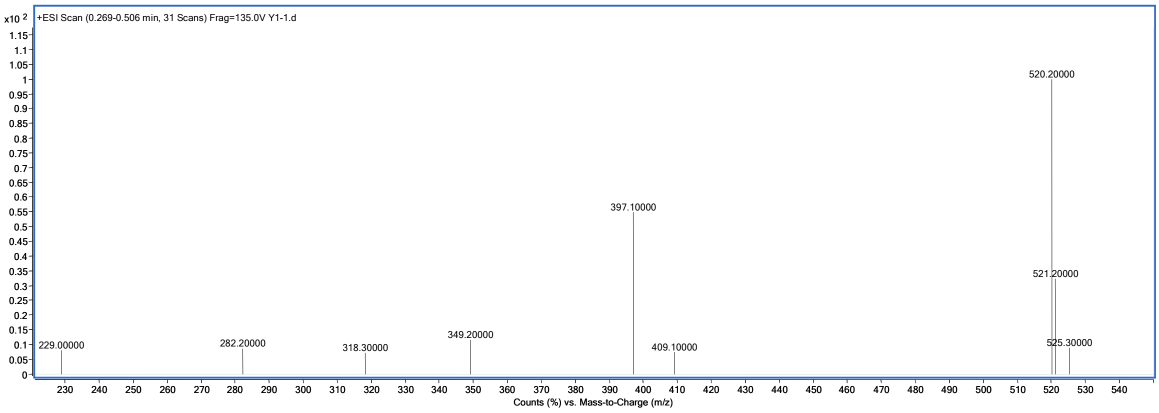

Compound 11 (4β-*NH*-(4-anisidine)-4-deoxy-podophyllotoxin): 67% yield as white solid, ^{1}H NMR (300 MHz, $CDCl_3$): δ 2.970-3.004 (m, 1H, 2-H) , 3.151 (dd, *J*=5.1Hz, 1H, 3-H), 3.759 (s, 9H, 3′, 5′-OCH_3, Ar-OCH_3), 3.810 (s, 3H, 4′-OCH_3), 4.034 (t, *J*=9.9 Hz, 1H, 11-H), 4.383 (t, *J*=7.8Hz, 1H, 11-H), 4.581 (d, *J*=4.2 Hz, 1H, 4-H), 5.945 (d, *J*=4.8 Hz, 2H, OCH_2O), 6.324 (s, 2H, ArH), 6.491 (s, 1H, ArH) , 6.518 (s, 2H, ArH), 6.767 (s, 1H, ArH) , 6.797 (s, 1H, ArH), 6.825 (s,1H, ArH)

^{13}C NMR (75 MHz, $CDCl_3$): δ 39.059, 41.999, 43.828, 53.591, 56.025, 56.489,60.962, 69.276, 101.745, 108.539, 109.383, 110.087, 113.632, 115.362, 131.315, 131.835, 135.535, 137.420, 142.020, 147.788, 148.351, 152.810, 175.220; MS (ESI): m/z: 520 $[M+H]^+$

^{13}C NMR spectrum of 4*β*-*NH*-(3-anisidine)-4-deoxy-podophyllotoxin **(13)**.

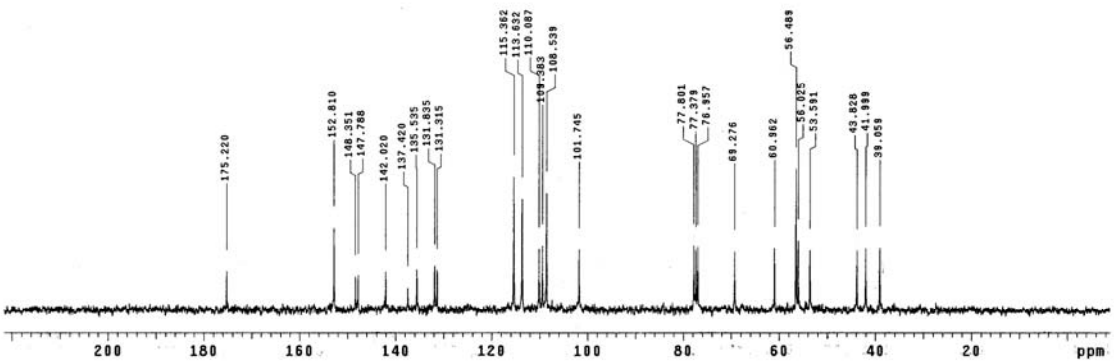

^1H NMR spectrum of Compound **13**.

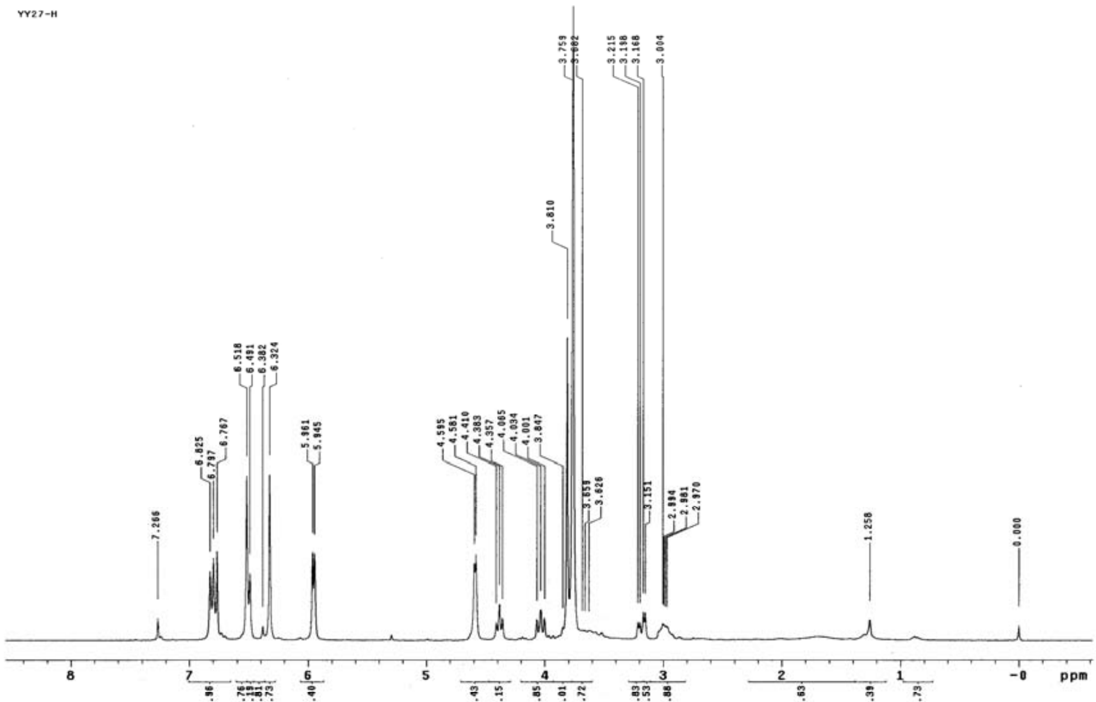

HMBC spectrums for Compound **13**.

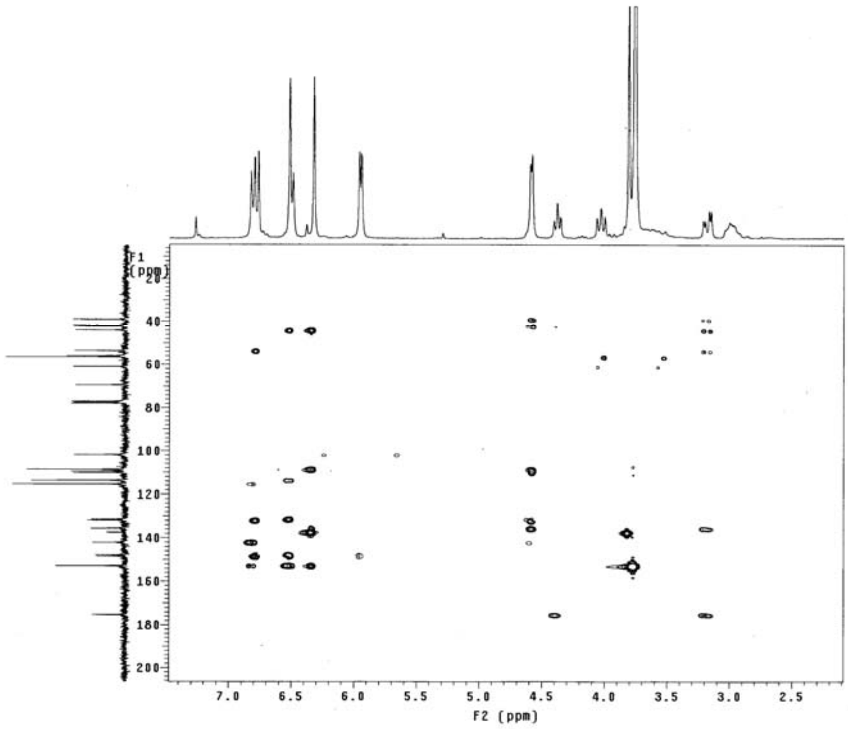

HSQC spectrums for Compound **13**.

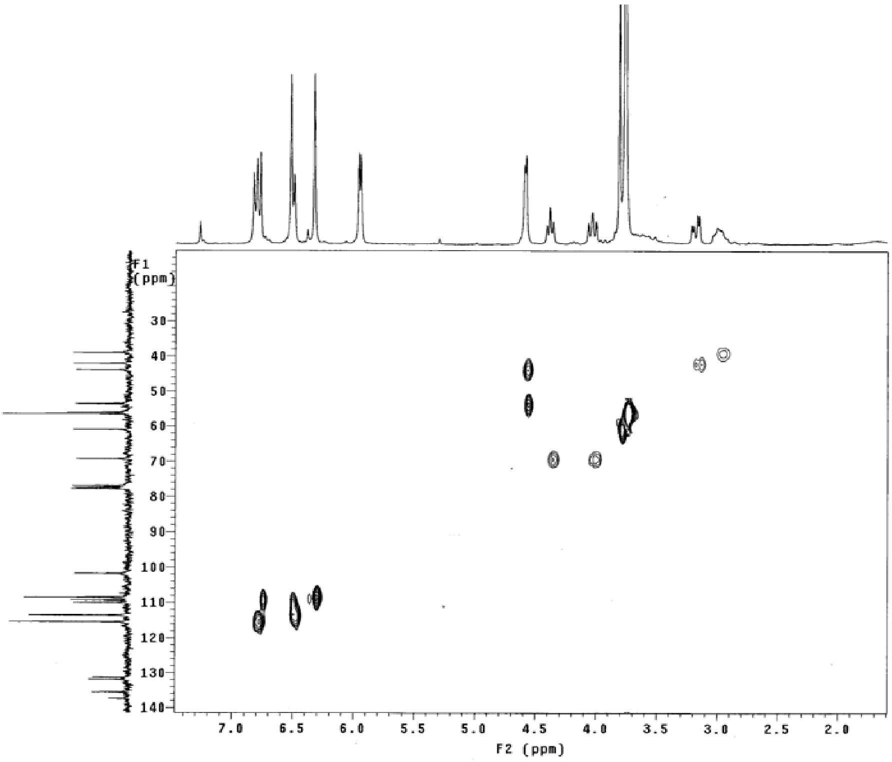

^{1}H-^{1}H COSY spectrums for Compound **13**.

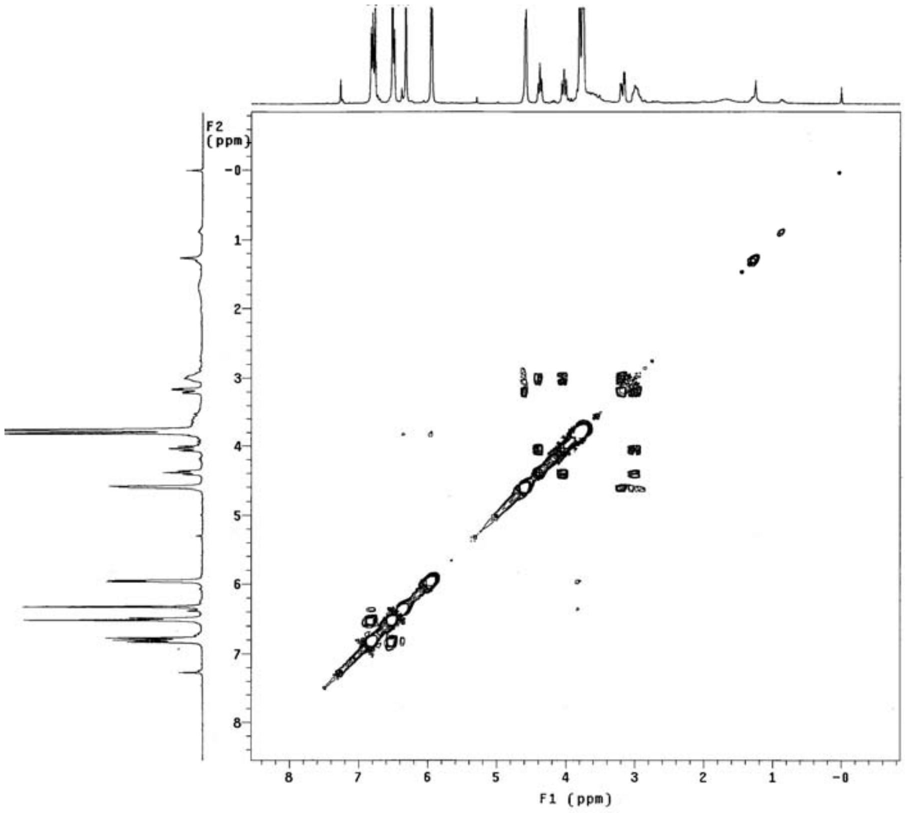

MS spectrums for Compound **13**.

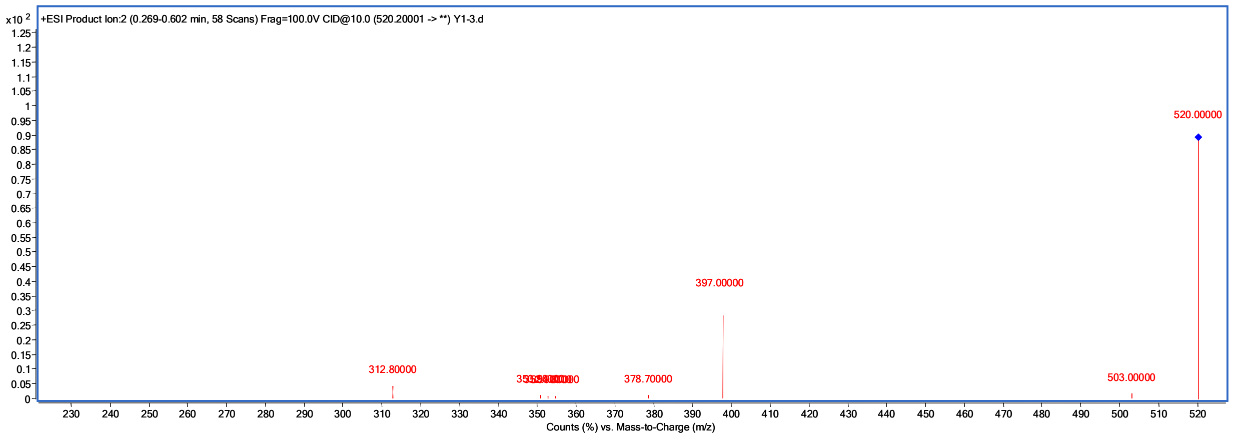

Compound 13 (4β-*NH*-(3-anisidine)-4-deoxy-podophyllotoxin): 57% yield as white solid, ^{1}H NMR (300 MHz, $CDCl_3$): δ 2.970-3.004 (m, 1H, 2-H) , 3.151 (dd, *J*=5.1Hz, 1H, 3-H), 3.759 (s, 9H, 3′, 5′-OCH_3, Ar-OCH_3), 3.810 (s, 3H, 4′-OCH_3), 4.034 (t, *J*=9.9 Hz, 1H, 11-H), 4.383 (t, *J*=7.8Hz, 1H, 11-H), 4.581 (d, *J*=4.2 Hz, 1H, 4-H), 5.945 (d, *J*=4.8 Hz, 2H, OCH_2O), 6.324 (s, 2H, ArH), 6.491 (s, 1H, ArH) , 6.518 (s, 2H, ArH), 6.767 (s, 1H, ArH) , 6.797 (s, 1H, ArH), 6.825 (s,1H, ArH) ^{13}C NMR (75 MHz, $CDCl_3$): δ 39.059, 41.999, 43.828, 53.591, 56.025, 56.489,60.962, 69.276,

101.745, 108.539, 109.383, 110.087, 113.632, 115.362, 131.315, 131.835, 135.535, 137.420, 142.020, 147.788, 148.351, 152.810, 175.220; MS (ESI): m/z: 520 $[M+H]^+$

^{13}C NMR spectrum of 4*β*-*NH*-(aniline)-4-deoxy-4′-demethylepipodophyllotoxin **(1′)**.

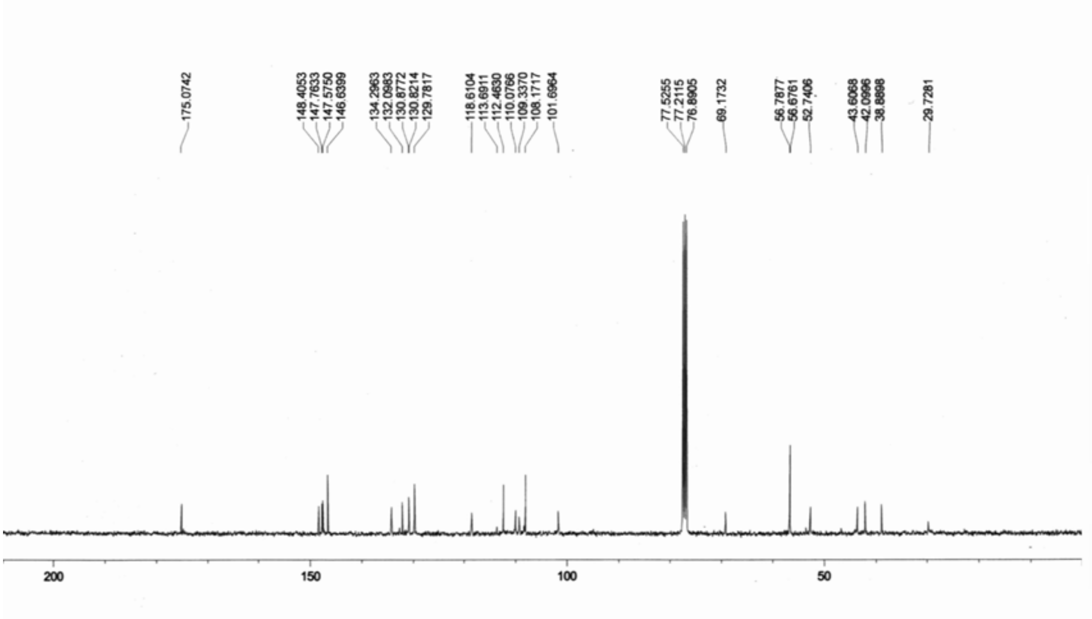

^1H NMR spectrum of Compound **1′**.

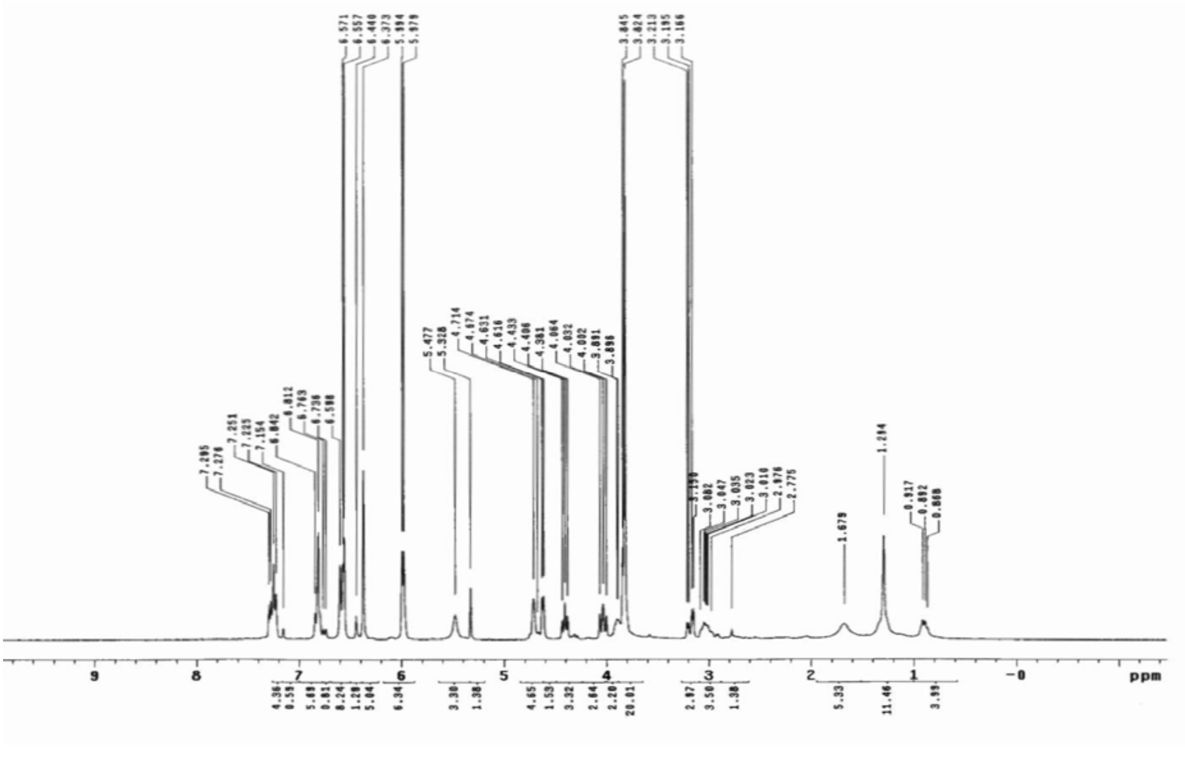

HMBC spectrums for Compound **1′**.

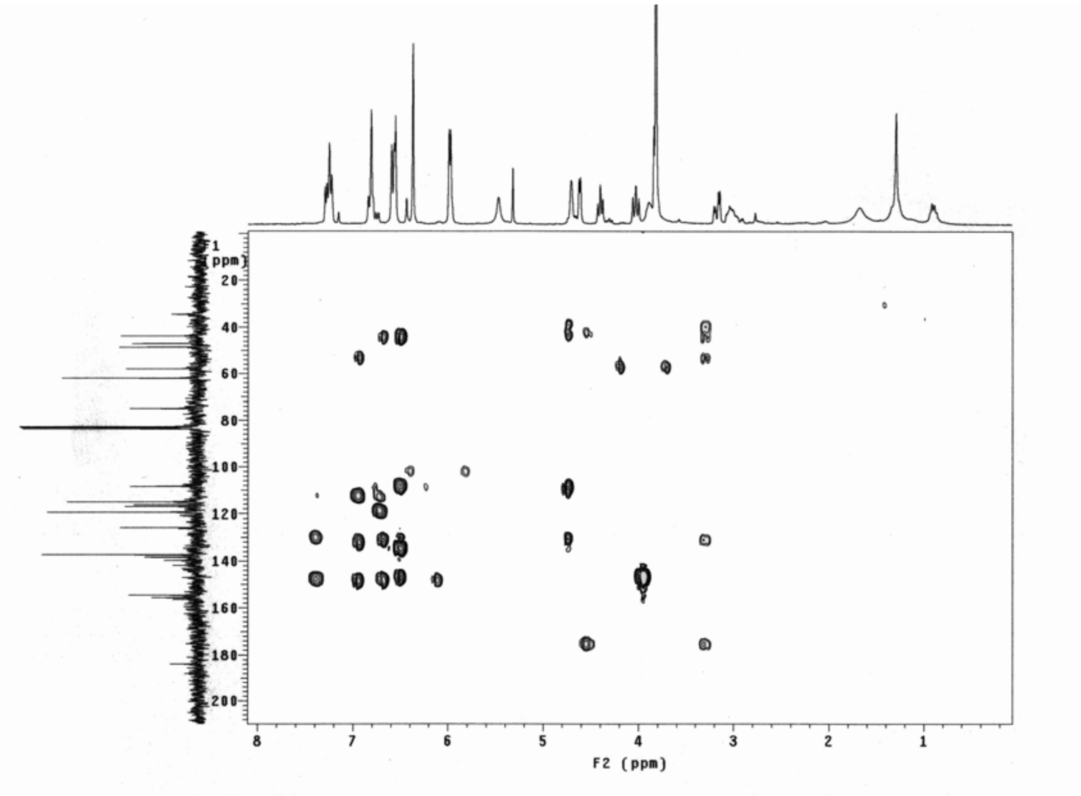

HSQC spectrums for Compound **1′**.

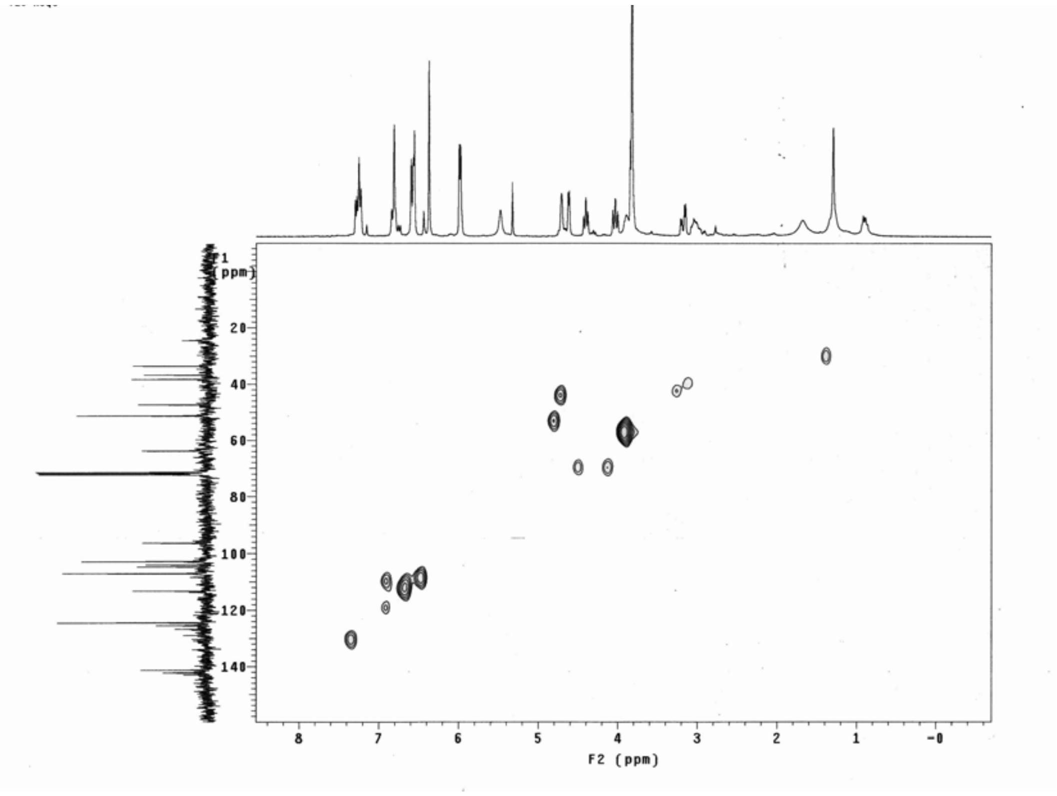

^{1}H-^{1}H COSY spectrums for Compound **1′**.

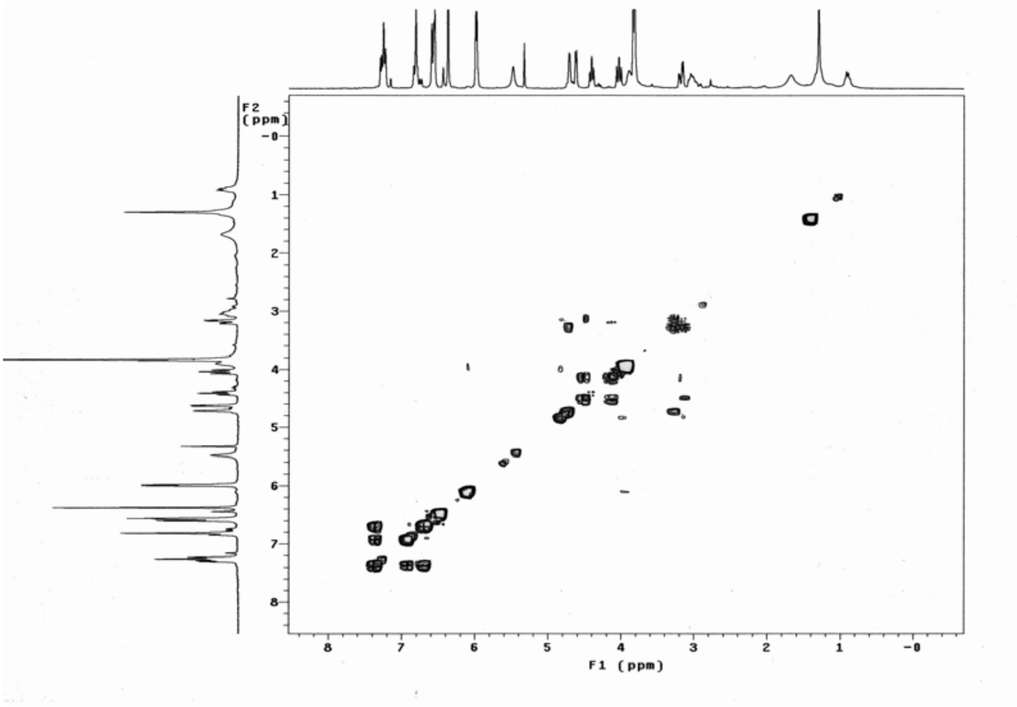

MS spectrums for compound Compound **1′**

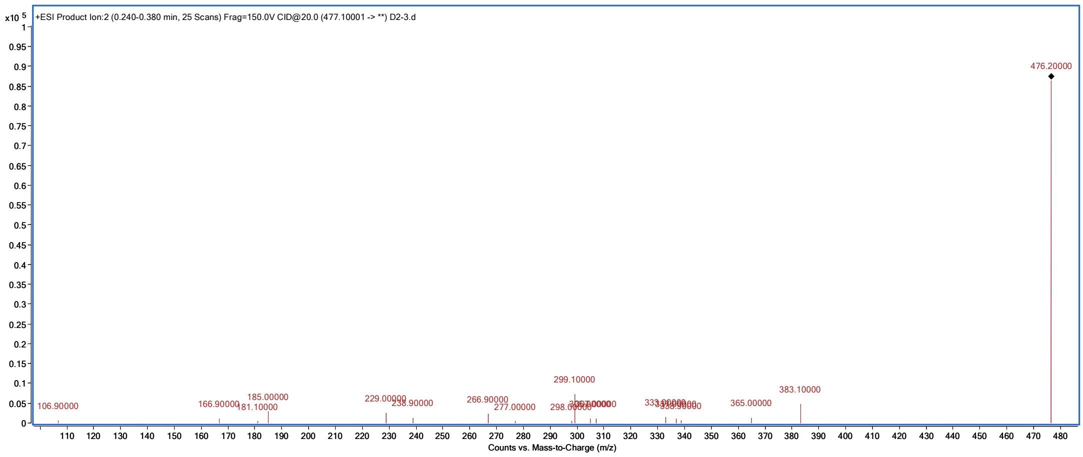

Compound 1′ (4β-*NH*-(aniline)-4-deoxy-4′-demethylepipodophyllotoxin): 81% yield as white solid, ^{1}H NMR (300 MHz, $CDCl_3$): δ 7.25 (m, 2H, ArH), 6.76 (s, 2H, ArH), 6.56 (s, 3H, ArH), 6.37 (s, 2H, ArH), 5.98 (d, *J*=4.5 Hz 2H, OCH_2O), 4.71 (s, 1H, 4-H,), 4.62 (d, *J*=4.5 Hz, 1H, 1-H), 4.41 (t, *J*=7.5 Hz, 1H, 11-H), 4.03 (t, *J*=9.0 Hz, 1H, 11-H), 3.82 (s, 6H, 3’, 5’-OCH_3), 3.17 (dd, *J*=4.8 Hz, 1H, 3-H), 2.98 (m, 1H, 2-H); ^{13}C NMR (75 MHz, $CDCl_3$): δ 175.07, 148.41, 147.76, 147.58, 146.64, 134.30, 132.10, 130.88, 130.82, 129.78, 118.61, 113.69, 112.46, 110.08, 109.34, 108.17, 101.70,

69.17, 56.68, 52.74, 43.61, 42.10, 38.89; MS (ESI): m/z: 476 [M+H]$^{+}$

^{13}C NMR spectrum of 4*β*-*NH*-(1-chloroaniline)-4-deoxy-4′-demethylepipodophyllotoxin **(3′)**.

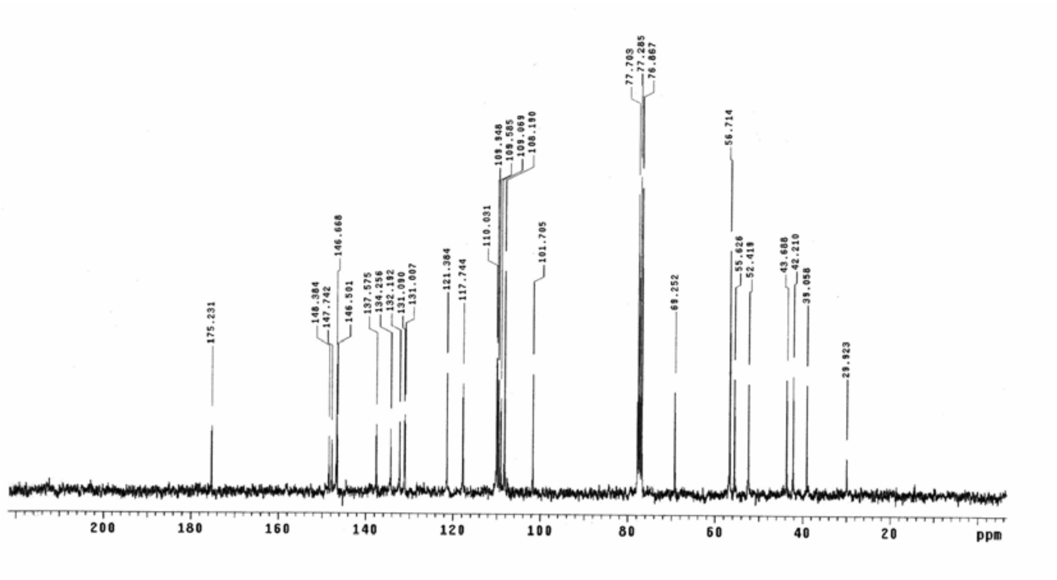

^{1}H NMR spectrum of Compound **3′**.

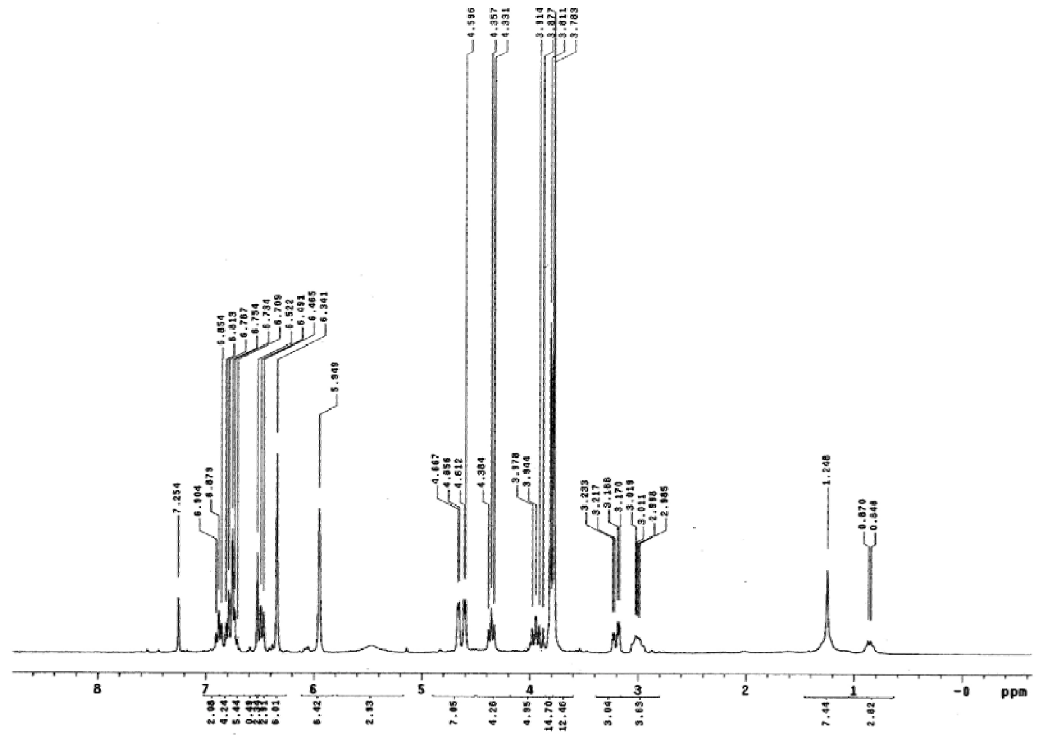

HMBC spectrums for Compound **3′**.

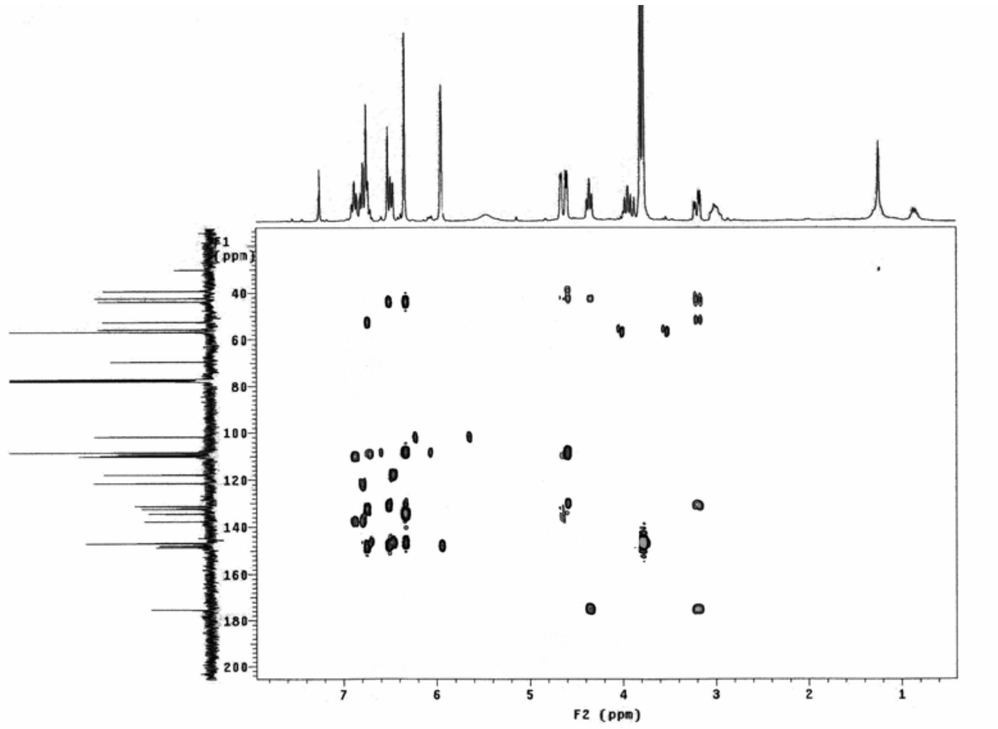

HSQC spectrums for Compound **3′**.

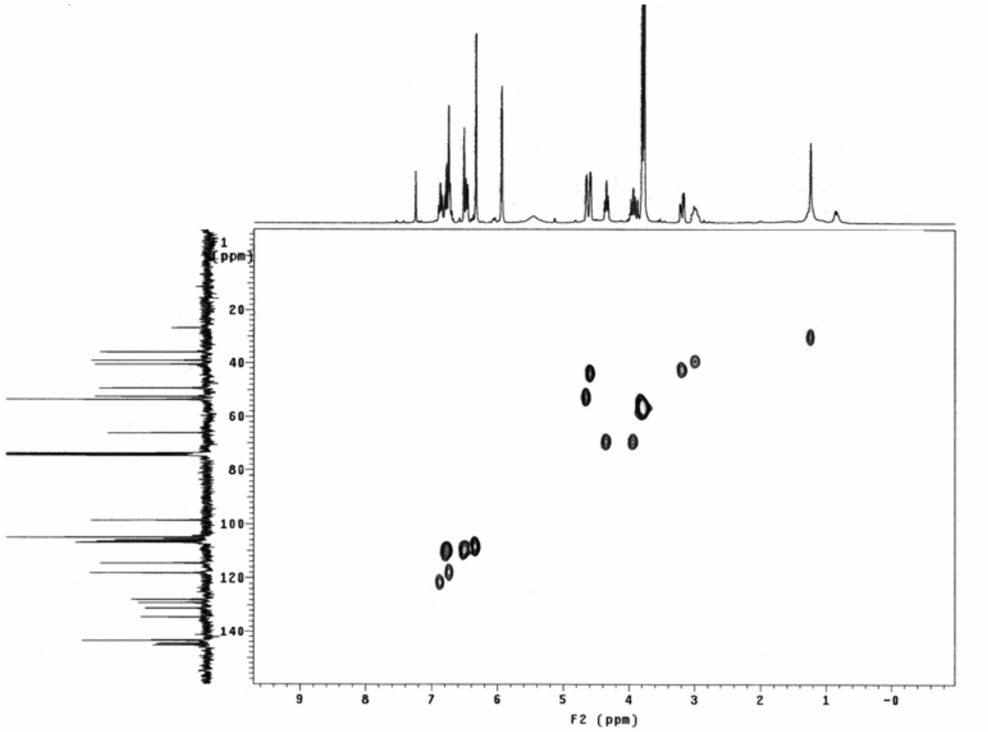

^{1}H-^{1}H COSY spectrums for Compound **3′**.

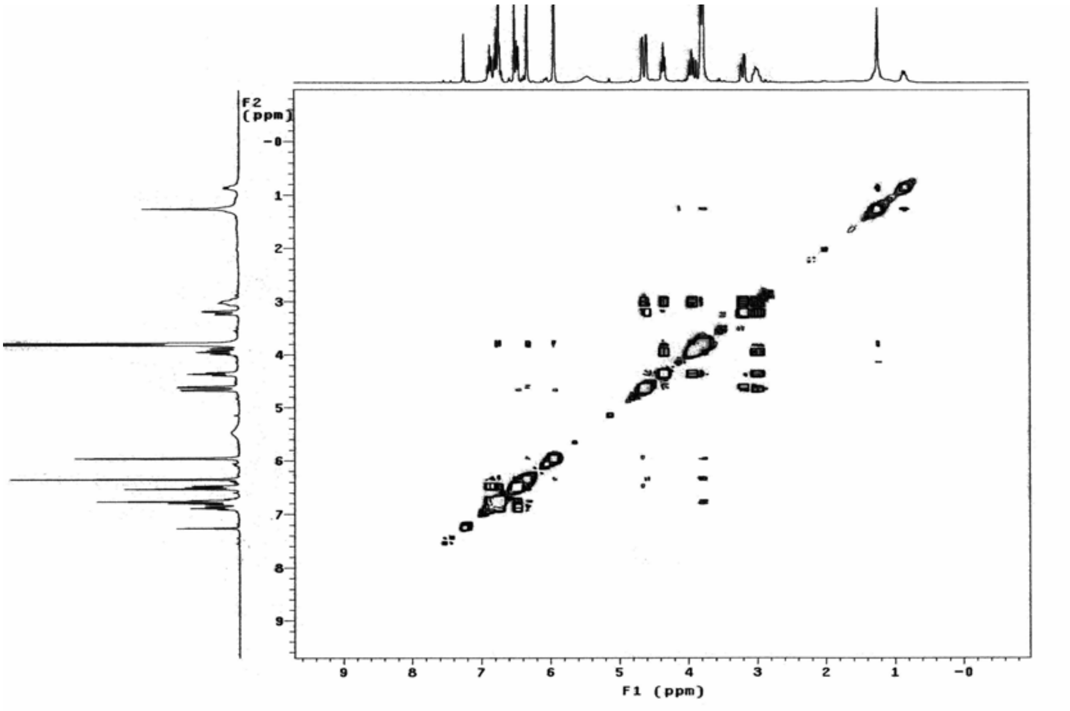

.

MS spectrums for Compound **3′**.

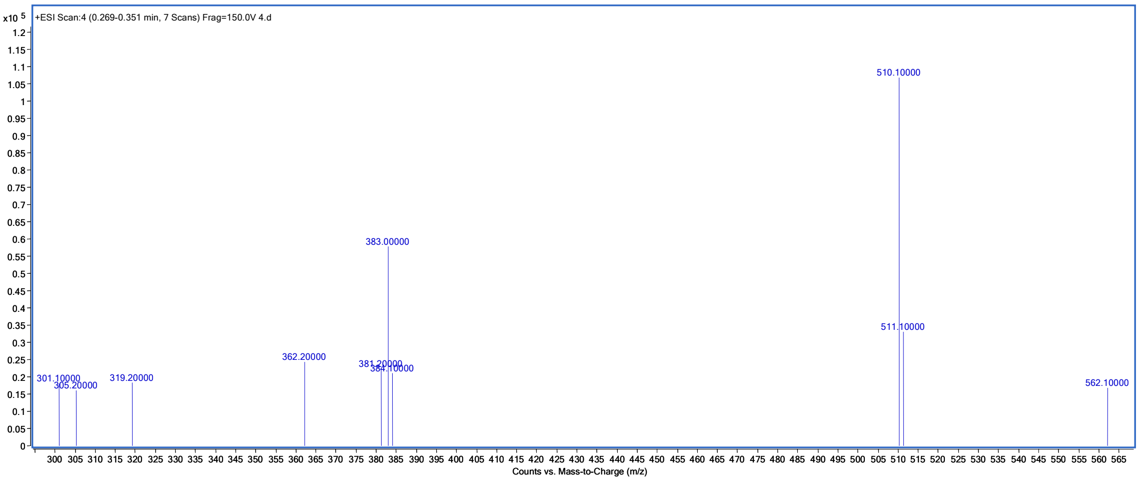

Compound 3′(4β-*NH*-(2-chloroaniline)-4-deoxy-4′-demethylepipodophyllotoxin): 76% yield as white solid, ^{1}H NMR (400 MHz, $CDCl_3$): δ 7.31 (t, *J*=8.0 Hz, 1H, ArH), 7.17 (t, *J*=8.0 Hz, 1H, ArH), 6.75 (s, 1H, ArH), 6.72 (t, *J*=8.0 Hz, 1H, ArH), 6.55 (d, *J*=8.0 Hz, 1H, ArH), 6.53 (s, 1H, ArH), 6.34 (s, 2H, ArH), 5.97 (s, 2H, OCH_2O), 4.62 (d, *J*=4.0 Hz, 1H, 1-H), 4.50 (d, *J*=4.0 Hz, 1H, 1-H), 4.37 (t, *J*=8.0 Hz, 1H, 11-H), 3.88 (t, *J*=8.0 Hz, 1H, 11-H), 3.78 (s, 6H, 3’, 5’-OCH_3), 3.14 (dd, *J*=4.0 Hz, 1H, 3-H), 3.01(m, 1H, 2-H); ^{13}C NMR (100 MHz, $CDCl_3$): δ 174.59, 148.39, 147.68,

146.44, 143.78, 134.09, 132.07, 130.51, 130.05, 129.68, 127.96, 119.09, 118.43, 110.41, 109.92, 109.11, 107.94, 101.57, 68.70, 56.48, 52.35, 43.41, 41.91, 38.58; MS (ESI): m/z: 510 $[M+H]^+$

^{13}C NMR spectrum of 4*β*-*NH*-(1-fluoroaniline)-4-deoxy-4′-demethylepipodophyllotoxin **(4′)**.

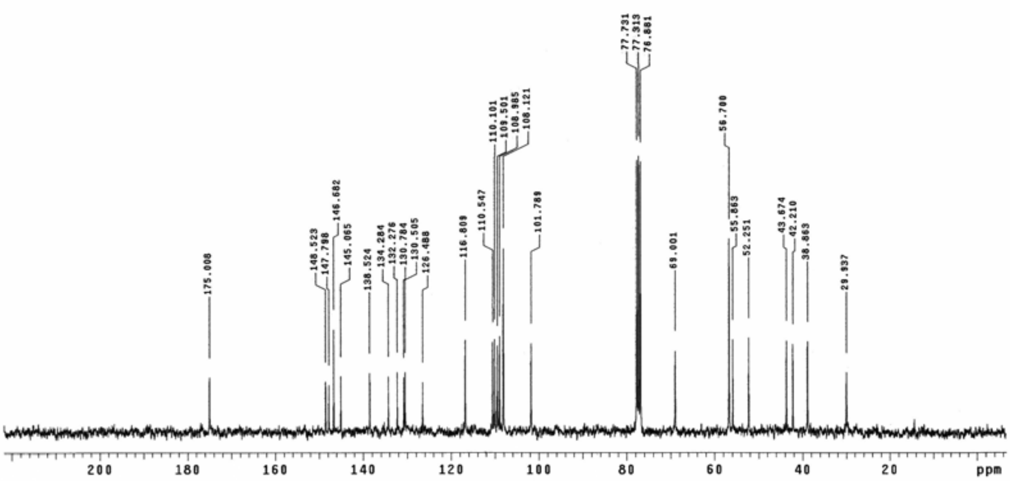

^{1}H NMR spectrum of Compound **4′**.

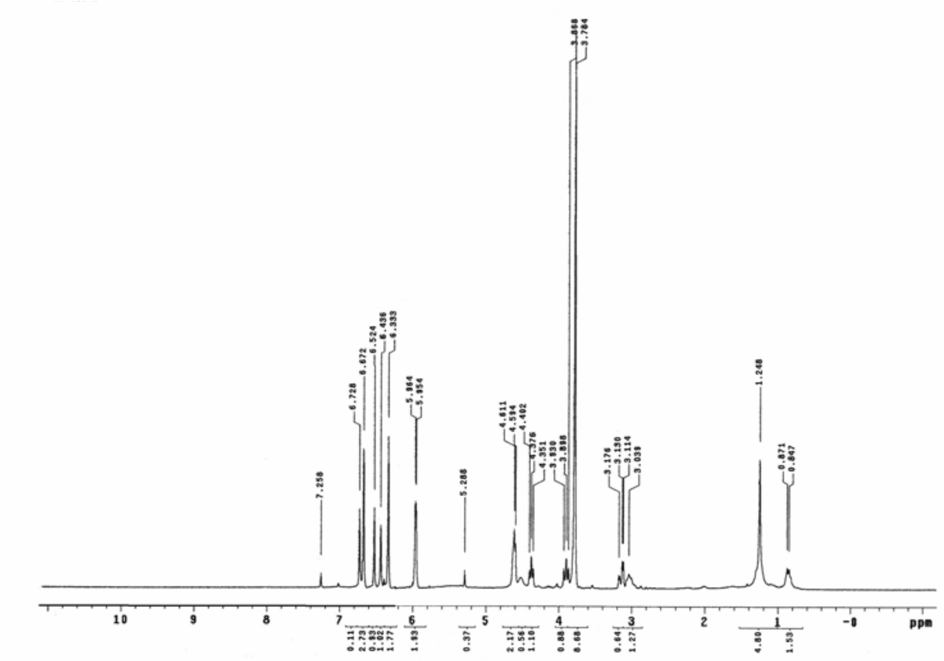

HMBC spectrums for Compound **4′**.

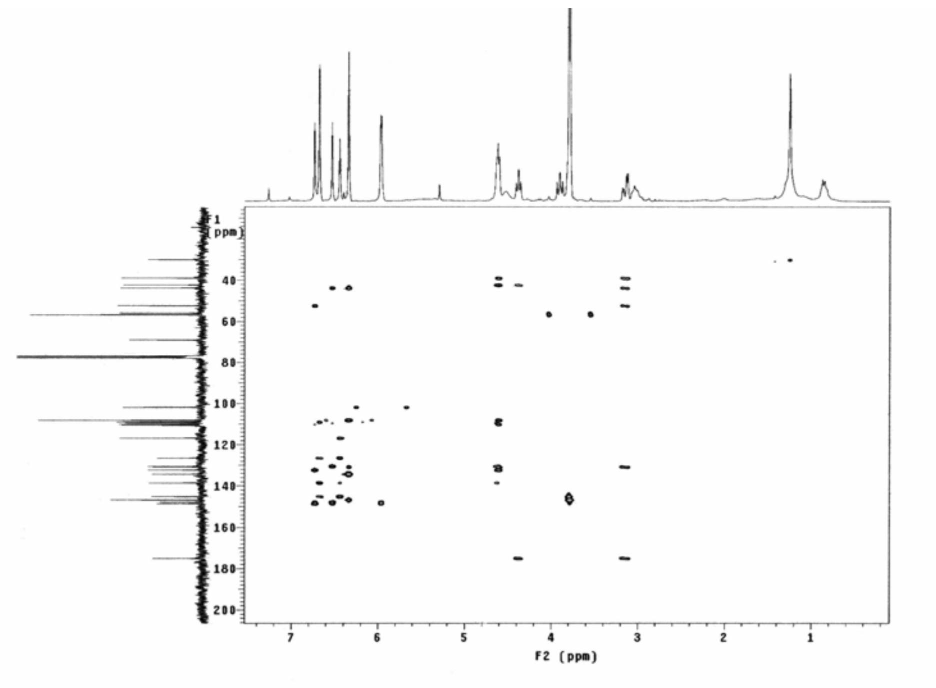

HSQC spectrums for Compound **4′**.

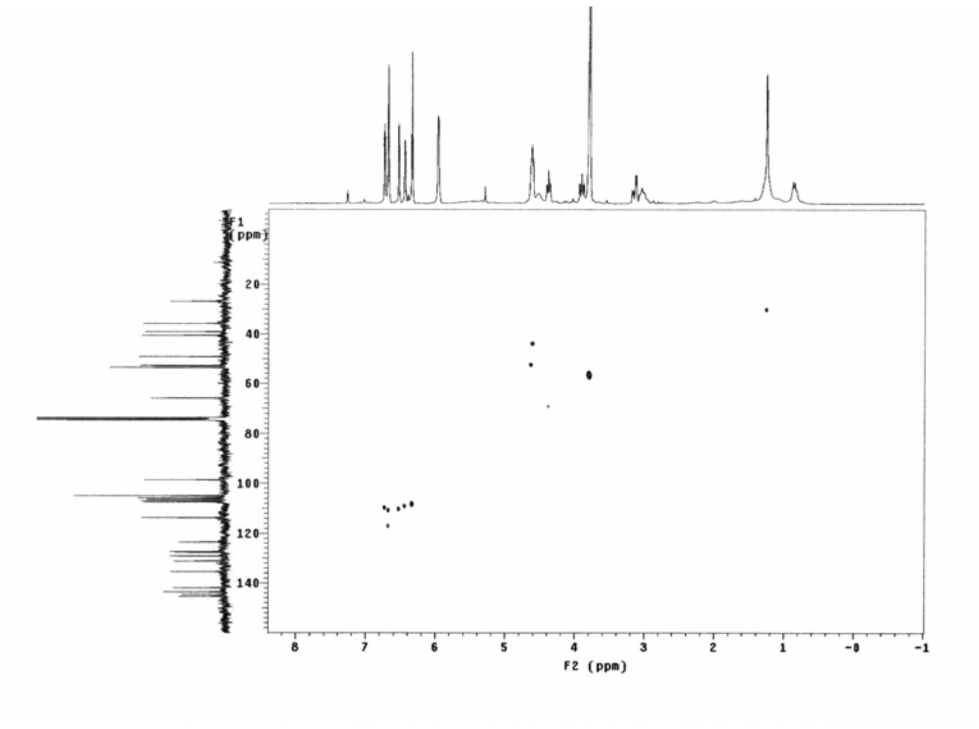

^{1}H-^{1}H COSY spectrums for Compound **4′**.

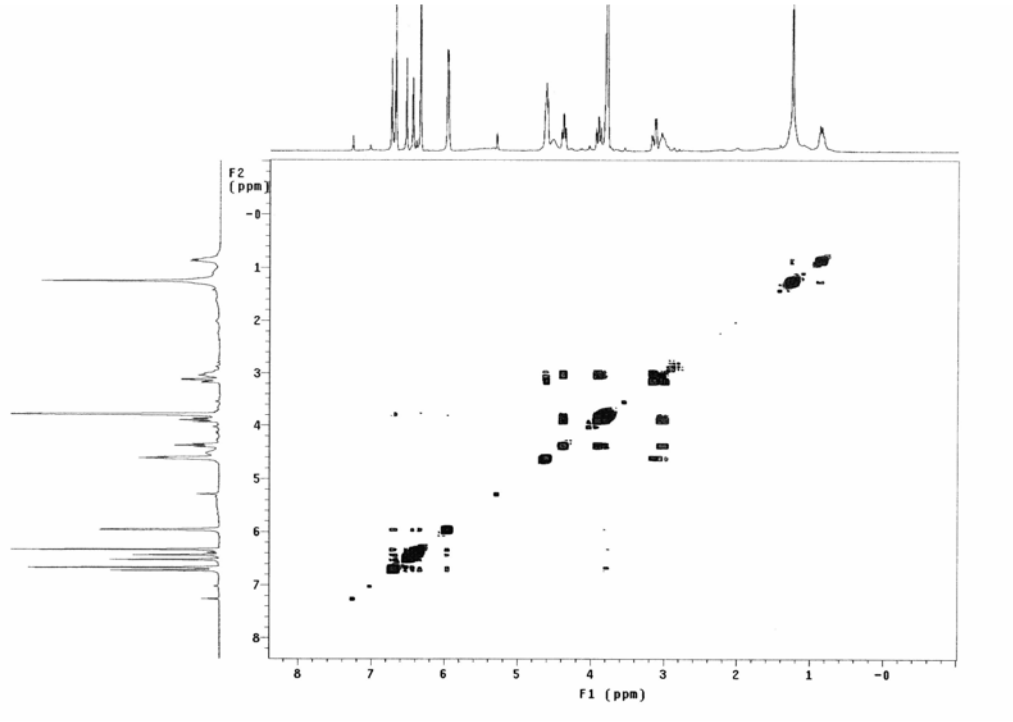

MS spectrums for Compound **4′**

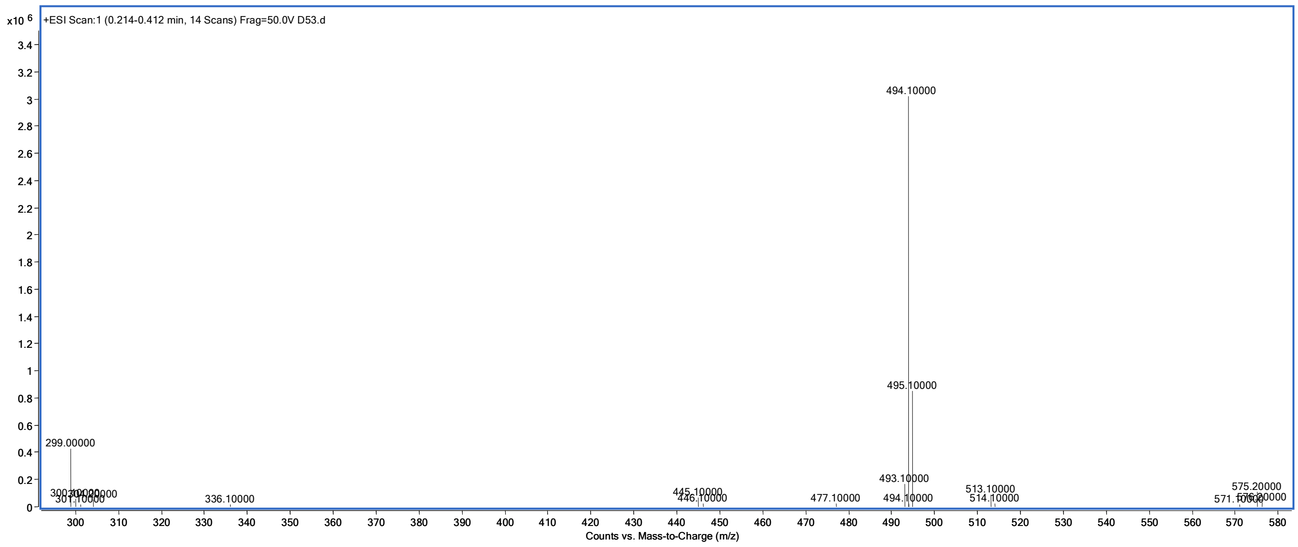

Compound 4′ (4β-*NH*-(2-fluoroaniline)-4-deoxy-4′-demethylepipodophyllotoxin): 89% yield as white solid, ^{1}H NMR (400 MHz, $CDCl_3$): δ7.02 (m, 2H, ArH), 6.75 (s, 1H, ArH), 6.72 (m, 1H, ArH), 6.58 (d, *J*=8.0 Hz, 1H, ArH), 6.53 (s, 1H, ArH), 6.33 (s, 2H, ArH), 5.94 (s, 2H, OCH_2O), 4.62 (d, *J*=4.0 Hz, 1H, 1-H), 4.56 (d, *J*=4.0 Hz, 1H, 1-H), 4.37 (t, *J*=8.0 Hz, 1H, 11-H), 3.97 (t, *J*=8.0 Hz, 1H, 11-H), 3.79 (s, 6H, 3’, 5’-OCH_3), 3.17 (dd, *J*=4.0 Hz, 1H, 3-H), 3.02 (m, 1H, 2-H); ^{13}C NMR (100 MHz, $CDCl_3$): δ174.67, 148.36, 147.64, 146.42, 135.84, 134.06, 131.99, 130.53,

130.18, 124.73, 117.88, 115.12, 114.93, 111.11, 109.94, 109.08, 107.92, 101.56, 68.73, 56.48, 52.34, 43.40, 41.83, 38.64; MS (ESI): m/z: 494 $[M+H]^+$

^{13}C NMR spectrum of 4*β*-*NH*-(2-bromoaniline)-4-deoxy-4′-demethylepipodophyllotoxin **(5′)**.

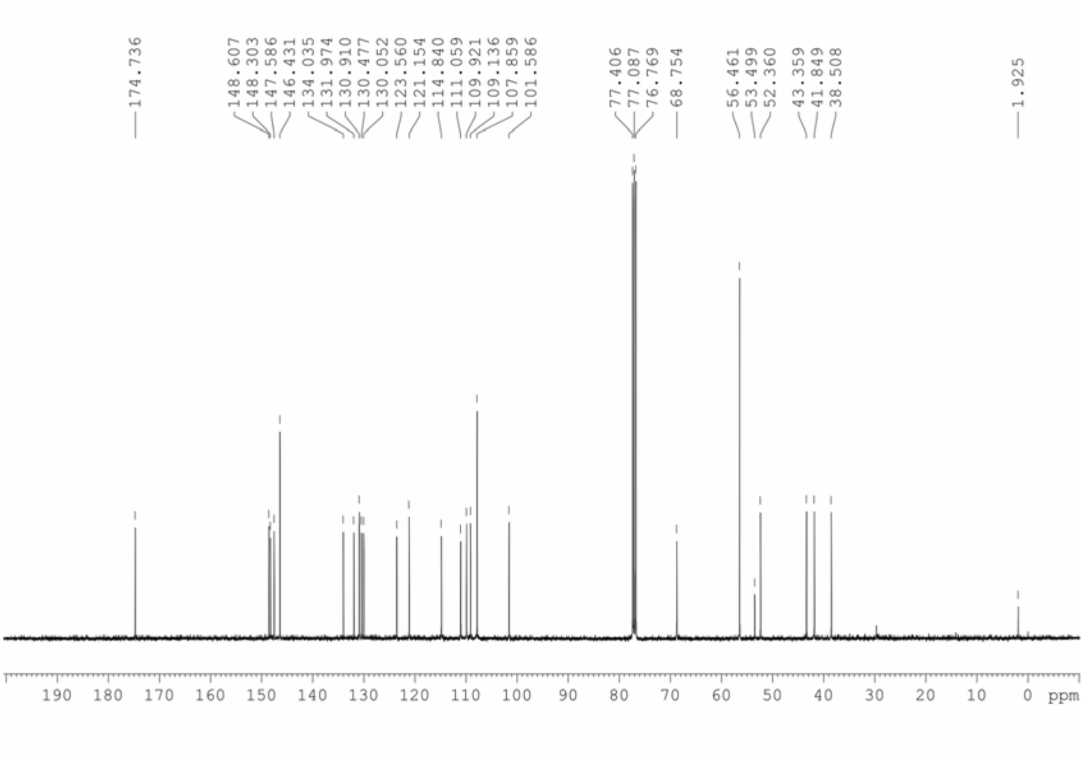

^{1}H NMR spectrum of Compound **5′**.

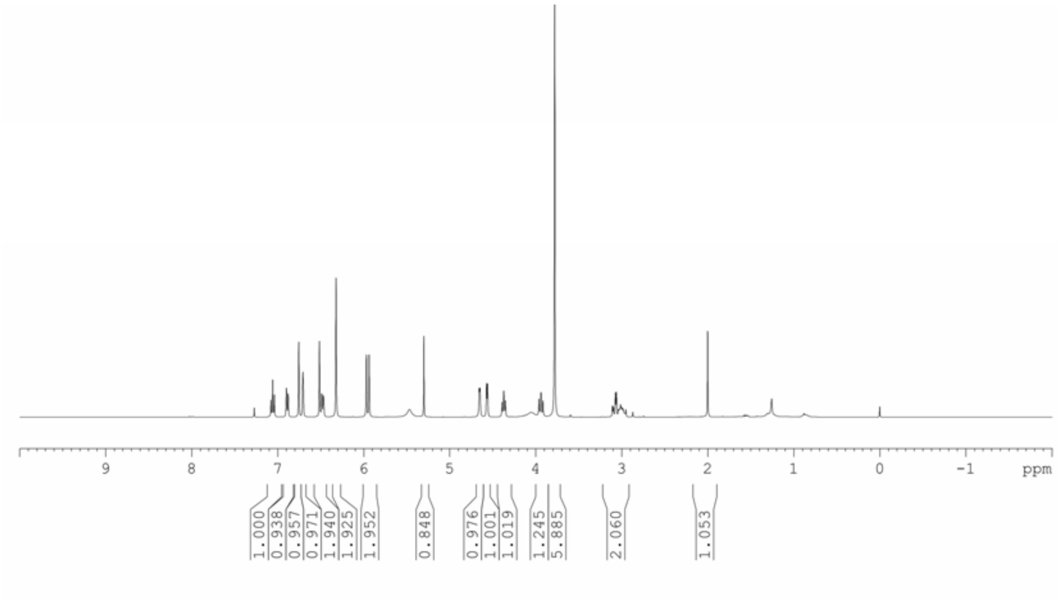

MS spectrums for Compound **5′**.

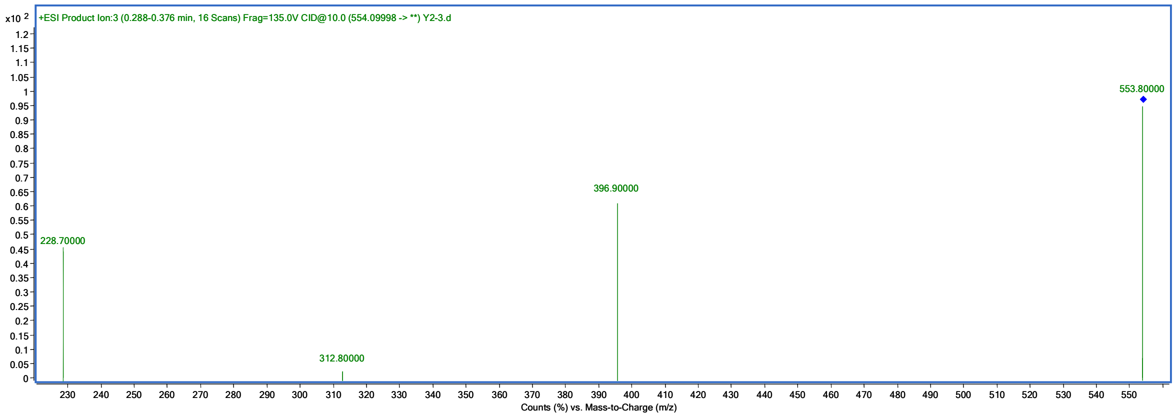

Compound 5′ (4β-*NH*-(3-bromoaniline)-4-deoxy-4′-demethylepipodophyllotoxin): 91% yield as white solid, ^{1}H NMR (400 MHz, $CDCl_3$): δ 7.07 (t, *J*=1.6 Hz, 1H, ArH), 6.89 (d, *J*=1.2 Hz, 1H, ArH), 6.72 (s, 1H, ArH), 6.62 (s, 1H, ArH), 6.47 (d, J=1.2 Hz, 1H, ArH), 6.55 (s, 1H, ArH), 6.35 (s, 2H, ArH), 5.95 (d, J=4.0 Hz, 2H, OCH_2O), 4.57(d, J=4.0 Hz, 1H, 4-H), 4.46(d, *J*=4.0 Hz,1H, 1-H), 4.39(t, *J*=6.3Hz, 1H, 11-H), 3.94(t, *J*=8.0 Hz, 1H, 11-H), 3.76(s, 6H, 3', 5'-OCH_3), 3.09(dd, J=4.8 Hz, 1H, 3-H), 2.97(m, 1H, 2-H); ^{13}C NMR (100 MHz, $CDCl_3$): δ 174.74, 148.61, 148.30, 147.59,146.43, 134.04, 131.97, 130.91, 130.48, 130.05, 123.56, 121.15, 114.84, 111.06, 109.92, 109.14, 107.86, 101.59, 68.75, 56.46, 52.36, 43.36, 41.85, 38.51; MS (ESI): m/z: 555 $[M+H]^+$

^{13}C NMR spectrum of 4*β*-*NH*-(2-chloroaniline)-4-deoxy-4′-demethylepipodophyllotoxin **(6′)**.

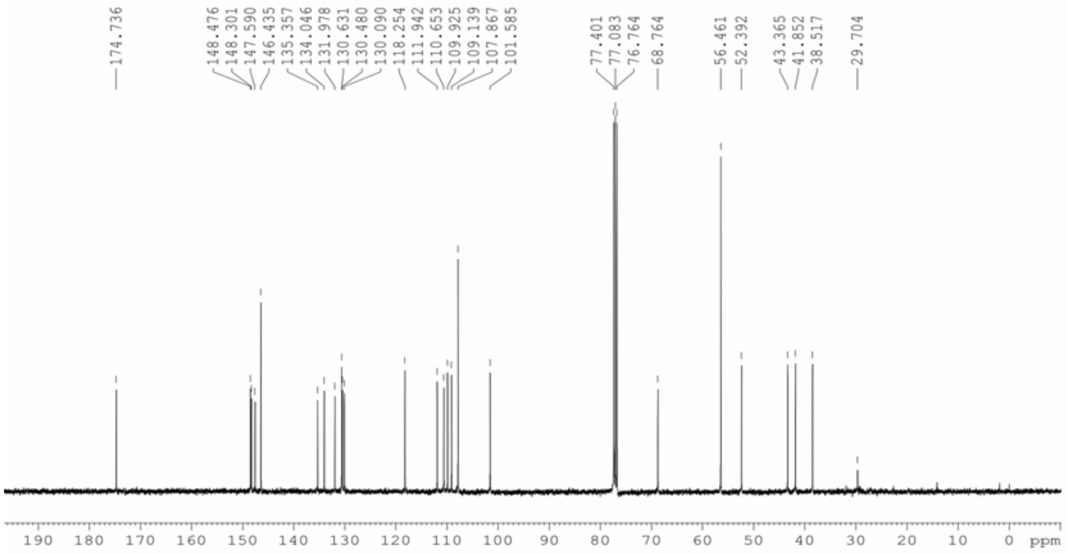

^{1}H NMR spectrum of Compound **6′**.

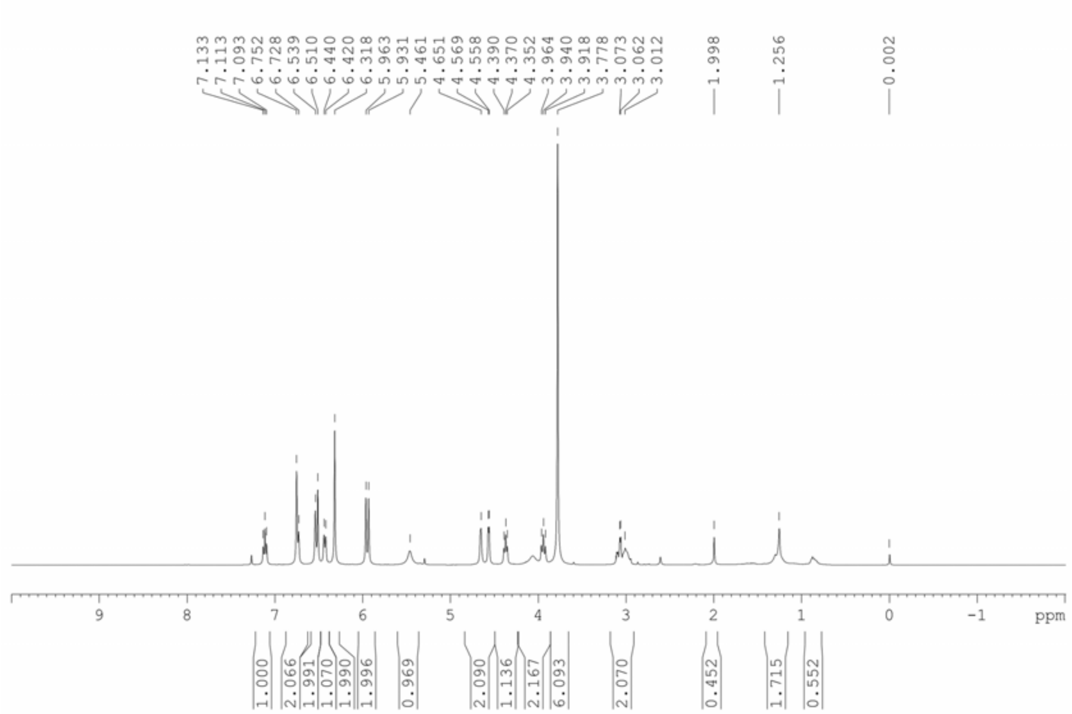

MS spectrums for Compound **6′**.

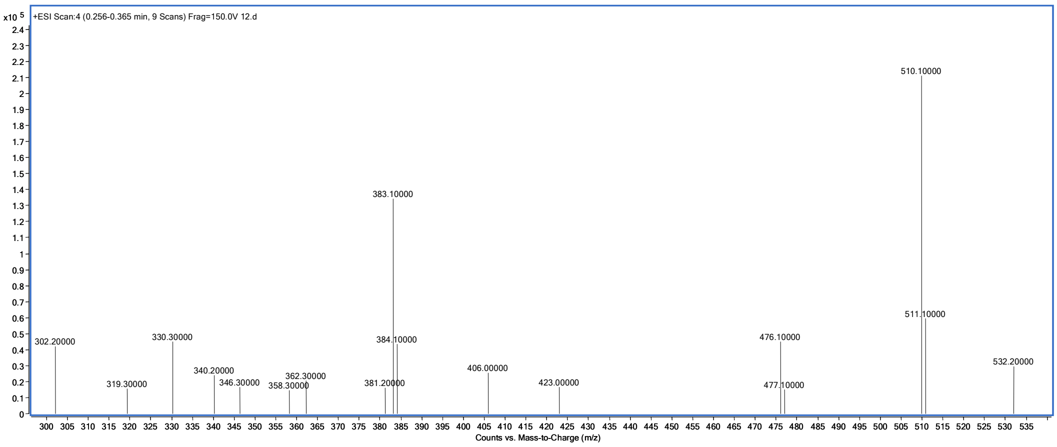

Compound 6′ (4β-*NH*-(3-chloroaniline)-4-deoxy-4′-demethylepipodophyllotoxin): 76% yield as white solid, ^{1}H NMR (400 MHz, $CDCl_3$): δ 7.11(t, *J*=8.0 Hz, 1H, ArH), 6.73 (m, 2H, ArH), 6.51 (s, 1H, ArH), 6.54 (s, 1H, ArH), 6.42 (d, *J*=1.6 Hz, 1H, ArH), 6.32 (s, 2H, ArH), 5.93 (d, *J*=12.8 Hz 2H, OCH_2O), 4.65 (s,1H, 1-H), 4.56 (t, *J*=4.4 Hz, 1H, 4-H), 4.37 (t, *J*=8.0 Hz, 1H, 11-H), 3.94 (t, *J*=9.2 Hz, 1H, 11-H), 3.78 (s, 6H, 3’, 5’-OCH_3), 3.06 (dd, *J*=4.8 Hz, 1H, 3-H), 3.01 (m, 1H, 2-H); ^{13}C NMR (100 MHz, $CDCl_3$): δ 174.74, 148.48, 148.30, 147.59, 146.44, 135.36, 134.05, 131.98,

130.63, 130.48, 130.09, 118.25, 111.94, 110.65, 109.93, 109.14, 107.87, 101.58, 68.76, 56.46, 52.39, 43.37, 41.85, 38.52; MS (ESI): m/z: 510 $[M+H]^+$

^{13}C NMR spectrum of 4*β*-*NH*-(2-fluoroaniline)-4-deoxy-4′-demethylepipodophyllotoxin **(7′)**.

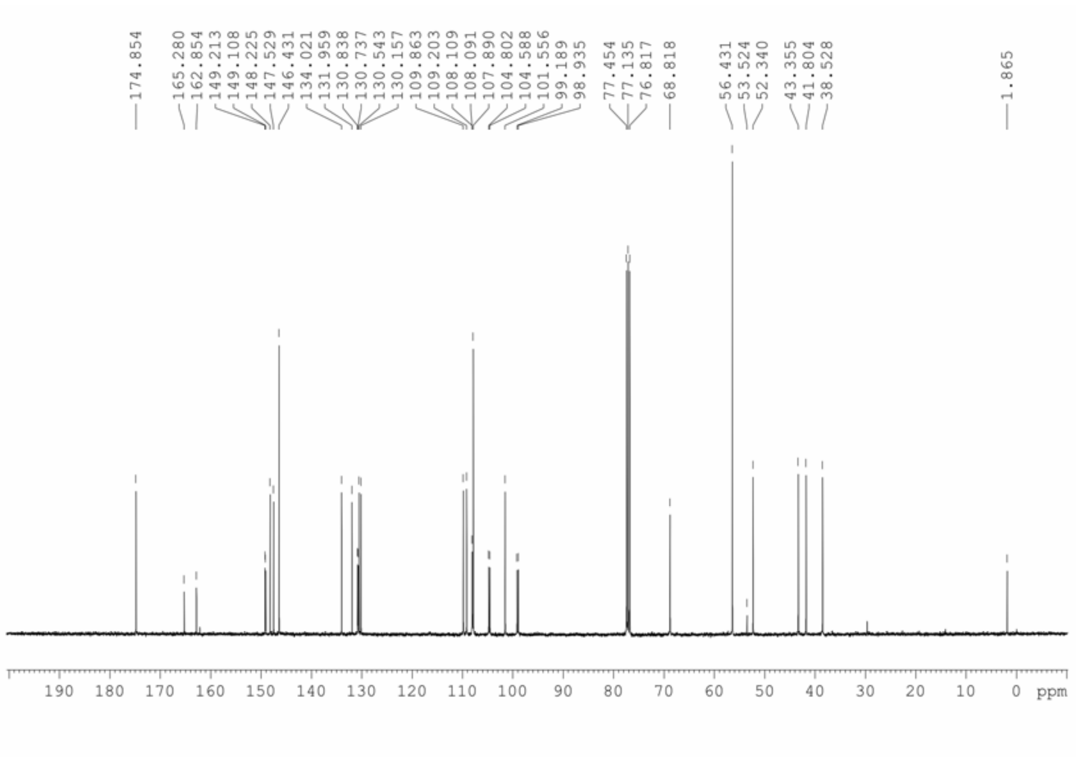

^{1}H NMR spectrum of Compound **7′**.

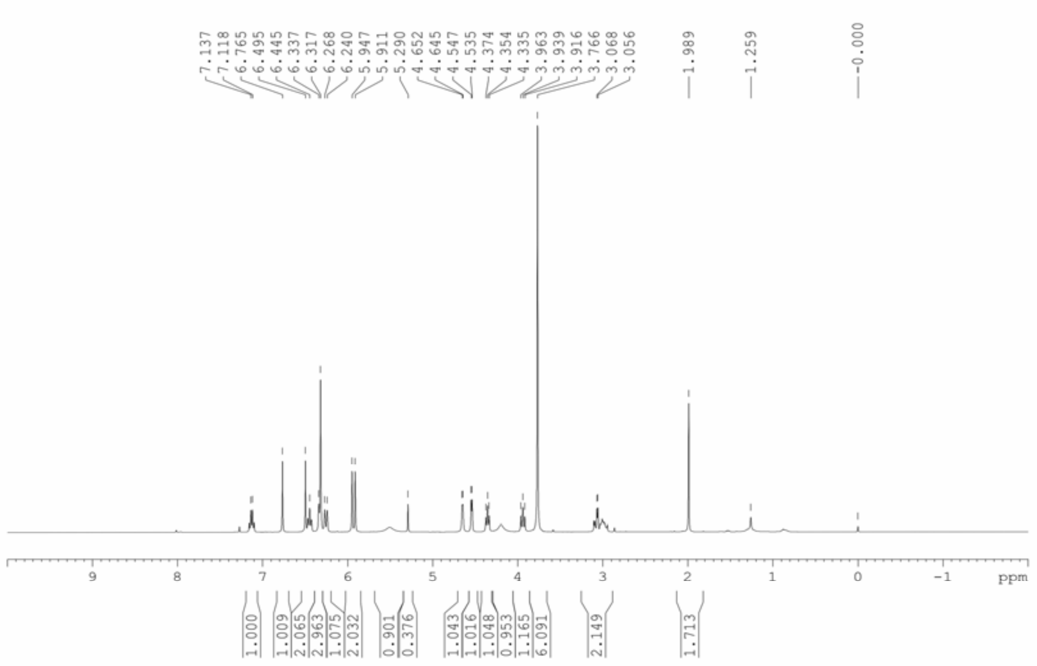

MS spectrums for Compound **7′**.

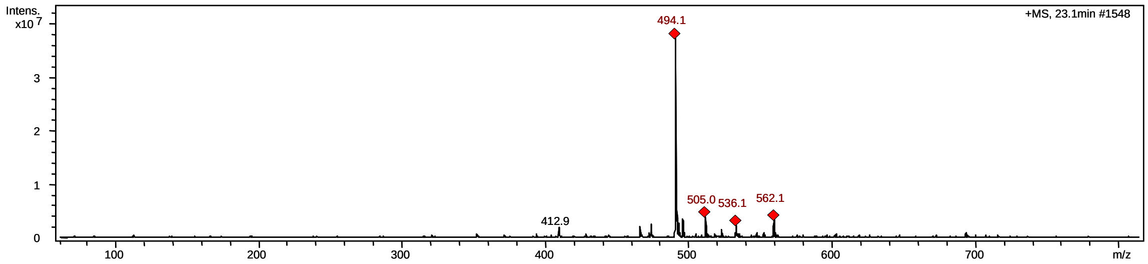

Compound 7′ (4β-*NH*-(3-fluoroaniline)-4-deoxy-4′-demethylepipodophyllotoxin): 84% yield as white solid, ^{1}H NMR (400 MHz, $CDCl_3$): 7.12 (d, J=7.6 Hz, 1H, ArH), 6.77 (s, H, ArH), 6.50 (s, 1H, ArH), 6.45 (t, *J*= 1.6 Hz, 1H, ArH), 6.34 (s, 1H, ArH), 6.32 (s, 2H, ArH), 6.24 (d, *J*=11.2 Hz, 1H, ArH), 5.91 (d, *J*=14.2 Hz, 2H, OCH_2O), 4.65 (t, *J*=5.6 Hz,1H, 1-H), 4.54 (t, *J*=4.8 Hz, 1H, 4-H), 4.35 (t, *J*=7.6 Hz, 1H, 11-H), 3.94 (t, *J*=9.4 Hz, 1H, 11-H), 3.77 (s, 6H, 3', 5'-OCH_3), 3.06 (dd, *J*=4.8 Hz, 1H, 3-H), 3.00 (m, 1H, 2-H); ^{13}C NMR (75 MHz, $CDCl_3$): δ 174.85, 149.11, 148.23, 147.53, 146.43, 134.02, 131.795, 130.54, 130.16, 109.86, 109.20, 108.09, 107.89, 104.59, 101.56, 68.82, 56.43, 52.340, 43.36, 41.80, 38.53; MS (ESI): m/z: 494 $[M+H]^+$

^{13}C NMR spectrum of 4*β*-*NH*-(3-bromoaniline)-4-deoxy-4′-demethylepipodophyllotoxin **(8′)**.

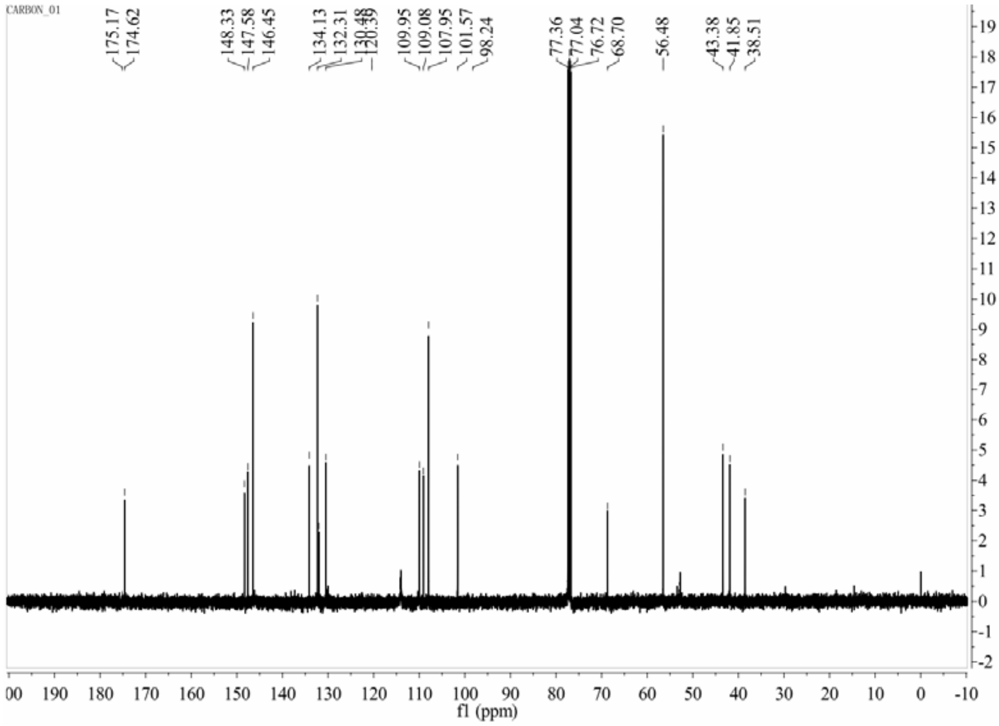

^1H NMR spectrum of Compound **8′**.

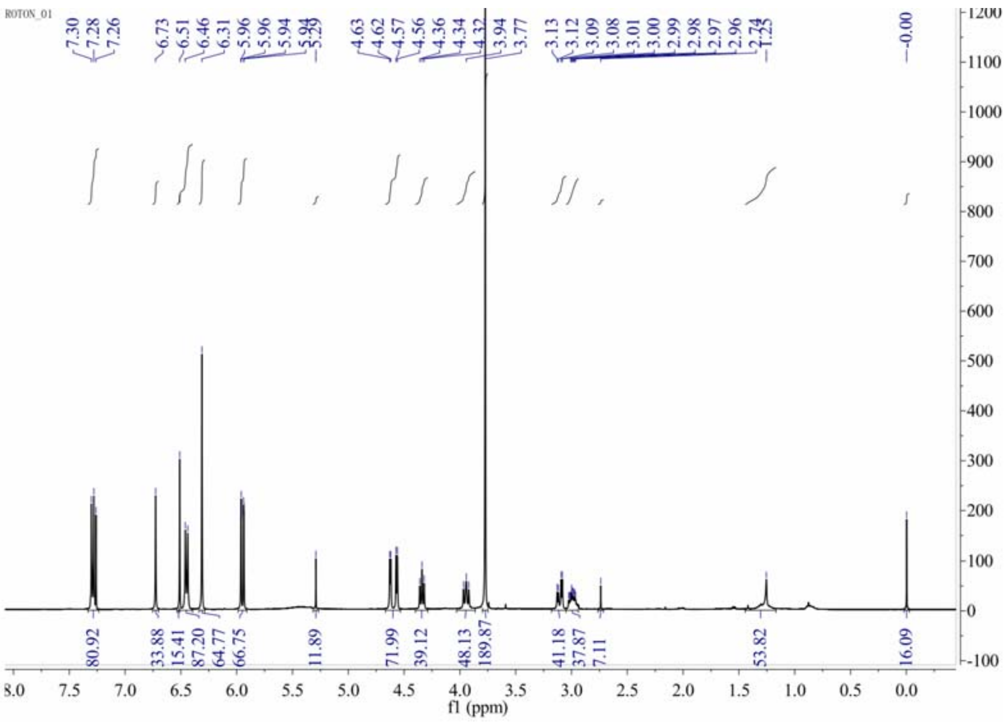

HMBC spectrums for Compound **8′**.

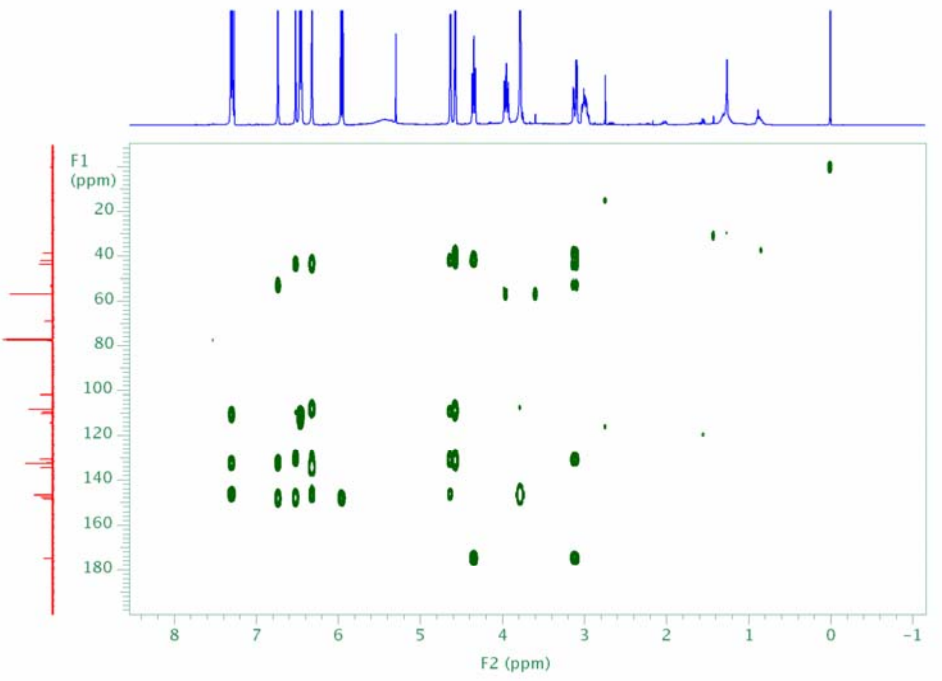

41.21 0.46 2212.7

HSQC spectrums for Compound **8′**.

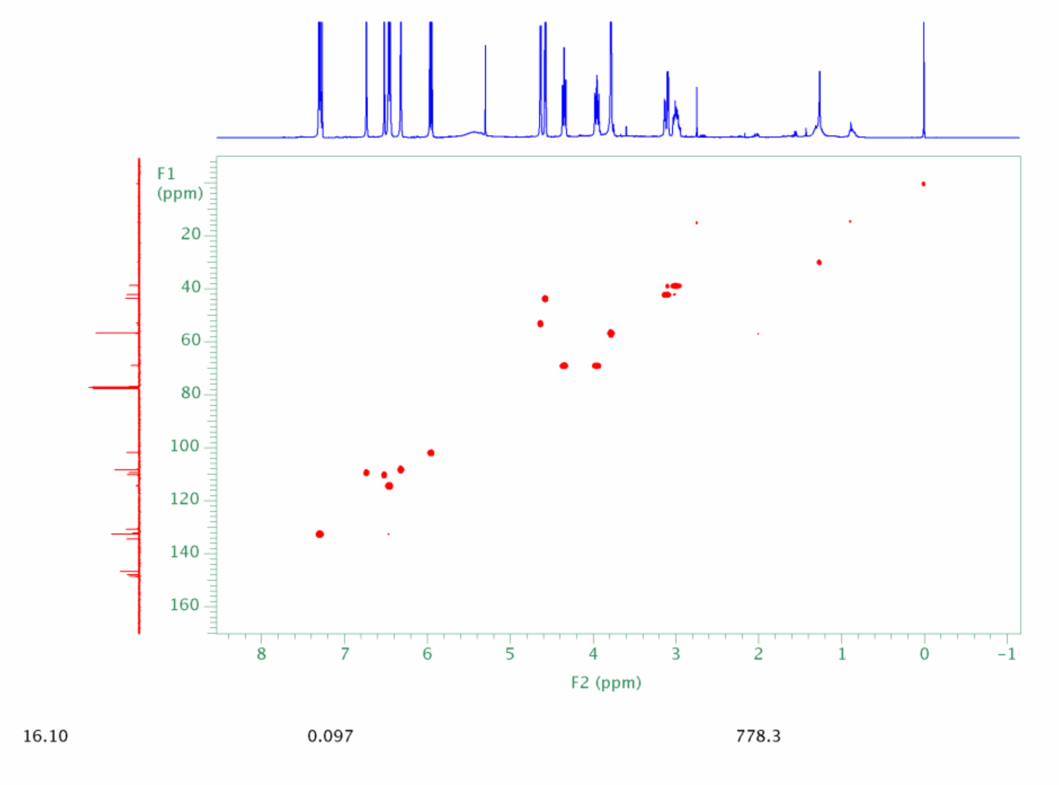

^{1}H-^{1}H COSY spectrums for Compound **8′**

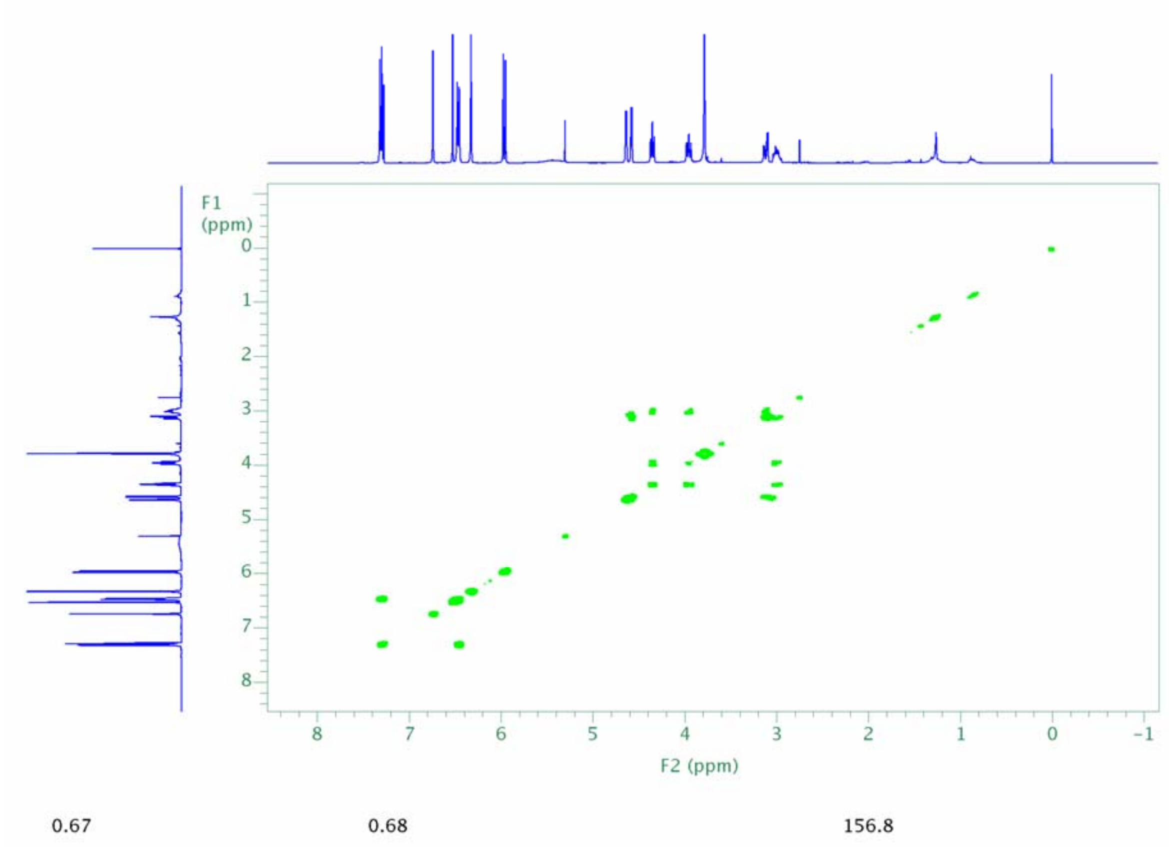

MS spectrums for Compound **8′**.

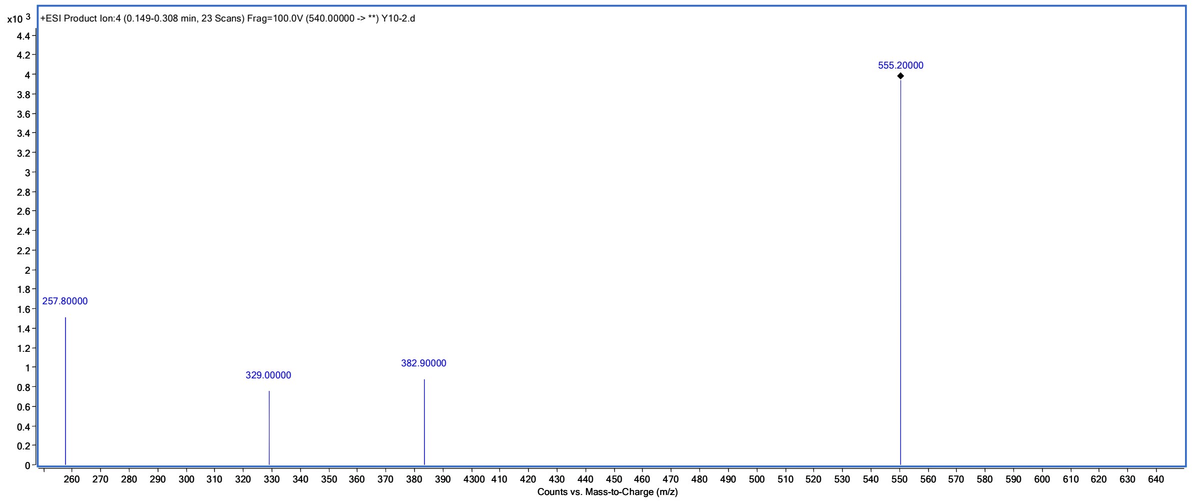

Compound 8′(4β-*NH*-(4-bromoaniline)-4-deoxy-4′-demethylepipodophyllotoxin): 91% yield as white solid, ^{1}H NMR (400 MHz, $CDCl_3$): δ 7.28 (d, *J*=8.0 Hz, 2H, ArH), 6.73 (s, 1H, ArH), 6.51 (s, 1H, ArH), 6.46 (d, *J*=8.0 Hz, 2H, ArH), 6.31 (s, 2H, ArH), 5.94 (d, *J*=8.0 Hz, 2H, OCH_2O), 4.62 (d, *J*=4.0 Hz,1H, 4-H), 4.56 (d, *J*=4.0 Hz, 1H, 1-H), 4.34 (t, *J*=8.0Hz, 1H, 11-H), 3.94 (t, *J*=8.0 Hz, 1H, 11-H), 3.77 (s, 6H, 3’, 5’-OCH_3), 3.08 (dd, *J*=8.0 Hz, 1H, 3-H), 3.01 (m, 1H, 2-H); ^{13}C NMR (100 MHz, $CDCl_3$): δ 174.62, 148.33, 147.58, 146.46, 137.25, 134.13, 132.31, 131.97, 130.48, 130.37, 113.78, 113.21, 109.90, 109.12, 107.95, 101.57, 68.70, 56.48, 52.58, 43.38, 41.85, 38.51; MS (ESI): m/z: 555 $[M+H]^+$

^{13}C NMR spectrum of 4*β*-*NH*-(3-chloroaniline)-4-deoxy-4′-demethylepipodophyllotoxin **(9′)**.

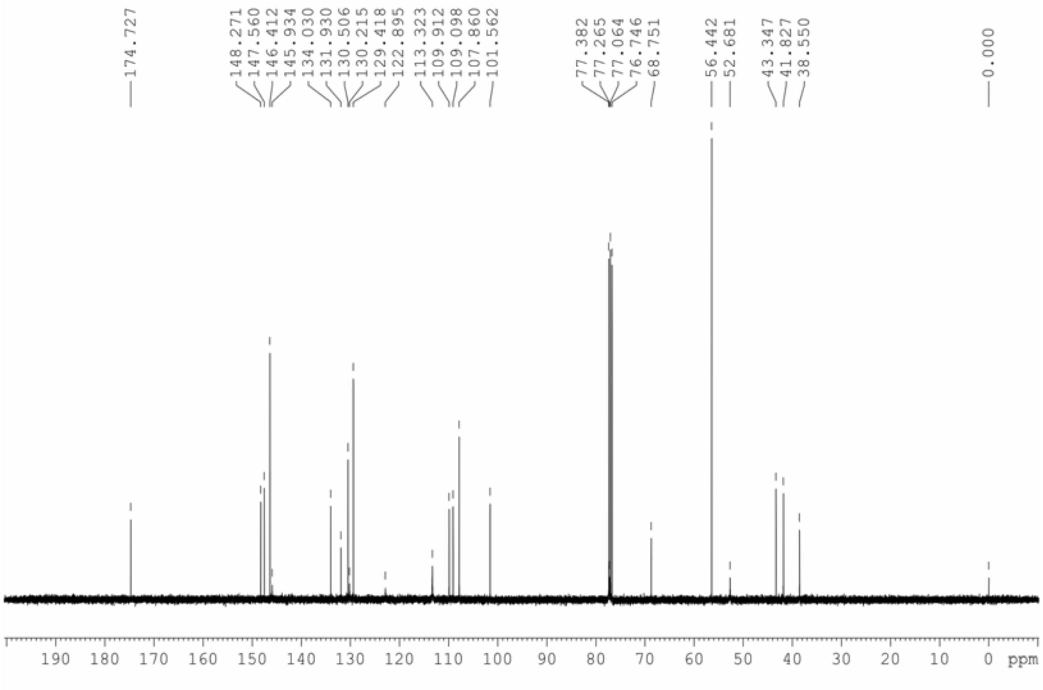

^{1}H NMR spectrum of Compound **9′**.

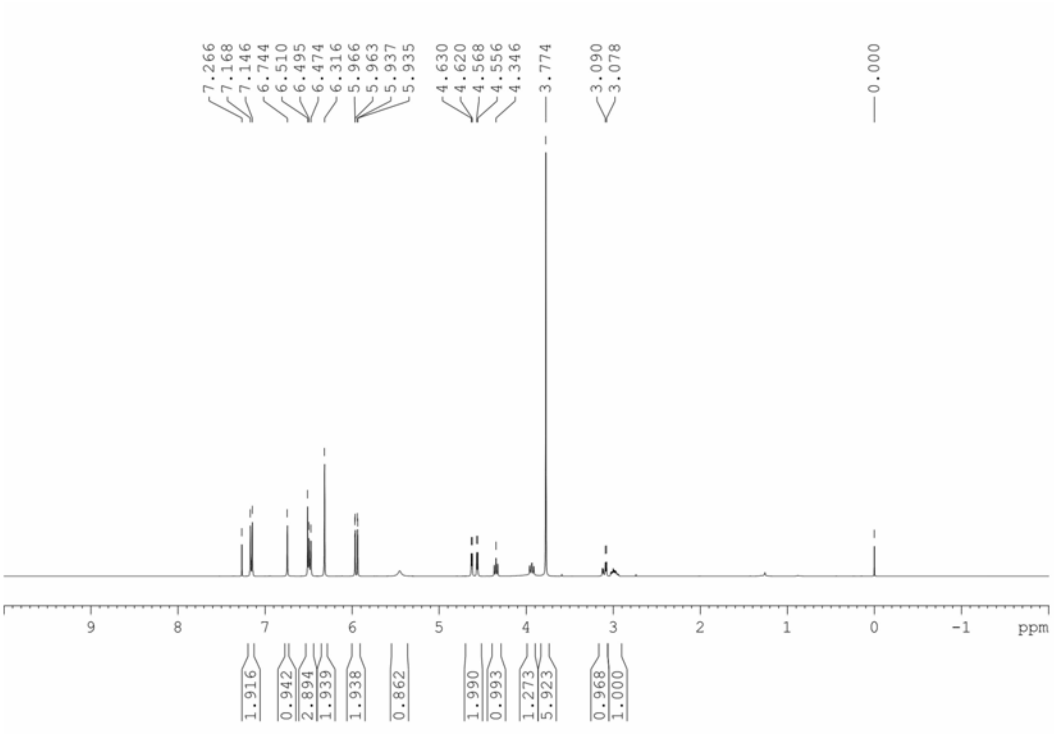

HMBC spectrums for Compound **9′**.

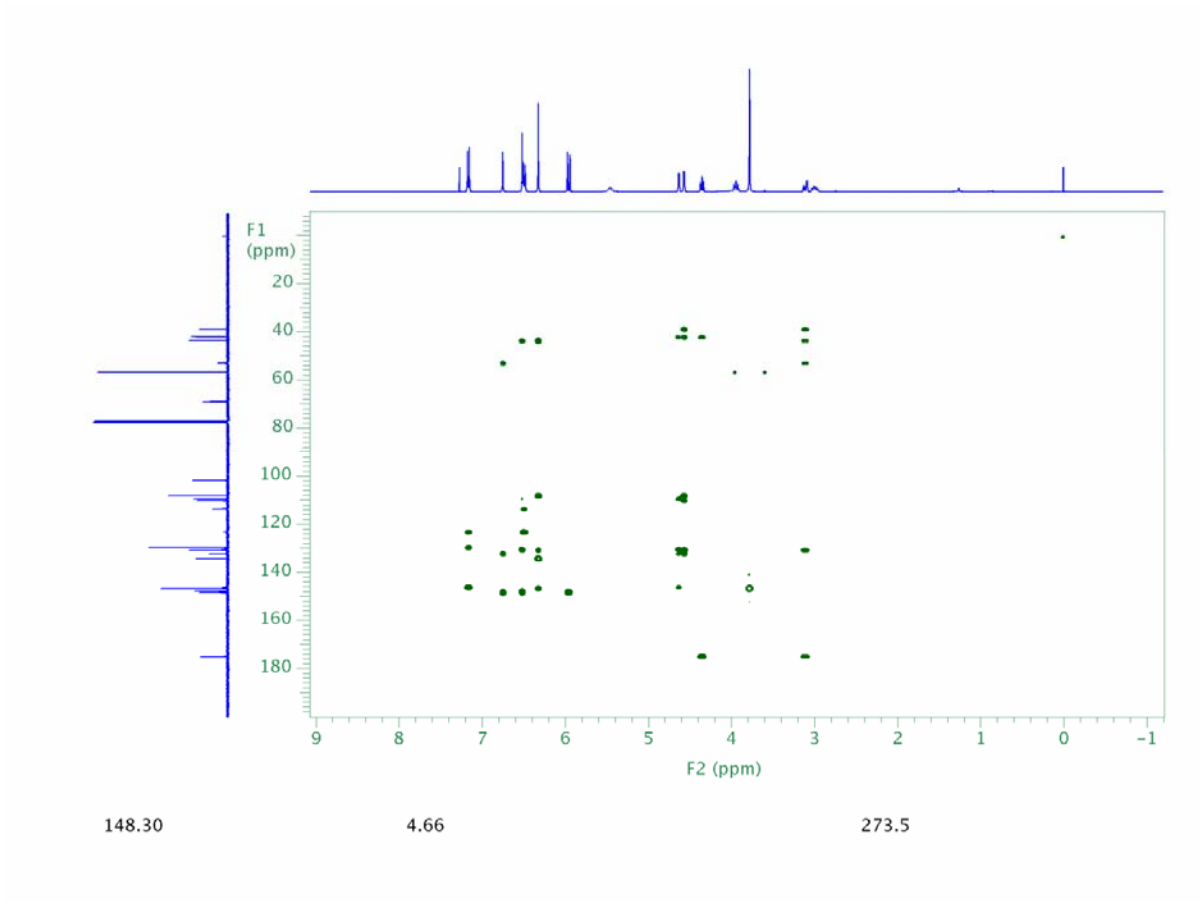

HSQC spectrums for Compound **9′**.

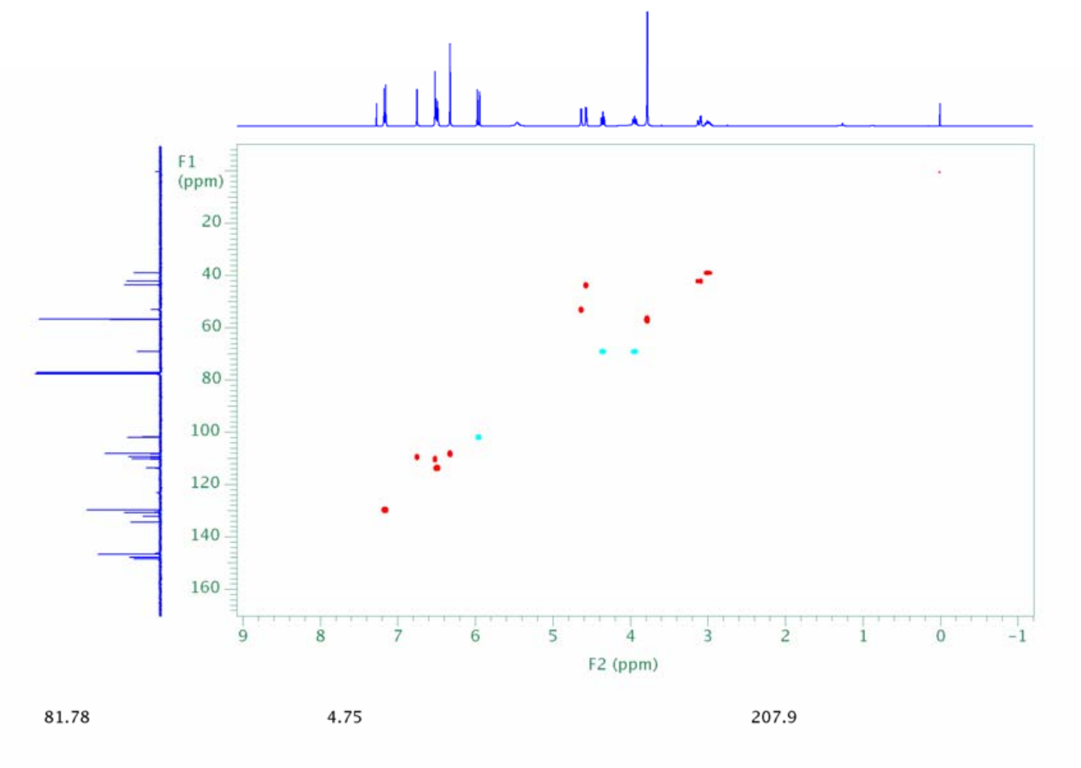

^{1}H-^{1}H COSY spectrums for Compound **9′**.

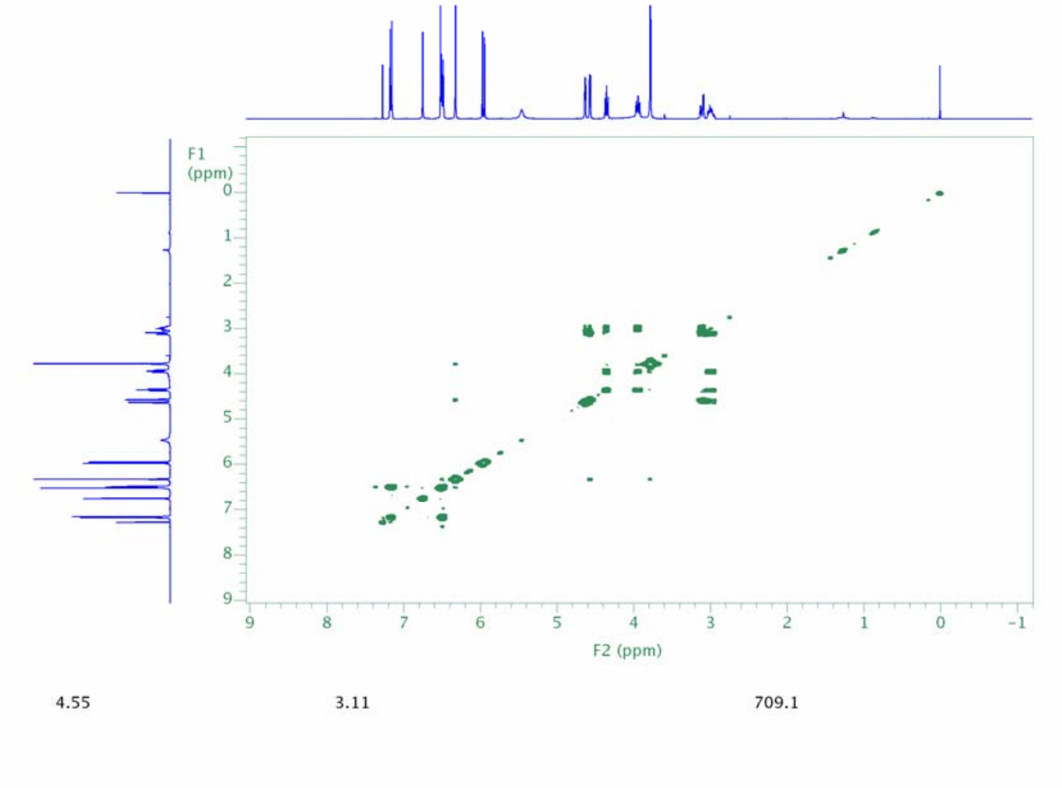

MS spectrums for Compound **9′**.

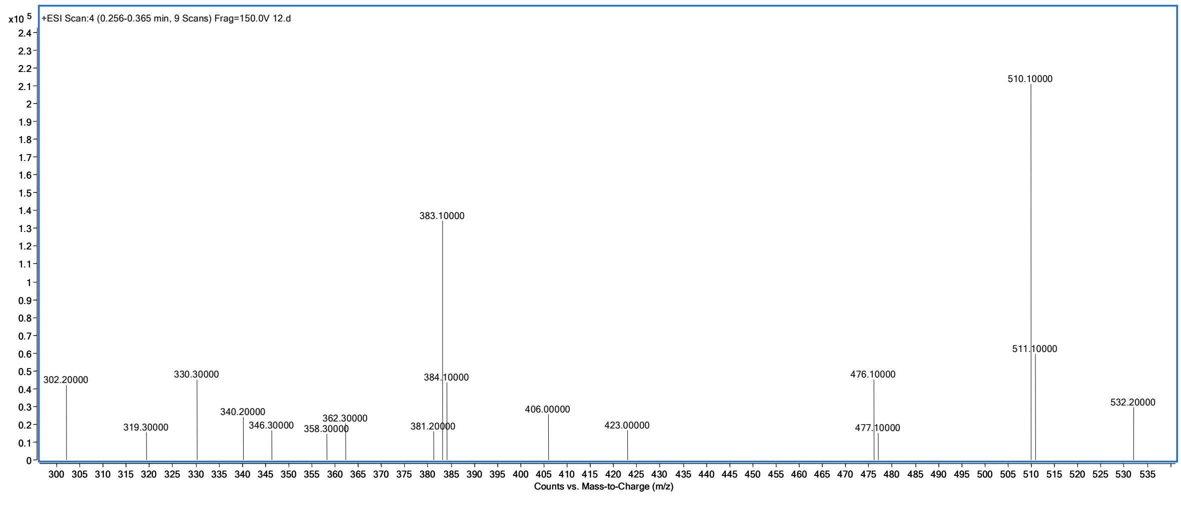

Compound 9′ (4β-*NH*-(4-chloroaniline)-4-deoxy-4′-demethylepipodophyllotoxin): 74% yield as white solid, ^{1}H NMR (400 MHz, $CDCl_3$): δ 7.15 (d, *J*=8.0 Hz, 2H, ArH), 6.74 (s, 1H, ArH), 6.51 (s, 1H, ArH), 6.49 (d, *J*=8.0 Hz, 2H, ArH), 6.32 (s, 2H, ArH), 5.94 (d, *J*=8.0 Hz, 2H, OCH_2O), 4.62 (d, *J*=4.0 Hz, 1H, 4-H), 4.56 (d, *J*=4.0 Hz, 1H, 1-H), 4.43 (t, *J*=8.0Hz, 1H, 11-H), 3.95 (t, *J*=8.0 Hz, 1H, 11-H), 3.77(s, 6H, 3’, 5’-OCH_3), 3.01 (dd, *J*=4.0 Hz, 1H, 3-H), 2.90(m, 1H, 2-H); ^{13}C NMR (100

MHz, $CDCl_3$): δ 174.72, 148.23, 147.60, 146.41, 145.93, 134.03, 131.93, 130.51, 130.21, 129.42, 122.90, 113.32, 109.91, 109.01, 107.86, 101.56, 68.75, 56.44, 52.68, 43.34, 41.82, 38.55; MS (ESI): m/z: 510 $[M+H]^+$

^{13}C NMR spectrum of 4*β*-*NH*-(3-fluoroaniline)-4-deoxy-4′-demethylepipodophyllotoxin **(10′)**.

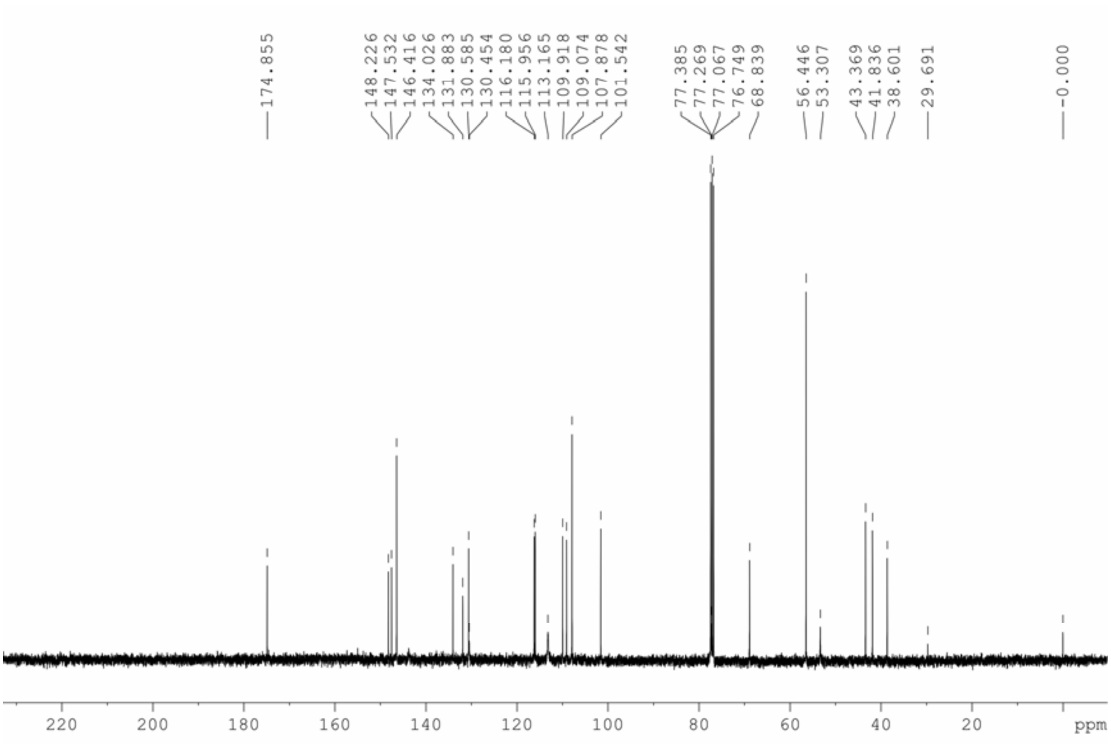

^1H NMR spectrum of Compound **10′**.

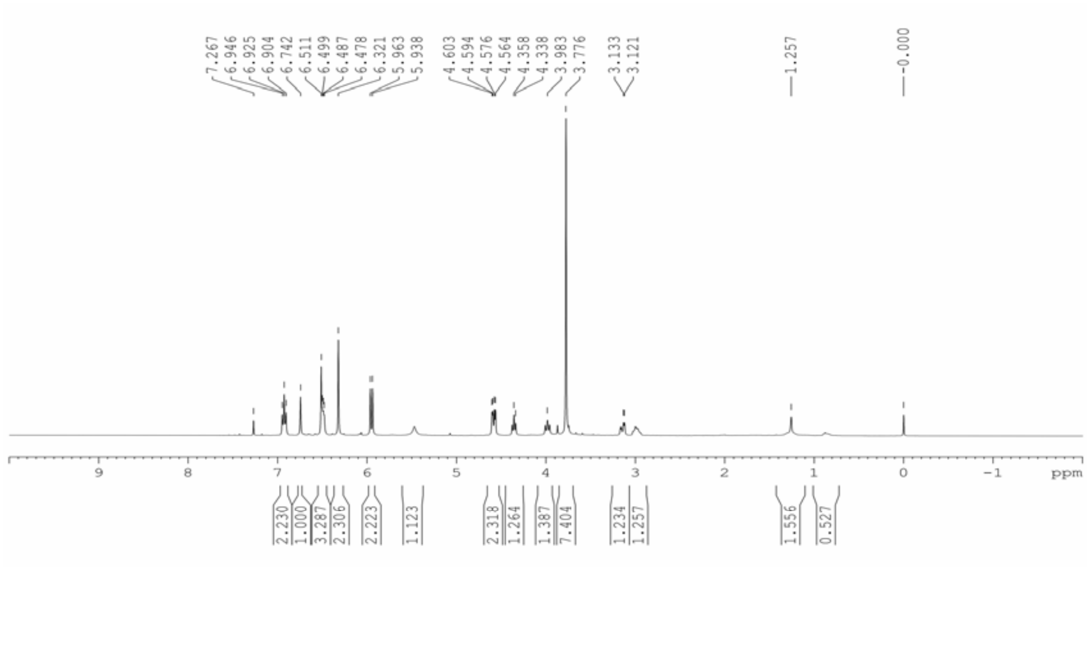

HMBC spectrums for Compound **10′**.

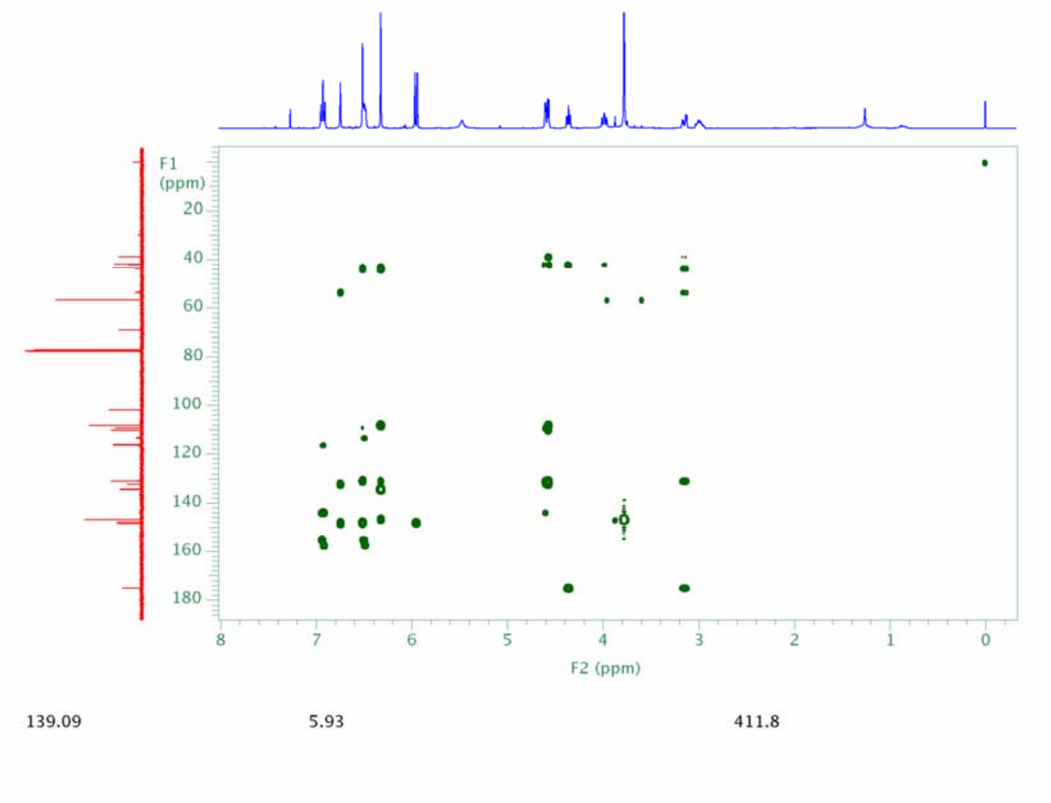

HSQC spectrums for Compound **10′**.

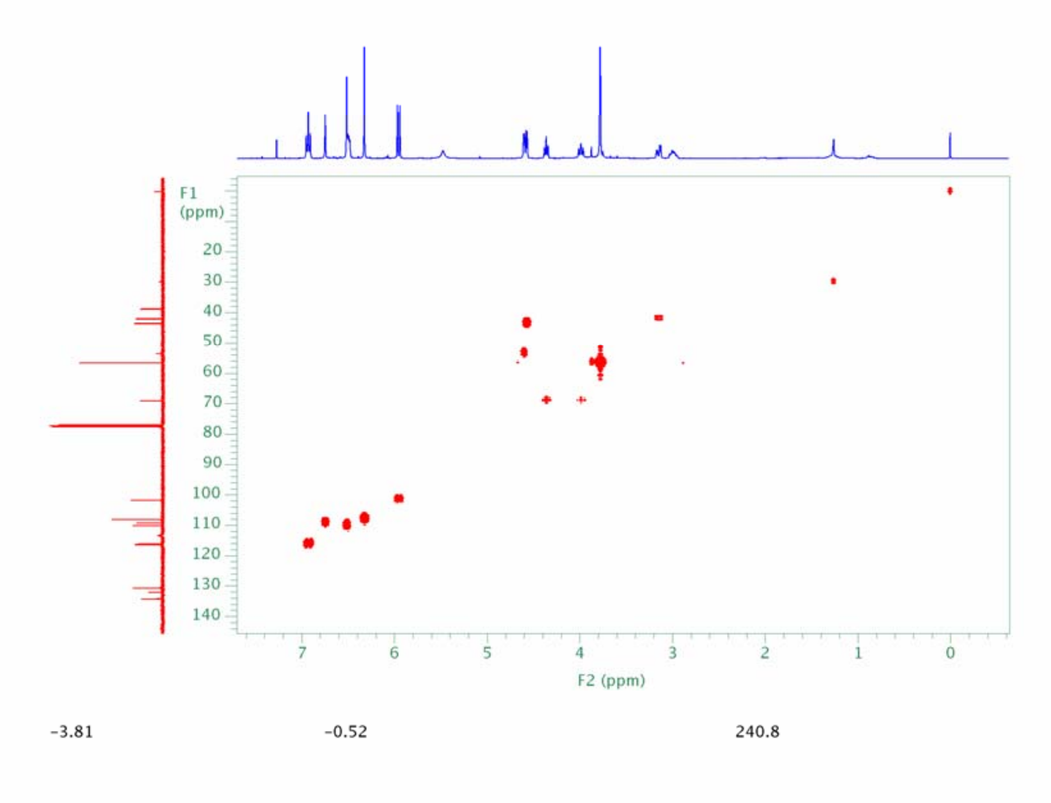

^{1}H-^{1}H COSY spectrums for Compound **10′**.

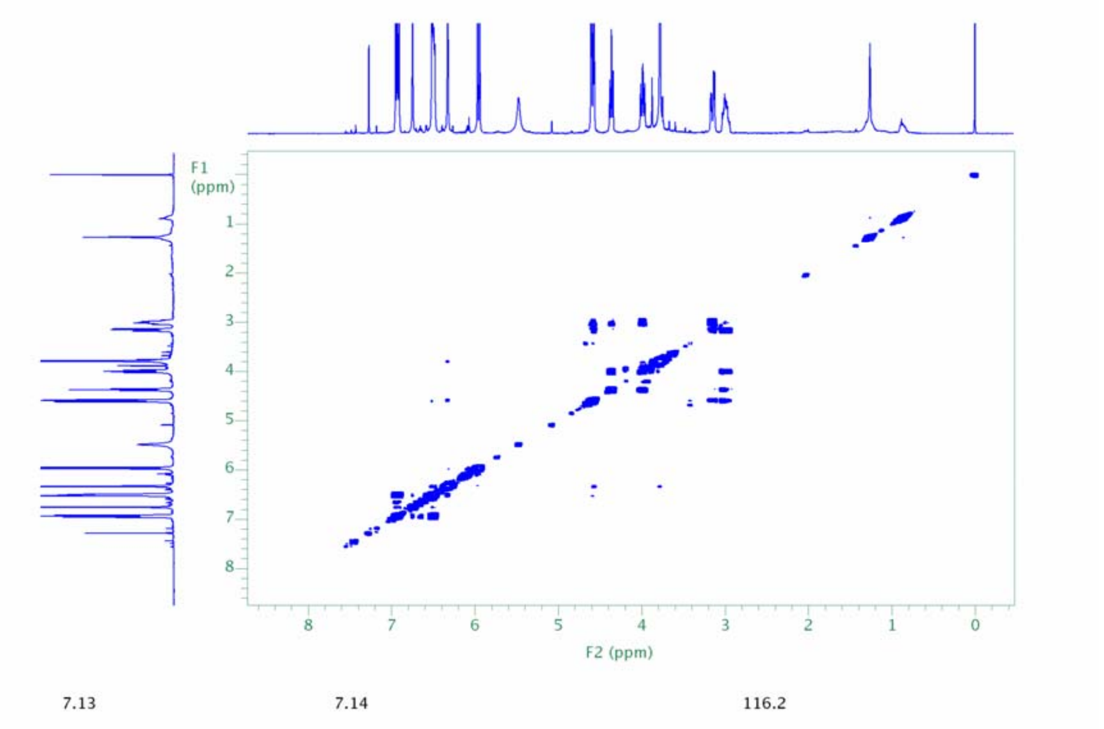

MS spectrums for Compound **10′**.

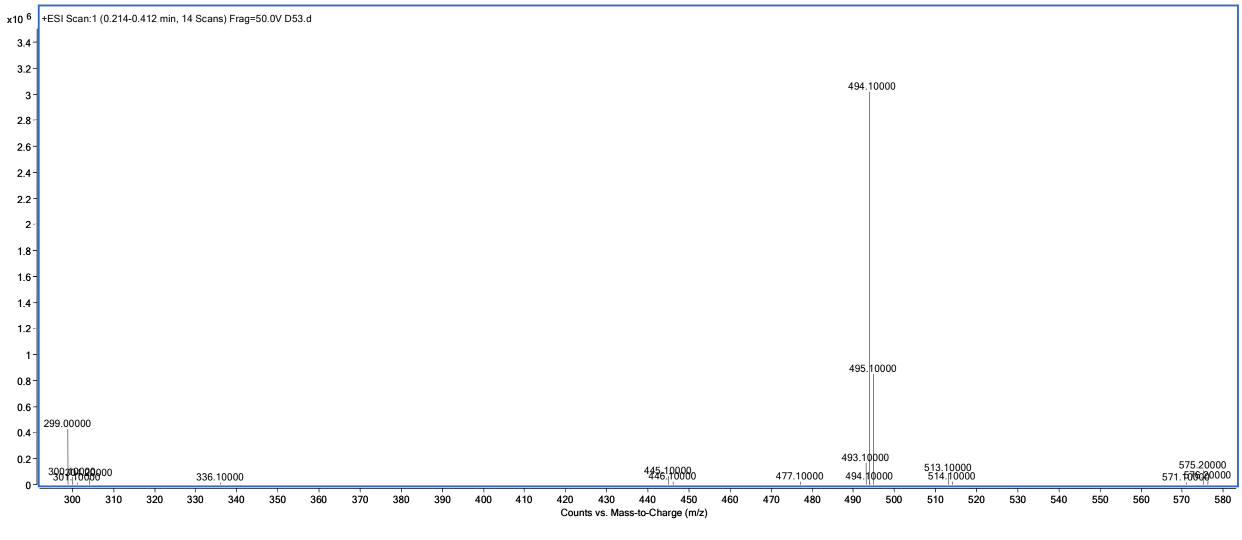

Compound 3′F (4β-N-(4-fluoroaniline)-4-deoxy-4′-demethylepipodophyllotoxin): 88% yield as white solid, ^{1}H NMR (400 MHz, $CDCl_3$): δ 6.93 (t, *J*=8.0 Hz, 2H, ArH), 6.74 (s, 1H, ArH), 6.51 (s, 1H, ArH), 6.48 (t, *J*=4.0 Hz, 2H, ArH), 6.32 (s, 2H, ArH), 5.94 (d, *J*=8.0 Hz, 2H, OCH_2O), 4.56 (dd, *J*=4.0 Hz, 2H, 1-H, 4-H), 4.36 (t, *J*=8.0 Hz, 1H, 11-H), 3.98 (t, *J*=8.0 Hz, 1H, 11-H), 3.78 (s, 6H, 3’, 5’-OCH_3), 3.12 (dd, *J*=4.0 Hz, 1H, 3-H), 2.99 (m, 1H, 2-H); ^{13}C NMR (100 MHz, $CDCl_3$): δ 174.86,

148.23, 147.53, 146.41, 145.93, 134.03, 131.93, 130.51, 130.21, 116.18, 115.96, 113.17, 109.92, 109.01, 107.88, 101.54, 68.84, 56.45, 53.31, 43.37, 41.84, 38.60; MS (ESI): m/z: 494 $[M+H]^+$

^{13}C NMR spectrum of 4*β*-*NH*-(1-anisidine)-4-deoxy-4′-demethylepipodophyllotoxin **(11′)**.

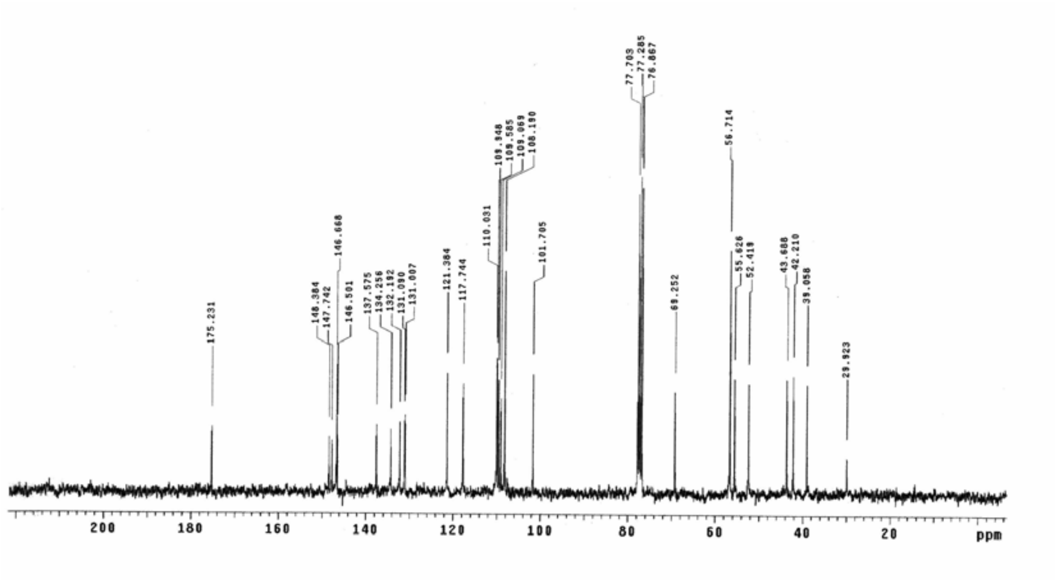

^{1}H NMR spectrum of Compound **11′**.

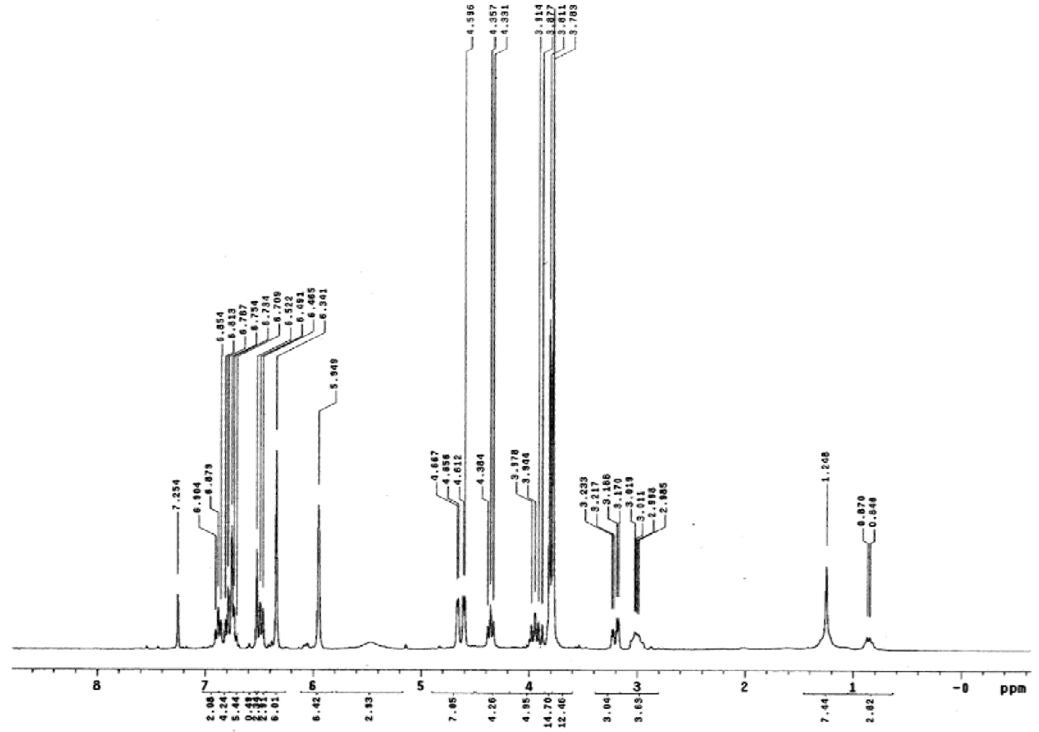

HMBC spectrums for Compound **11′**.

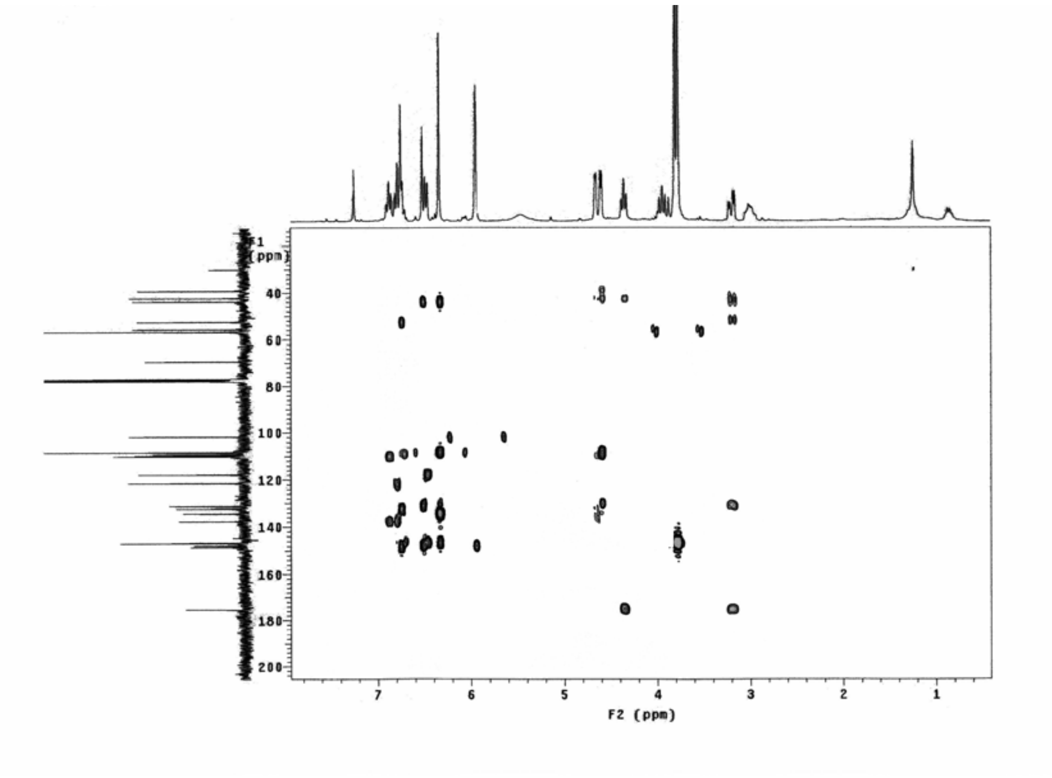

HSQC spectrums for Compound **11′**.

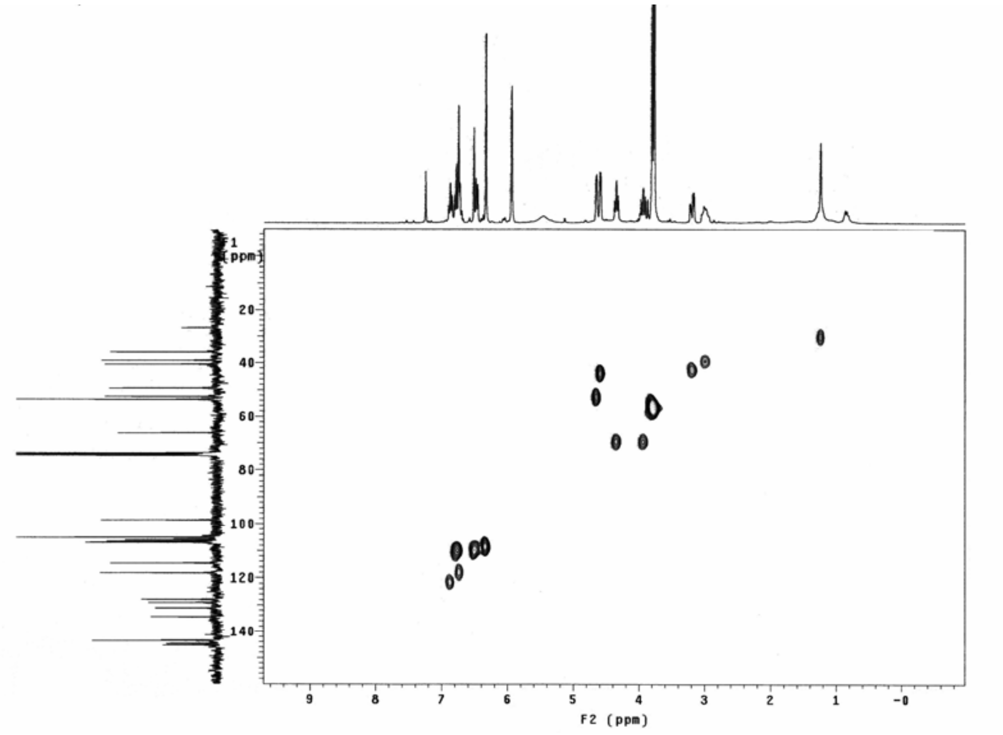

^{1}H-^{1}H COSY spectrums for Compound **11′**.

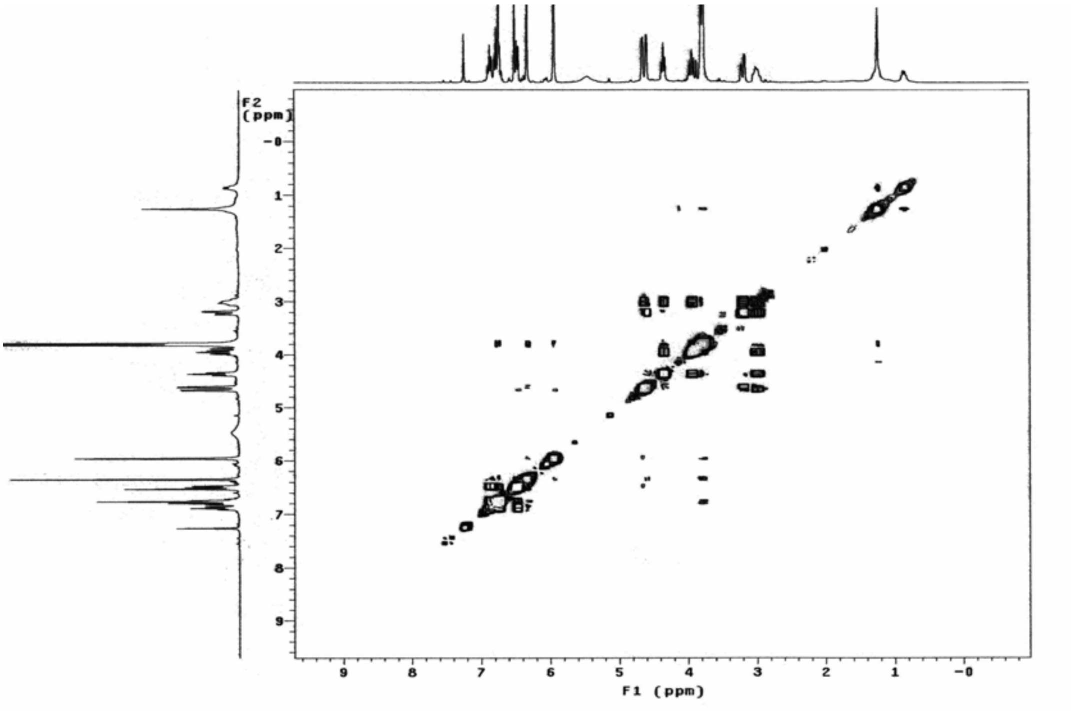

MS spectrums for Compound **11′**.

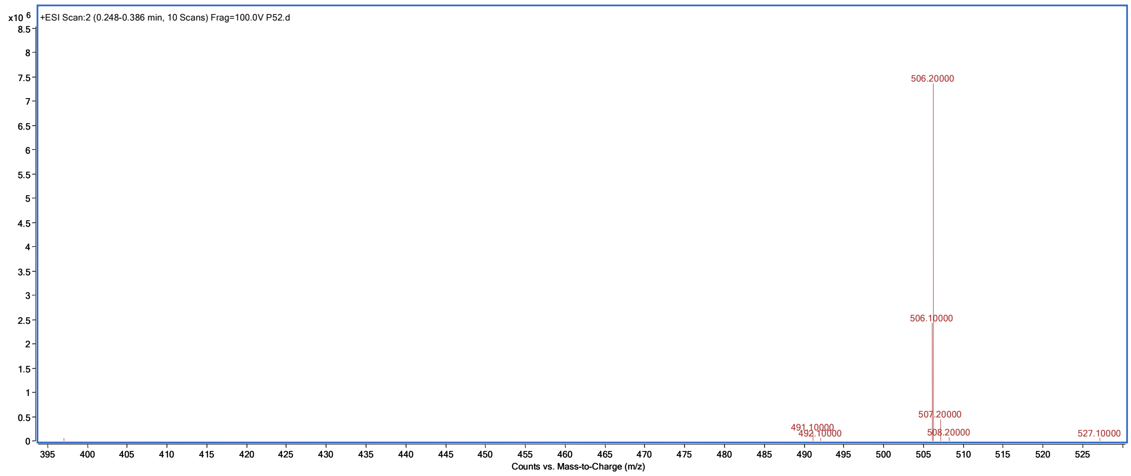

Compound 11′ (4β-N-(1-anisidine)-4-deoxy-4′-demethylepipodophyllotoxin): 52% yield as white solid, ^{1}H NMR (300 MHz, CDCl3): δ 2.985-3.019(m, 1H, 2-H) , 3.170 (dd, *J*=4.8Hz, 1H, 3-H), 3.783(s, 6H, 3′, 5′-OCH_3), 3.811 (s, 3H, Ar-OCH_3) , 3.944 (t, *J*=9.3 Hz, 1H, 11-H), 4.357 (t, *J*=7.8Hz, 1H, 11-H), 4.596 (d, *J*=1.8 Hz, 1H, 4-H), 4.656 (d, *J*=3.3 Hz, 1H, 1-H), 5.949 (s, 2H, OCH_2O), 6.341 (s, 2H, ArH), 6.465 (d, *J*=7.8 Hz, 1H, ArH) , 6.522 (s, 1H, ArH), 6.709-6.813 (m, 3H, ArH) , 6.879 (t, *J*=7.5 Hz, 1H, ArH) ^{13}C NMR (75 MHz, $CDCl_3$): δ 39.058, 42.210, 43.688,

52.419, 55.626, 56.714, 69.252, 101.705, 108.190, 109.069, 109.585, 109.948, 110.031, 117.744, 121.384, 131.007, 131.090, 132.192, 134.256, 137.575, 146.501, 146.668, 147.742, 148.384, 175.231; MS (ESI): m/z: 506 $[M+H]^+$

^{13}C NMR spectrum of 4*β*-*NH*-(2-anisidine)-4-deoxy-4′-demethylepipodophyllotoxin **(12′)**.

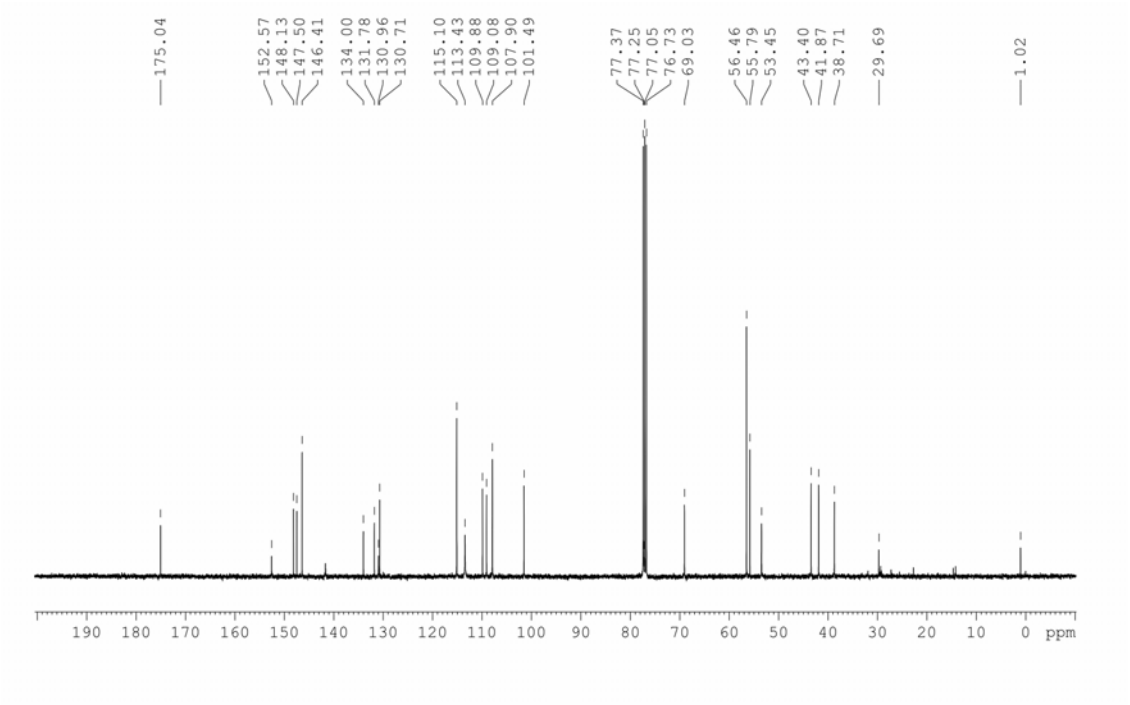

^{1}H NMR spectrum of Compound **12′**.

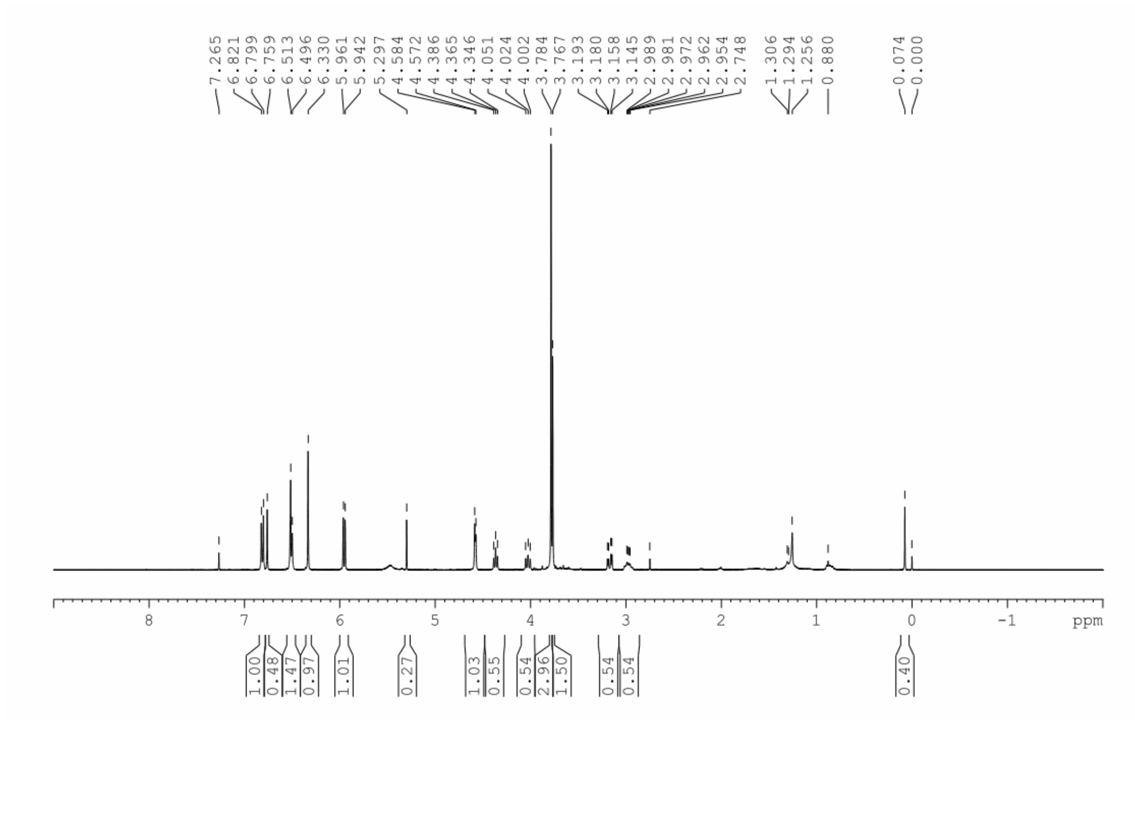

HMBC spectrums for Compound **12′**.

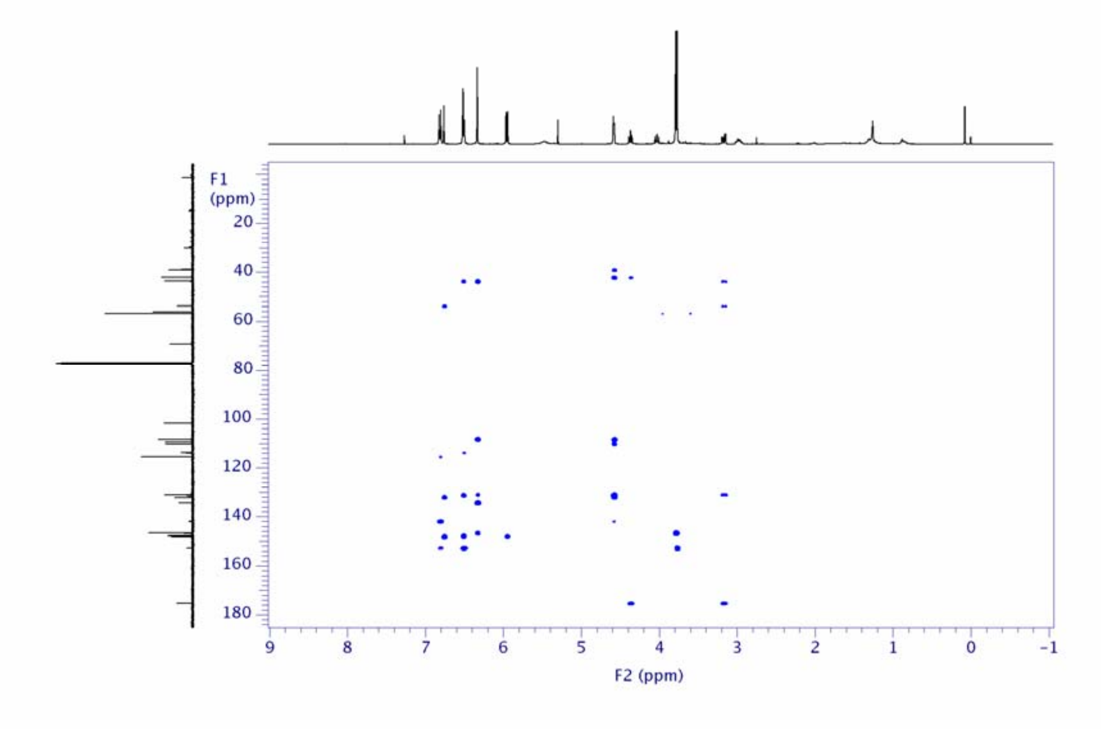

HSQC spectrums for Compound **12′**.

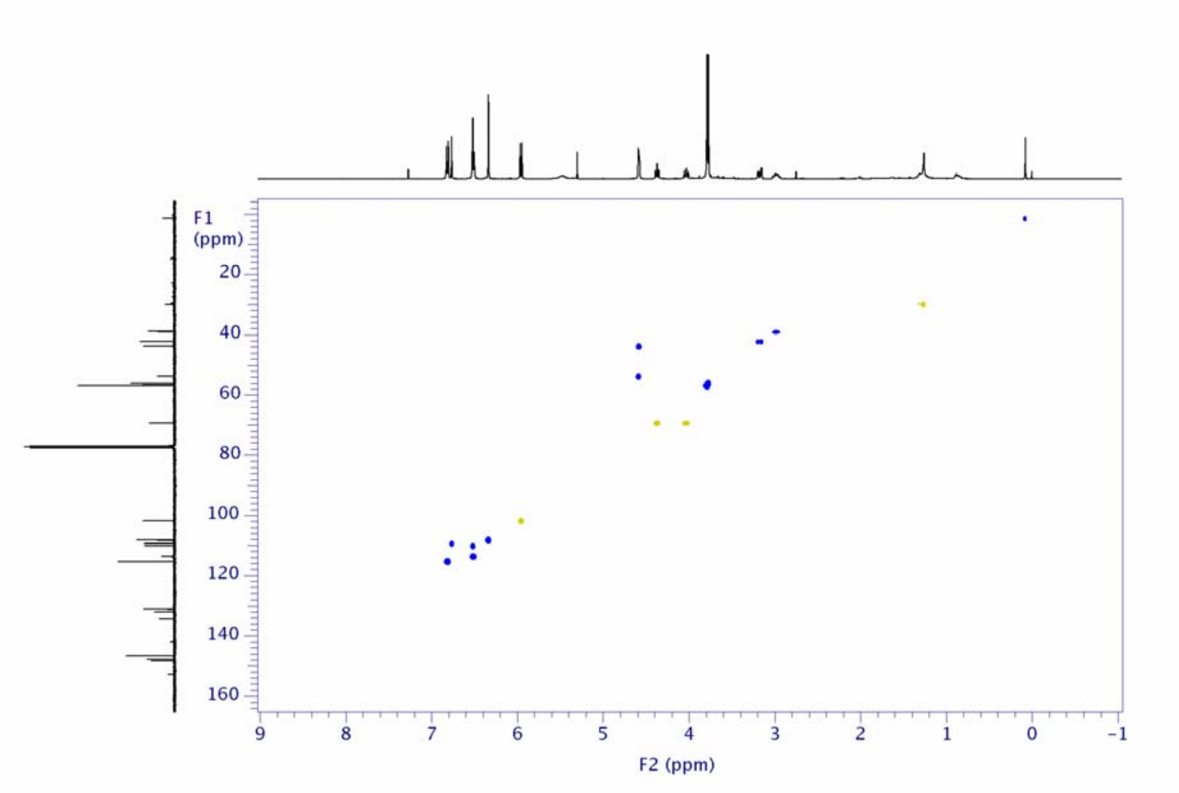

^{1}H-^{1}H COSY spectrums for Compound **12′**.

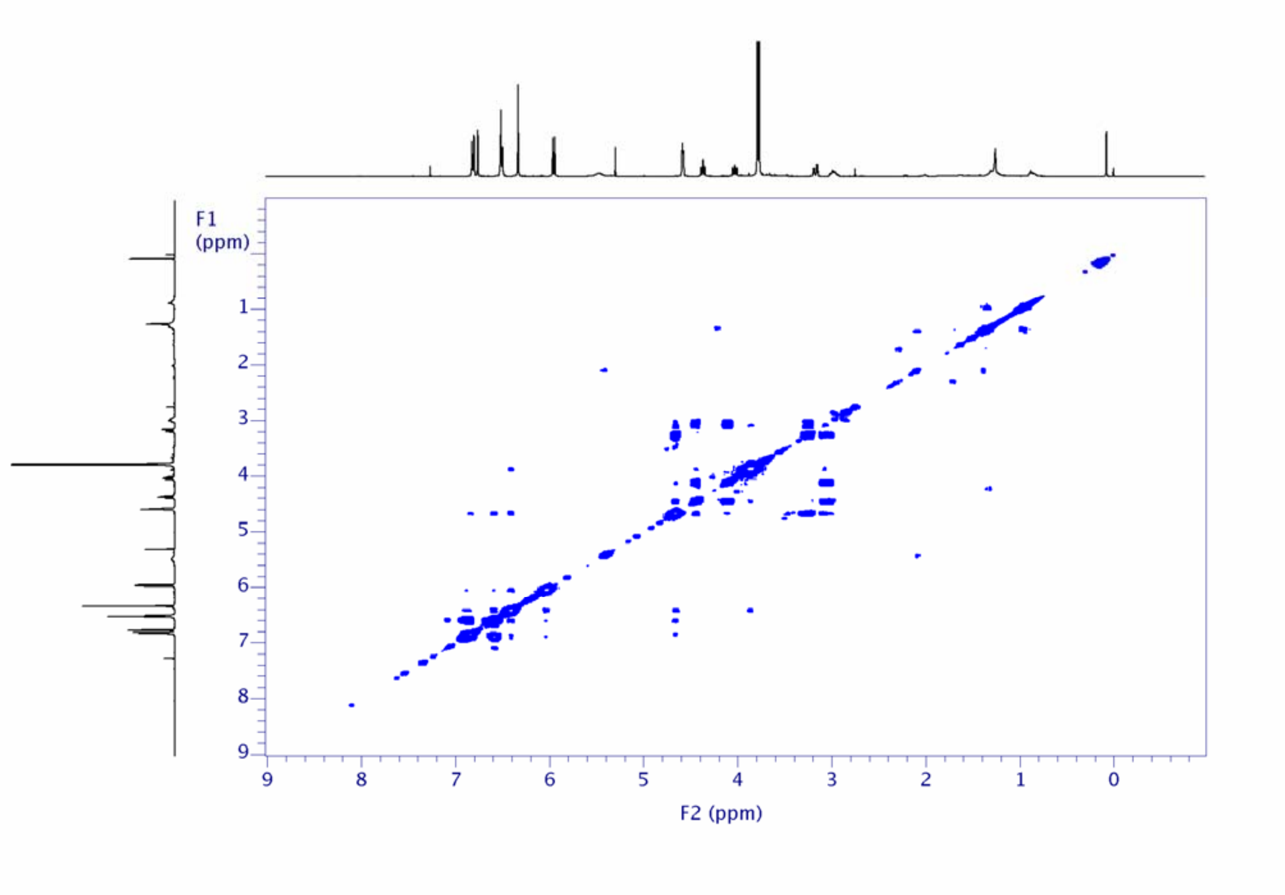

MS spectrums for Compound **12′**.

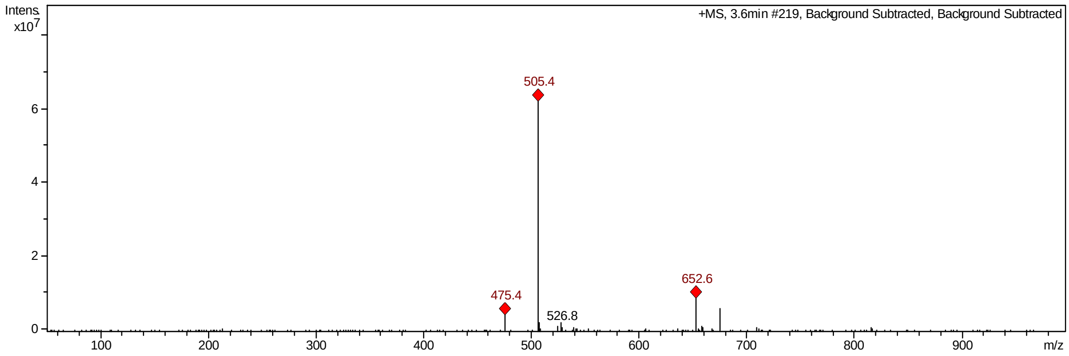

Compound 12′ (4β-*NH*-(4-anisidine)-4-deoxy-4′-demethylepipodophyllotoxin): 64% yield as white solid, ^{1}H NMR (400 MHz, $CDCl_3$): δ 2.954-2.989 (m, 1H, 2-H) , 3.145 (dd, *J*=5.2 Hz, 1H, 3-H), 3.767 (s, 3H, Ar-OCH_3), 3.784 (s, 6H, 3′, 5′-OCH_3), 3.997 (t, *J*=9.8 Hz, 1H, 11-H), 4.365 (t, *J*=8.0 Hz, 1H, 11-H), 4.572 (d, *J*=4.8 Hz, 2H,1-H, 4-H), 5.942 (d, *J*=7.6 Hz, 2H, OCH_2O), 6.330 (s, 2H, ArH), 6.496 (s, 1H, ArH) , 6.513 (s, 2H, ArH), 6.759 (s,1H, ArH) , 6.799 (s, 1H, ArH), 6.821 (s,

1H, ArH) ^{13}C NMR (100 MHz, $CDCl_3$): δ 38.71, 41.87, 43.40, 53.45, 55.79, 56.46, 69.03, 76.73, 77.05, 77.25, 77.37, 101.49, 107.90, 109.88, 113.43, 115.10, 130.71, 130.96, 131.78, 134.00, 146.41, 147.50, 148.13, 152.57, 175.04; MS (ESI): m/z: 506 $[M+H]^+$

^{13}C NMR spectrum of 4*β*-*NH*-(3-anisidine)-4-deoxy-4′-demethylepipodophyllotoxin **(13′)**.

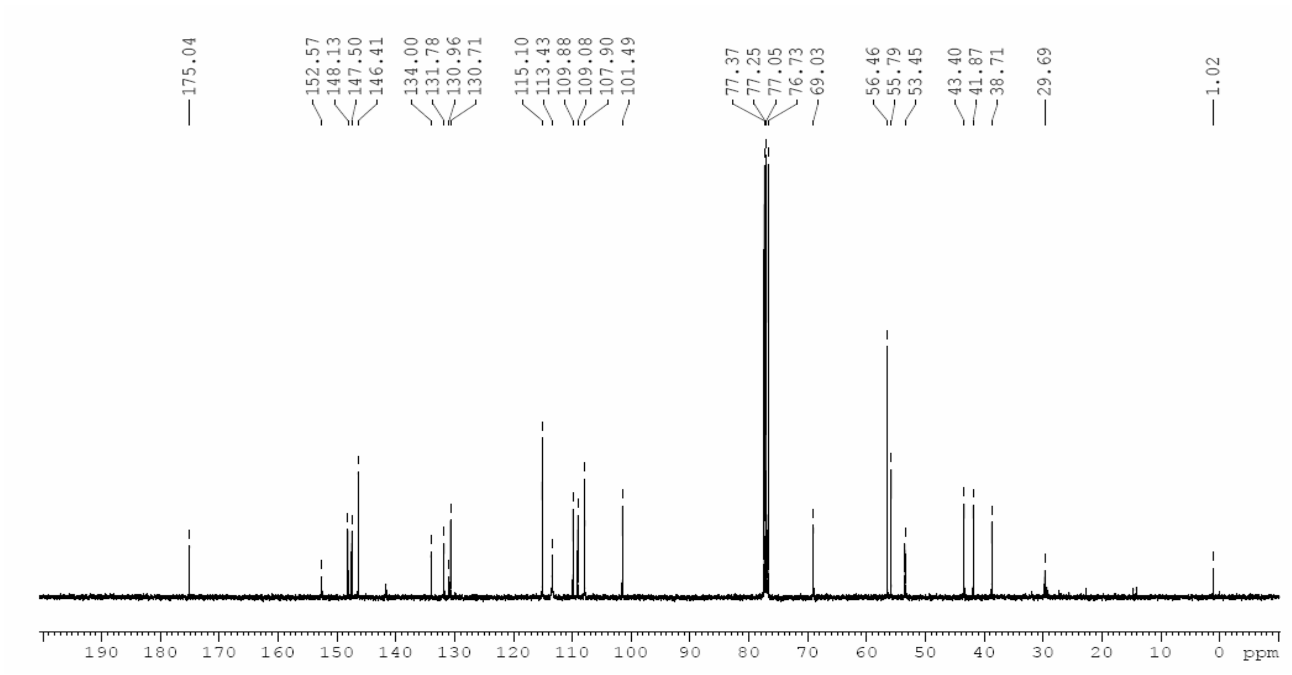

^{1}H NMR spectrum of Compound **12′**.

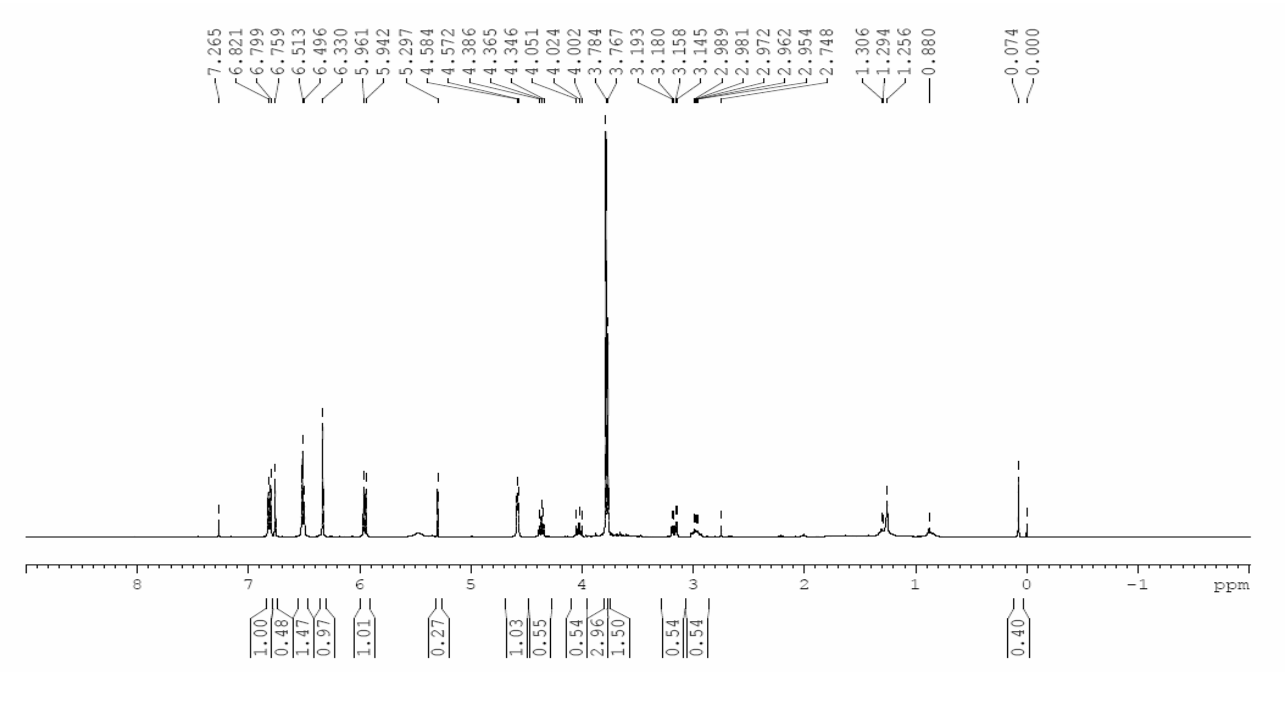

MS spectrums for Compound **13′**.

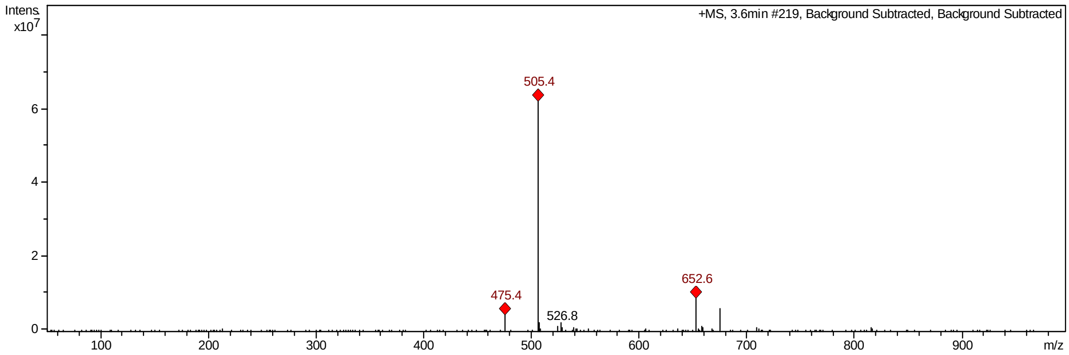

Compound 13′ (4β-N-(4-anisidine)-4-deoxy-4′-demethylepipodophyllotoxin): 64% yield as white solid, ^{1}H NMR (400 MHz, $CDCl_3$): δ 2.95 (m, 1H, 2-H) , 3.15 (dd, *J*=5.2 Hz, 1H, 3-H), 3.77 (s, 3H, Ar-OCH_3), 3.78 (s, 6H, 3’, 5’-OCH_3), 4.00 (t, *J*=9.8 Hz, 1H, 11-H), 4.37 (t, *J*=8.0 Hz, 1H, 11-H), 4.57 (d, *J*=4.8 Hz, 2H,1-H, 4-H), 5.94 (d, *J*=7.6 Hz, 2H, OCH_2O), 6.33 (s, 2H, ArH), 6.50 (s, 1H, ArH) , 6.51 (s, 2H, ArH), 6.76 (s,1H, ArH) , 6.80 (s, 1H, ArH), 6.82 (s, 1H, ArH) ^{13}C NMR (100 MHz, $CDCl_3$): δ 38.71, 41.87, 43.40, 53.45, 55.79, 56.46, 69.03, 76.73, 77.05, 77.25, 77.37, 101.49, 107.90, 109.88, 113.43, 115.10, 130.71, 130.96, 131.78, 134.00, 146.41, 147.50, 148.13, 152.57, 175.04; MS (ESI): m/z: 506 $[M+H]^+$

^{13}C NMR spectrum of 4*β*-*NH*-(5-bromo-2-methoxyaniline)-4-deoxy-podophyllotoxin **(14)**.

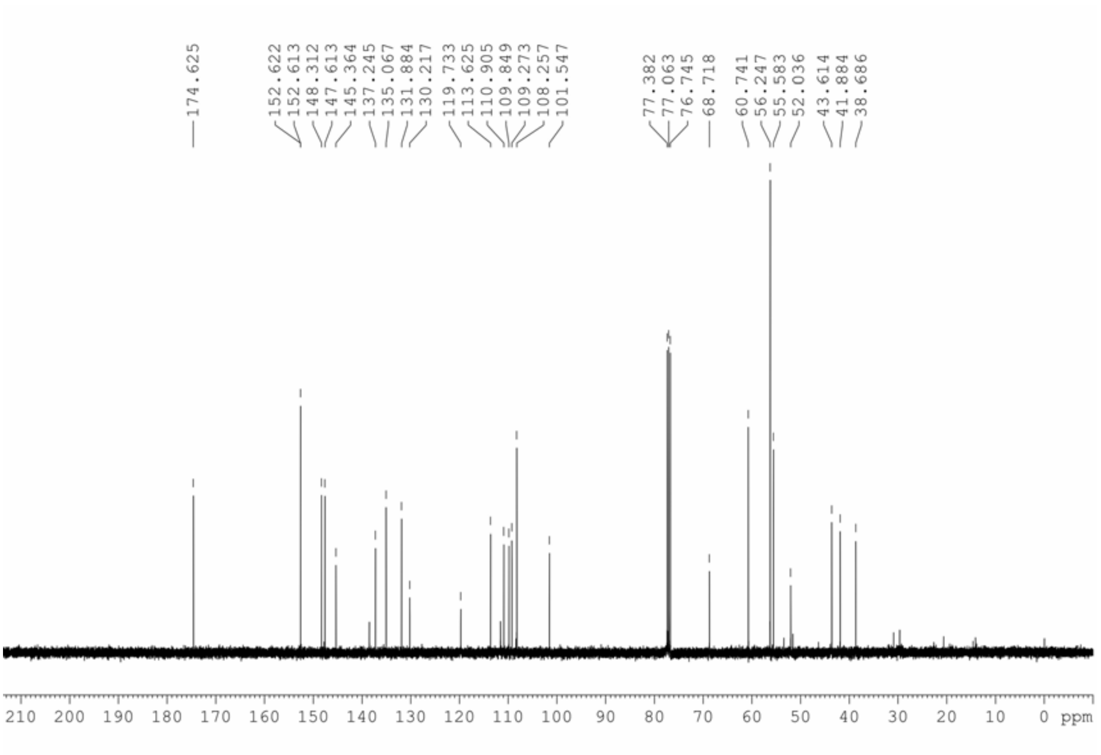

^{1}H NMR spectrum of Compound **14**.

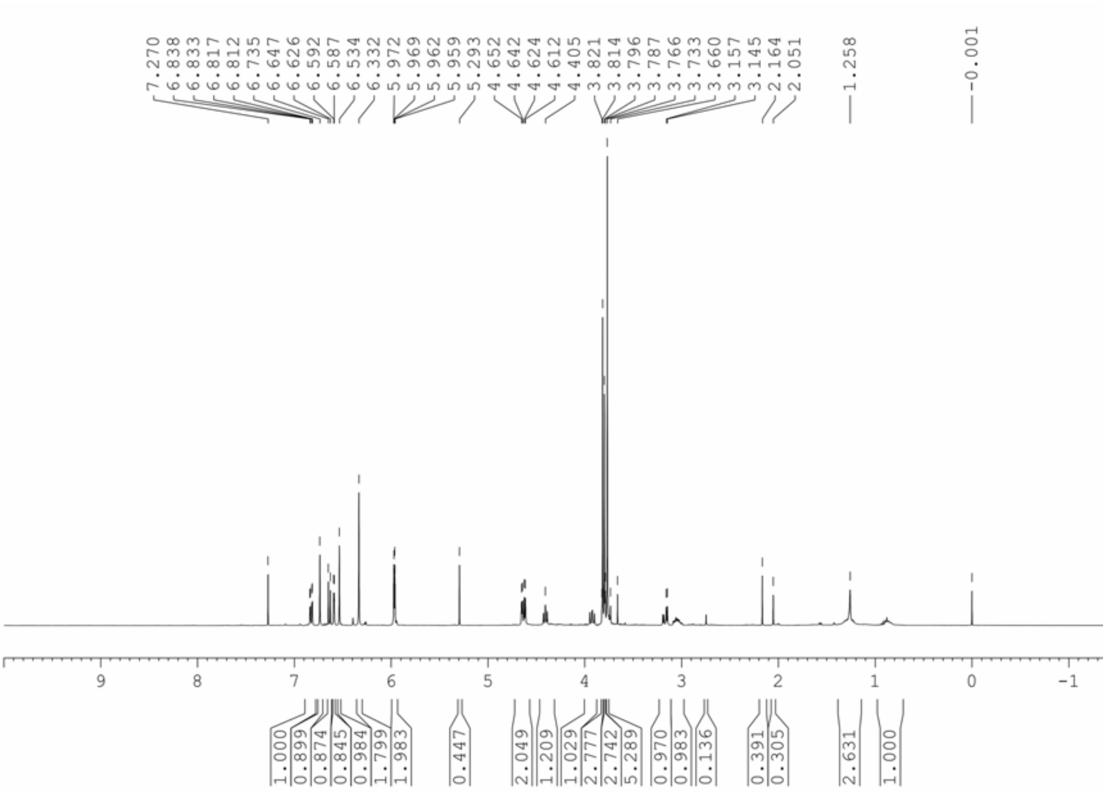

HMBC spectrums for Compound **14**.

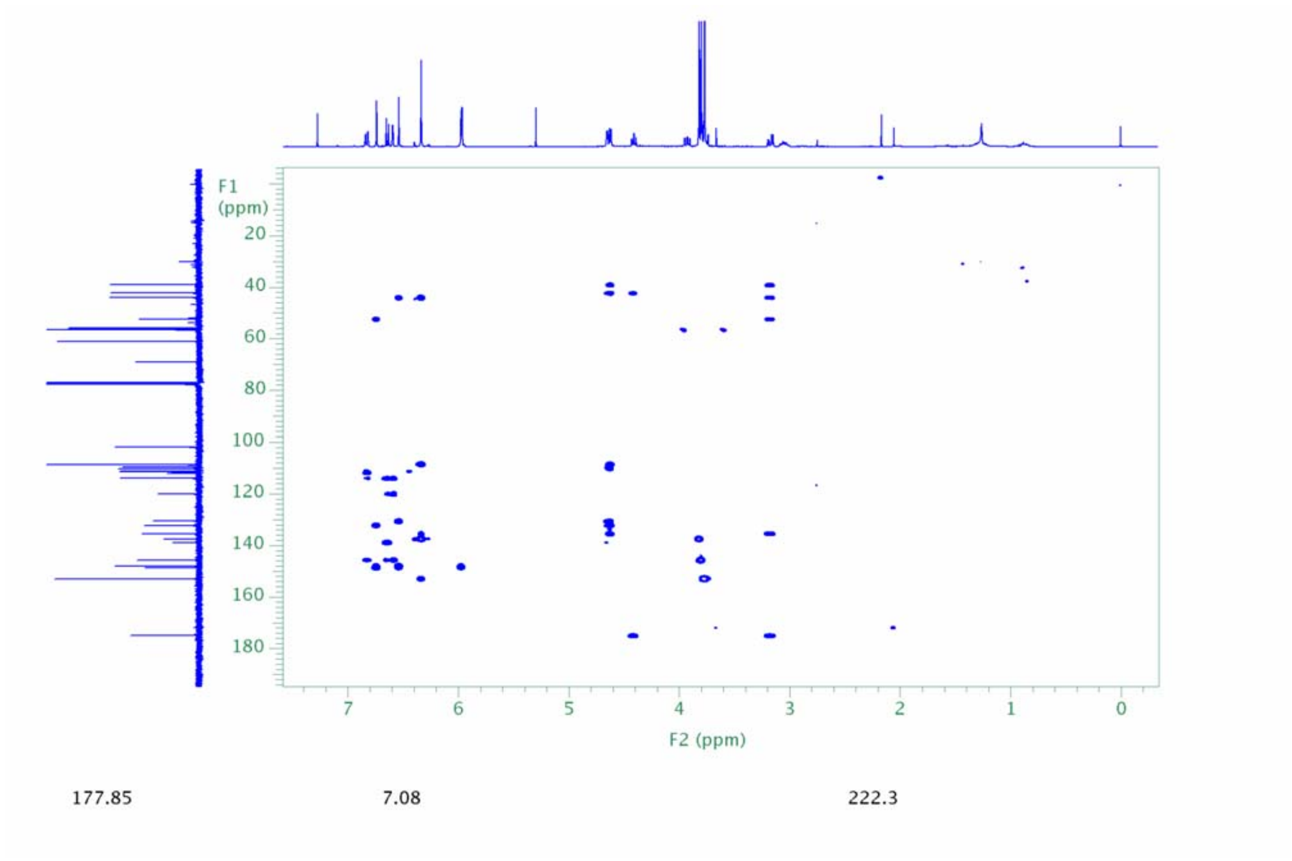

HSQC spectrums for Compound **14**.

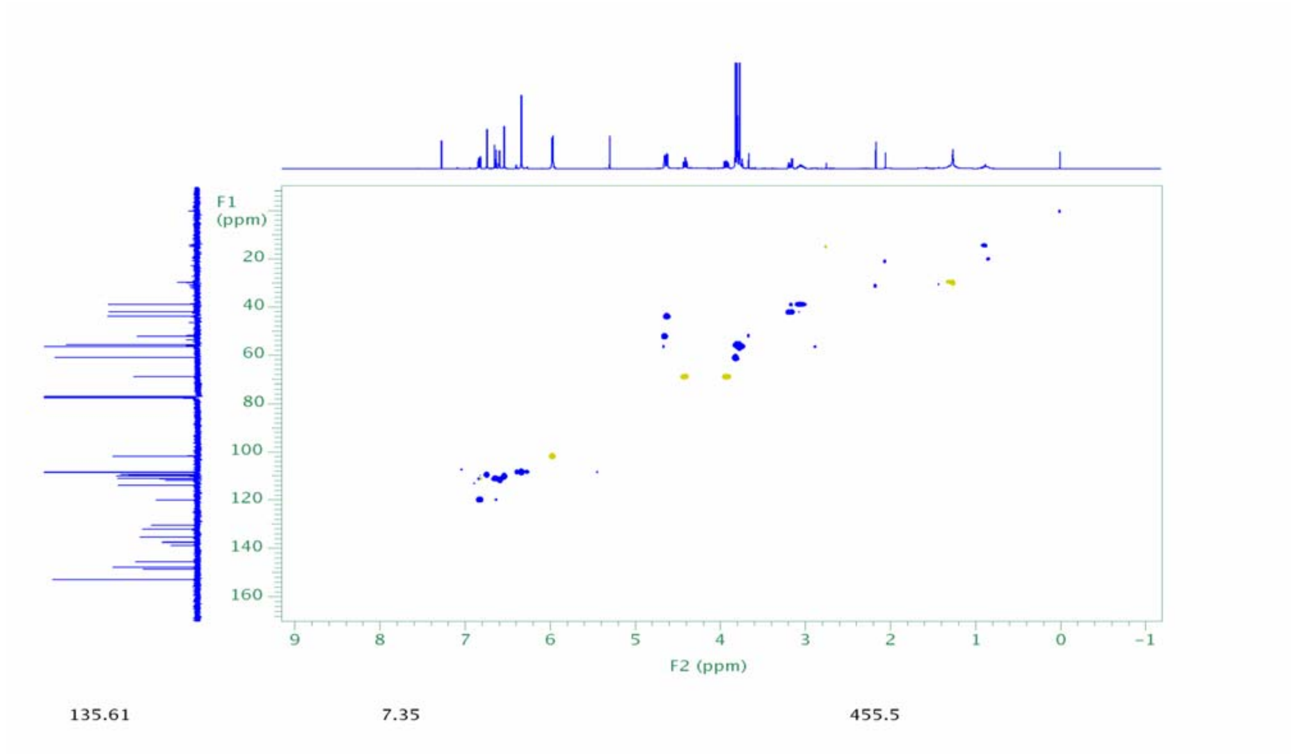

^{1}H-^{1}H COSY spectrums for Compound **14**.

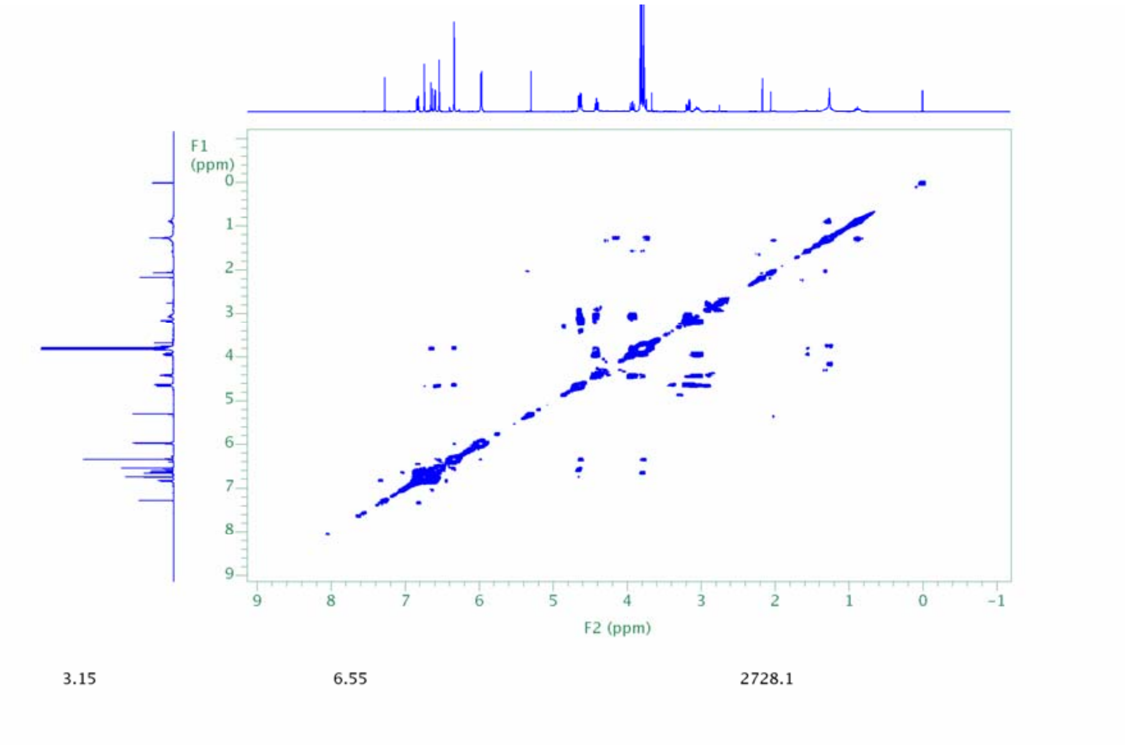

MS spectrums for Compound **14**.

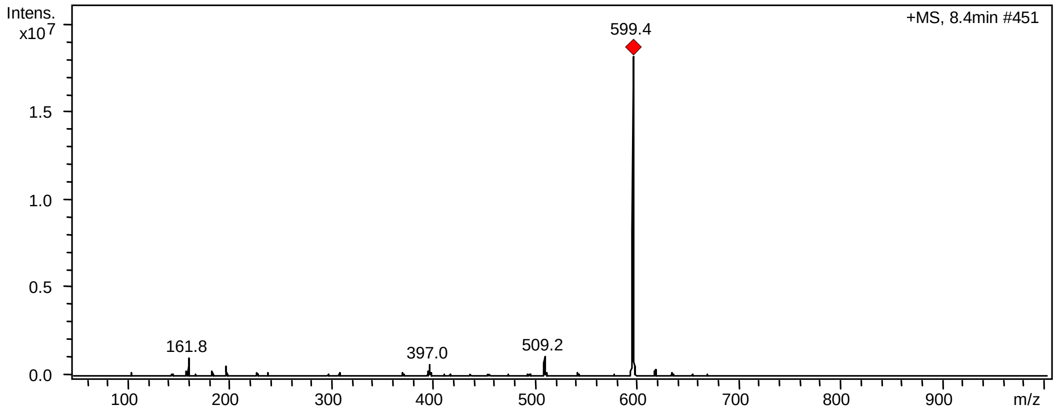

Compound 14 (4β-*NH*-(5-bromo-2-methoxyaniline)-4-deoxy-podophyllotoxin): 51% yield as white solid, ^{1}H NMR (400 MHz, $CDCl_3$): δ 2.97 (m, 1H, 2-H) , 3.12 (dd, *J*=4.0Hz, 1H, 3-H), 3.75 (s, 6H, 3′, 5′-OCH_3), 3.78 (s, 3H, Ar-OCH_3) , 3.81 (s, 3H, 4′-OCH_3) , 3.97 (t, *J*=10.0 Hz, 1H, 11-H), 4.38 (t, *J*=8.0Hz, 1H, 11-H), 4.54 (d, *J*=8.0 Hz, 1H, 4-H), 4.59 (d, *J*=4.0 Hz, 1H, 1-H), 5.91 (d, *J*=16.0 Hz, 2H, OCH_2O), 6.31 (s, 2H, ArH), 6.47 (d, *J*=4.0 Hz, 1H, ArH), 6.49 (s, 1H, ArH), 6.75 (s, 1H, ArH), 6.80 (s, 1H, ArH), 7.31 (s, 1H, ArH) ^{13}C NMR (100 MHz, $CDCl_3$): δ 38.63, 41.68,

43.50, 52.97, 56.29, 58.16, 60.67, 68.84, 101.50, 108.12, 109.11, 109.79, 111.92, 112.83, 114.02, 117.20, 130.52, 131.57, 135.20, 136.98, 142.66, 147.49, 148.10, 148.61, 152.50, 174.93; MS (ESI): m/z: 599 $[M+H]^+$

^{13}C NMR spectrum of 4*β*-*NH*-(5-chloro-2-methoxyaniline)-4-deoxy-podophyllotoxin **(15)**.

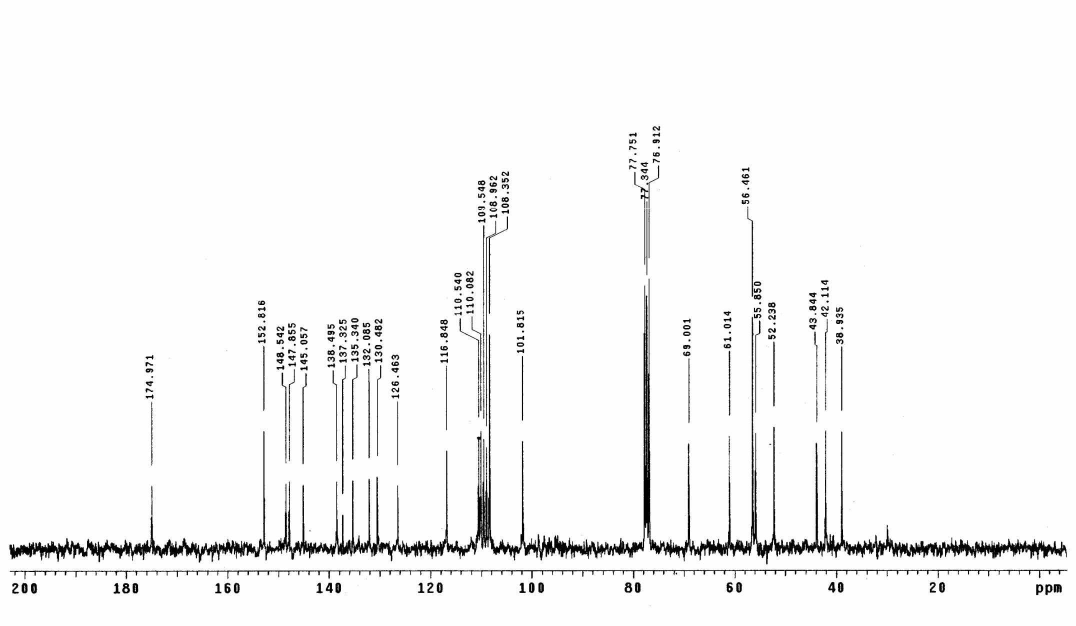

^{1}H NMR spectrum of Compound **15**.

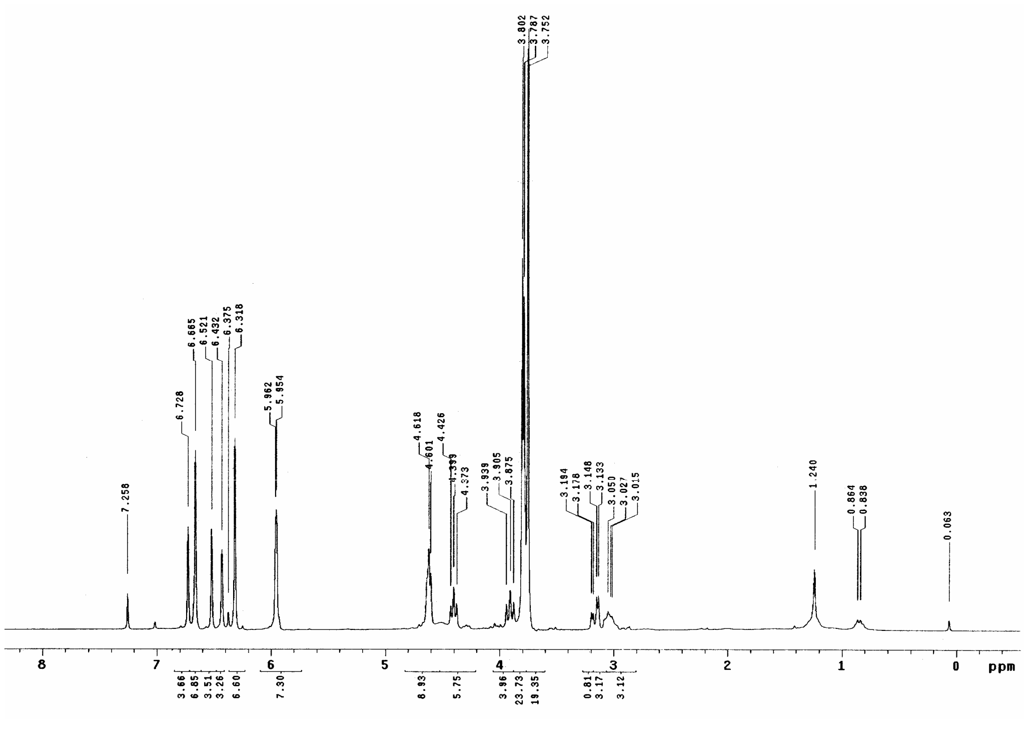

HMBC spectrums for Compound **15**.

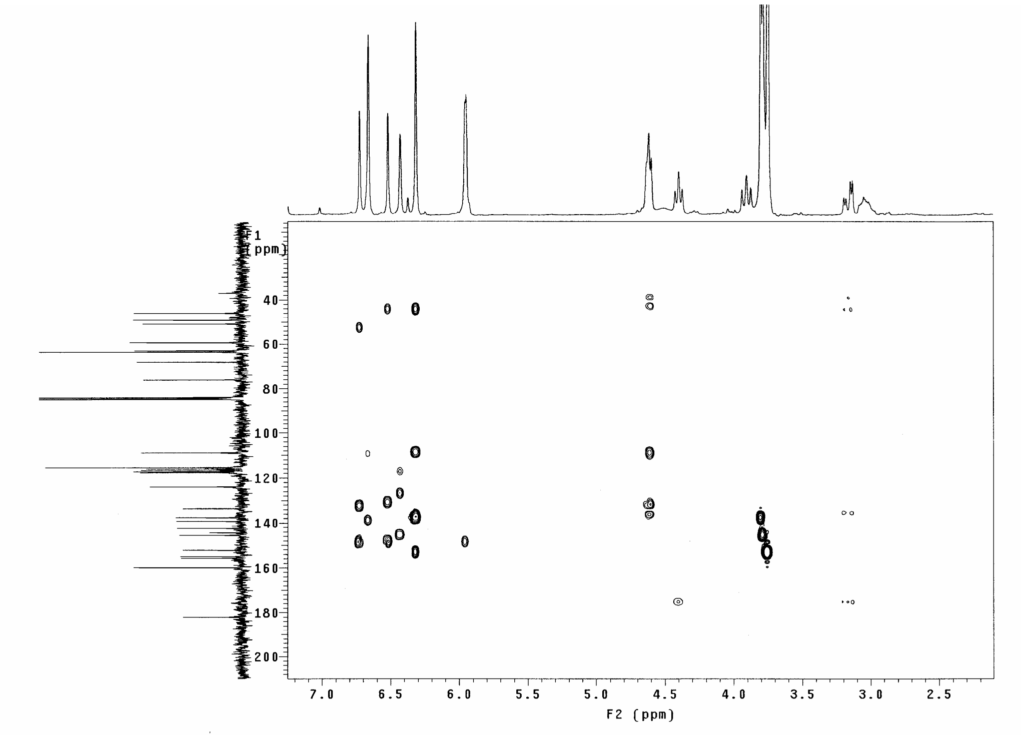

HSQC spectrums for Compound **15**.

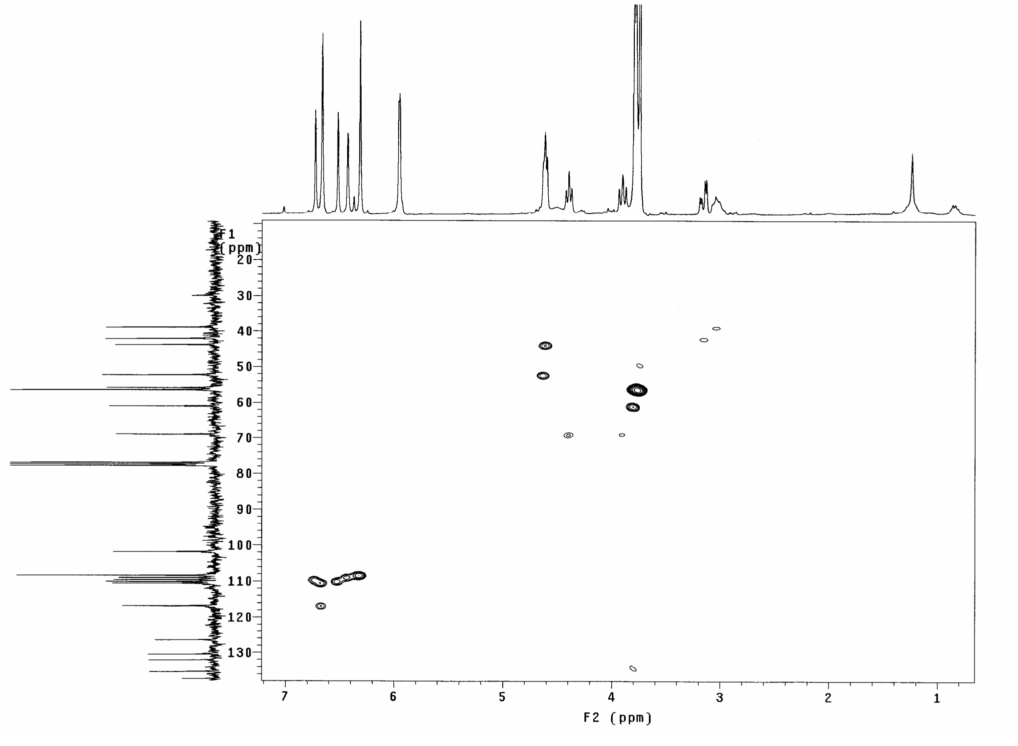

^{1}H-^{1}H COSY spectrums for Compound **15**.

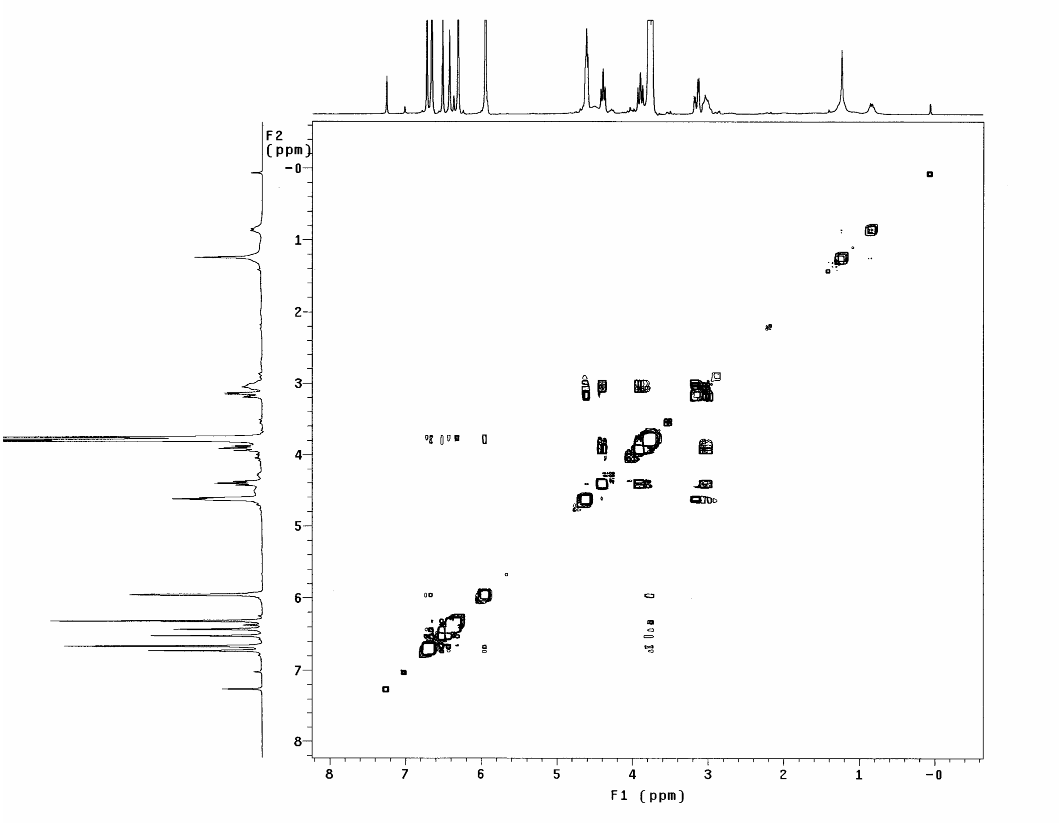

MS spectrums for Compound **15**.

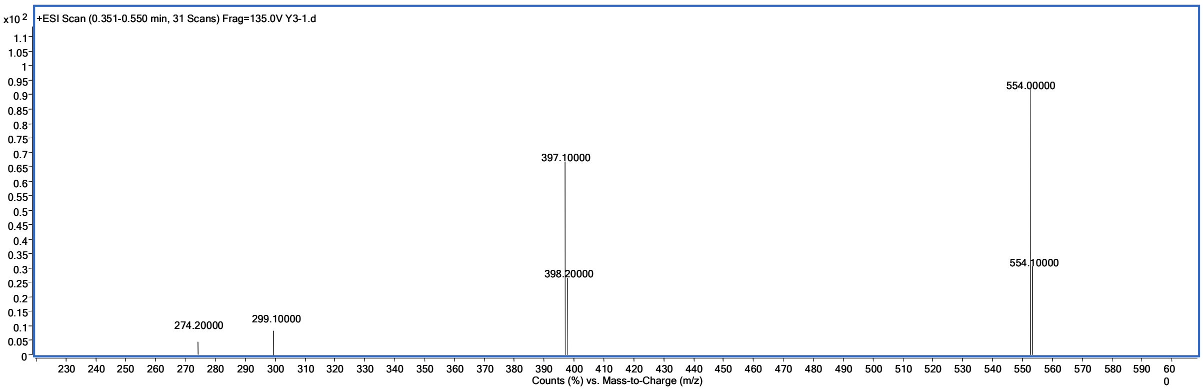

Compound 15 (4β-*NH*-(5-chloro-2-methoxyaniline)-4-deoxy-podophyllotoxin): 49% yield as white solid, ^{1}H NMR (300 MHz, $CDCl_3$): δ 2.978-3.037 (m, 1H, 2-H) , 3.104 (dd, *J*=4.8Hz, 1H, 3-H), 3.758 (s, 6H, 3′, 5′-OCH_3), 3.838 (s, 3H, Ar-OCH_3) , 3.960 (s, 3H, 4′-OCH_3) , 3.991 (t, *J*=9.9 Hz, 1H, 11-H), 4.388 (t, *J*=7.8Hz, 1H, 11-H), 4.571 (d, *J*=4.2 Hz, 1H, 4-H), 5.940 (d, *J*=7.2 Hz, 2H, OCH_2O), 6.316 (s, 2H, ArH), 6.407 (dd, *J*=2.1 Hz, 1H, ArH) , 6.514 (s, 1H, ArH), 6.617 (d, *J*=2.4

Hz, 1H, ArH) , 6.750 (s, 1H, ArH), 6.824 (t, *J*=8.7 Hz, 1H, ArH) ^{13}C NMR (75 MHz, $CDCl_3$): δ 38.890, 42.014, 43.814, 53.436, 56.503, 57.234, 60.976, 69.051, 101.773, 108.497, 109.285, 110.157, 111.536, 114.602, 123.972, 130.752, 131.906, 135.388, 137.488, 142.513, 147.858, 148.168, 148.477, 152.838, 174.981; MS (ESI): m/z: 554 $[M+H]^+$

^{13}C NMR spectrum of 4*β*-*NH*-(5-fluoro-2-methoxyaniline)-4-deoxy-podophyllotoxin **(16)**.

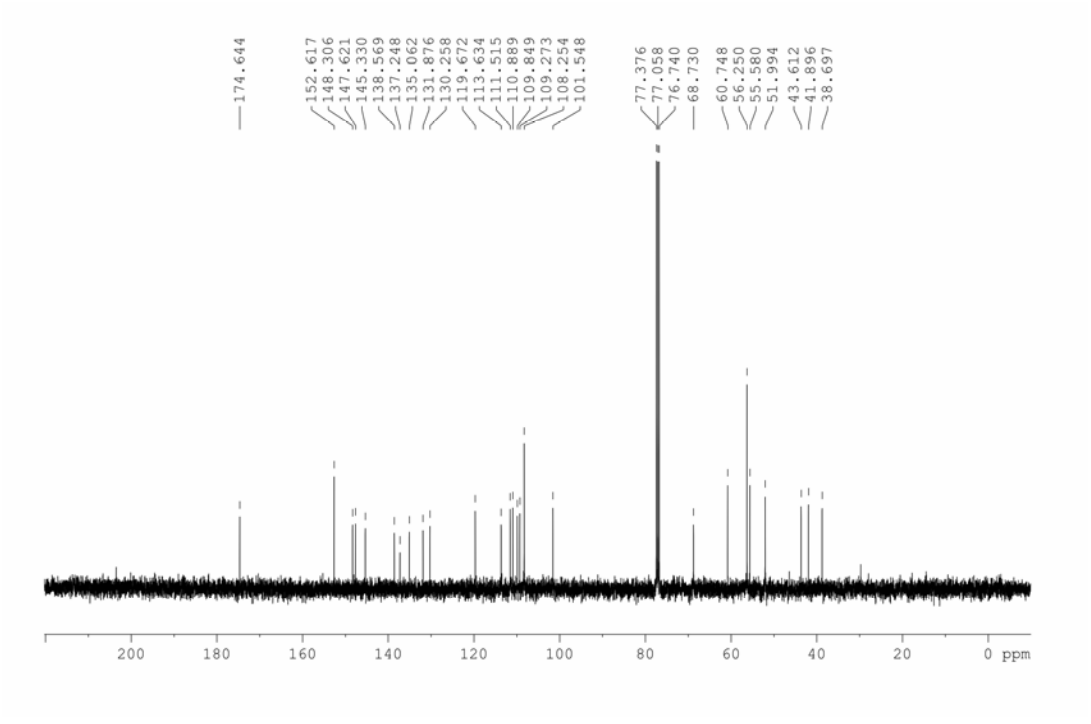

^{1}H NMR spectrum of Compound **16**.

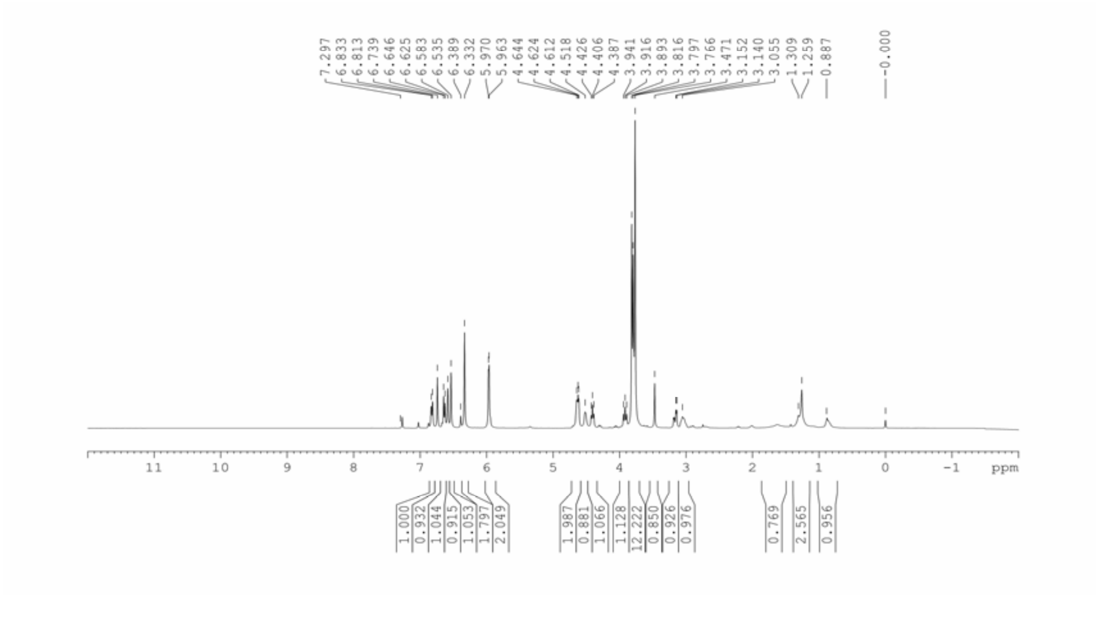

HMBC spectrums for Compound **16**.

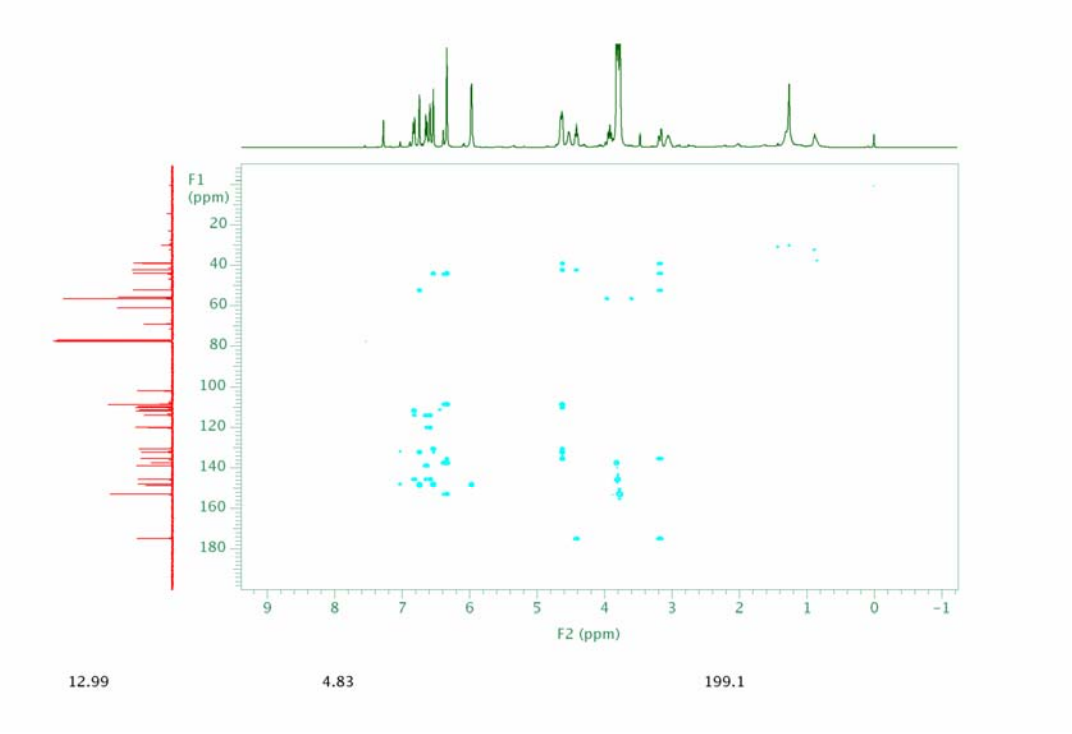

HSQC spectrums for Compound **16**.

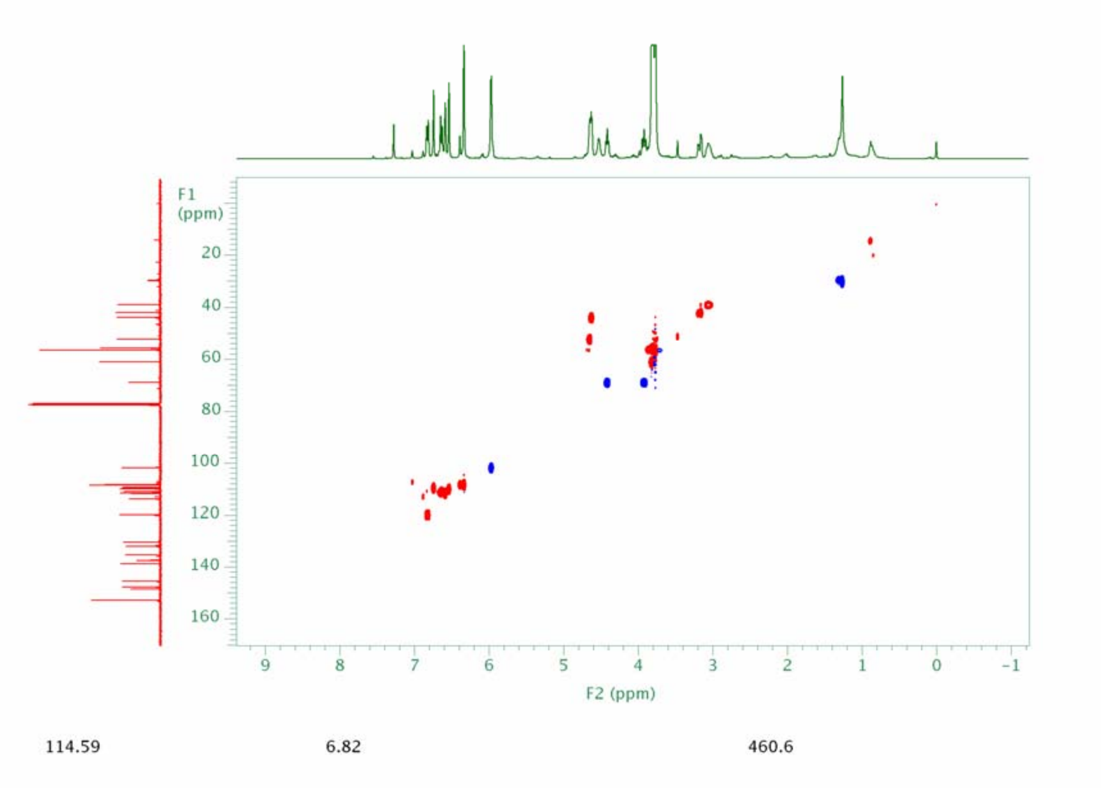

^{1}H-^{1}H COSY spectrums for Compound **16**.

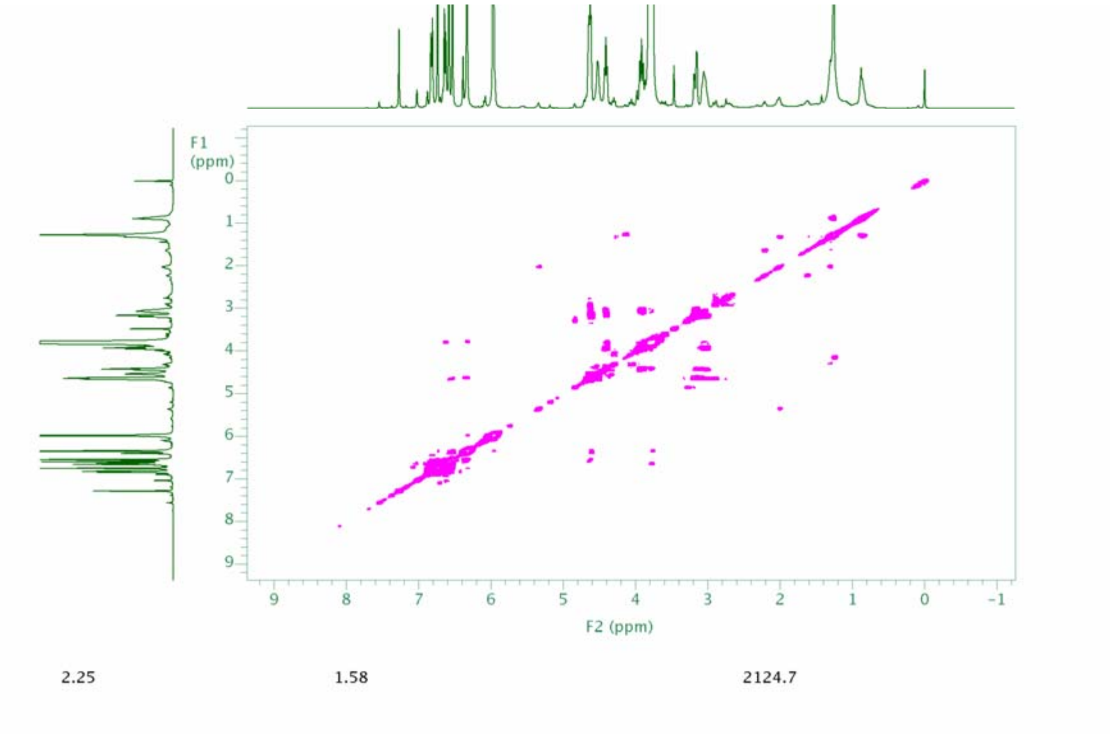

MS spectrums for Compound **16**.

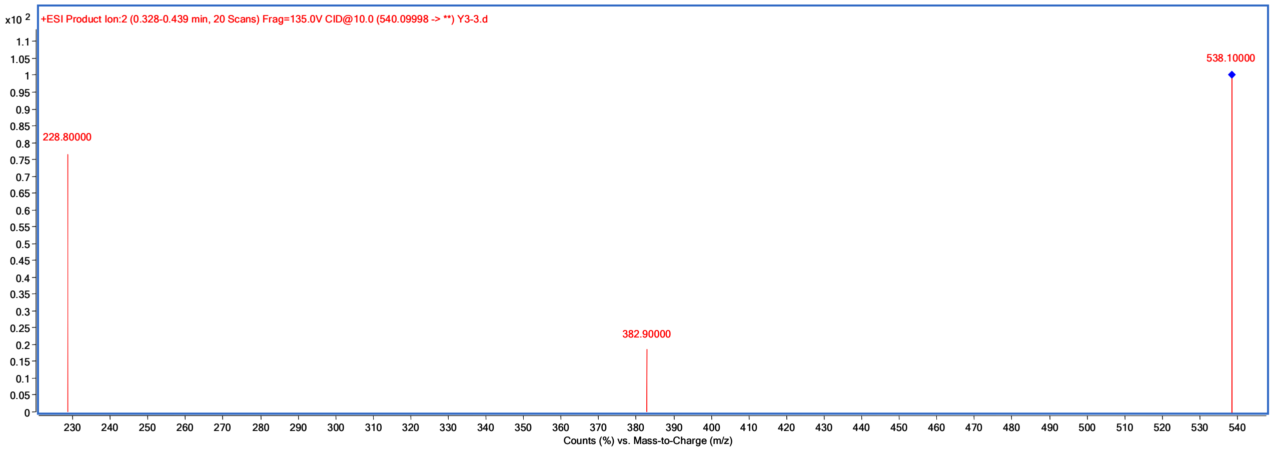

Compound 16 (4β-*NH*-(5-fluoro-2-methoxyaniline)-4-deoxy-podophyllotoxin): 76% yield as white solid, ^{1}H NMR (300 MHz, $CDCl_3$): 2.997-3.009 (m, 1H, 2-H), 3.110 (dd, *J*=4.8 Hz, 1H, 3-H), 3.757 (s, 6H, 3′, 5′-OCH_3), 3.808 (d, J=8.1 Hz, 6H, 4′-OCH_3, Ar-OCH_3) 3.995 (t, J=9.9 Hz, 1H, 11-H), 4.386 (t, *J*=7.5 Hz, 1H, 11-H), 4.579 (t, J=5.1 Hz, 2H, 4-H, 1-H), 5.952 (d, J=5.1 Hz 2H, OCH_2O), 6.235 (d, J=9.0 Hz, 1H, ArH), 6.315 (s, 2H, ArH) , 6.380 (s, 1H, ArH), 6.520 (s, 1H, ArH), 6.755 (s, 1H, ArH), 6.853 (t, *J*=9.0 Hz, 1H, ArH) ^{13}C NMR (75 MHz, $CDCl_3$):δ 38.890 , 42.013,

43.814, 53.436, 56.503, 57.713, 61.005, 69.051, 101.576,101.801, 107.456, 108.497, 109.285, 110.185, 116.375, 130.696, 131.962, 135.338, 142.738, 147.887, 148.534, 153.867, 175.009; MS (ESI): m/z: 538 $[M+H]^+$

^{13}C NMR spectrum of 4*β*-*NH*-(3-bromo-4-methoxyaniline)-4-deoxy-podophyllotoxin **(17)**.

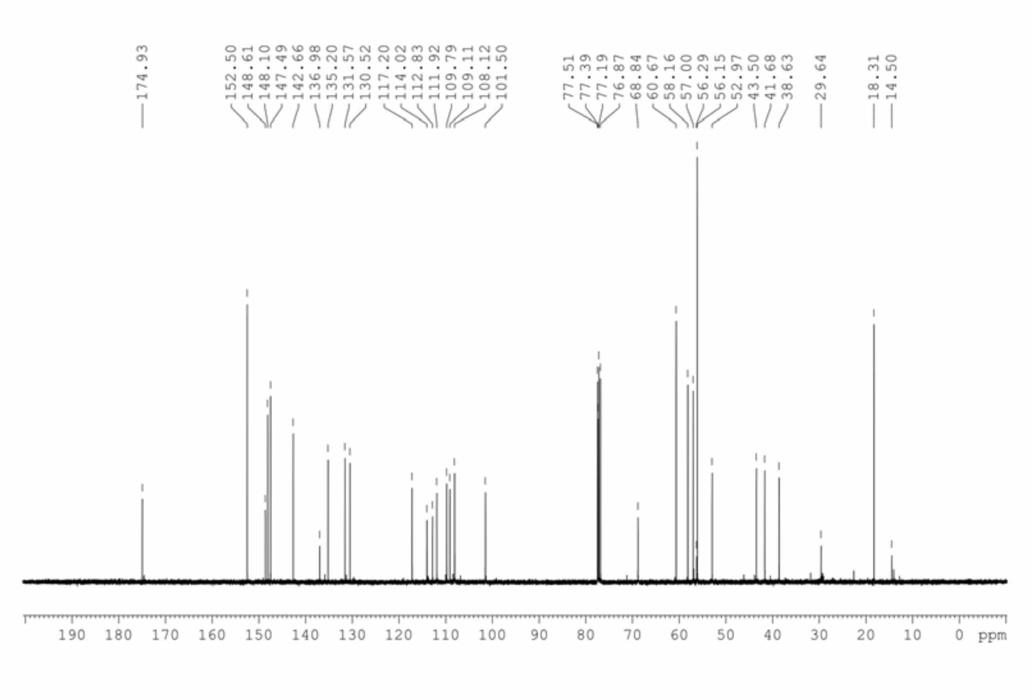

^{1}H NMR spectrum of Compound **17**.

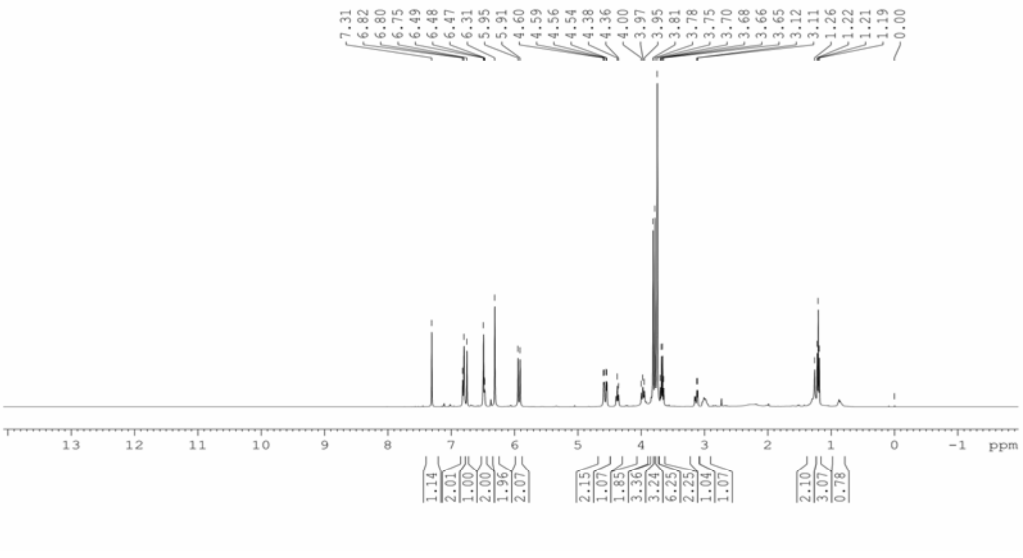

HMBC spectrums for Compound **17**.

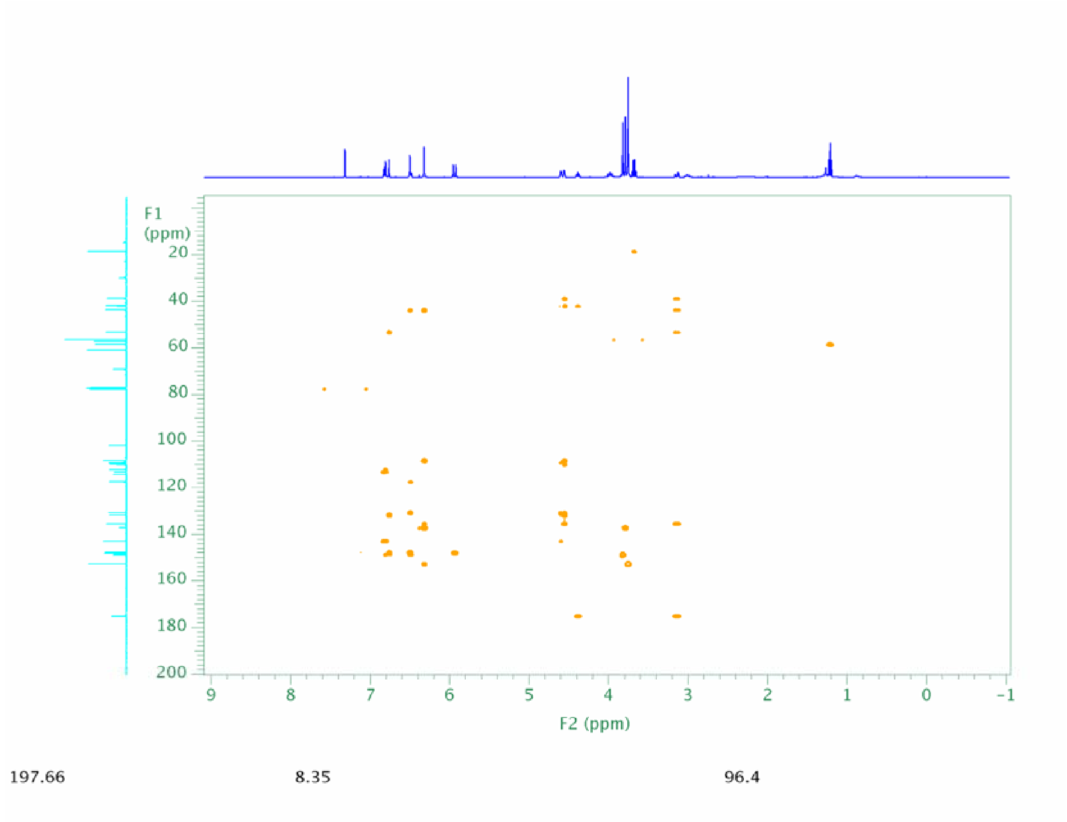

HSQC spectrums for Compound **17**.

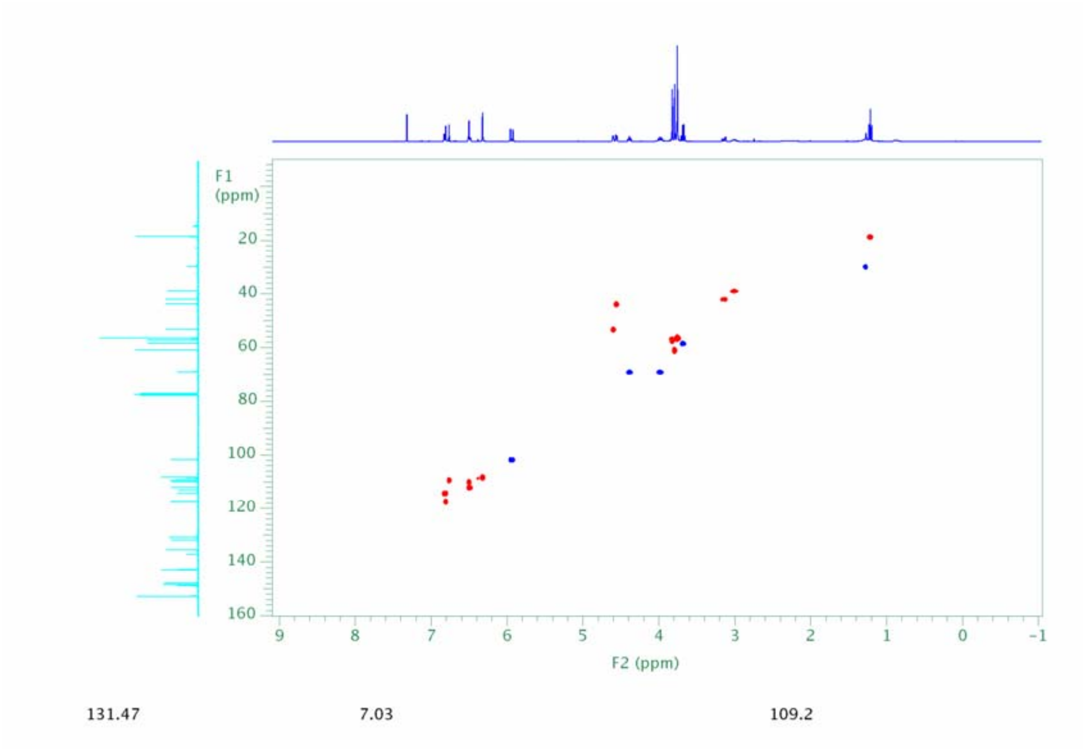

^{1}H-^{1}H COSY spectrums for Compound **17**.

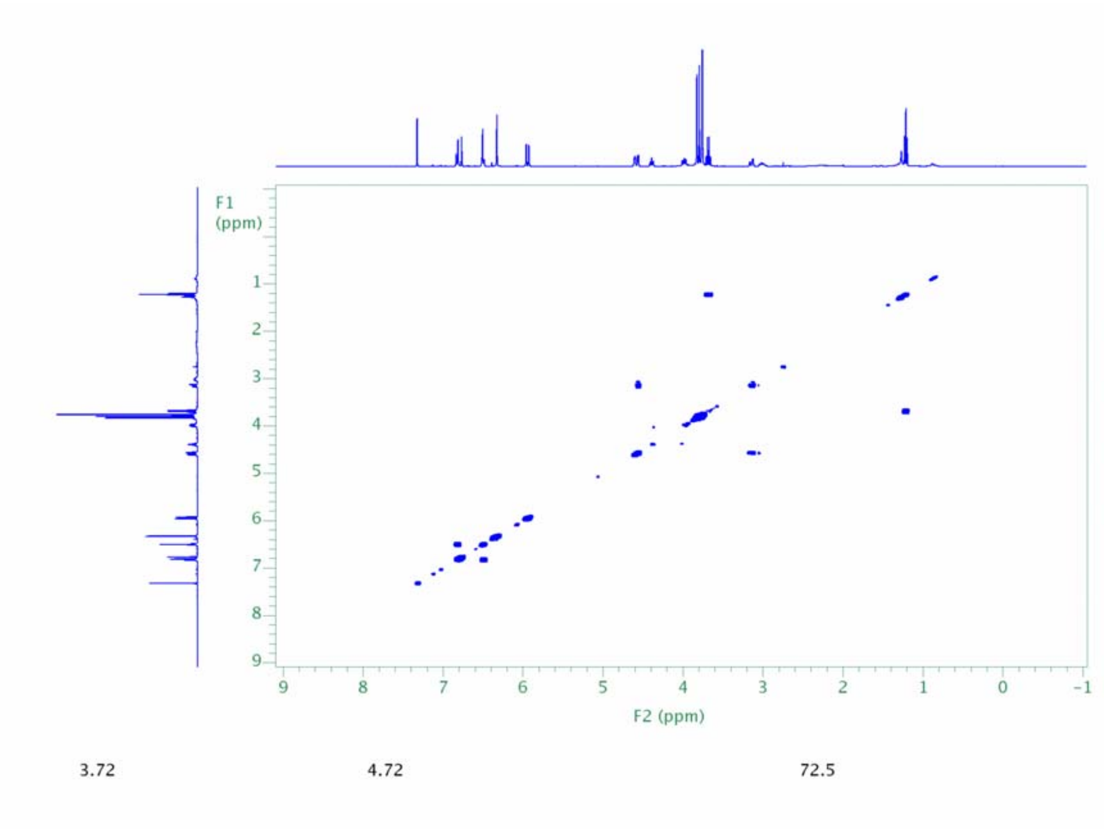

MS spectrums for Compound **17**.

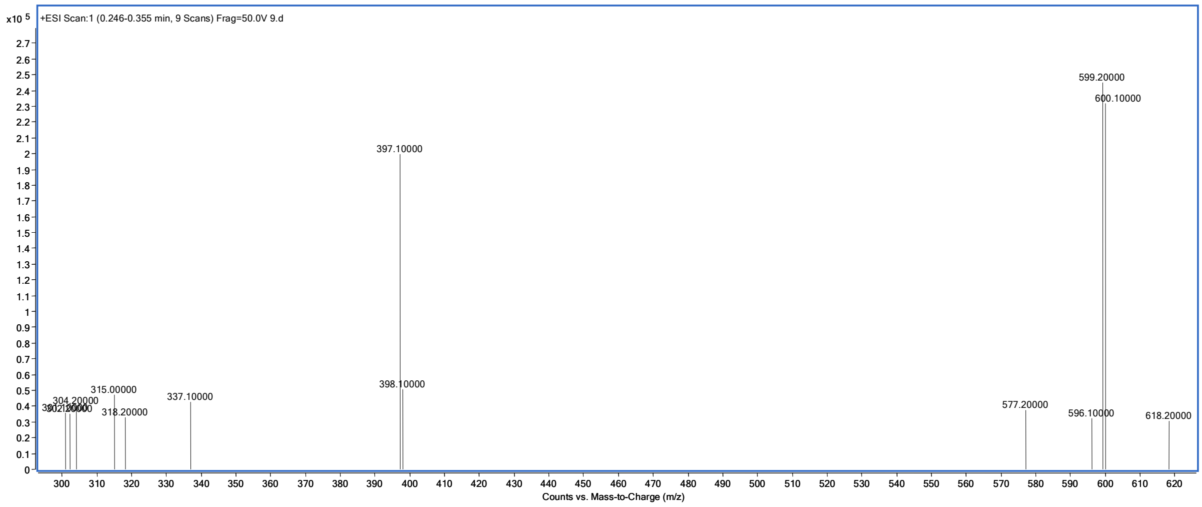

Compound 17 (4β-*NH*-(3-bromo-4-methoxyaniline)-4-deoxy-podophyllotoxin): 82% yield as white solid, ^{1}H NMR (300 MHz, $CDCl_3$): 2.997-3.009 (m, 1H, 2-H), 3.145 (dd, *J*=4.8 Hz, 1H, 3-H), 3.787 (s, 6H, 3′, 5′-OCH_3), 3.814 (s, J=8.1 Hz, 3H, Ar-OCH_3), 3.821 (s, 4′-OCH_3) 3.914 (t, *J*=9.9 Hz, 1H, 11-H), 4.405 (t, *J*=7.5 Hz, 1H, 11-H), 4.612 (t, *J*=4.8 Hz, 1H, 4-H), 4.642 (t, *J*=4.0 Hz, 1H,

1-H), 5.969 (d, J=5.1 Hz 2H, OCH_2O), 6.332 (s, 2H, ArH) , 6.534 (s, 1H, ArH), , 6.587 (d, J=1.6 Hz, 1H, ArH), 6.647 (s, 1H, ArH), 6.735 (s, 1H, ArH), 6.812 (dd, *J*=2.0 Hz, 1H, ArH) ^{13}C NMR (75 MHz, $CDCl_3$):δ38.686, 41.884, 43.614, 52.036, 55.583, 56.247, 60.741, 68.718, 101.547, 108.257, 109.273, 109.849, 110.905, 113.625, 119.733, 130.217, 131.884, 135.067, 137.245, 145.364, 147.613, 148.312, 152.613, 152.622, 174.625; MS (ESI): m/z: 599 $[M+H]^+$

^{13}C NMR spectrum of 4*β*-*NH*-(3-chloro-4-methoxyaniline)-4-deoxy-podophyllotoxin **(18)**.

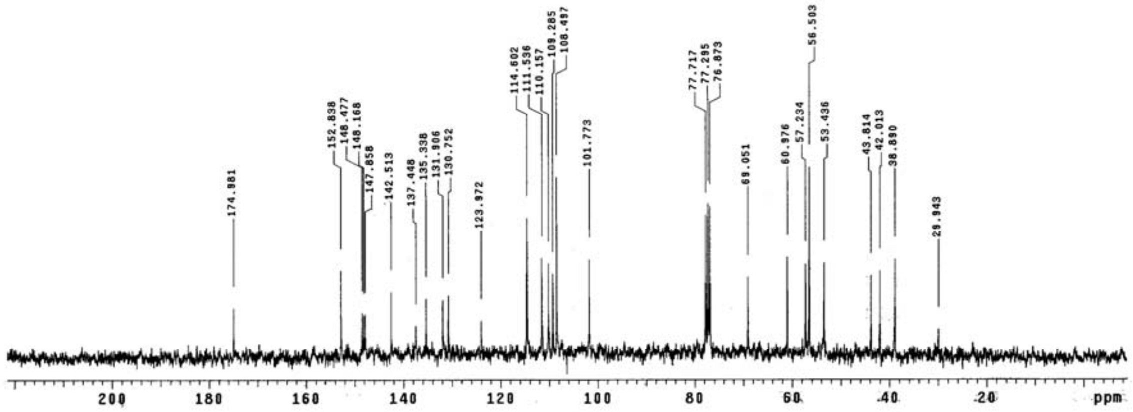

^{1}H NMR spectrum of Compound **18**.

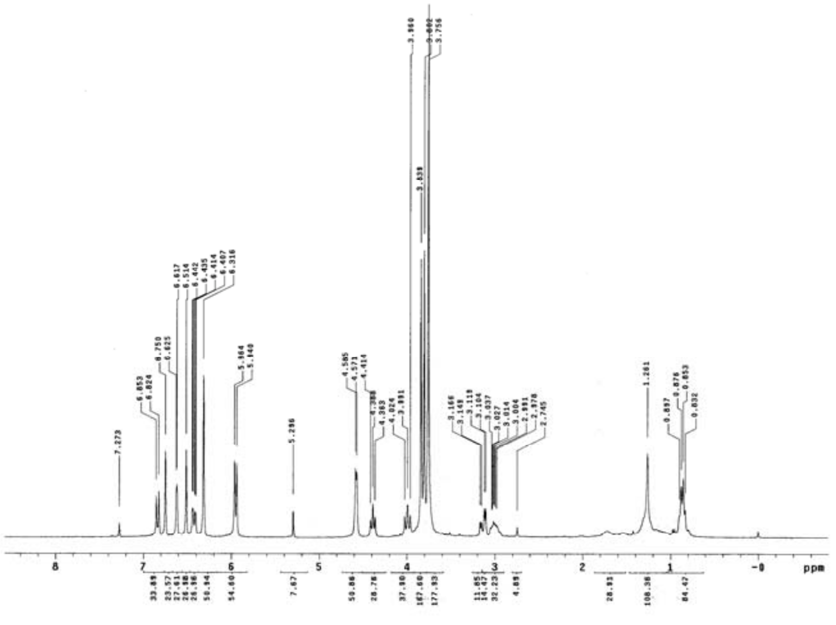

HMBC spectrums for Compound **18**.

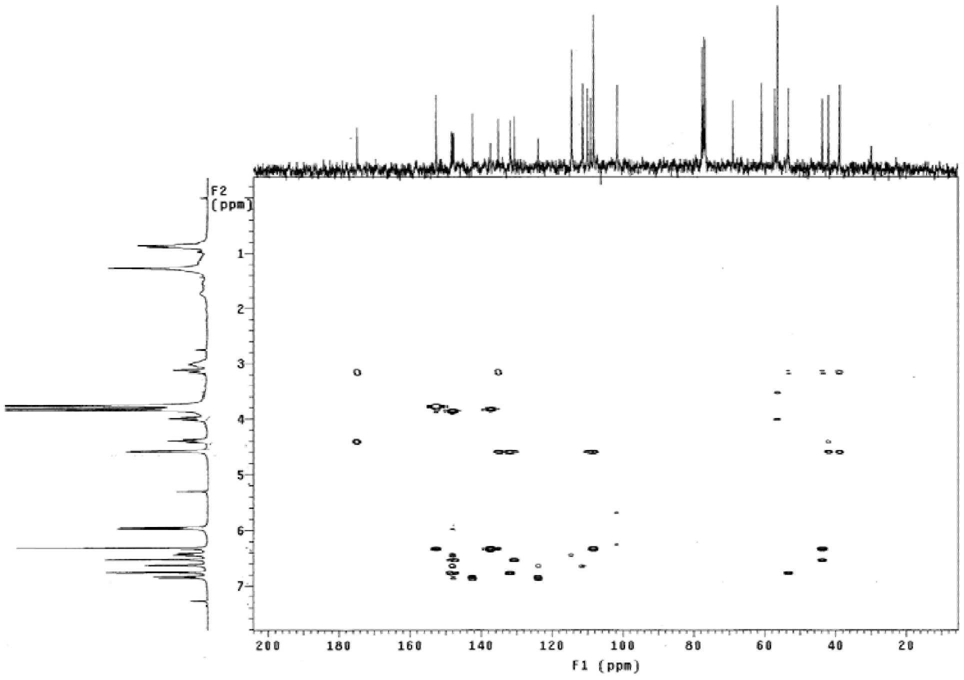

HSQC spectrums for Compound **18**.

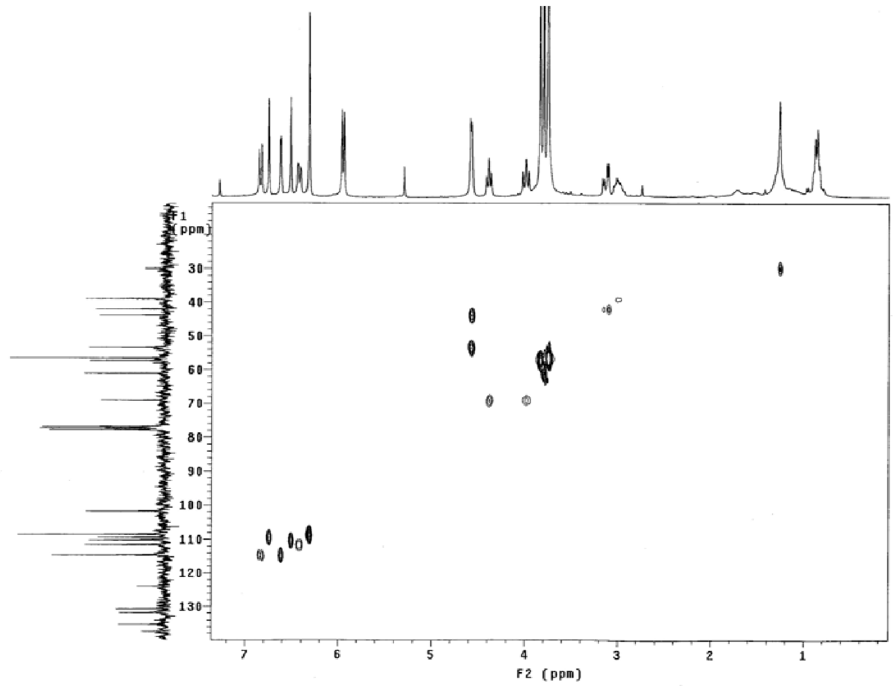

^1H-^1H COSY spectrums for Compound **18**.

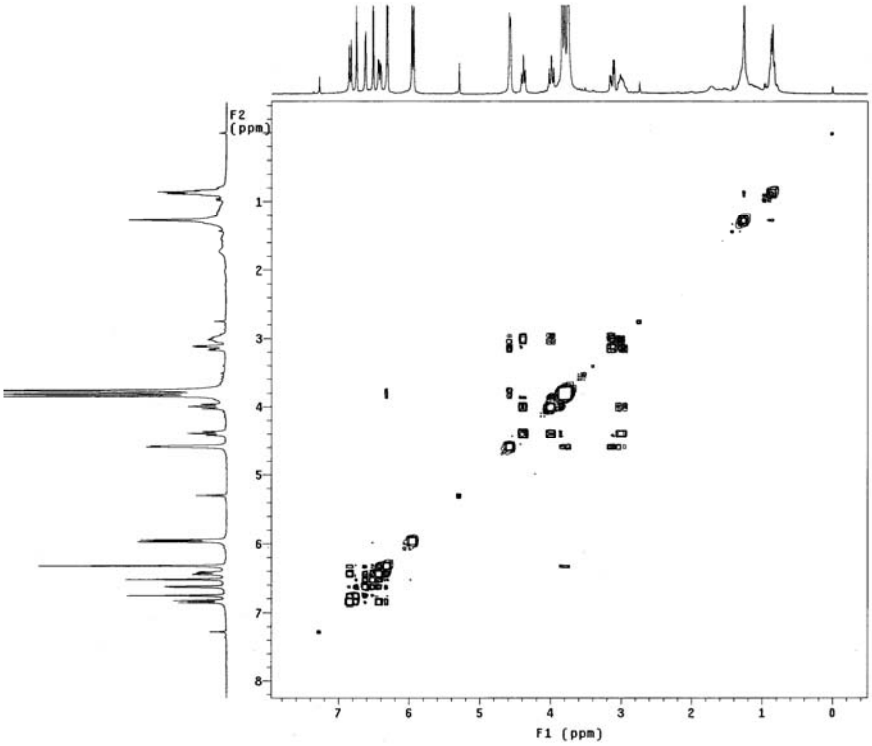

MS spectrums for Compound **18**.

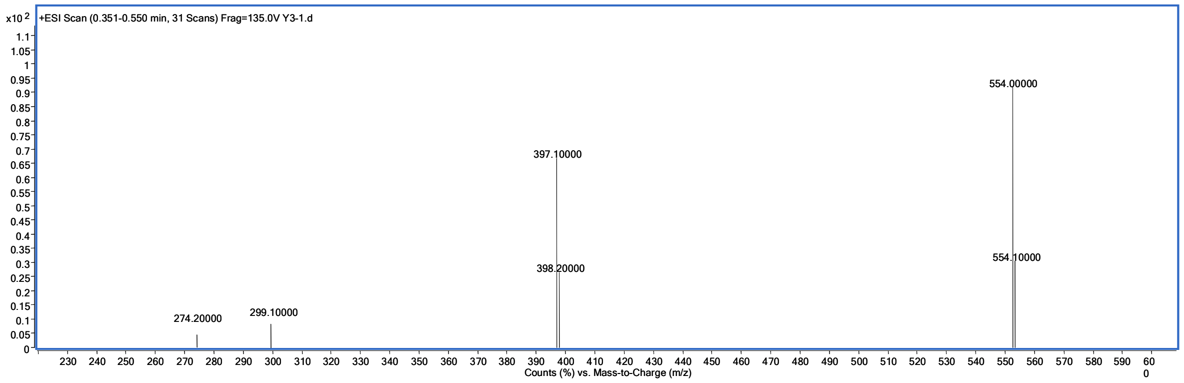

Compound 18 (4β-*NH*-(3-chloro-4-methoxyaniline)-4-deoxy-podophyllotoxin): 55% yield as white solid, ^{1}H NMR (300 MHz, $CDCl_3$): δ3.015-3.050 (m, 1H), 3.133 (dd, *J*=4.8 Hz, 1H), 3.752 (s, 6H), 3.783 (s, 3H), 3.802 (s, 3H), 3.905 (t, *J*=9.6 Hz, 1H), 4.399 (t, *J*=7.9 Hz, 1H), 4.601 (d, *J*=5.1 Hz, 2H), 5.954 (d, *J*=2.4 Hz, 2H), 6.318 (s, 2H), 6.432 (s, 1H), 6.521 (s, 1H),6.665 (s, 2H), 6.728 (s, 1H) ^{13}C NMR (75 MHz, $CDCl_3$): δ38.935, 42.114, 43.844, 52.238, 55.850, 56.461, 61.014, 69.001, 101.815, 108.352, 108.962, 109.548, 110.082, 110.540, 116.848, 126.463, 130.482, 132.085,

135.340, 137.325, 138.495, 145.057, 147.855, 148.542, 152.816, 174.971; MS (ESI): m/z: 554 $[M]^+$

^{13}C NMR spectrum of 4*β*-*NH*-(3-fluoro-4-methoxyaniline)-4-deoxy-podophyllotoxin **(19)**

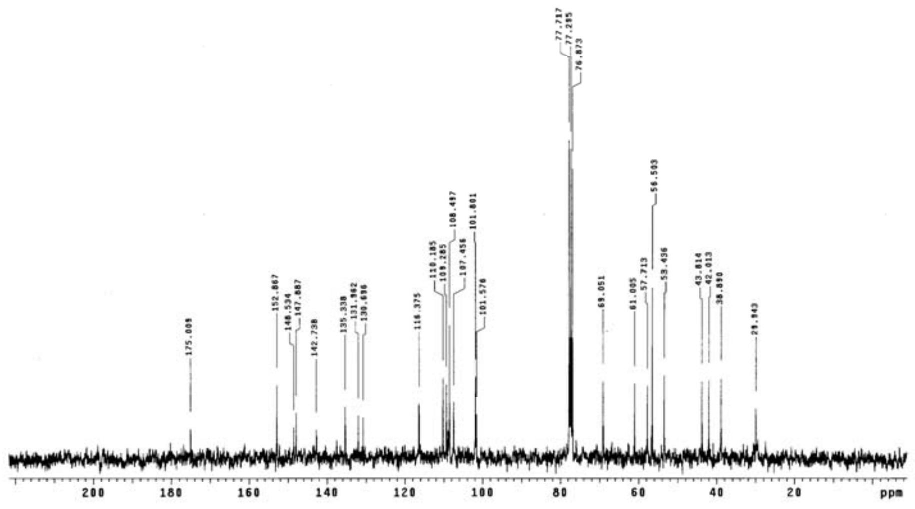

^{1}H NMR spectrum of Compound **19**.

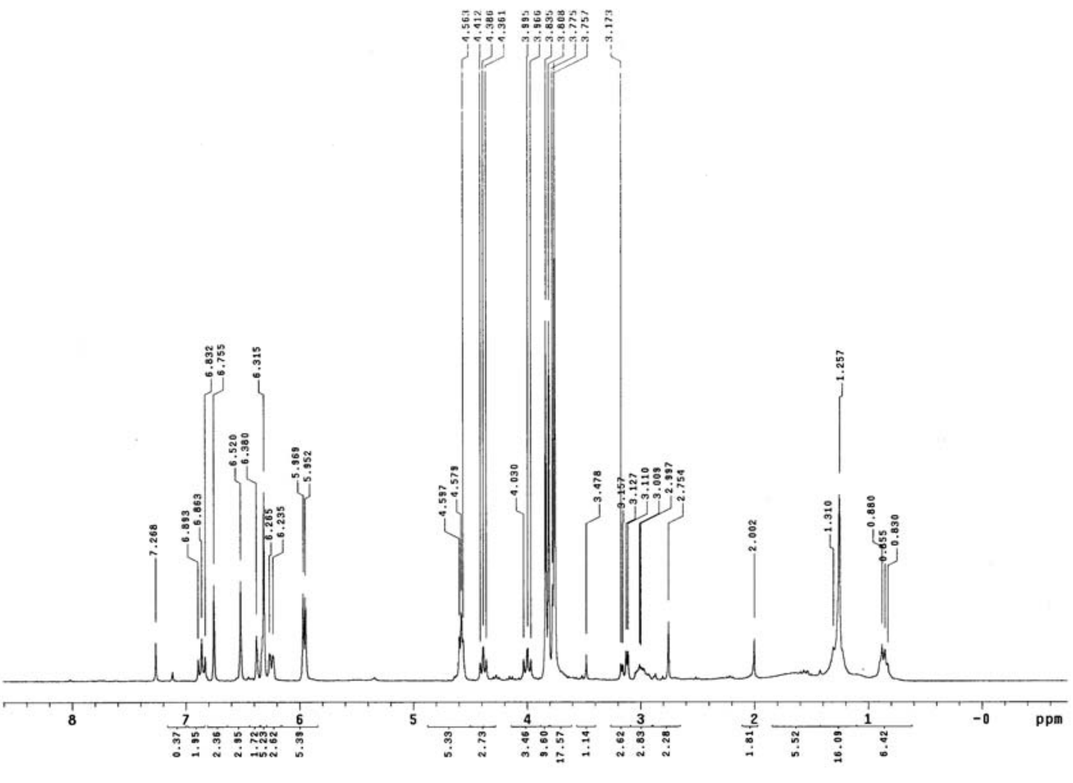

HMBC spectrums for Compound **19**.

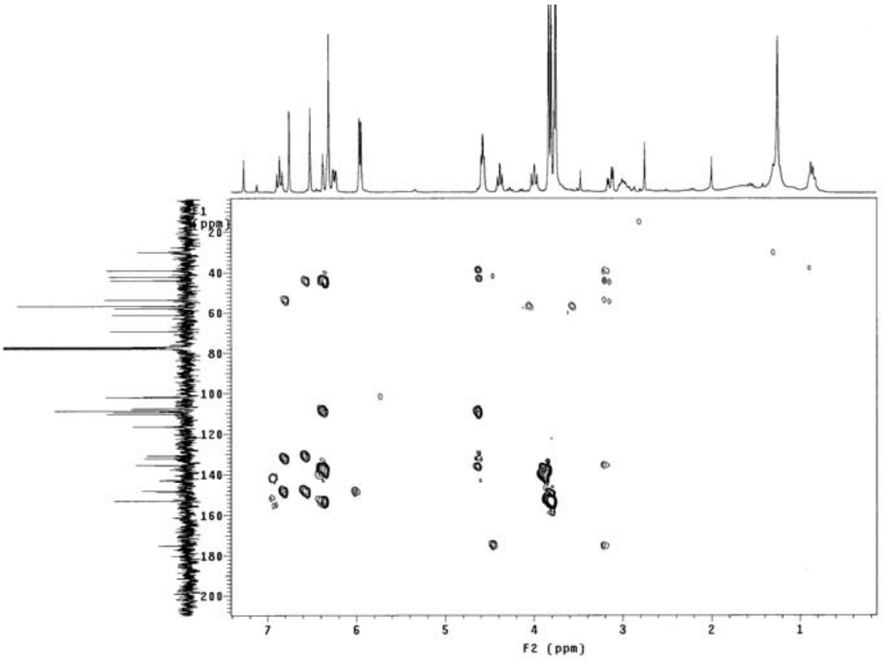

HSQC spectrums for Compound **19**.

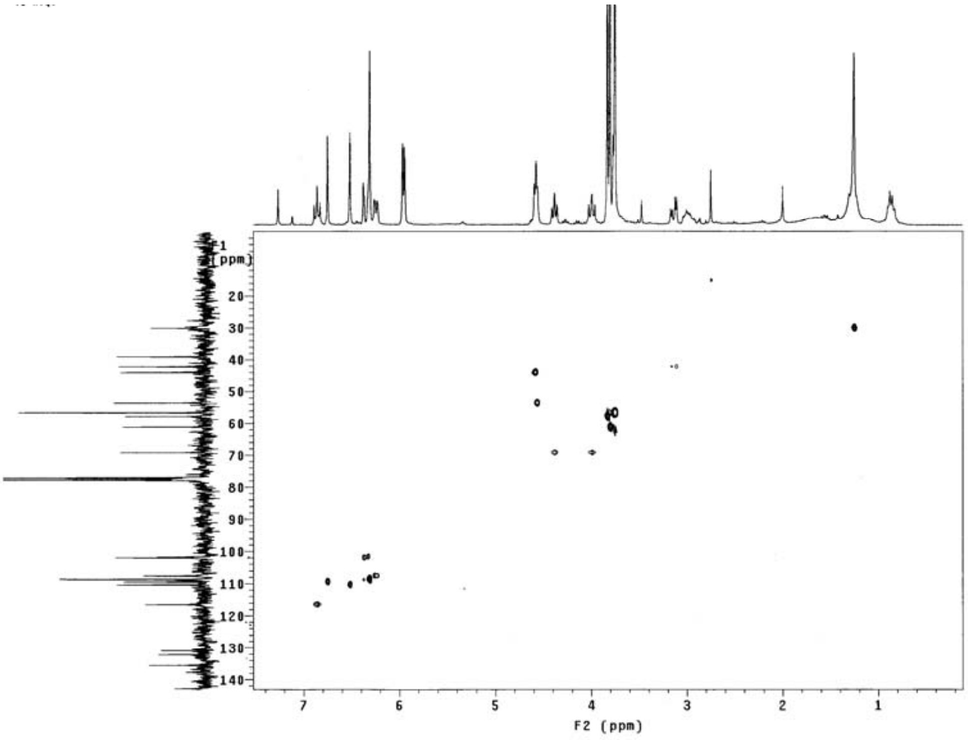

^{1}H-^{1}H COSY spectrums for Compound **19**.

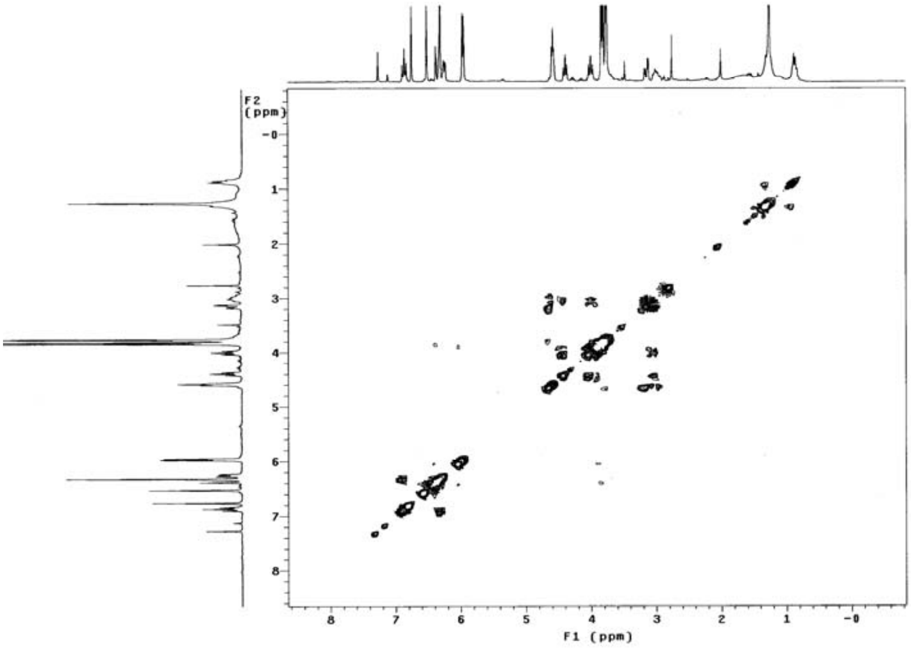

MS spectrums for compound **19**.

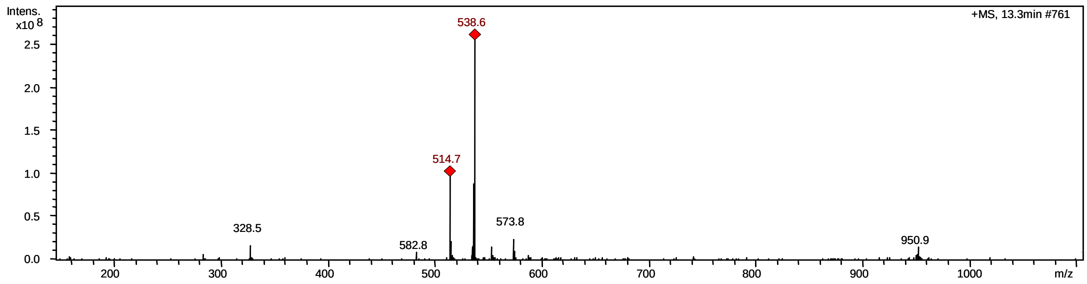

Compound 19 (4β-*NH*-(3-fluoro-4-methoxyaniline)-4-deoxy-podophyllotoxin): 39% yield as white solid, ^{1}H NMR (400 MHz, $CDCl_3$): 3.055 (m, 1H, 2-H), 3.140 (dd, *J*=4.8 Hz, 1H, 3-H), 3.766 (s, 6H, 3′, 5′-OCH_3), 3.797 (s, 3H, 4′-OCH_3), 3.816(s, 3H, Ar-OCH_3), 3.916 (t, *J*=9.2 Hz, 1H, 11-H), 4.406 (t, *J*=8.2 Hz, 1H, 11-H), 4.624 (t, *J*=4.8 Hz, 1H, 4-H), 5.963 (d, J=2.8 Hz 2H, OCH_2O), 6.332 (s, 2H, ArH) , 6.535 (s, 1H, ArH), , 6.583 (s, 1H, ArH), 6.625(d, *J*=8.4Hz, 1H, ArH), 6.739 (s, 1H, ArH), 6.8132(d, *J*=8.0 Hz, 1H, ArH) ^{13}C NMR (100 MHz, $CDCl_3$): δ 38.697, 41.896, 43.612, 51.994, 55.580, 56.250, 60.748, 68.730, 101.548, 108.254, 109.273, 109.849, 110.889, 111.515, 113.634, 119.672, 130.258, 131.876, 135.062, 137.248, 138.569, 145.330, 147.621, 148.306,

152.617, 174.644; MS (ESI): m/z: 538 $[M]^+$

^{13}C NMR spectrum of 4*β*-*NH*-(5-bromo-2-methoxyaniline)-4-deoxy-4′-demethylepipodophyllotoxin **(14′)**.

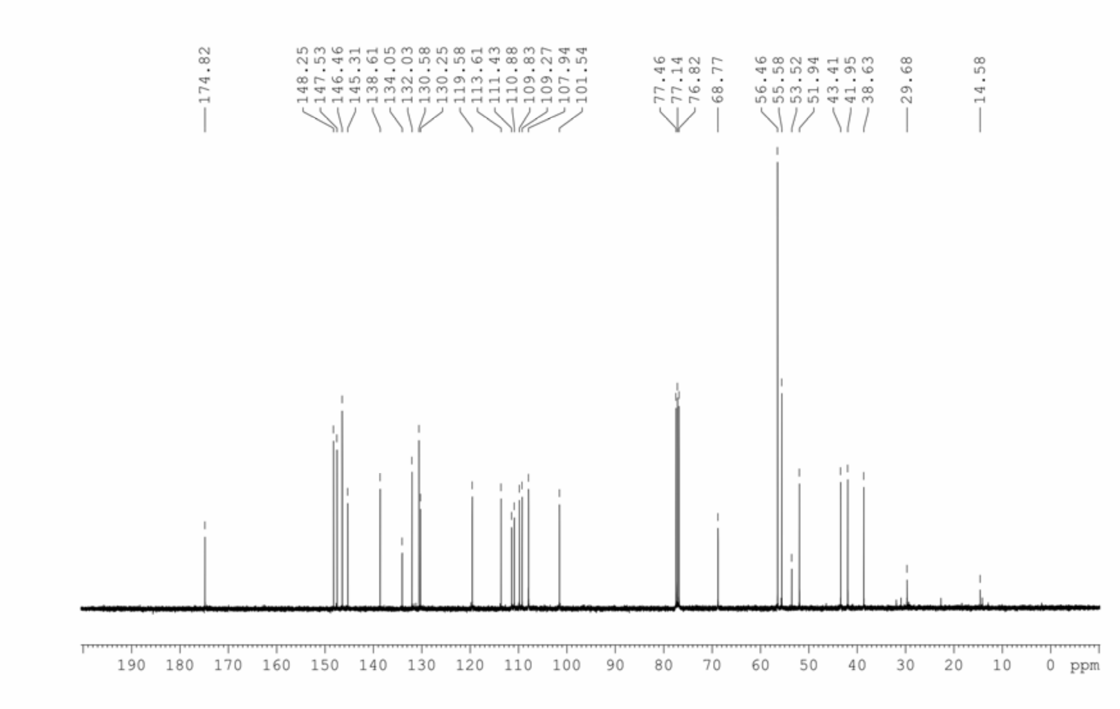

^{1}H NMR spectrum of Compound **14′**.

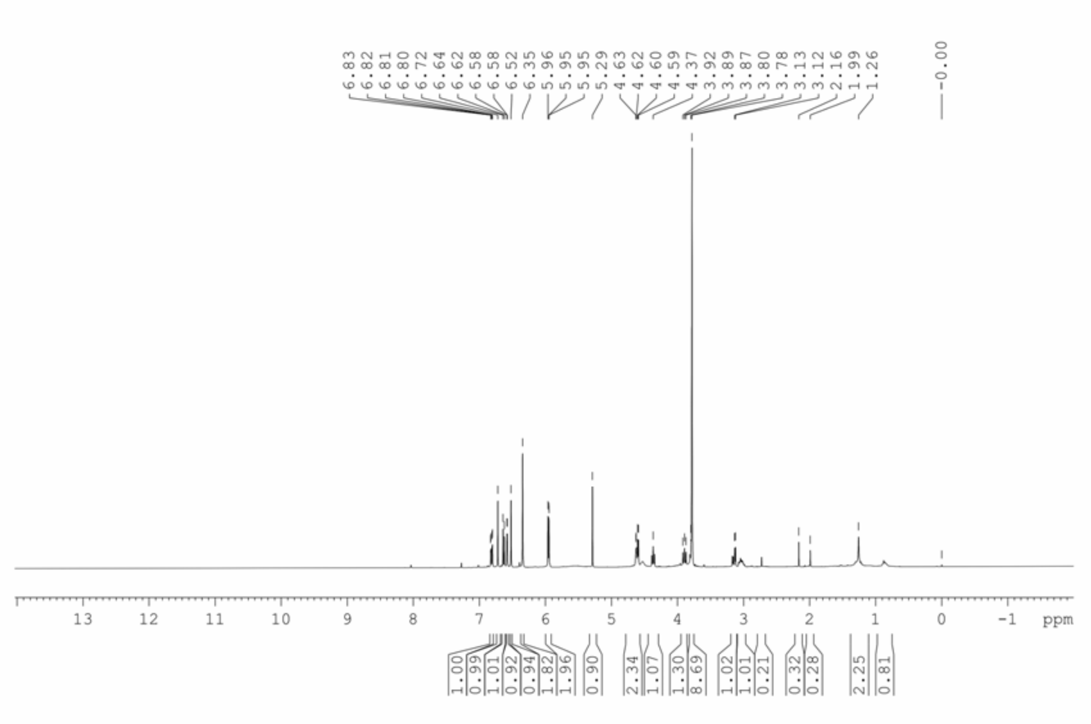

HMBC spectrums for Compound **14′**.

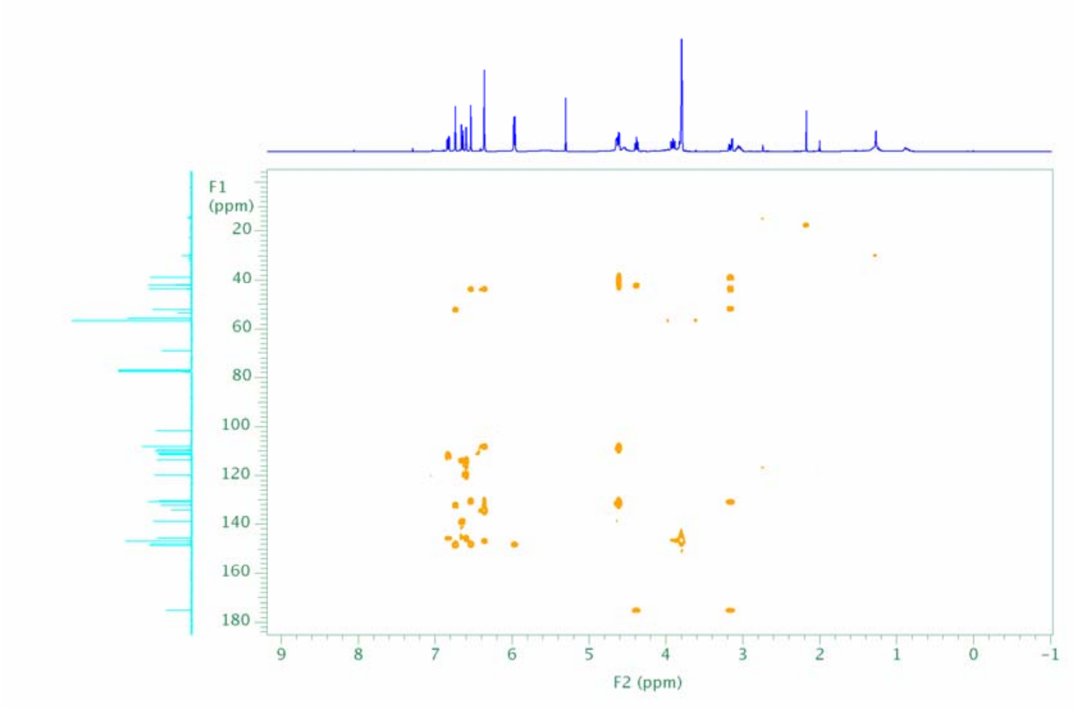

HSQC spectrums for Compound **14′**.

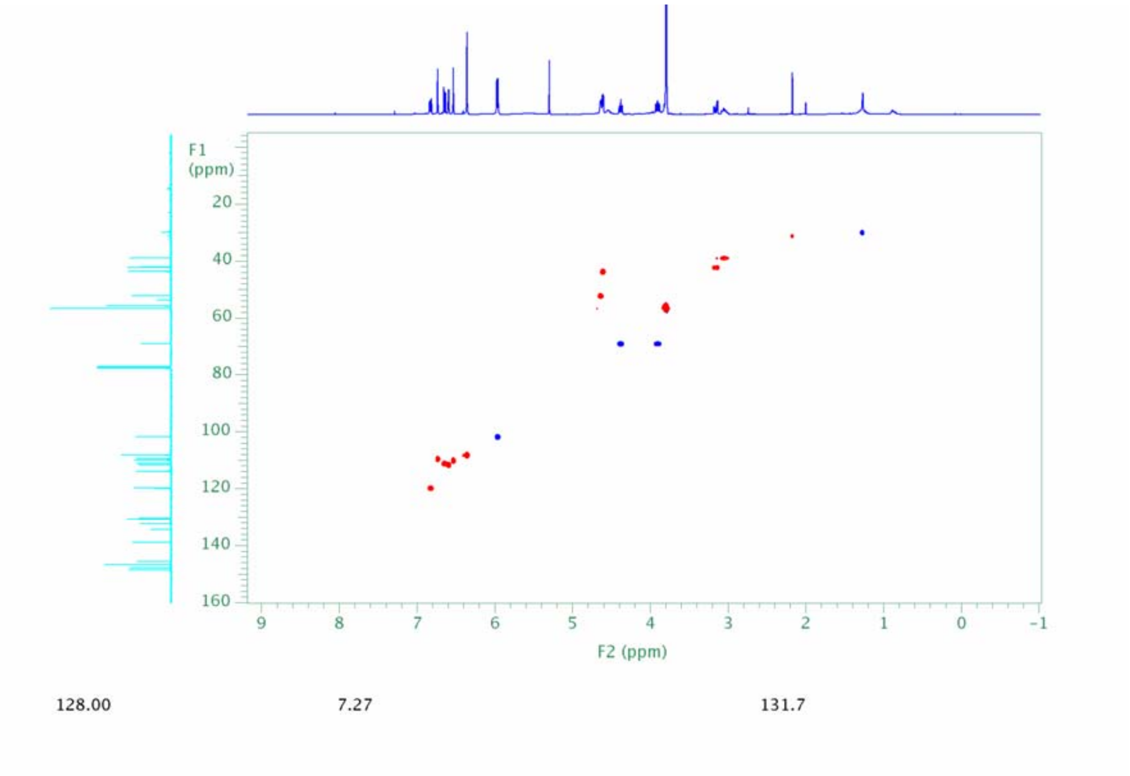

^{1}H-^{1}H COSY spectrums for Compound **14′**.

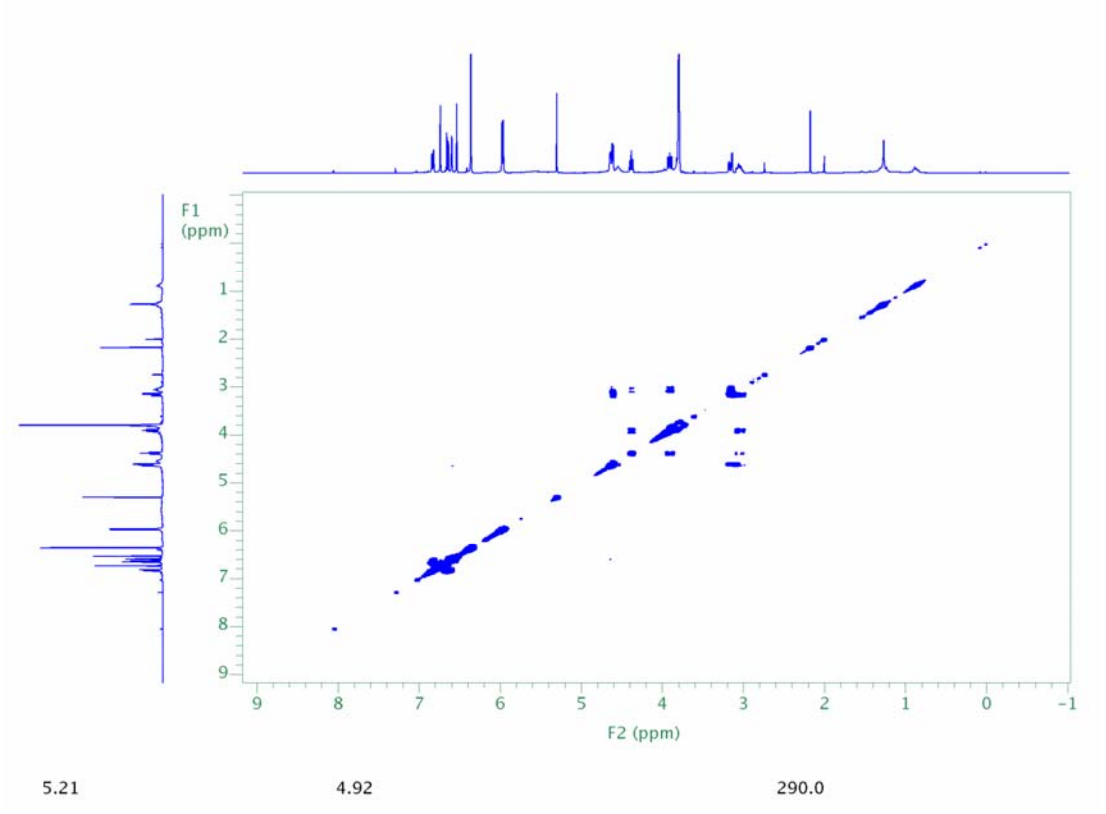

MS spectrums for Compound **14′**

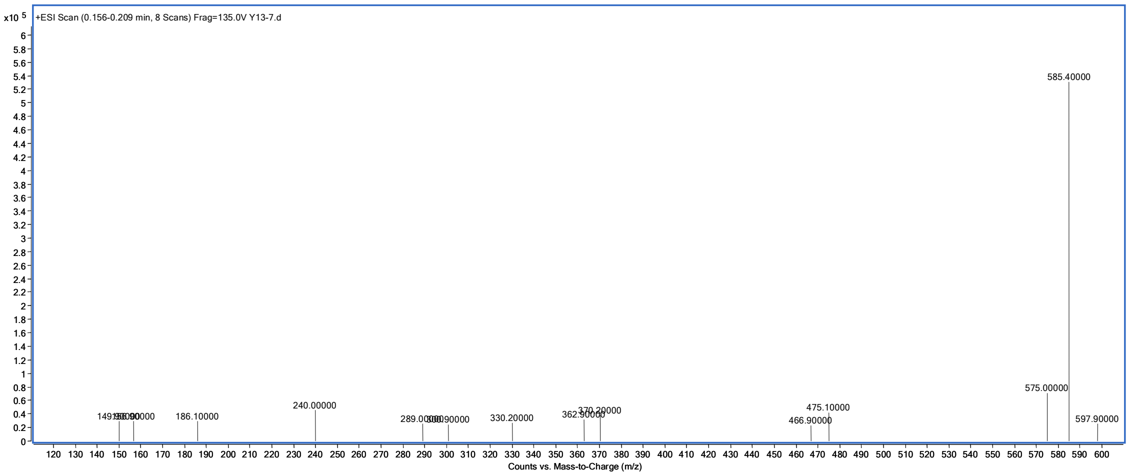

Compound 14′ (4*β*-*NH*-(5-bromo-2-methoxyaniline)-4-deoxy-4′-demethylepipodophyllotoxin): 39% yield as white solid, ^{1}H NMR (400 MHz, $CDCl_3$): δ 3.09 (m, 1H, 2-H) , 3.11 (dd, *J*=4.0Hz, 1H, 3-H), 3.75 (s, 6H, 3′, 5′-OCH_3), 3.80 (s, 3H, Ar-OCH_3) , 3.81 (s, 3H, 4′-OCH_3) , 3.94 (t, *J*=8.0 Hz, 1H, 11-H), 4.37 (t, *J*=8.0Hz, 1H, 11-H), 4.58 (d, *J*=4.0 Hz, 1H, 4-H), 4.65 (d, *J*=4.0 Hz, 1H,

1-H), 5.94 (d, *J*=8.0 Hz, 2H, OCH_2O), 6.23 (d, *J*=4.0 Hz, 1H, ArH), 6.31 (s, 2H, ArH), 6.46 (t, *J*=8.0 Hz, 1H, ArH), 6.52 (s, 1H, ArH), 6.76 (s, 1H, ArH), 7.11 (q, *J*=4.0 Hz, 1H, ArH) ^{13}C NMR (100 MHz, $CDCl_3$): δ38.48, 41.74, 43.39, 53.30, 56.55, 57.08, 68.63, 101.50, 108.07, 108.95, 109.73, 112.18, 112.71, 113.84, 117.10, 123.74, 130.31, 131.90, 133.57, 134.11, 141.00, 146.44, 147.57, 148.11, 175.00; MS (ESI): m/z: 585 $[M]^+$

^{13}C NMR spectrum of 4*β*-*NH*-(5-chloro-2-methoxyaniline)-4-deoxy-4′-demethylepipodophyllotoxin

(15′).

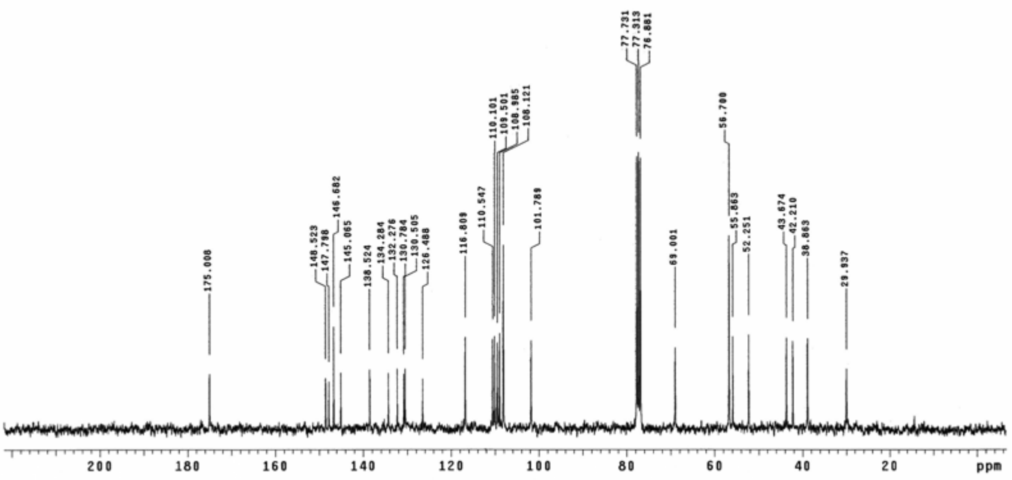

^{1}H NMR spectrum of Compound **15′**.

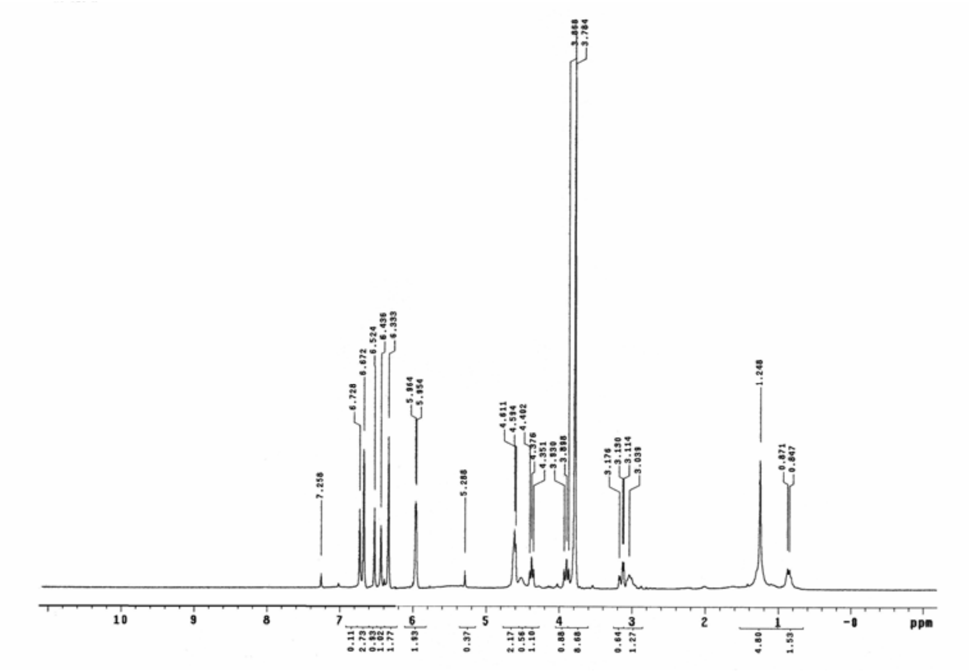

HMBC spectrums for Compound **15′**

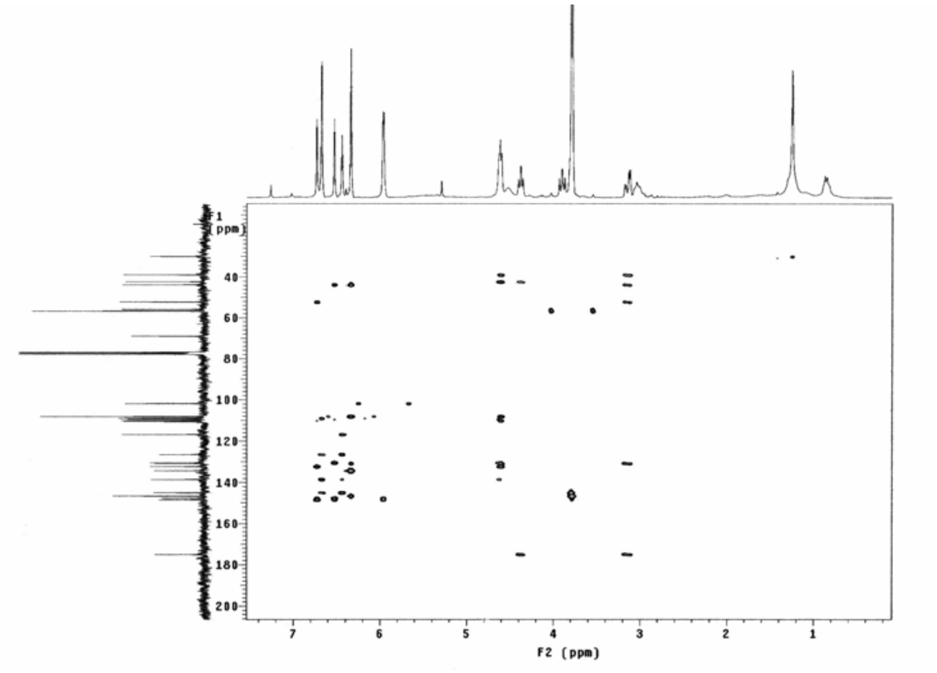

HSQC spectrums for Compound **15′**.

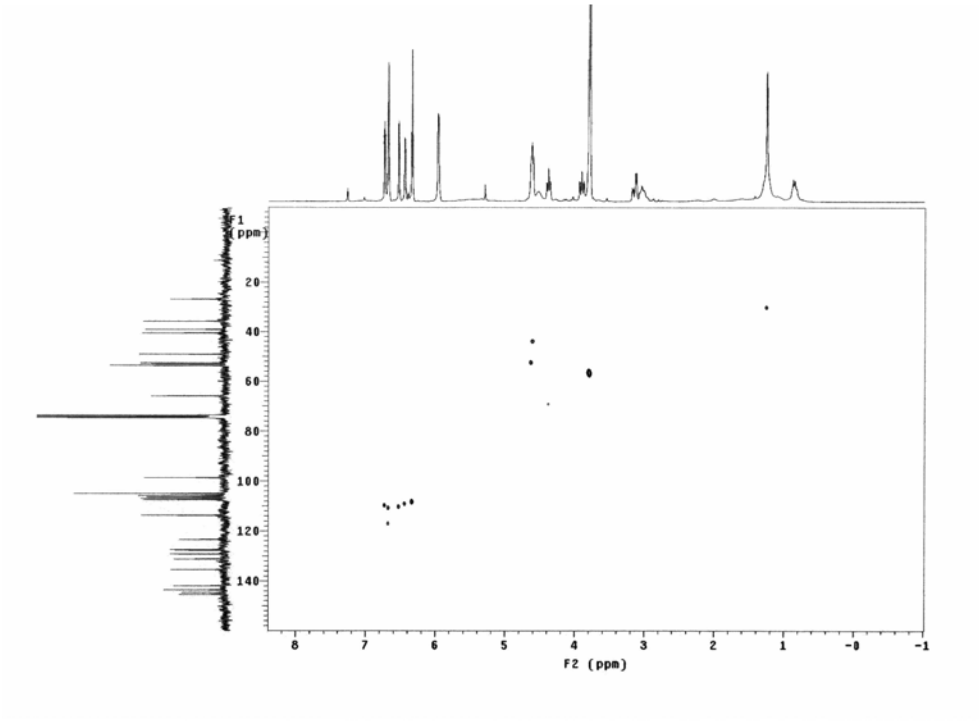

^{1}H-^{1}H COSY spectrums for Compound **15′**.

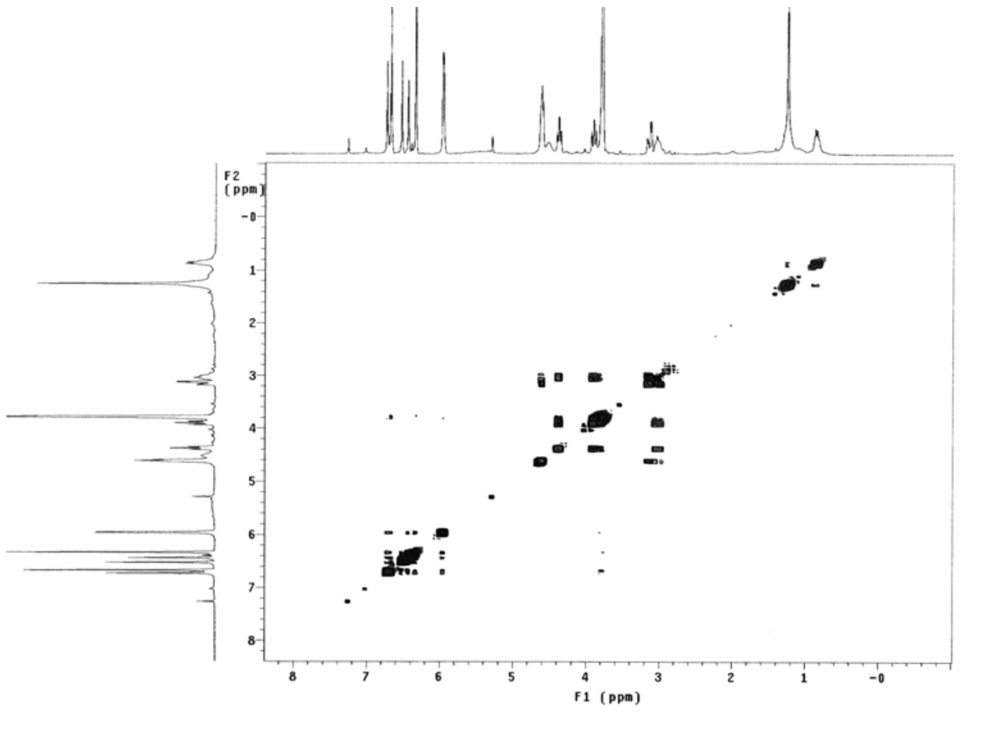

MS spectrums for Compound **15′**.

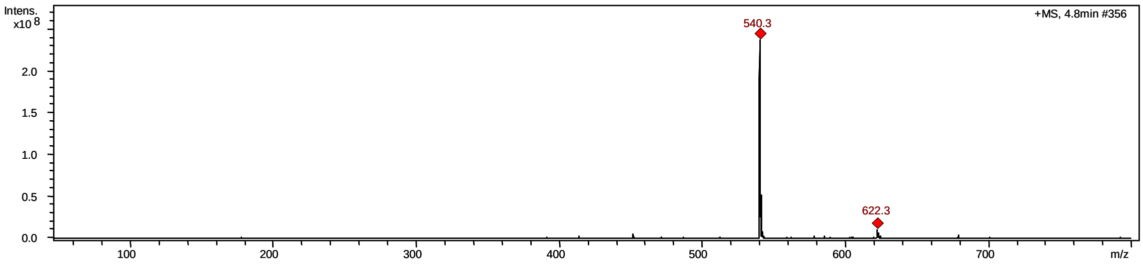

Compound 15′ (4*β*-*NH*-(5-chloro-2-methoxyaniline)-4-deoxy-4′-demethylepipodophyllotoxin): 76% yield as white solid, ^{1}H NMR (300 MHz, $CDCl_3$): δ 2.971-3.042 (m, 1H, 2-H) , 3.101 (dd, *J*=4.5Hz, 1H, 3-H), 3.793 (s, 6H, 3′, 5′-OCH_3), 3.849 (s, 3H, Ar-OCH_3) , 3.997 (t, *J*=9.9 Hz, 1H, 11-H), 4.383 (t, *J*=7.8Hz, 1H, 11-H), 4.584 (d, *J*=5.1Hz, 2H,1-H, 4-H), 5.959 (d, *J*=5.7Hz, 2H, OCH_2O), 6.327 (s, 2H, ArH), 6.402 (dd, *J*=2.7 Hz, 1H, ArH) , 6.526 (s, 1H, ArH), 6.616 (d, *J*=2.7 Hz, 1H, ArH) , 6.741 (s, 1H, ArH), 6.827 (d, *J*=8.4 Hz, 1H, ArH) ^{13}C NMR (75 MHz, $CDCl_3$): δ 38.821, 42.131, 43.639, 53.544, 56.734, 57.241, 69.014, 101.783, 108.204, 109.192, 110.206, 111.594, 114.638, 124.048, 130.762, 132.124, 134.393, 142.482, 146.713, 147.861, 148.261, 148.542, 174.998; MS (ESI): m/z: 540 $[M]^+$

^{13}C NMR spectrum of 4*β*-*NH*-(5-fluoro-2-methoxyaniline)-4-deoxy-4′-demethylepipodophyllotoxin **(16′)**.

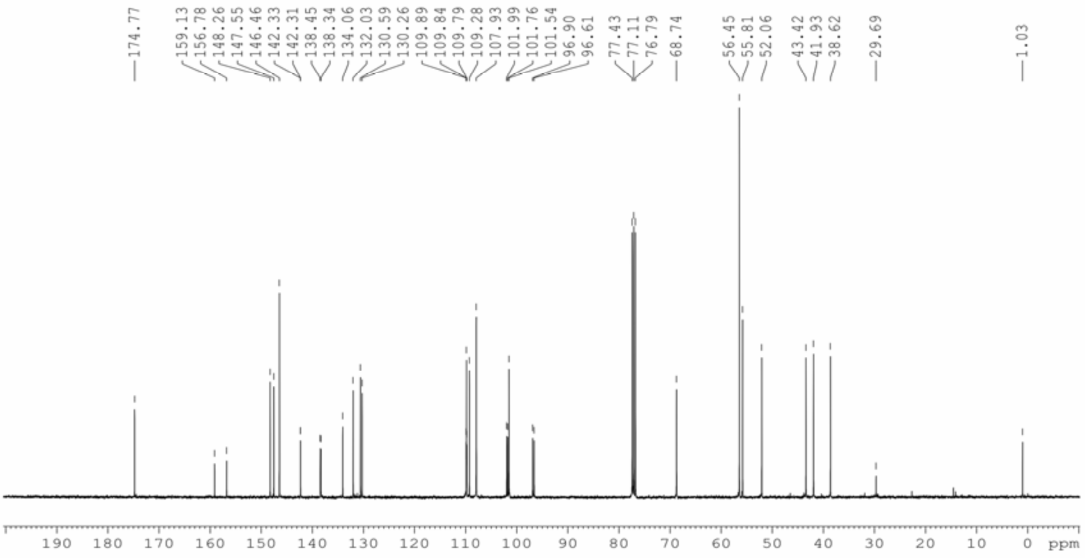

^{1}H NMR spectrum of Compound **16′**.

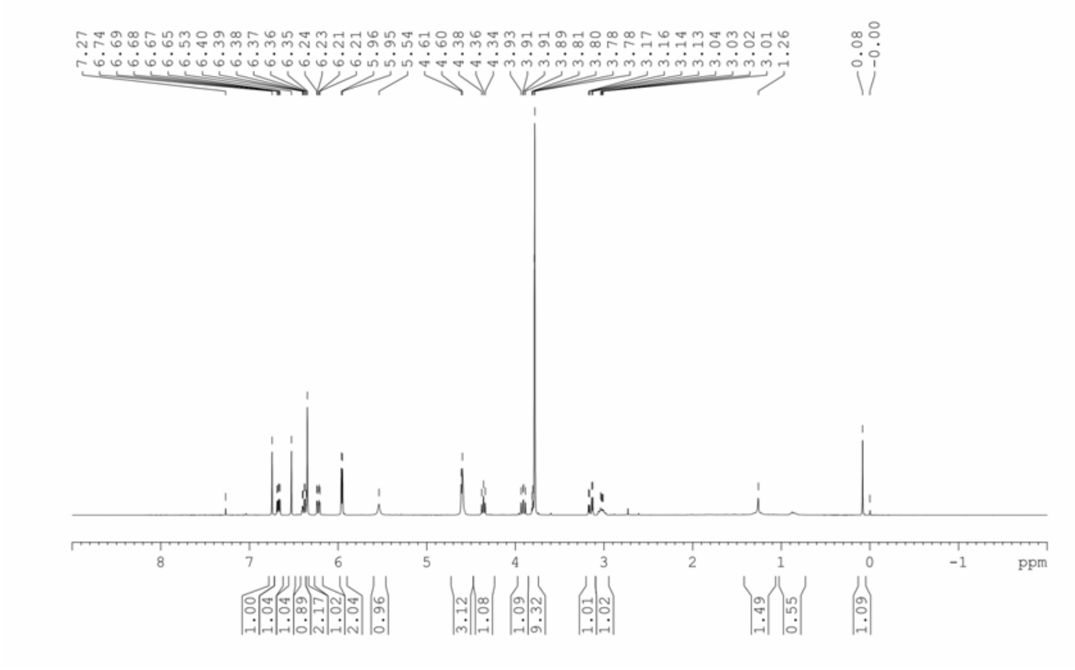

HMBC spectrums for Compound **16′**.

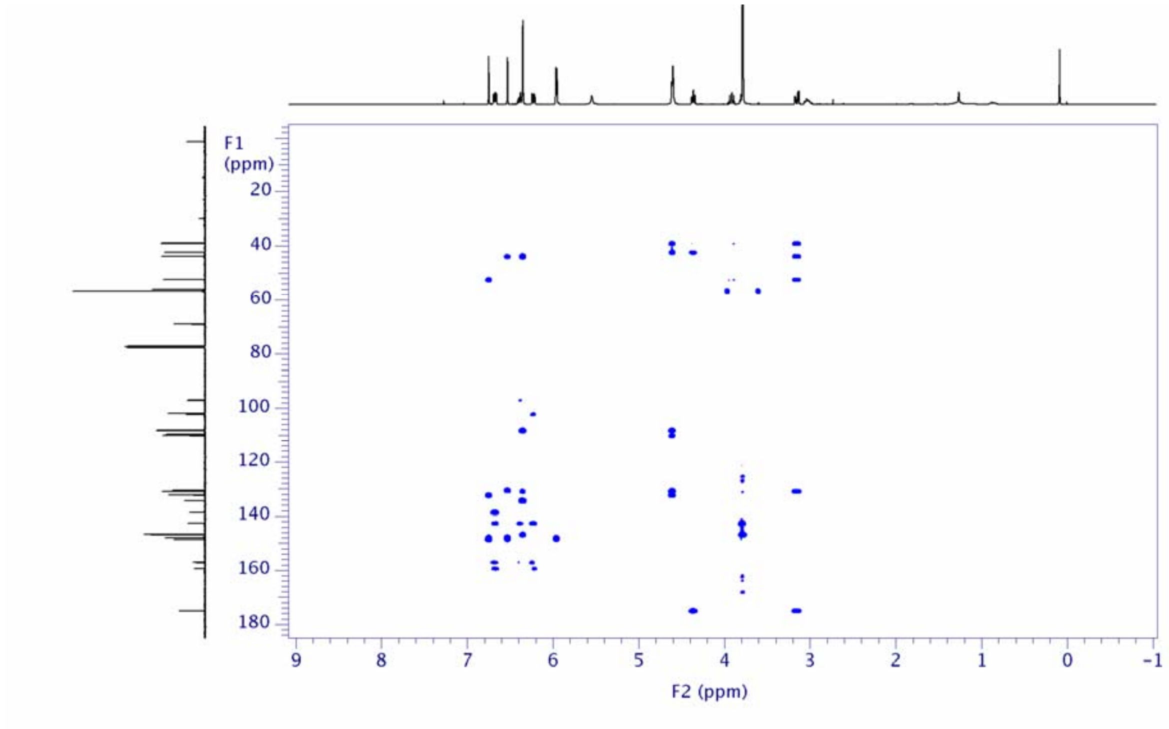

HSQC spectrums for Compound **16′**.

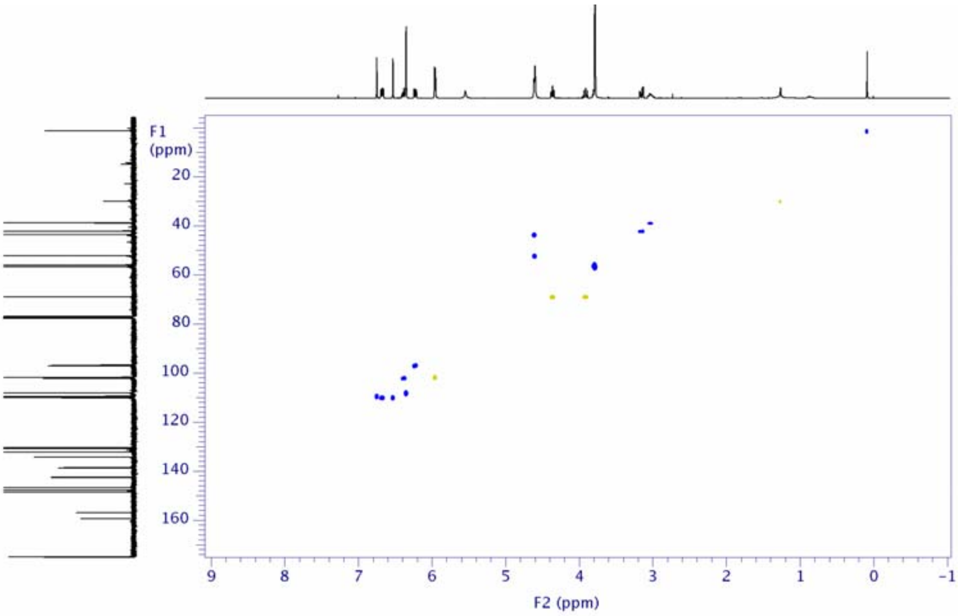

^{1}H-^{1}H COSY spectrums for Compound **16′**.

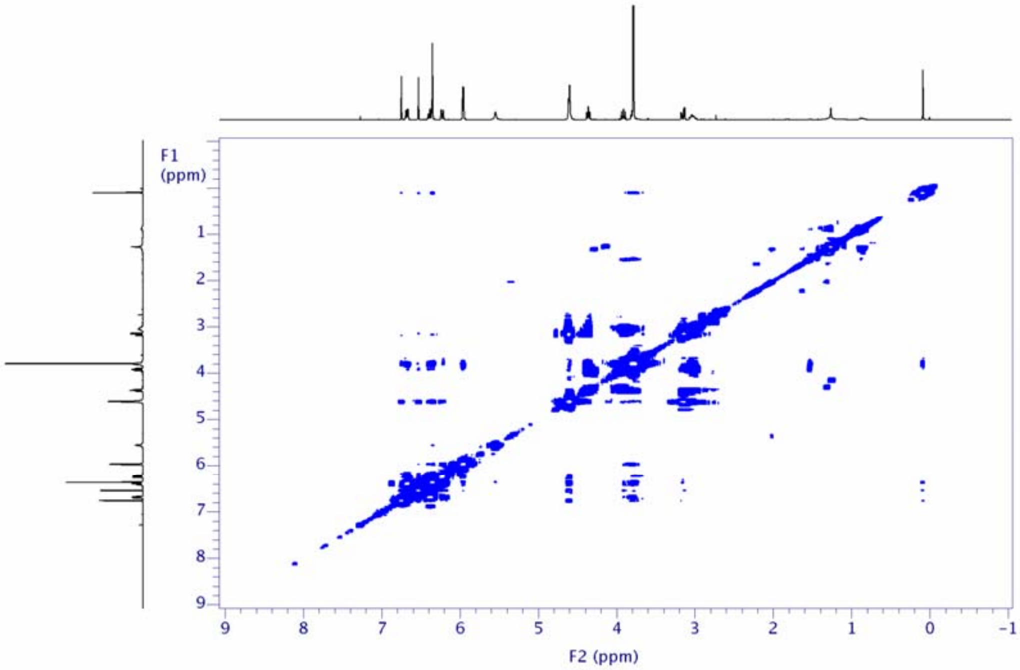

MS spectrums for Compound **16′**.

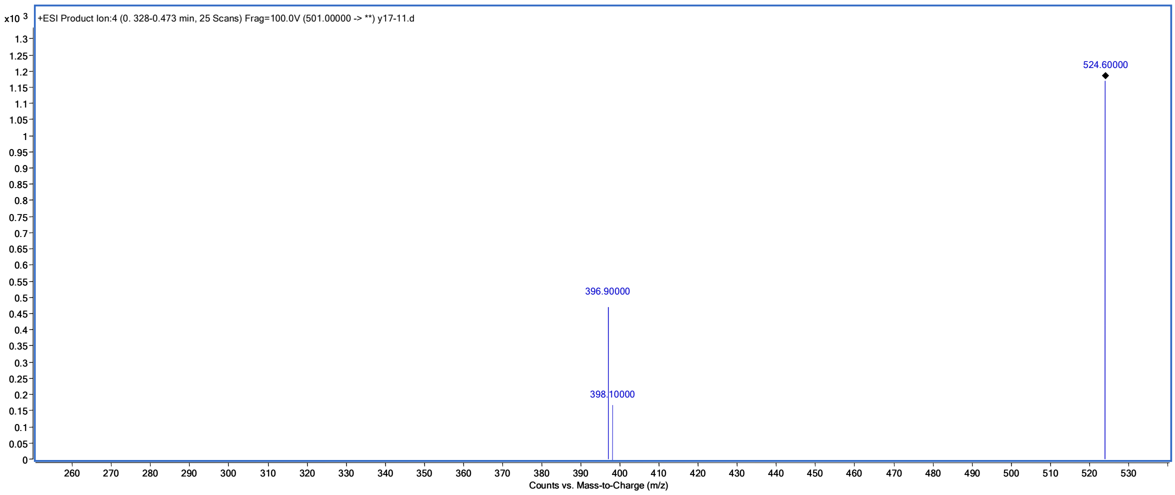

Compound 16′ (4*β*-*NH*-(5-fluoro-2-methoxyaniline)-4-deoxy-4′-demethylepipodophyllotoxin): 88% yield as white solid, ^{1}H NMR (300 MHz, $CDCl_3$): 2.953-2.987 (m, 1H, 2-H), 3.079 (dd, *J*=4.8 Hz, 1H, 3-H), 3.773 (s, 6H, 3′, 5′-OCH_3), 3.826 (s, 3H, Ar-OCH_3) 3.971 (t, J=7.8 Hz, 1H, 11-H), 4.354 (t, *J*=7.5 Hz, 1H, 11-H), 4.552 (s, 2H, 4-H, 1-H), 5.932 (d, J=7.8 Hz 2H, OCH_2O), 6.226 (d, J=9.0 Hz, 1H, ArH), 6.314 (s, 2H, ArH) , 6.367 (s, 1H, ArH), 6.502 (s, 1H, ArH), 6.744 (s, 1H, ArH), 6.853 (t, *J*=9.0 Hz, 1H, ArH) ^{13}C NMR (75 MHz, $CDCl_3$):δ 38.810 , 42.090, 43.619, 53.400, 56.695, 57.704, 69.073, 101.533, 107.425, 108.137, 109.280, 110.156, 116.286, 130.712, 130.816, 132.107, 134.289, 142.779, 146.682, 147.810, 148.478, 175.105; MS (ESI): m/z: 524 $[M+H]^+$

^{13}C NMR spectrum of 4*β*-*NH*-(3-bromo-4-methoxyaniline)-4-deoxy-4′-demethylepipodophyllotoxin **(17′)**.

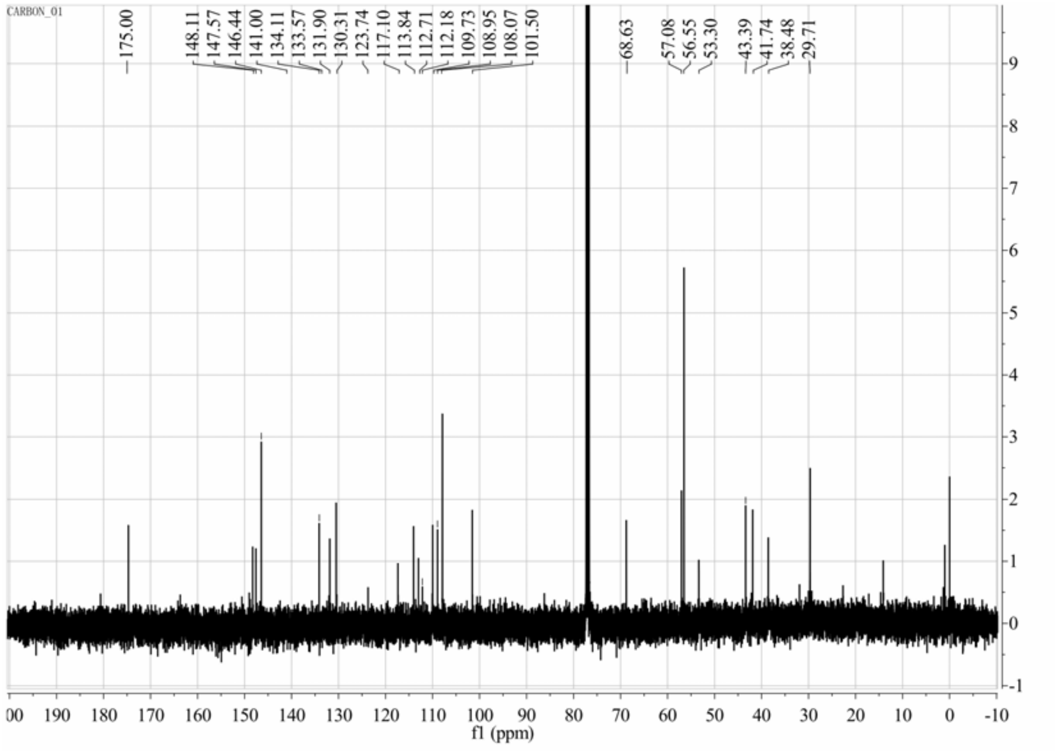

^{1}H NMR spectrum of Compound **17′**.

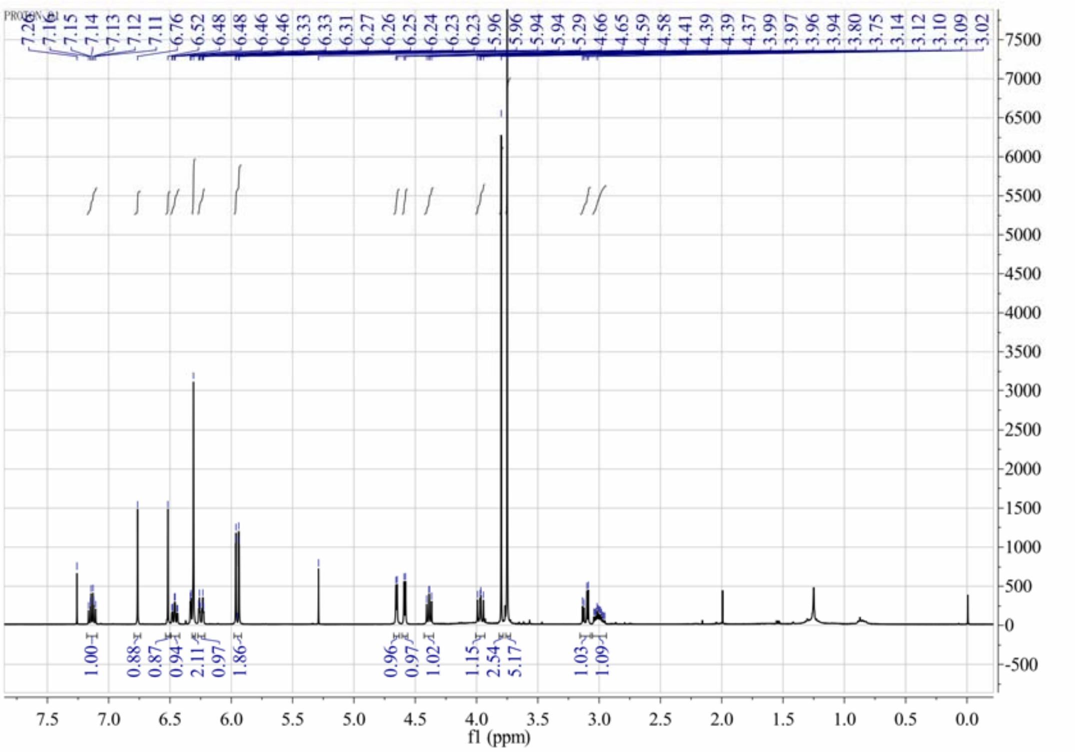

HMBC spectrums for Compound **17′**.

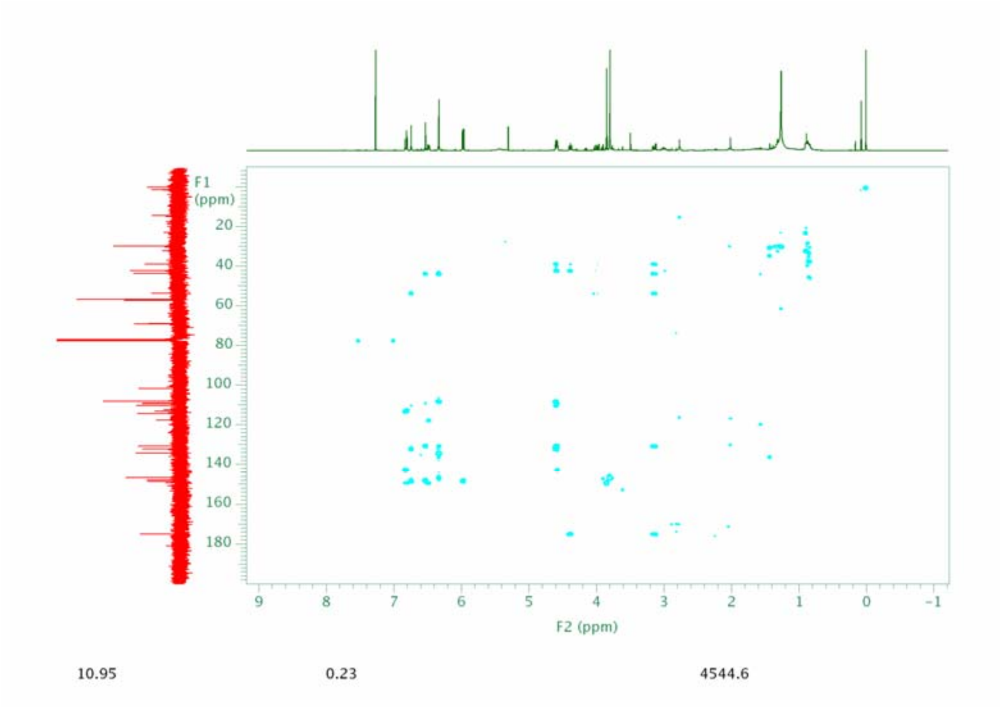

HSQC spectrums for Compound **17′**.

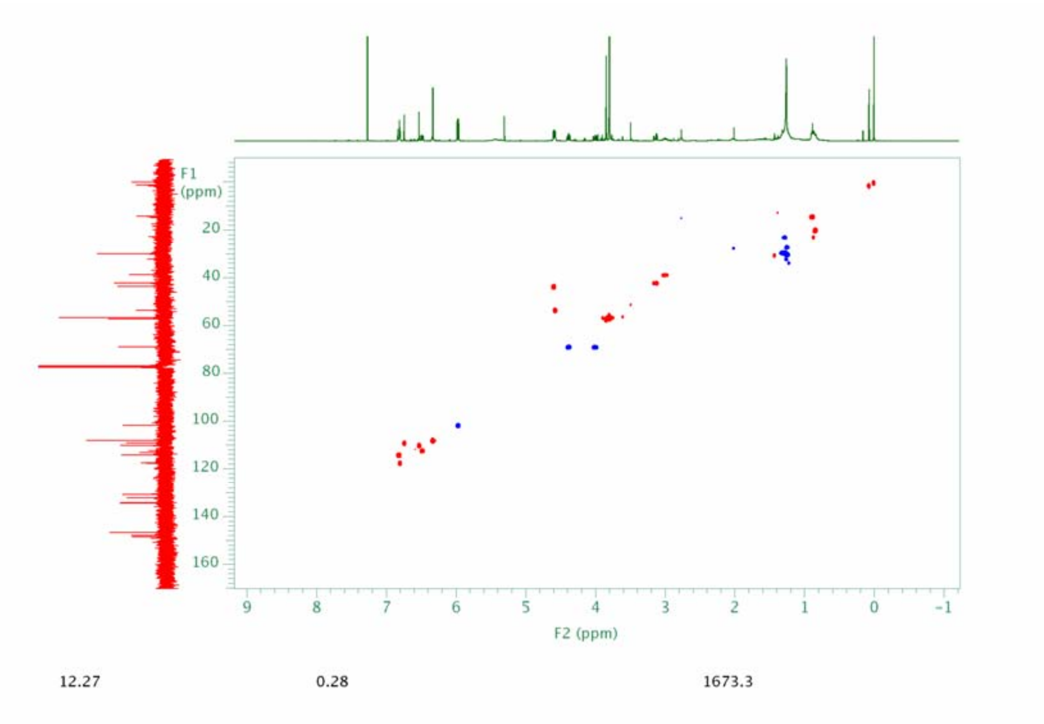

^{1}H-^{1}H COSY spectrums for Compound **17′**.

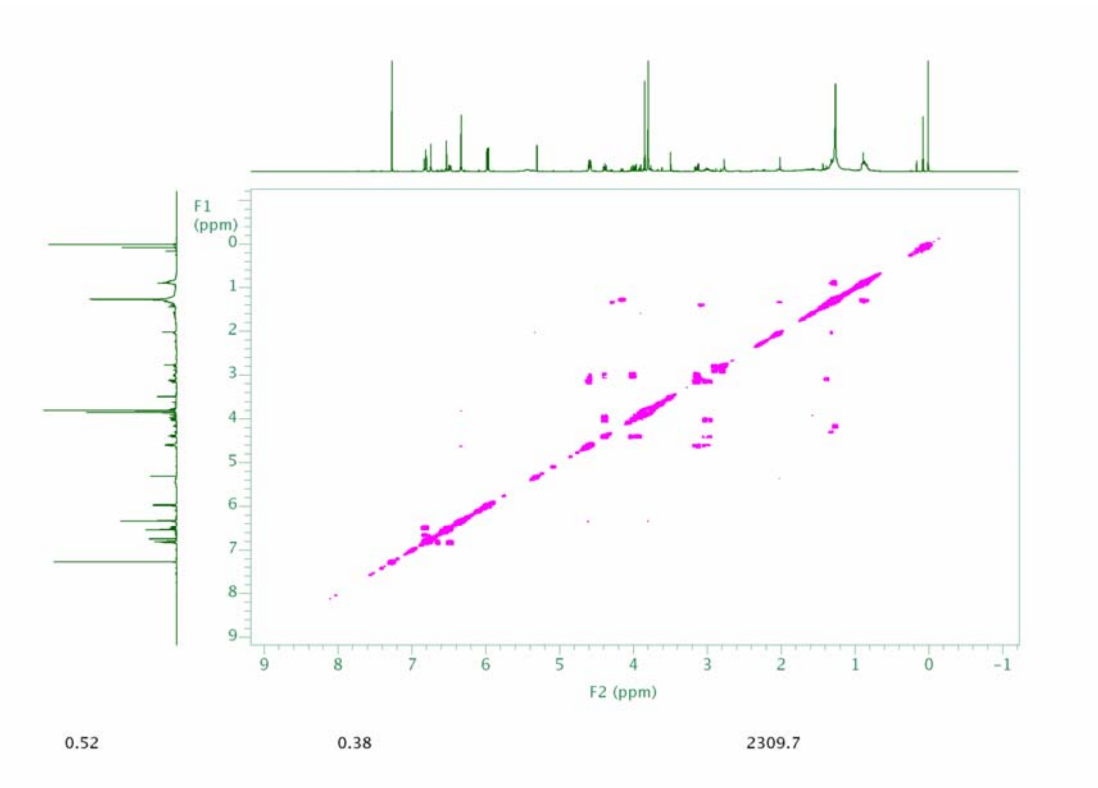

MS spectrums for Compound **17′**.

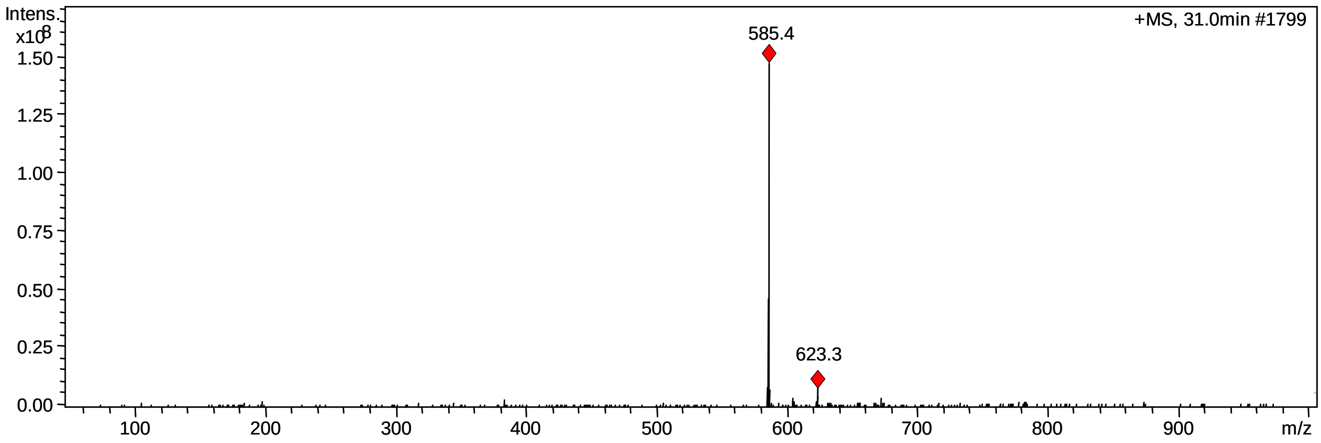

Compound 17′ (4β-*NH*-(3-bromo-4-methoxyaniline)-4-deoxy-4′-demethylepipodophyllotoxin): 88% yield as white solid, ^{1}H NMR (400 MHz, $CDCl_3$): δ2.96 (m, 1H, 2-H), 3.12 (dd, J=4.8 Hz, 1H, 3-H), 3.78 (s, 9H, 3′, 5′-OCH_3, ArOCH_3), 3.89 (t, *J*=8.0 Hz, 1H, 11-H), 4.37 (t, *J*=6.3Hz, 1H, 11-H), 4.56 (dd, *J*=4.0 Hz, 2H, 1H, 4-H), 5.95 (d, J=4.0 Hz, 2H, OCH_2O), 6.35 (s, 2H, ArH), 6.52 (s, 1H, ArH), 6.58 (s, 1H, ArH), 6.62 (s, 1H, ArH), 6.72 (s, 1H, ArH), 6.80 (dd, *J*=4.0 Hz, 1H, ArH), ^{13}C NMR (100 MHz, $CDCl_3$): δ38.63, 41.95, 43.41, 51.94, 55.58, 56.46, 68.77, 101.54, 107.94, 109.27,

109.83, 110.88, 111.43, 113.61, 119.58, 130.25, 130.58, 132.03, 134.05, 138.61, 145.31, 146.46, 147.53, 148.25, 174.82; MS (ESI): m/z: 585 $[M+H]^+$

^{13}C NMR spectrum of 4*β*-*NH*-(3-chloro-4-methoxyaniline)-4-deoxy-4′-demethylepipodophyllotoxin **(18′)**.

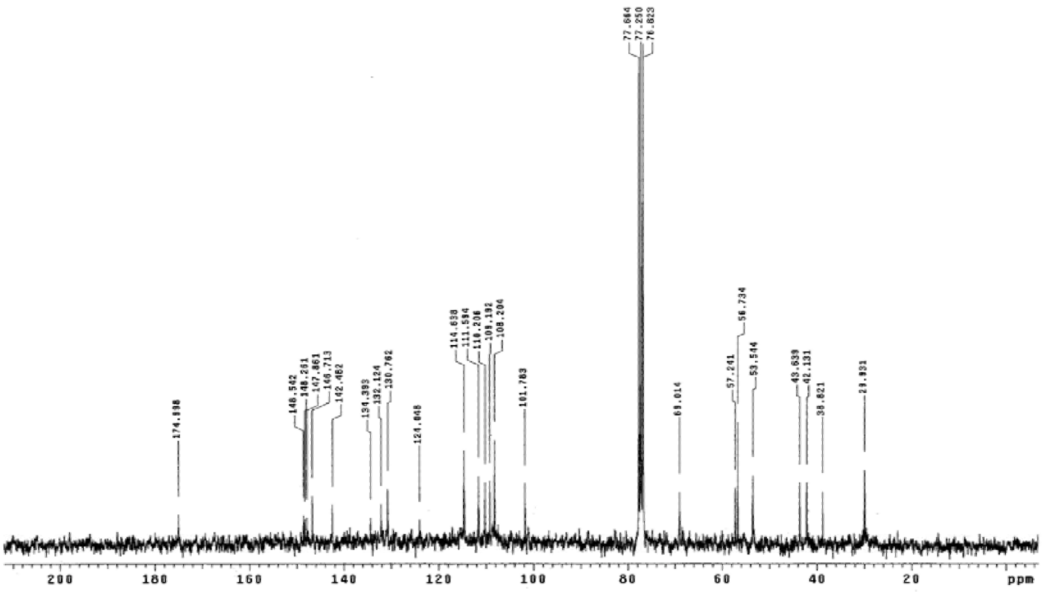

^1H NMR spectrum of Compound **18′**.

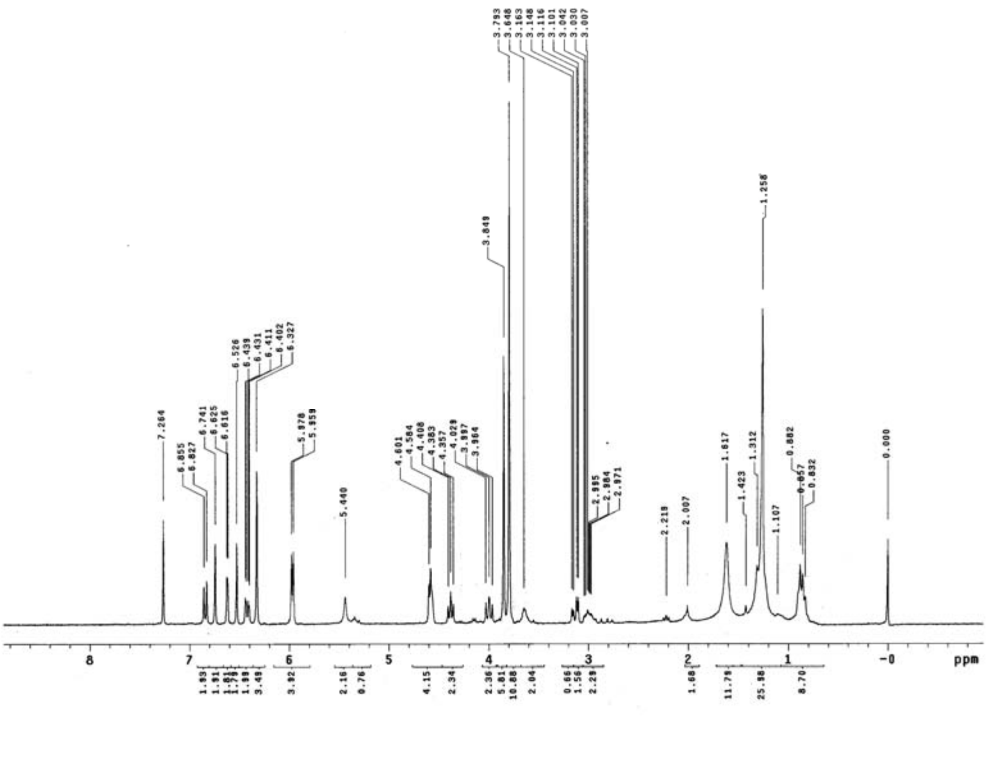

HMBC spectrums for Compound **18′**.

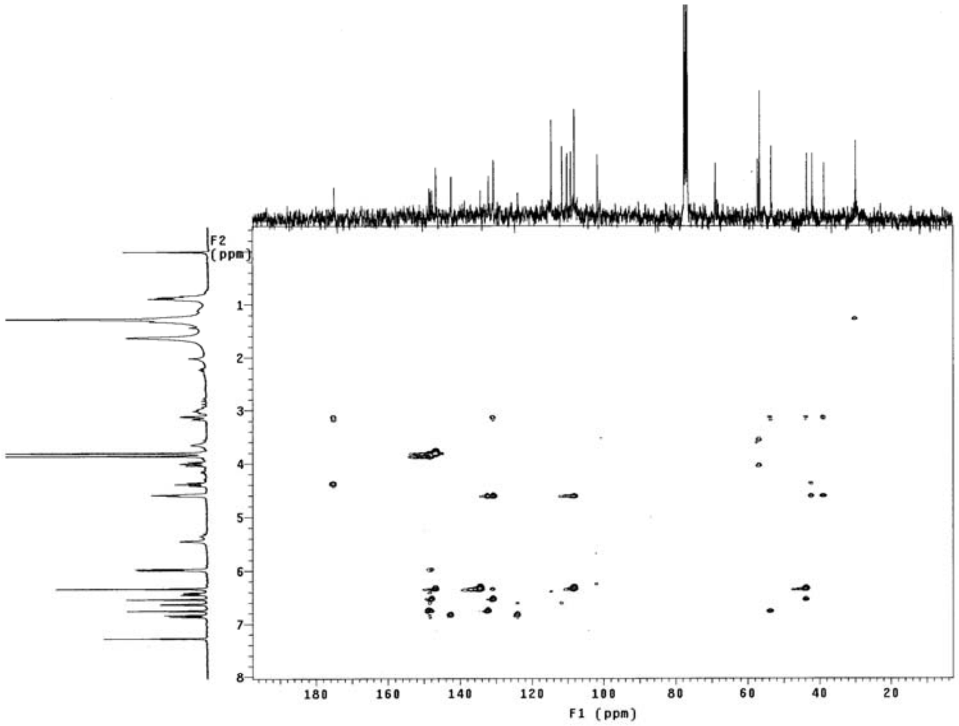

HSQC spectrums for Compound **18′**.

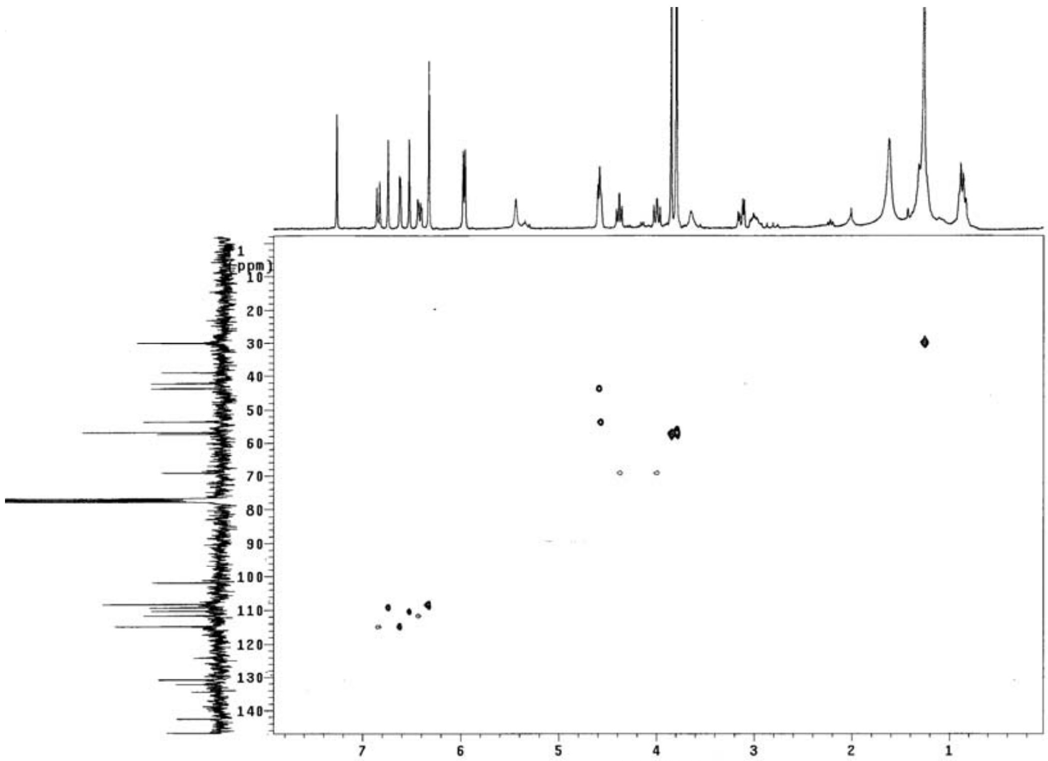

^{1}H-^{1}H COSY spectrums for Compound **18′**.

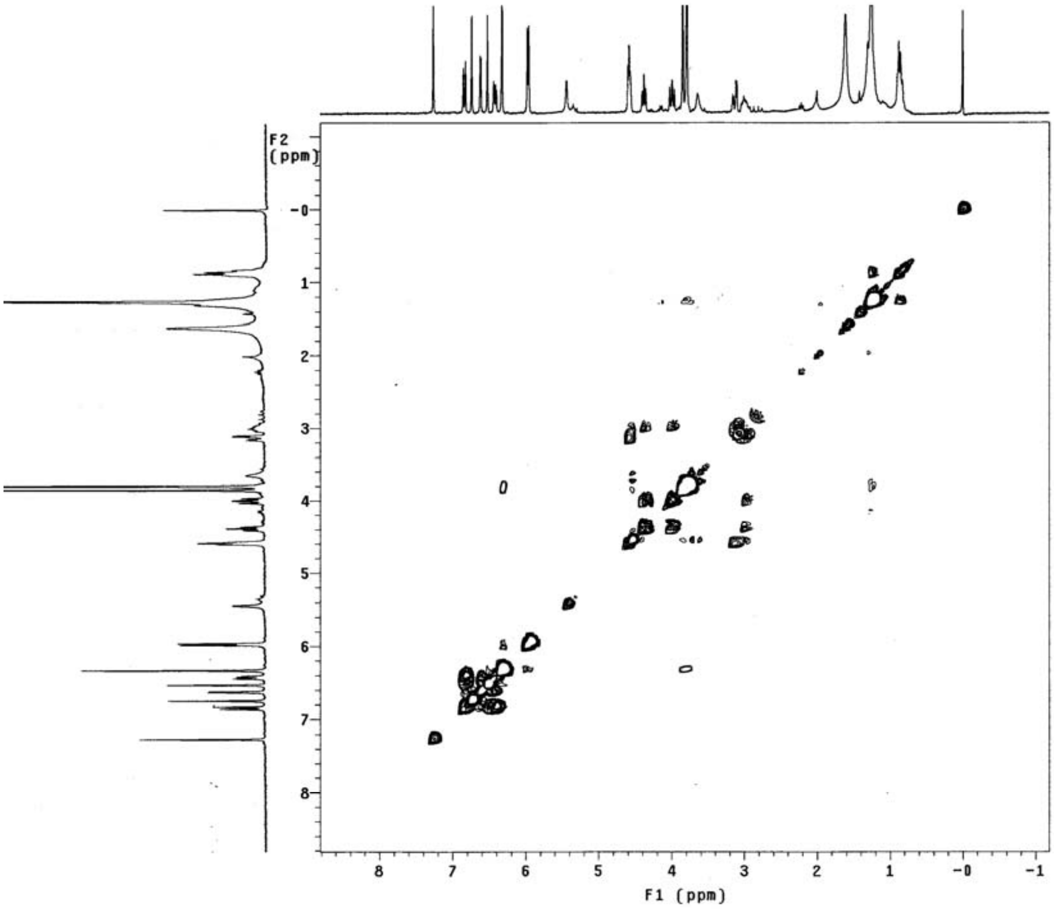

MS spectrums for Compound **18′**.

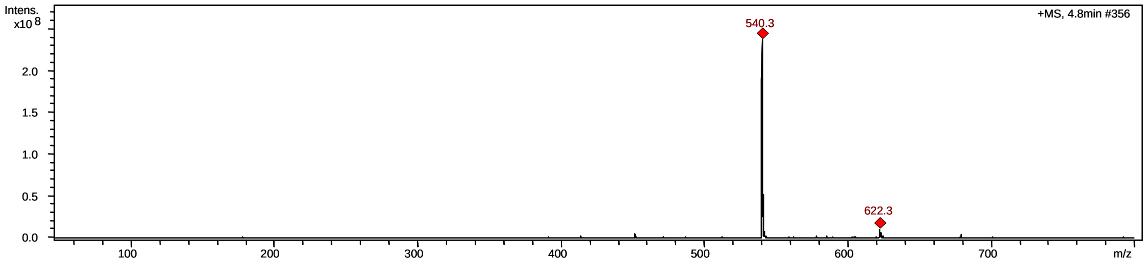

Compound 18′ (4β-*NH*-(3-chloro-4-methoxyaniline)-4-deoxy-4′-demethylepipodophyllotoxin): **5**6% yield as white solid, ^{1}H NMR (300 MHz, $CDCl_3$): δ3.039 (m, 1H), 3.114 (dd, *J*=4.8 Hz, 1H), 3.784 (s, 9H), 3.898 (t, *J*=9.3 Hz, 1H), 4.376 (t, *J*=7.8 Hz, 1H), 4.594 (d, *J*=5.1 Hz, 2H), 5.954 (d, *J*=3.0Hz, 2H), 6.333 (s, 2H), 6.436 (s, 1H) ,6.524 (s, 1H),6.672 (s, 2H), 6.728 (s, 1H) ^{13}C NMR (75 MHz, $CDCl_3$): δ38.863, 42.210, 43.674, 52.251, 55.863, 56.700, 69.001, 101.789, 108.121,

108.985, 109.501, 110.101, 110.547, 126.488, 130.505, 130.784, 132.276, 134.284, 138.524, 145.065, 146.682, 147.798, 148.523, 175.008; MS (ESI): m/z: 541 $[M+H]^+$

^{13}C NMR spectrum of 4*β*-*NH*-(3-fluoro-4-methoxyaniline)-4-deoxy-4′-demethylepipodophyllotoxin

(19′).

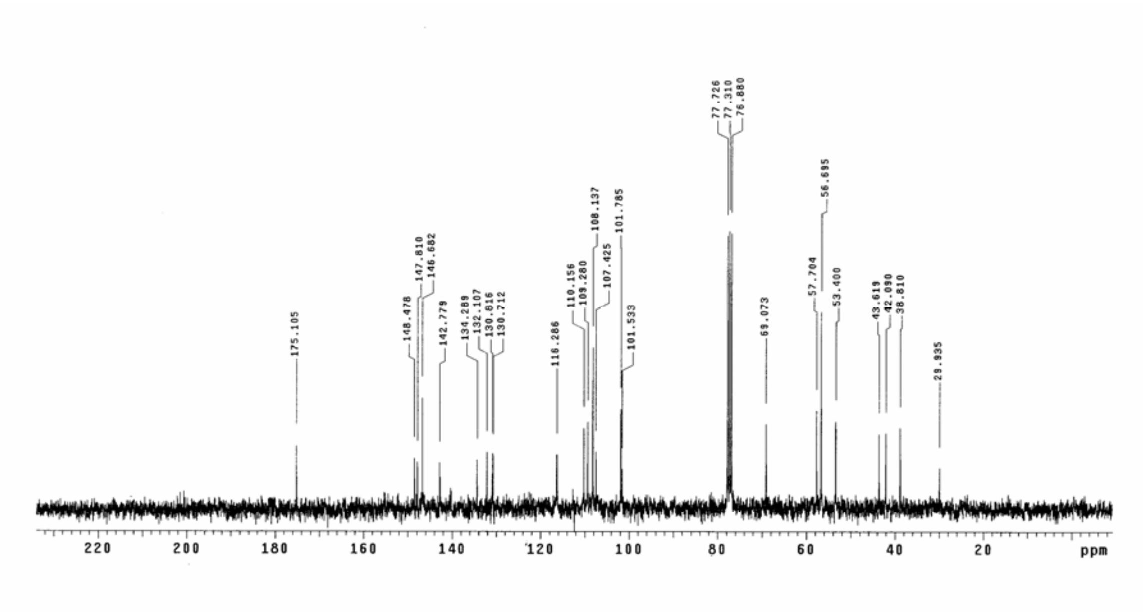

^1H NMR spectrum of Compound **19′**

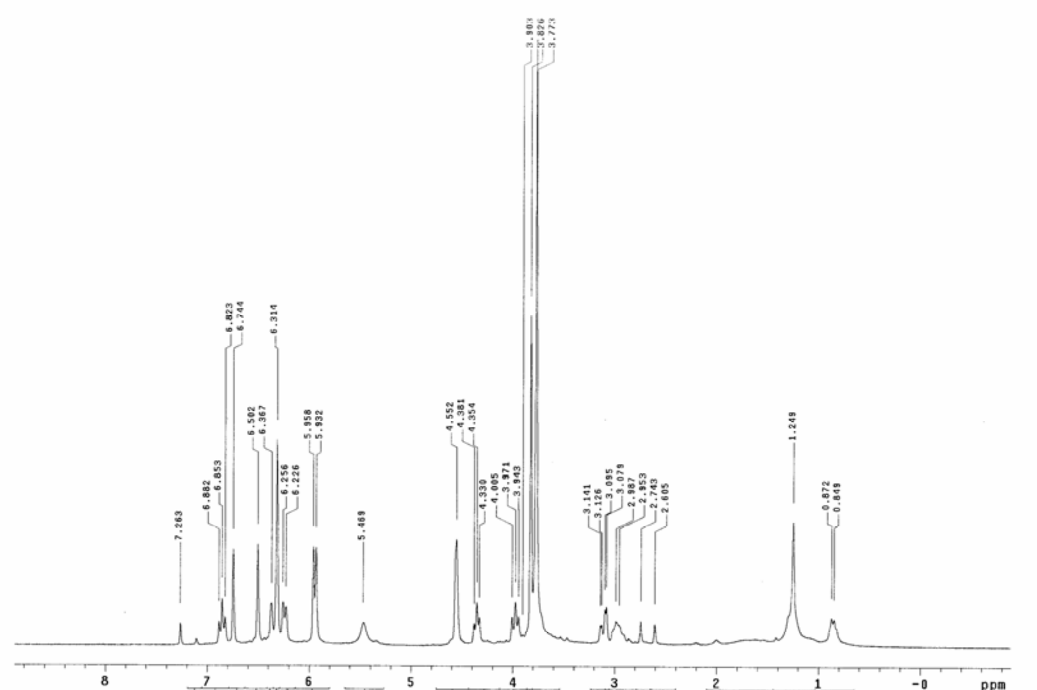

HSQC spectrums for Compound **19′**.

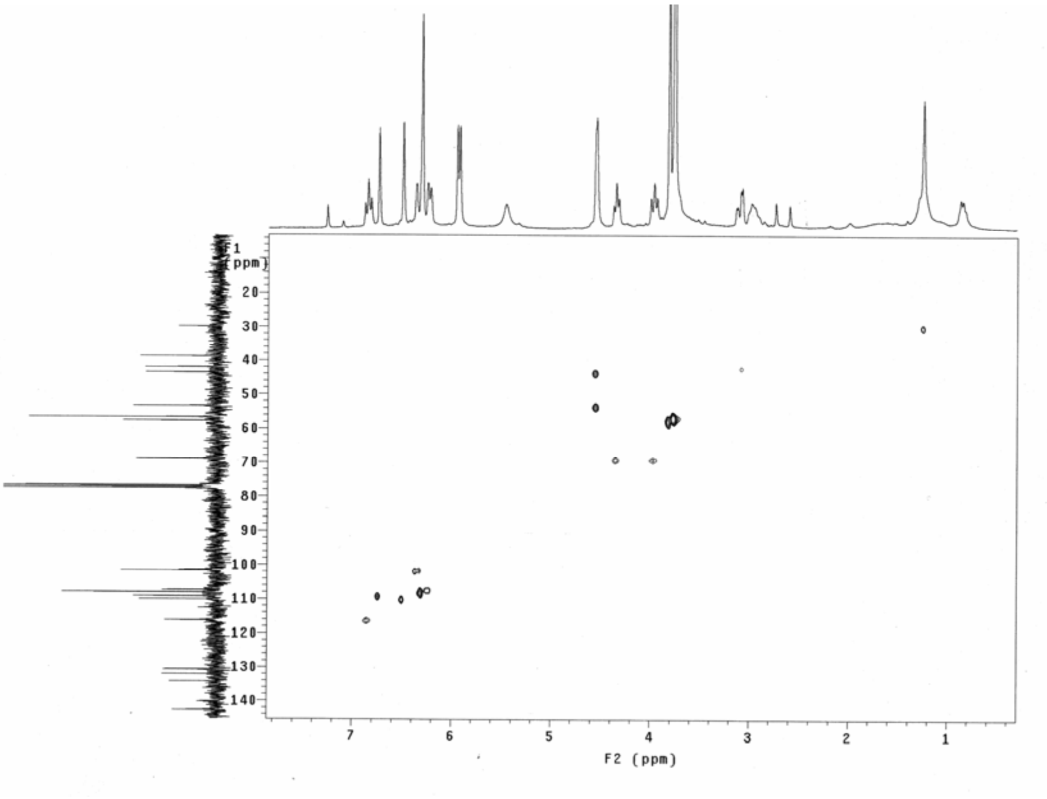

^{1}H-^{1}H COSY spectrums for Compound **19′**.

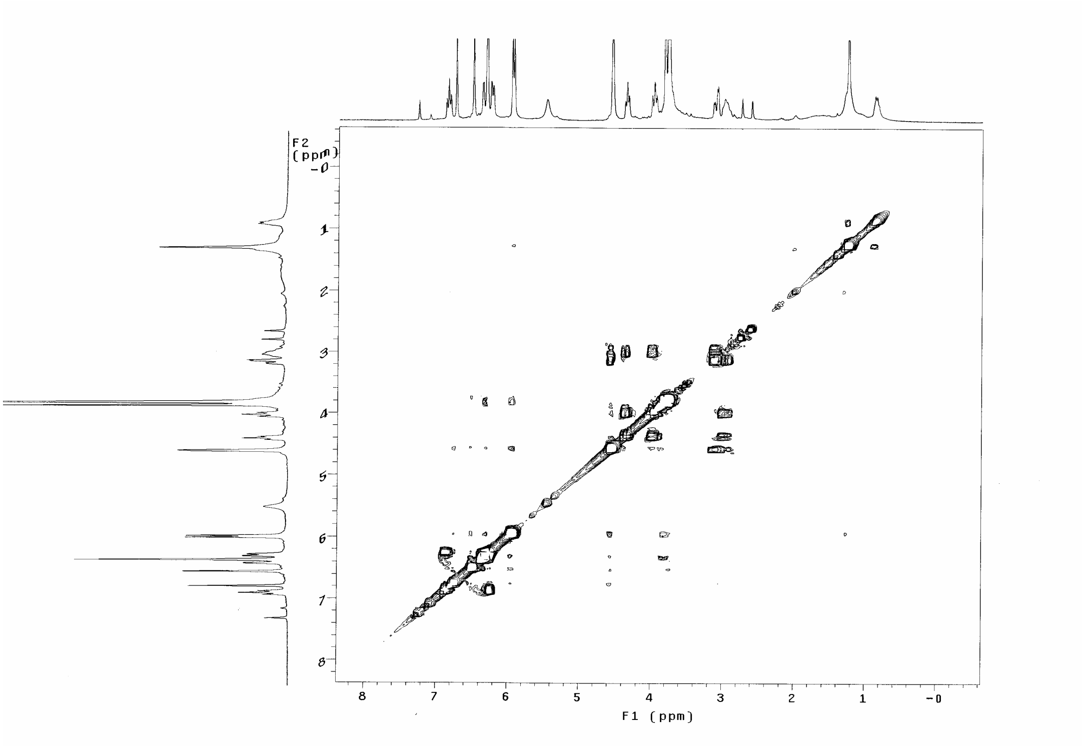

MS spectrums for Compound **19′**.

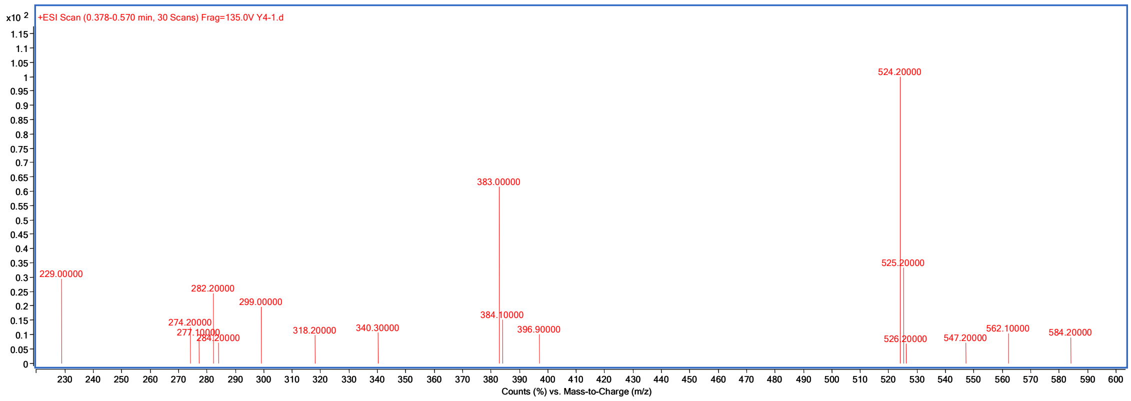

Compound 19′ (4*β*-*NH*-(3-fluoro-4-methoxyaniline)-4-deoxy-4′-demethylepipodophyllotoxin): **43**% yield as white solid, ^{1}H NMR (400 MHz, $CDCl_3$): δ 3.01 (m, 1H, 2-H) , 3.13 (dd, *J*=4.0Hz, 1H, 3-H), 3.78 (s, 9H, 3′, 5′-OCH_3, Ar-OCH_3), 3.91 (t, *J*=8.0 Hz, 1H, 11-H), 4.383 (t, *J*=8.0Hz, 1H, 11-H), 4.60 (d, *J*=4.0Hz, 2H,1-H, 4-H), 5.95 (d, *J*=4.0Hz, 2H, OCH_2O), 6.21 (dd, J=4.0Hz, 1-H, ArH), 6.35 (s, 2H, ArH), 6.37 (dd, *J*=4.0 Hz, 1H, ArH) , 6.40 (s, 1H, ArH), 6.65 (dd, *J*=4.0 Hz, 1H, ArH) , 6.74 (s, 1H, ArH), ^{13}C NMR (100 MHz, $CDCl_3$): δ 38.62, 41.93, 43.42, 52.06, 55.81, 56.45, 68.74, 96.90, 101.76,, 107.93, 109.28, 109.79, 109.84, 109.89, 130.26, 130.59, 132.03, 134.06, 138.34, 138.45, 142.31, 142.33, 146.46, 147.55, 148.26, 156.78, 159.13, 174.77; MS (ESI): m/z: 524 $[M+H]^+$
